# A Compact Reprogrammed Genetic Code for De Novo Discovery of Proteolytically Stable Thiopeptides

**DOI:** 10.1021/jacs.3c12037

**Published:** 2024-03-16

**Authors:** Alexander A. Vinogradov, Yue Zhang, Keisuke Hamada, Shunsuke Kobayashi, Kazuhiro Ogata, Toru Sengoku, Yuki Goto, Hiroaki Suga

**Affiliations:** † Department of Chemistry, Graduate School of Science, The University of Tokyo, Bunkyo-ku, Tokyo 113-0033, Japan; ‡ Department of Biochemistry, Graduate School of Medicine, Yokohama City University, Kanazawa-ku, Yokohama 236-0004, Japan

## Abstract

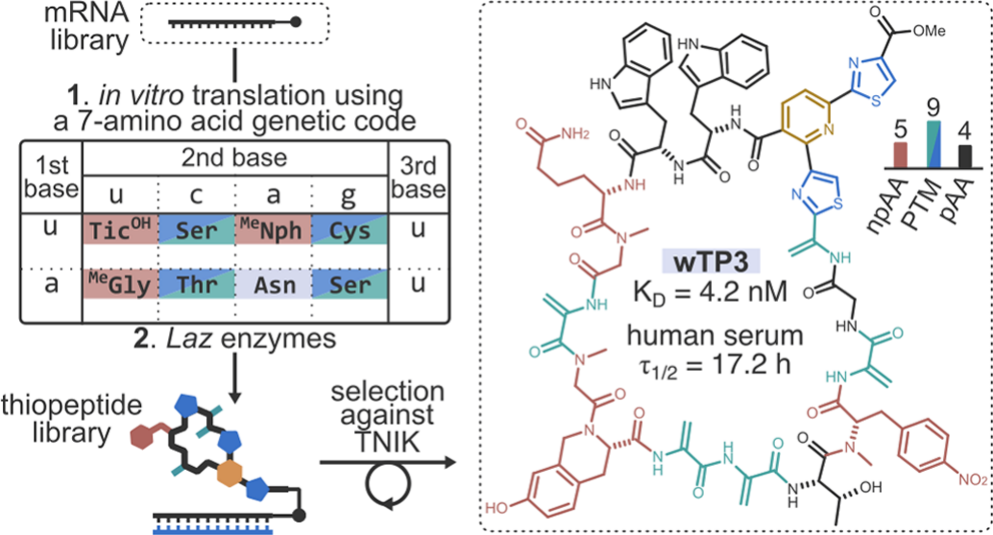

Thiopeptides make up a group of structurally complex peptidic natural products holding promise in bioengineering applications. The previously established thiopeptide/mRNA display platform enables de novo discovery of natural product-like thiopeptides with designed bioactivities. However, in contrast to natural thiopeptides, the discovered structures are composed predominantly of proteinogenic amino acids, which results in low metabolic stability in many cases. Here, we redevelop the platform and demonstrate that the utilization of compact reprogrammed genetic codes in mRNA display libraries can lead to the discovery of thiopeptides predominantly composed of nonproteinogenic structural elements. We demonstrate the feasibility of our designs by conducting affinity selections against Traf2- and NCK-interacting kinase (TNIK). The experiment identified a series of thiopeptides with high affinity to the target protein (the best *K*_D_ = 2.1 nM) and kinase inhibitory activity (the best IC_50_ = 0.15 μM). The discovered compounds, which bore as many as 15 nonproteinogenic amino acids in an 18-residue macrocycle, demonstrated high metabolic stability in human serum with a half-life of up to 99 h. An X-ray cocrystal structure of TNIK in complex with a discovered thiopeptide revealed how nonproteinogenic building blocks facilitate the target engagement and orchestrate the folding of the thiopeptide into a noncanonical conformation. Altogether, the established platform takes a step toward the discovery of thiopeptides with high metabolic stability for early drug discovery applications.

## Introduction

Thiopeptides are a large group of structurally complex ribosomally synthesized and post-translationally modified peptide (**RiPP**) natural products.^1−3^ The group is defined by the presence of a nitrogen-containing heterocycle (usually pyridine) grafted into the backbone of a macrocyclic peptide. Most thiopeptides are also heavily functionalized by Cys/Ser/Thr-derived azol(in)e moieties and dehydroamino acids [**dhAAs**; dehydroalanine (**Dha**) and (Z)-dehydrobutyrine (**Dhb**) derived from Ser and Thr, respectively], which confer considerable rigidity and hydrophobicity to the macrocycles. Despite the limited repertoire of building blocks comprising the structures, thiopeptides are renowned for their diverse biological activities.^3^ They act as bacterial protein synthesis inhibitors targeting either the ribosome^4,5^ or EF-Tu;^6,7^ possess anticancer activities via the inhibition of transcription factor Fox-M1^8,9^ and/or the 20S proteasome;^10,11^ induce mitophagy in mammalian cell models;^12^ and inhibit renin,^13^ a protease that regulates blood pressure, among many other activities.^14^ Accordingly, the utilization of thiopeptide scaffolds in medicinal chemistry has garnered considerable attention.^15−23^ Two semisynthetic thiopeptide derivatives entered clinical trials as antibiotic agents,^24,25^ and numerous research groups reengineered thiopeptide biosynthesis to improve their bioactivities and pharmacological properties.^26−32^

**Figure 1:**
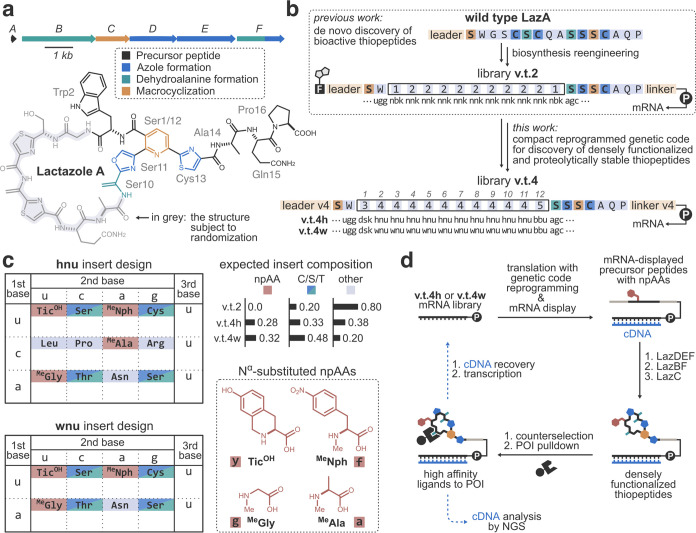
Design of the thiopeptide/mRNA display platform with a compact reprogrammed genetic code. (a) Biosynthetic gene cluster and the chemical structure of lactazole A, a thiopeptide whose reengineered biosynthesis serves as the foundation for the discovery platform. (b) Reengineering of lactazole biosynthesis. Previous work^33^ reported the construction of mRNA display library v.t.2. In this work, the libraries are redesigned to accommodate compact reprogrammed genetic codes for the discovery of densely functionalized thiopeptides. See also Figure S2 for the complete description of v.t.4 library designs. (c) Reprogrammed genetic code tables for translation. Library v.t.4h and v.t.4w contain random inserts composed of hnu- and wnu-encoded amino acids, respectively. These degenerate codons, particularly wnu, lead to a high density of C/S/T residues and npAAs in the thiopeptide inserts compared to the previously reported^33^ library v.t.2. (d) Selection scheme. mRNA-barcoded precursor peptides are produced using the constructed translation system, and upon sequential three-step treatment with LazDEF/LazBF/LazC are converted to thiopeptides. The resulting library is subjected to a pulldown against immobilized TNIK to enrich for high-affinity protein ligands. The process is repeated by recovering cDNA, amplifying it by PCR, and transcribing it to mRNA for the following round of selection. POI: protein of interest.

Previously, we established an mRNA display-based platform for the *de novo* discovery of functional natural product-like thiopeptides.^33−35^ The platform leverages a reengineered *in vitro* biosynthesis of lactazole A, a thiopeptide from *Streptomyces lactacystinaeus* (Figure 1a).^36^ To access large (>10^12^ unique compounds) libraries of lactazole-like thiopeptides, partially randomized and mRNA-barcoded precursor peptides (LazA variants) are produced with the flexible *in vitro* translation (**FIT**) system,^37^ and are then treated with lactazole biosynthetic enzymes (LazBCDEF) to convert the linear precursors into macrocyclic thiopeptides for downstream affinity selections. The precursor libraries contain a random insert between the two islets of amino acids indispensable for maturation (Ser1–Trp2 and Ser10–Ser11–Ser12–Cys13–Ala14; Figure 1a,b). Previously,^33^ we utilized this platform to identify a series of thiopeptides acting as potent and selective inhibitors of Traf2- and NCK-interacting kinase (**TNIK**), a protein implicated in several forms of cancer.^38−40^ However, the discovered thiopeptides featured random inserts composed predominantly of proteinogenic amino acids, which limited their likeness to natural products and led to modest metabolic stabilities. For instance, the most active discovered inhibitor, TP15, degraded in human serum, with a half-life (τ_1/2_) of only 1.8 h.

Here, we address these issues by developing a discovery platform that yields bioactive thiopeptides with high metabolic stabilities (Figure 1b–d). To drastically increase the density of nonproteinogenic elements in library thiopeptides, we employed compact (or reduced) genetic codes, i.e., translation tables encoding a limited number of distinct amino acids. The translation tables designed herein contained only seven or 11 amino acids to improve the number of enzymatically installed post-translational modifications (**PTM**s). Additionally, *in vitro* genetic code reprogramming was utilized for the ribosomal incorporation of nonproteinogenic amino acids (**npAA**s) in the random insert. To this end, we constructed the FIT system with a compact reprogrammed genetic code containing four npAAs; developed a new protocol for the enzymatic maturation of LazA variants; designed new thiopeptide precursor libraries; and finally performed affinity selections against TNIK. The selections identified six potent thiopeptide ligands, five of which inhibited the kinase in sub- or single-digit μM concentrations. As designed, the discovered compounds consisted chiefly of nonproteinogenic elements (up to 15 npAAs/PTMs in an 18-residue macrocycle) and consequently had a high metabolic stability in human serum. X-ray structural analysis of the interaction between TNIK and one of the discovered thiopeptides, wTP3, pointed to a predominantly hydrophobic association driven by the steric complementarity between the protein and ligand. Altogether, these advances enable the *de novo* discovery of biologically active thiopeptides which approach their natural counterparts in terms of the density of privileged nonproteinogenic elements and the high metabolic stability.

## Results and Discussion

### Construction of the Selection Platform

**Figure 2:**
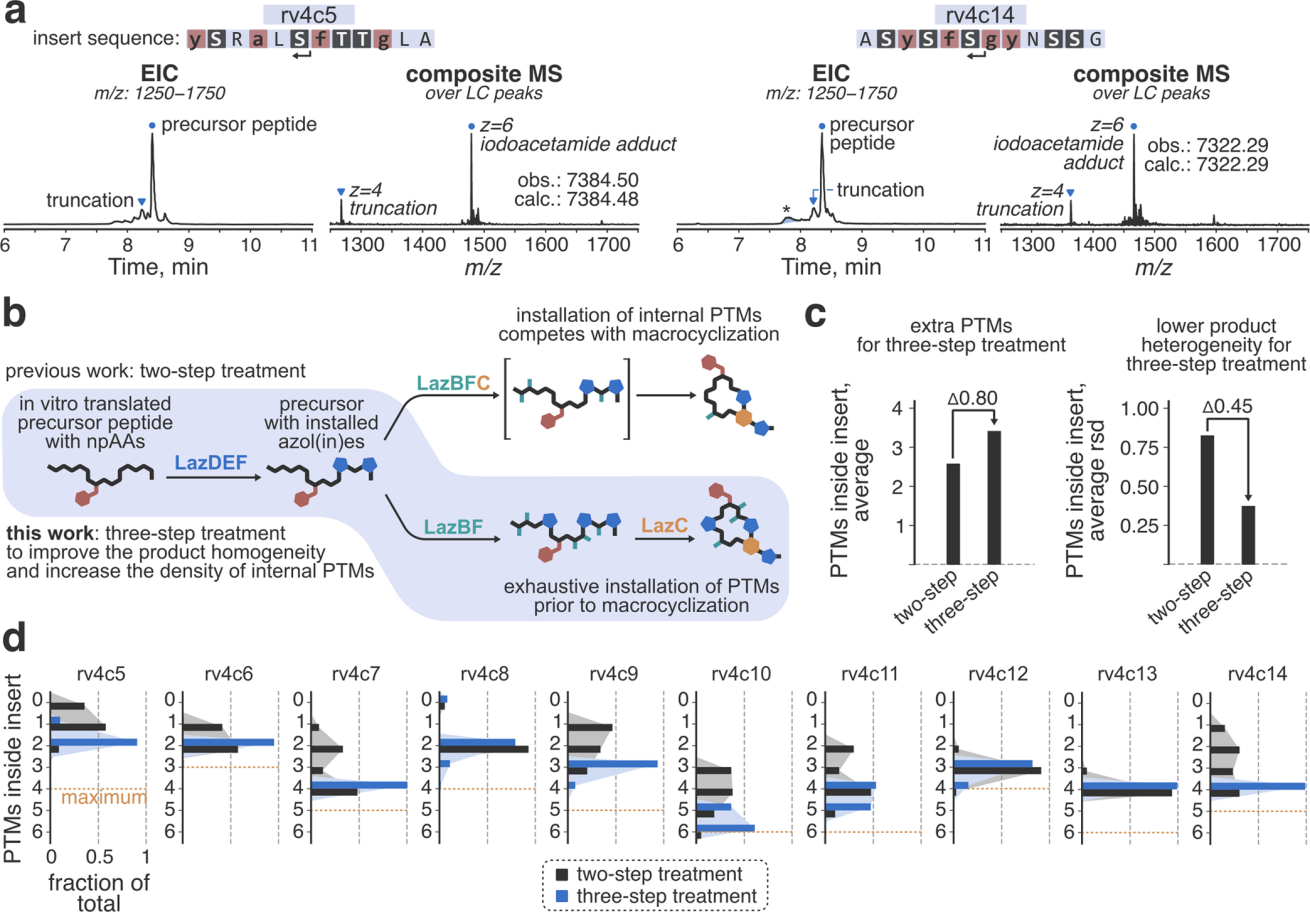
Construction of the selection platform. (a) Translational incorporation of multiple npAAs into partially randomized LazA variants. Peptides rv4c5 and rv4c14 were expressed with the FIT system using the reprogrammed genetic code (S. I. 2.6), and the translation outcomes were analyzed by LC/MS. Displayed are extracted ion current (EIC) chromatograms and composite MS spectra integrated over peptide-derived peaks. Formation of the expected peptides as the major product was observed in both cases. The left arrows shown below the insert sequences indicate the location of the truncations. *: translation protein carryover. (b) Development of a three-step thiopeptide maturation protocol, in which LazA-derived precursor peptides are sequentially treated with LazDEF/LazBF/LazC. (c) The use of the three-step protocol improves both the density of PTMs (average number of PTMs inside insert) and the homogeneity of the resulting products (average relative standard deviation of the number of PTMs inside insert) compared to the previously employed two-step treatment.^33^ The statistics are derived from the analysis of the rv4c5–14 maturation products by LC/MS ((d) and Figures S4–S13).

To build the selection platform, we first designed a reprogrammed genetic code that can express LazA precursors with multiple npAAs.^37^ Because LazA precursor peptides contain an indispensable 38 amino acid-long N-terminal sequence (leader peptide) utilized by Laz enzymes for substrate recognition (Figure S2), only four codon boxes (Tyr, His, Phe, and Lys) are available for genetic code reprogramming. Our preliminary experiments showed that the Lys codon is unsuitable for reprogramming due to the high rates of translational misincorporation. To increase the number of available codon boxes, we introduced an I4V mutation in the leader peptide. This conservative mutation liberated the Ile codon box for reprogramming and enabled the construction of a reprogrammed genetic code that incorporated four types of npAAs by reassignment of the Tyr, His, Phe, and Ile codon boxes. We chose N-Me-Gly (single letter abbreviation: **g**), N-Me-Ala (**a**), N-Me-Phe(*p*NO_2_) (**f**), and Tic(OH) (**y**) as suppressor amino acids (Figure 1c). These npAAs were previously shown to have high efficiency of translational incorporation.^41,42^ Additionally, N^α^-blocked amino acids improve lipophilicity (and hence cell uptake), provide conformational constraint, and increase the proteolytic stability of macrocyclic peptides.^43^ After some experimentation (data not shown), we arrived at the reprogrammed genetic code where the Ile codon (AUU) is decoded by N^Me^-Gly-tRNA^Pro1E2_GAU_^, the Tyr codon (UAU) by N^Me^-Nph-tRNA^Pro1E2^_GUA_, the His codon (CAU) by N^Me^-Ala-tRNA^Pro1E2_GUG_^, and the Phe codon (UUU) by Tic^OH^-tRNA^Pro1E2_GAA_^. We chose engineered tRNA body sequence (tRNA^Pro1E2^)^44^ as its use generally led to the highest translational fidelity compared to the alternatives. Thus, to conduct *in vitro* translation of npAA-containing LazA variants, tRNAs aminoacylated with npAAs by the use of flexizymes [25 μM each; Supporting Information (**S.I.**) 2.6; Table S3] were added to the translation mixture depleted of Ile, Tyr, His, and Phe amino acids. Under these conditions, LC/MS analysis pointed to the clean expression of single npAA-containing peptides with minimal translation side products (amino acid misincorporation and peptide truncation; Figure S3). Translation of ten LazA variants with multiple npAAs inside the insert (rv4c5–14) also yielded the expected peptides as the major product in every case (Figures 2a and S4–S13), although in three cases, appreciable amounts of truncated peptides were observed. We deemed the occasional formation of truncated sequences a minor inconvenience because, during mRNA display, such byproducts are not barcoded by mRNA, and as such, remain invisible in affinity selections.

Next, we turned to biosynthesis reengineering. Random inserts in LazA-based libraries may contain Cys/Ser/Thr residues (C/S/T), which can be modified by Laz enzymes to azol(in)es and dhAAs. Our previously reported selection led to the identification of 15 hit thiopeptides, 13 of which contained at least one C/S/T residue in the random insert.^33^ However, clean enzymatic installation of PTMs occurred only in three cases. Formation of product mixtures (undesirable because it complicates the identification of active species) due to the partial modification of insert C/S/Ts was also observed for four peptides. To improve the density and homogeneity of PTMs inside the random inserts, we redesign the enzymatic incubation conditions. Our previously employed^33^ two-step enzyme protocol featured the treatment of the precursor library with LazDEF (azole formation)^45^ followed by LazBCF (dhAAs formation^46^ and macrocyclization^47^). In this setup, insert C/S/T residues might undergo PTMs, although once the minimal set of PTMs for the lactazole scaffold is installed (i.e., Dha1 and Dha10-Oxz11-Dha12-Thz13, where **Oxz** stands for oxazole and **Thz** for thiazole),^34^ the pyridine synthase LazC can terminate the biosynthesis by cleaving the leader peptide, thus preventing further leader-dependent azole/dhAA modifications. We reasoned that adding LazC after LazBF and LazDEF are given sufficient time to act should produce more extensively modified thiopeptides and also alleviate the formation of product mixtures (Figure 2b). To test this hypothesis, we *in vitro* translated C/S/T-rich LazA variants rv4c5–14 and tested their maturation using either the redesigned three-step maturation protocol [a sequential addition of LazDEF (3 h reaction), LazBF (4 h), and LazC (3 h)] or the earlier two-step incubation [LazDEF (3 h) followed by LazBCF (7 h)] (S. I. 2.3 and 2.6). LC/MS analysis of the thiopeptide product distributions (Figures 2c,d and S4–S13) showed that the three-step reaction improved the extent of insert modification (3.4 vs 2.6 insert PTMs on average) and the homogeneity of the resulting products (average relative standard deviation of the number of insert PTMs: 0.37 vs 0.82) without affecting the macrocyclization efficiency. In other words, the redesigned three-step maturation was superior to the previous protocol along both analyzed dimensions.

To take full advantage of the reprogrammed FIT system and the redesigned maturation protocol, we designed new LazA-based precursor libraries (Figures 1b,c and S2). To maximize the probability of finding nonproteinogenic elements in the insert regions (translationally installed npAAs and enzymatic PTMs on C/S/T residues), we decided to explore the use of compact genetic codes. Compact (or reduced) genetic codes decrease the number of amino acids in the alphabet. Notwithstanding the low diversity of combinatorial peptide/protein libraries derived from such alphabets, compact genetic codes were previously employed in affinity selections with great success.^48−51^ Here, we designed two mRNA libraries by changing the size of the amino acid alphabet inside the random insert of LazA variants. The more diverse of the two, library **v.t.4h** featured an insert composed of repeating hnu degenerate codons, which encode 11 amino acids each (Figure 1b,c). There is a 1/3 probability of an npAA or C/S/T residue in every hnu position. The library **v.t.4w** contained an even smaller alphabet containing seven amino acids accessed via wnu codons, which encoded an npAA with a 3/8 chance and a C/S/T residue with a 1/2 probability. Both v.t.4h and v.t.4w libraries bore conservative dsk and bbu degenerate codons in the positions flanking the fixed modification sites (positions 3 and 12) to ensure efficient macrocyclization.^52^ Other changes compared to the previously reported library v.t.2 included the aforementioned I4V mutation in the leader peptide, and the redesign of the C-terminal linker to liberate the Tyr codon box for genetic code reprogramming (Figure S2). Our thesis with these designs was that during affinity selections, the relatively low diversity of the resulting libraries (1.8 × 10^12^ and 1.8 × 10^10^ theoretical peptides for v.t.4h and v.t.4w, respectively) can be compensated by the use of privileged building blocks, similar to how natural thiopeptides derive their diverse bioactivities from a handful of constituents.

### Affinity Selection against TNIK

**Figure 3:**
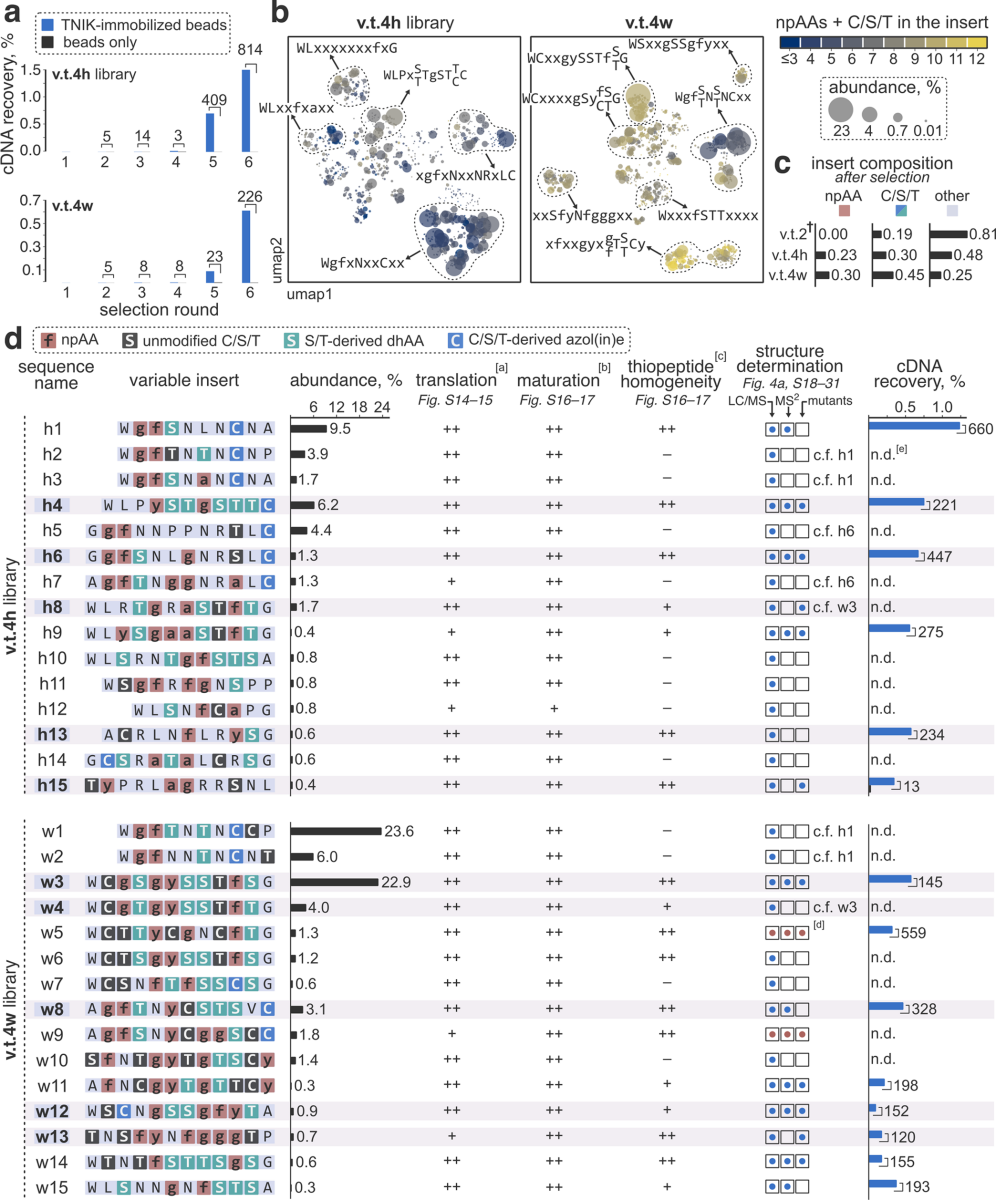
Results of the affinity selections against TNIK. (a) The progress of the selections as monitored by the cDNA recovery rate. Numbers above the bars indicate the ratio of cDNA recovery between TNIK pulldown and counterselection fractions. (b) Uniform manifold approximation and projection (umap) embedding of top 1000 most abundant random insert sequences obtained after six rounds of selection. The sequences are colored according to the number of npAAs and C/S/T residues in the insert. (c) Insert compositions in round six libraries. As designed, libraries v.t.4h and v.t.4w furnished sequences with high npAA/C/S/T content. ^†^: Data for the previously reported v.t.2 selection is provided for comparison.^33^ (d) Initial characterization of the selected hits. Shown are the random insert sequences with the established modification pattern. Here and elsewhere in the text, residues highlighted in black represent unmodified C/S/T residues; in red—translationally installed npAAs (Figure 1c for chemical structures and one-letter codes); in blue—C/S/T-derived azol(in)es; in green—S/T-derived dhAAs (Dha and Dhb). The sequences selected for resynthesis are highlighted in light pink. ^[a]^: scale: ++/+/–, where “++” indicates expected peptide as the major translation product and “+” indicates expected peptide accompanied by major side products. ^[b]^: the efficiency of the enzymatic maturation; scale: ++/+/–, where “++” indicates the full conversion to thiopeptides with no/minimal accumulation of partially modified peptides and shunt products. ^[c]^: scale: ++/+/–, with “++” and “+” indicating the cases where a single species accounted for ≥85% and ≥70% of the observed thiopeptide products, respectively. ^[d]^: for w5 and w9, reliable structures could not be ascertained. ^[e]^: n.d.: not determined.

To ascertain the plausibility of our designs, we conducted affinity selections against TNIK (S. I. 2.3). The mRNA libraries v.t.4h or v.t.4w (initial 3.6 × 10^13^ molecules in each case) were *in vitro* translated using the reprogrammed genetic code (Figure 1). The resulting mRNA-barcoded precursor peptides were reverse transcribed to generate peptide-mRNA/cDNA conjugates, which were treated with Laz enzymes in the three-step protocol as described above. The resulting thiopeptide libraries were then subjected to a counterselection, which entailed an incubation with Dynabeads M-280, followed by a selection against TNIK immobilized onto the same beads. The recovered cDNA was amplified by PCR and transcribed for the following round. In total, we performed six rounds of selection, upon which both libraries indicated strong enrichment of TNIK-binding species as quantified by qPCR (Figure 3a). Next-generation sequencing of round six cDNA populations showed the convergence of both libraries to about ten sequence families each (Figure 3b). Curiously, even though v.t.4w is strictly a subset of the larger v.t.4h library, the resulting sequences were largely different. Only one major family, WgfXNxxCxx (insert sequence), was found in both data sets. Both selections resulted in sequences containing multiple npAAs and C/S/T residues, and as designed, the more compact genetic code of the v.t.4w library led to a higher density of npAA and C/S/T elements (Figure 3c).

We selected 15 sequences from each library (h1–15 from v.t.4h and w1–15 from v.t.4w) for the following characterization (Figure 3d). First, we investigated the fidelity of the reprogrammed translation. LC/MS analysis of the precursor peptides expressed using the constructed FIT system showed formation of the expected peptides as the major product in every case (Figures S14 and S15). Consistent with our initial studies, truncated peptides likely stemming from ribosomal drop-off^53^ were the main source of impurities, whereas the more problematic amino acid misincorporation was insignificant in all but four cases (h7, h9, h14, and w9). Notably, w13, which contained six npAAs including four consecutive *N*-methyl residues (the fggg motif), produced the expected peptide as the major product, which highlighted the robustness of the constructed translation system.

Next, we investigated the maturation of the selected precursor peptides by treating the translation products with LazDEF/LazBF/LazC in the three-step sequence during the selection. According to LC/MS, conversion of the linear precursors to the corresponding macrocyclic thiopeptides proceeded smoothly with little or no accumulation of partially modified peptides and shunt modification products in 29 out of the 30 cases (Figures S16 and S17). For the v.t.4h-derived hits, a single major thiopeptide product formed in seven out of the 15 sequences. The homogeneity of the maturation was higher for the v.t.4w-derived peptides (in 11 out of the 15 cases, a single major thiopeptide formed), despite the higher C/S/T content of the inserts (on average, 5.3 C/S/T residues in the random insert of w1–15 sequences compared to 3.1 for h1–15). This outcome was rather unexpected because with an increase in the number of C/S/T residues, the number of possible products grows exponentially. For instance, w7 contains seven C/S/T residues in the insert and can theoretically yield 9216 unique thiopeptides, yet Laz enzymes modified w7 to a single product containing one Thz and four dhAAs, leaving two residues unmodified. These results highlight the remarkable substrate-level cooperativity of Laz enzymes^54^—especially when it comes to modifying C/S/T-rich local environments—considering that the pattern of C/S/T residues in w7 and other discovered sequences bears no resemblance to that of wild-type LazA. Despite the extensive efforts to unravel the substrate recruitment and discrimination by Laz and related RiPP enzymes,^47,54−62^ our understanding of such a promiscuous (deep modification of random inserts) but at the same time selective (formation of one or at most a few products) processing of precursor peptides is lacking. Altogether, we narrowed our focus to 18 sequences which furnished mostly homogeneous thiopeptides (seven from the library v.t.4h and 11 from v.t.4w) for the following experiments. The remaining hit thiopeptides were not analyzed because identification of the active species in compound mixtures is challenging.

**Figure 4:**
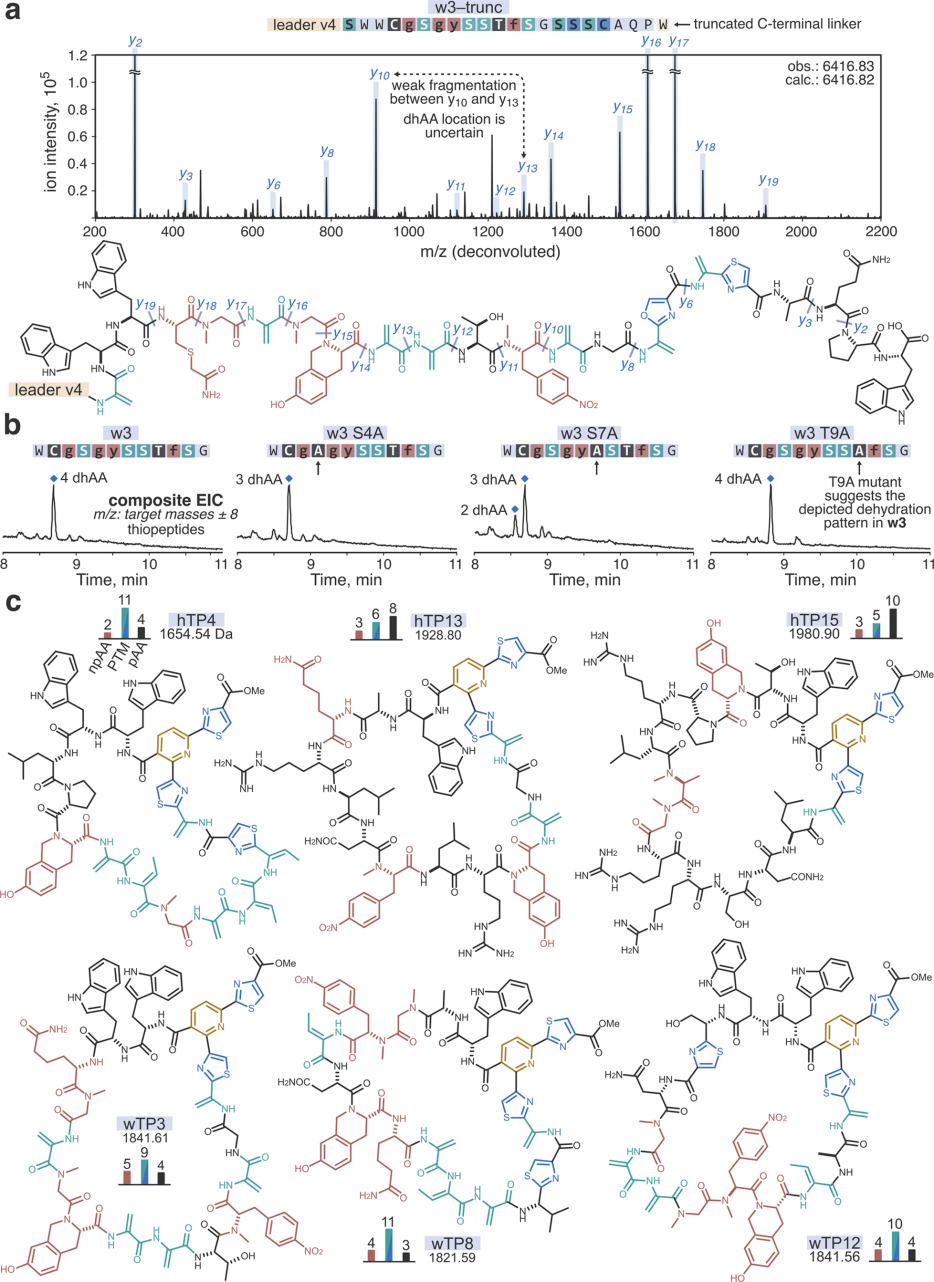
Elucidation of the structures of the discovered hits using w3 as an example. (a) MS/MS analysis of the insert modification pattern in C-terminally truncated *w3* precursor peptide (S. I. 2.6–2.7). Shown is a zoomed-in section of a charge-deconvoluted CID fragmentation spectrum for the LazDEF/LazBF-modified *w3–trunc*; *b*-ion assignments and neutral molecule losses are omitted for clarity. Fragmentation assignments are mapped onto the suggested chemical structure of the modified *w3–trunc* shown below. (b) Mutational analysis results. The specified *w3* mutants were expressed with the reprogrammed translation system and sequentially treated with LazDEF/LazBF/LazC. Reaction outcomes were analyzed by LC/MS. Displayed are composite EIC chromatograms for the detected thiopeptide products (scaled *Y*-axes). The combination of MS/MS and mutational analyses enabled an unambiguous structural assignment for wTP3. (c) Example chemical structures of the identified hit thiopeptides. The top and bottom rows feature the structures derived from library v.t.4h and v.t.4w, respectively. The bar graphs denote the numbers of npAA, PTM, and proteinogenic amino acids (pAAs) in the structures.

LC/MS analysis reveals the number of PTMs in a thiopeptide but not their location within the insert, particularly when not every C/S/T residue is modified. Additionally, LC/MS fails to disambiguate the nature of a PTM in some cases. For instance, the net mass change of −38.05 Da in a Ser-Cys-containing substrate can be attributed to the formation of either Dha-Thz or Oxz-thiazoline PTMs (Figure S1). To determine the structures of the discovered thiopeptides while being material-limited (our *in vitro* translation setup can routinely deliver up to ∼100 ng of material), we devised a strategy that combines tandem mass spectrometry (MS/MS) and mutagenesis (S. I. 2.7). For the MS/MS analysis, we prepared C-terminally truncated LazA variants and incubated the translation products with LazDEF/LazBF, omitting the LazC-catalyzed macrocyclization step. This treatment provided linear, but otherwise fully modified precursor peptides, which—in contrast to macrocyclic thiopeptides—are amenable to MS/MS (Figure 4a). However, MS/MS alone was insufficient in some cases, where incomplete *b*- and *y*-ion fragmentation ladders precluded unambiguous PTM assignments. To complement the mass spectrometry results, we prepared LazA variants containing Ser/Thr/Cys to Ala mutations as appropriate, studied their maturation with LazDEF/LazBF/LazC, and compared the number of PTMs in the resulting thiopeptides against the parent sequence (Figure 4b). The combination of MS/MS and mutagenesis enabled us to make structural assignments for 13 out of the 15 studied thiopeptides (Figures S18–S31), while eight additional structures were inferred by analogy.

Finally, we conducted an mRNA display assay, which mimicked a single round of selection, to probe the affinity of the discovered thiopeptides toward TNIK for 14 sequences (Figure 3d). This semiquantitative experiment indicated that all tested constructs bound to Dynabeads-immobilized TNIK, but not to beads only. Collectively, these results prompted us to select ten thiopeptides, five from each library, for resynthesis (hTP4, hTP6, hTP8, hTP13, and hTP15 derived from the eponymous precursors for library v.t.4h; and analogously, wTP3, wTP4, wTP8, wTP12, and wTP13 for v.t.4w). Thiopeptides from the WgfXNxxCxx family, prominently represented in both selections, could not be selected because of the challenges associated with the synthesis of thiazoline-containing peptides.

### Synthesis and Biochemical Characterization of Discovered Thiopeptides

To facilitate the synthesis, we simplified the structures of the selection hits in three ways. We truncated the C-terminal linker region, made an Oxz to Thz mutation in the scaffold moiety, and replaced all sulfide-containing amino acids with their CH_2_ isosteres (Figure S32). The resulting compounds, particularly those derived from the v.t.4w hits (examples in Figure 4c), featured complex, densely functionalized structures reminiscent of natural thiopeptides. For instance, thiopeptide wTP8 contained three Thz residues, five dhAAs, four npAAs, and only three proteinogenic amino acids. Nevertheless, we envisaged that our previously established strategy^63^ for solid phase-based peptide synthesis (**SPPS**) of lactazole-like thiopeptides could be leveraged to produce the requisite compounds. Briefly, the approach relies on the standard Fmoc/^t^Bu SPPS protocols for peptidyl chain assembly (S. I. 5.1). This process necessitated the synthesis of several custom Fmoc-protected amino acids, including the central pyridine-dithiazole-containing building block, thiazole-containing dipeptides, various selenocysteine derivatives, and two other npAAs (S. I. 4.1–4.6). Linear SPPS products are released from the solid support without removing the side-chain-protecting groups using a mildly acidic cleavage^64^ and are then macrocyclized in solution. In the final step, oxidative elimination of selenocysteine derivatives^65^ furnishes the requisite thiopeptides. In the event, the synthesis of all ten compounds progressed smoothly, yielding the target thiopeptides in mg quantifies, sufficient for the following assays (S. I. 5.2–5.3). The isolated compounds were characterized by UPLC (≥93% purity) and LC/MS. For six thiopeptides, the structural identity was further confirmed by ^1^H NMR spectroscopy (S. I. 7).

**Figure 5:**
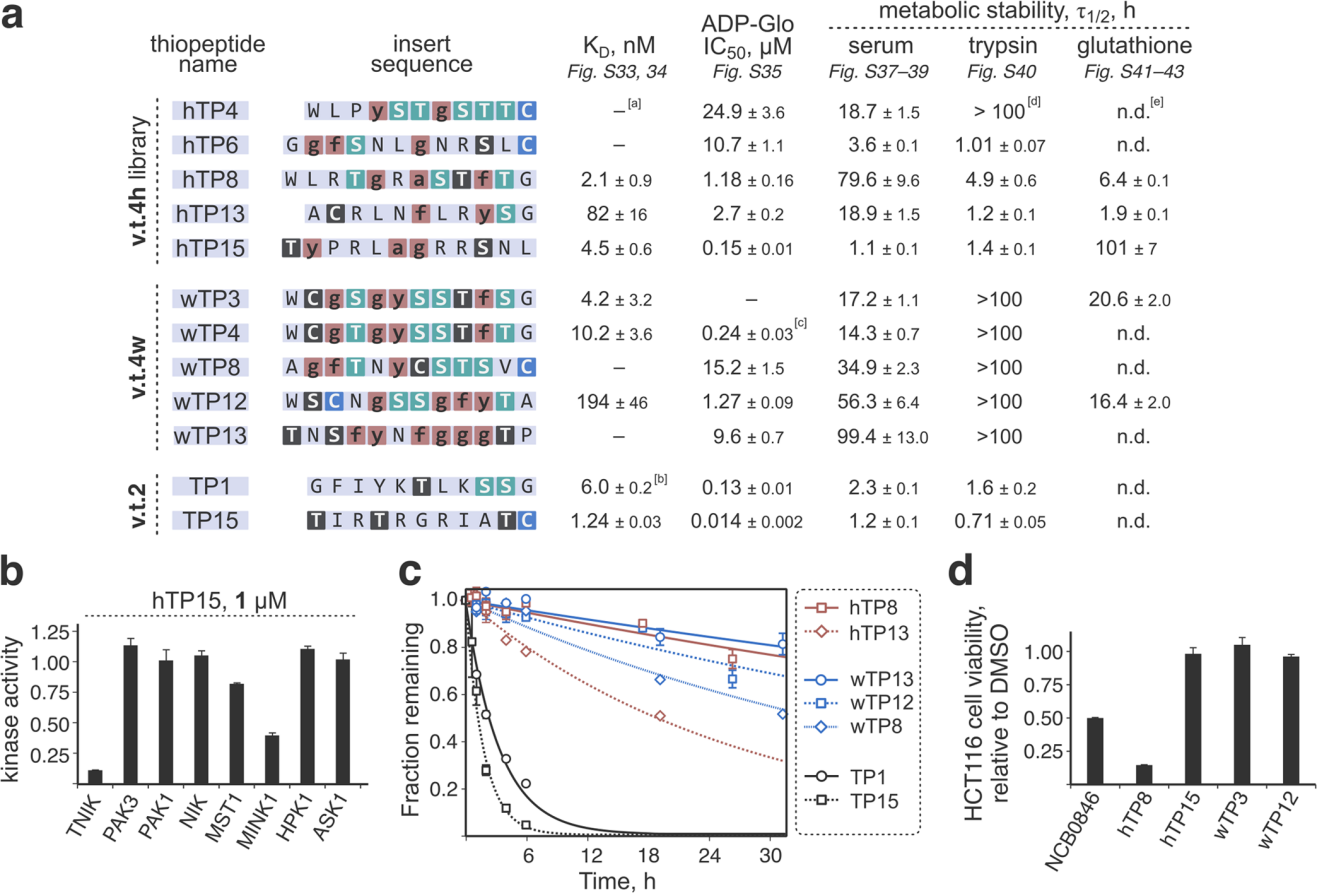
Biochemical characterization of the synthesized thiopeptides. (a) Values for binding affinity to TNIK (K_D_; average from three multicycle kinetic experiments; data fit assuming the 1:1 binding model; see also Table S5); in vitro kinase inhibition (IC_50_ against TNIK derived from at least two ADP-Glo-based experiments conducted in triplicate each; data fit to the standard 4-parameter logistic curve); and metabolic stability in the presence of human serum, trypsin, and glutathione (half-life, τ_1/2_; a single experiment conducted in triplicate; data fit to the standard first-order exponential decay curve). ^[a]^: no measurable binding/inhibition ^[b]^: binding and inhibition data for TP1 and TP15 are taken from ref (33). ^[c]^: the compound did not completely inhibit the kinase even at high concentrations (see Figure S35) ^[d]^: no measurable degradation during the course of the experiment ^[e]^: not determined. (b) Kinase selectivity profiling outcomes for hTP15, tested at 1 μM thiopeptide concentration. Analogous data for hTP8 is summarized in Figure S36. (c) Degradation of the five best compounds (hTP8, hTP13, wTP8, wTP12, and wTP13) in human serum compared to the previously discovered^33^ TP1 and TP15. (d) Cellular activity of the discovered thiopeptides in HCT116 cells. Shown are the cell viability results after a 24 h incubation with the compounds (S.I. 2.11). Thiopeptides hTP8 and wTP12 as well as the positive control (NCB0846)^38^ were assayed at 10 μM concentration; hTP15 and wTP3, at 8 μM.

A surface plasmon resonance experiment established that six discovered thiopeptides bound to TNIK with high affinity, with three compounds (hTP8, hTP15, and wTP3) demonstrating single-digit nM dissociation constants (best K_D_ = 2.1 nM for hTP8; Figures 5a, S33, and S34). Four thiopeptides inhibited TNIK in an ADP-Glo kinase activity assay with single-digit μM IC_50_ values, and two compounds (hTP15 and wTP4) stood out as sub-μM inhibitors (Figure S35). A kinase selectivity profiling experiment conducted for hTP8 and hTP15 against a panel of eight enzymes related to TNIK (all belonging to the Ste-20 kinase family;^66^Figure S36a), indicated that the compounds are selective inhibitors of the target protein. In addition to TNIK, hTP15 (1 μM) partially inhibited (60% inhibition) the highly related MINK (94% sequence similarity) but not any of the remaining kinases (Figure 5b). At 10 μM concentration, hTP8 suppressed the activity of TNIK almost completely (94%), whereas other kinases remained partially active (<65% inhibition for all tested enzymes; Figure S36b). Taken together, these results validate the established discovery pipeline. Although the v.t.4h and v.t.4w libraries are 50- and 5000-fold less diverse than our previously employed library v.t.2, the selections still led to multiple potent and selective ligands for the target protein.

To investigate the metabolic stability of the discovered compounds, we incubated the thiopeptides in human serum at 37 °C in the presence of an internal standard and quantified the amount of the remaining analyte at various time points by LC/MS (S. I. 2.10). We found that eight out of ten compounds had excellent stability in serum, with half-lives ranging from 14 to 99 h (Figures 5a,c and S37). All library v.t.4w-derived thiopeptides demonstrated high resistance to proteolysis, whereas the two unstable constructs, hTP6 and hTP15, both were Arg-containing peptides identified from the v.t.4h selection. Accordingly, LC/MS analysis detected the accumulation of partially proteolyzed hTP6 and hTP15, with the primary cleavage sites located around the Arg residues (Figures S38 and S39). The tryptic activity of human serum is well-documented,^67^ which may explain the compromised stabilities of hTP6 and hTP15. To directly probe the susceptibility of the thiopeptides to trypsin, we incubated the compounds with agarose-immobilized trypsin and quantified the outcomes by LC/MS against an internal standard (Figures 5a and S40). The results reiterated the serum stability assay; the Arg-containing peptides were rapidly digested, whereas all v.t.4w-derived structures, which did not contain basic residues by design, were completely stable. Despite the absence of Arg and Lys in the structures of hTP4, hTP8, wTP3, wTP4, wTP8, and wTP12, their high stability in serum was unexpected because these peptides had multiple dhAAs (as many as six for hTP4) which can potentially react with numerous serum-derived thiols.^68^ Accordingly, the slow disappearance of these dhAA-rich thiopeptides from human serum was not accompanied by the formation of discernible proteolytic fragments. To directly assay the stability of the discovered compounds to metabolic thiols, five compounds (hTP8, hTP13, hTP15, wTP3, and wTP12) were incubated with 5 mM glutathione at pH 7.5 and 37 °C. At various time points, the reactions were terminated by the addition of iodoacetamide, and the amounts of intact thiopeptides were quantified by LC/MS (Figures 5a and S41–S43). The reactivities with respect to glutathione were variable and did not correlate with the number of dhAAs in the structure. For example, hTP13, which contained only one Dha and one Dha-Thz, rapidly reacted with glutathione with a half-life of less than 2 h, whereas wTP3 (four Dha and one Dha-Thz) was considerably more stable (τ_1/2_ = 20.6 h). These results helped in rationalizing the high metabolic stability of some (wTP3 and wTP12) but not all studied thiopeptides (hTP8 and hTP13). Why hTP8 and hTP13 are stable in serum despite their moderate reactivity to thiols remains unexplained. Perhaps, their high lipophilicity promotes serum protein binding which can increase their chemical stability.^69^

Lastly, we investigated four discovered thiopeptides (hTP8, hTP15, wTP3, and wTP12) in a cell viability assay conducted against HCT116 colon carcinoma cells. Multiple previous studies utilizing small molecules targeting TNIK demonstrated that its inhibition is lethal in the cell lines with a dysregulated Wnt/β-catenin signaling pathway^38,70−73^ (HCT116 contains a mutation in the CTNNB1 gene).^74^ We found that after a 24 h incubation, hTP8 was more cytotoxic than a small molecule control, NCB0846 (both 10 μM),^38^ whereas the remaining tested compounds were inactive (Figure 5d). A dose-dependent 48 h long incubation with hTP8 established an IC_50_ value of 7.2 μM, in line with several small molecule TNIK inhibitors (Figure S44a).^70,71^ Further RT-qPCR experiments aiming to confirm the on-target action of hTP8 revealed that in contrast to NCB0846,^38^ and similarly to the more selective TNIK inhibitors,^70,71^ hTP8 did not downregulate MYC and TNIK mRNA levels in HCT116 cells (Figure S44b,c). At present, the inconsistencies in the literature regarding the molecular phenotypes associated with TNIK inhibition preclude us from drawing definitive conclusions regarding the effects of hTP8. A dedicated study will be required to disambiguate its mode of action.

### Structural Analysis of the TNIK/wTP3 Interaction

**Figure 6:**
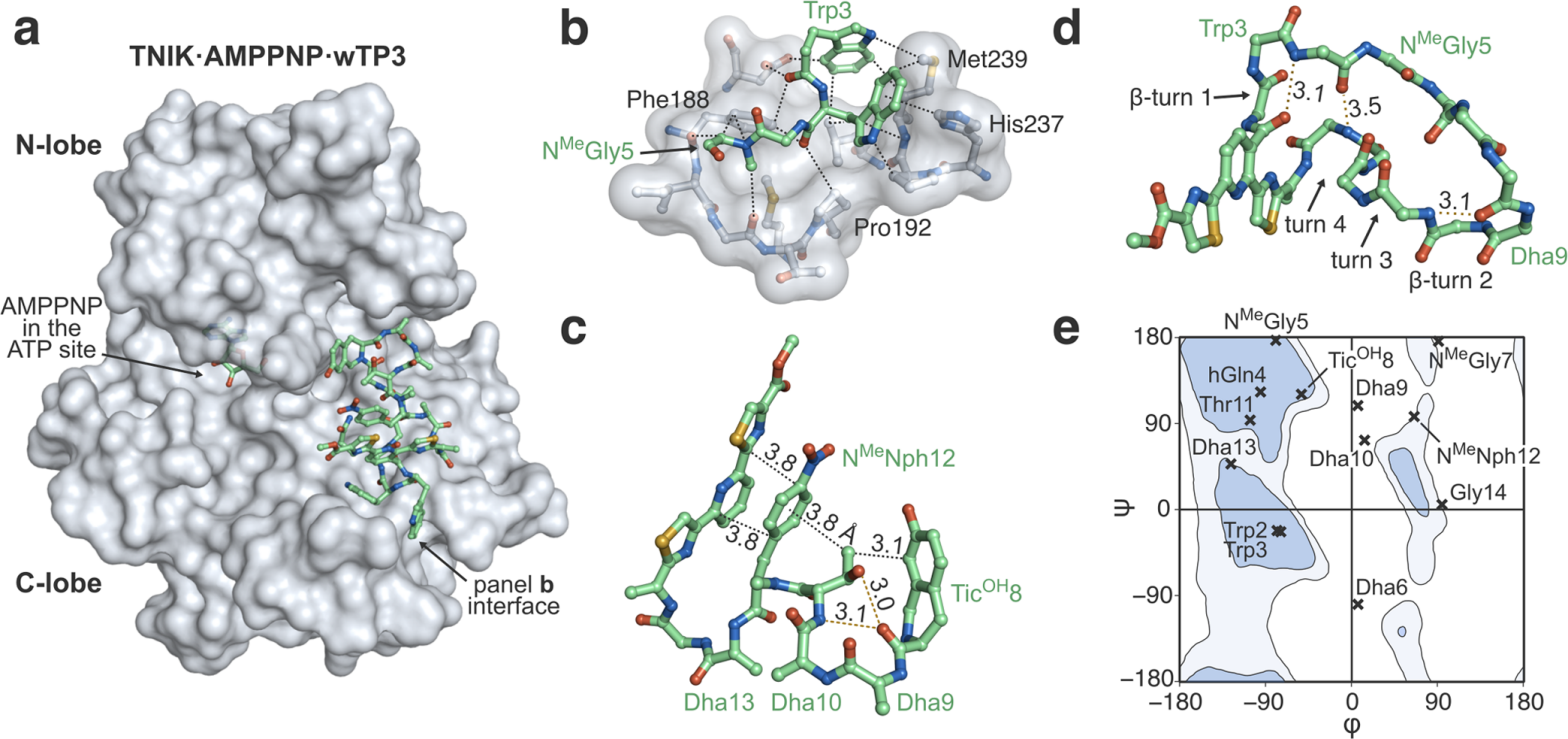
Structural analysis of the interaction between TNIK and wTP3. (a) Overview of the X-ray crystal structure of the TNIK·AMPPNP·wTP3 complex (pdb 8wm0). The protein surface is shown in gray; wTP3 and AMPPNP are displayed as ball and stick models. (b) Hydrophobic interactions between αG-helix and P+1 loop of TNIK and wTP3. Atoms within 4 Å distance are connected with dotted lines. (c) Intramolecular interactions in wTP3 with key distances highlighted. Hydrogen bonds are shown as yellow dotted lines. (d) Folding of TNIK-bound wTP3. The protein and amino acid side chains are omitted for clarity. The thiopeptide folds into a noncanonical structure featuring four turns. (e) Plot of φ/ψ dihedral angles of TNIK-bound wTP3. Inner and outer contours enclose 98 and 99.5% of the structures in Protein Data Bank, respectively. wTP3 contains several amino acids outside of the canonical Ramachandran space.

To understand how densely functionalized thiopeptides interact with their target proteins, we solved the X-ray crystal structure of the TNIK·AMPPNP·wTP3 complex at 2.8 Å resolution (Figure S45, Table S7). Like the previously identified TP1 and TP15 thiopeptides,^33^ wTP3 binds to the substrate binding site of TNIK. However, the binding mode of wTP3 deviates from those of TP1 and TP15 (Figure S46).^33^ The peptide contacts both the N- and C-lobes of TNIK, interacting primarily with the αG-helix and P+1 loop in the C-terminal lobe (Figure 6a). The N-lobe amino acids, Asp61-Glu62 in the αC-helix and Thr35-Tyr36 in the Gly-rich loop, additionally contact Tic^OH^8-Dha9 residues in wTP3 (Figure S47). The hydrogen bond between the side chain carboxylate of Glu62 and the amide nitrogen of Dha9 is the only polar contact in the interface, and the near absence of polar interactions between the protein and wTP3 is a striking feature of the structure. Instead, the high steric complementarity of wTP3 and TNIK appears to drive the association. Trp2, Trp3, and N-Me-Gly5 of wTP3 make numerous contacts with the exposed hydrophobic patch on the surface of TNIK (Figure 6b). The carbonyl oxygen of Trp3 and the N-Me group of N-Me-Gly5 occupy the P+1 site, whereas the indole groups of Trp2 and Trp3 are stacked against the αG-helix residues, primarily His237, Pro238, Met239, and Leu242. The predominantly hydrophobic nature of the interaction between TNIK and wTP3 is reminiscent of how natural thiopeptides engage their targets.^4,6^

Another notable feature of the structure is the intramolecular interactions and folding of wTP3. The pyridine-diazole moiety, the side chains of N-Me-Nph12, and Thr11 as well the phenol in Tic^OH^8 are stacked on top of each other providing π–π and CH-π interactions which appear to rigidify and stabilize the peptide structure (Figure 6c). wTP3 folds into a unique “quadruply twisted” conformation featuring two β-turns and two noncanonical turn elements organized without the use of intramolecular hydrogen bonds owing to the multiple nonproteinogenic elements in the structure (Figure 6d). A dihedral angle plot reveals that Dha6, Dha9, and Dha10 adopt conformations with φ angles close to 0, i.e., entirely outside of the canonical α-amino acid space (Figure 6e). This analysis highlights how the combinatorial use of α-, *N*-methyl-, and dehydroamino acid building blocks enables v.t.4h- and v.t.4w-derived thiopeptides to access large regions of the Ramachandran space and therefore sample noncanonical conformations.

## Conclusions

The identification of potent ligands and inhibitors of the target kinase from v.t.4h and v.t.4w library selections affirms the use of compact reprogrammed genetic codes for the discovery of densely functionalized thiopeptides. The library designs developed here necessitated several advances to the original platform.^33^ These included the construction of a reprogrammed *in vitro* translation system, the development of a three-step enzymatic maturation protocol, and the establishment of a strategy to elucidate the structures of the identified thiopeptides. As a result, v.t.4h and v.t.4w thiopeptides contain both translationally installed npAAs and enzymatically installed PTMs. The use of compact reprogrammed genetic codes decreased the compound diversity of the v.t.4h and v.t.4w libraries, but the selection against TNIK still led to the identification of potent protein ligands with affinities as high as *K*_D_ = 2.1 nM (hTP8). The discovered structures approach those of natural thiopeptides and many nonribosomal peptides in terms of the density of privileged nonproteinogenic elements. The 18-residue macrocyclic wTP8 contains as few as three proteinogenic amino acids, and all synthesized v.t.4w hit thiopeptides are composed of at least 60% noncanonical building blocks. Accordingly, the thiopeptides displayed a high resistance to proteolysis. The v.t.4w library design, which maximizes the density of nonproteinogenic amino acids and excludes basic residues from the random insert, appears to be especially promising in this regard. The structural analysis of the interaction between TNIK and wTP3 underscored not only how the noncanonical building blocks facilitate the target engagement but also their roles in thiopeptide folding and structure rigidification. Overall, the use of compact reprogrammed genetic codes in the thiopeptide-mRNA display takes another step toward the discovery of macrocyclic peptides with high metabolic stability and excellent pharmacological properties for early-stage drug discovery.

## Data Availability

Experimental procedures and results are summarized in the Supporting Information and Supplementary Tables. The TNIK·AMPPNP·wTP3 X-ray crystal structure was uploaded to Protein Data Bank (pdb entry 8wm0). Other data are available from the corresponding authors upon reasonable request.

## Supporting Information Available

Experimental procedures, additional figures and tables, and the characterization of the synthesized compounds (PDF)
DNA template assembly schemes (Table S1); a list of oligonucleotides (Table S2); tRNA aminoacylation conditions (Table S3); top 100 selection hits (Table S4); SPR kinetic data (Table S5); TNIK sequence information (Table S6); crystal analysis summary (Table S7) (XLSX)
