# Supplementary material for: A Compact Reprogrammed Genetic Code for De Novo Discovery of Proteolytically Stable Thiopeptides
Source: J Am Chem Soc. 2024 Mar 16;146(12):8058–70. doi: 10.1021/jacs.3c12037 (PMC10979747; doi:10.1021/jacs.3c12037)

Supporting information for

A compact reprogrammed genetic code for de novo discovery of proteolytically stable thiopeptides

Alexander A. Vinogradov[1,†,*], Yue Zhang[1,†], Keisuke Hamada[2], Shunsuke Kobayashi[2], Kazuhiro Ogata[2], Toru Sengoku[2], Yuki Goto[1,3*], Hiroaki Suga[1,*]

[1] Department of Chemistry, Graduate School of Science, The University of Tokyo, Bunkyo-ku, Tokyo 113-0033, Japan

[2] Department of Biochemistry, Graduate School of Medicine, Yokohama City University, Kanazawa-ku, Yokohama 236-0004, Japan

[3] Present address: Department of Chemistry, Graduate School of Science, Kyoto University, Sakyo, Kyoto 606-8502, Japan

* Correspondence to a_vin@chem.s.u-tokyo.ac.jp, y-goto@chem.s.u-tokyo.ac.jp, and hsuga@chem.s.u-tokyo.ac.jp

† Contributed equally

The authors declare the following competing interests: A.V., Y.Z., Y.G. and H.S. are listed as co-inventors on the patents pertaining to lactazole engineering. Other authors declare no competing interests.

Contents

1. General 3
2. Methods 6
2.1. mRNA library construction 6
2.2. Preparation of tRNA, flexizymes and aminoacylation 7
2.3. TNIK affinity selection 8
2.4. NGS and data analysis 9
2.5. Preparation of synthetic DNA for in vitro translation 10
2.6. Enzymatic reactions and LC/MS analysis of their outcomes 11
2.7. Determination of thiopeptide structures 13
2.8. Surface plasmon resonance 14
2.9. Kinase inhibition assays with ADP-Glo 15
2.10. Thiopeptide metabolic stability experiments 15
2.11. Cell assays 17
2.12. X-ray crystallography 18
3. Supplementary Figures 20
4. Synthesis of amino acids 79
4.1. Synthesis of Fmoc–N^{Me}–Phe(*p*NO_2)–OH 79
4.2. Synthesis of Fmoc–Tic(*p*O^tBu)–OH 80
4.3. Synthesis of Fmoc–Leu–Thz–OH 82
4.4. Synthesis of Fmoc–Val–Thz–OH 85
4.5. Synthesis of Fmoc–Ser(O^tBu)–Thz–OH 87
4.6. Synthesis of Fmoc–$^{(R,S)}$Abu($^{\beta}$SePh)–Thz–OH 89
5. Synthesis of thiopeptides 91
5.1. General procedures 91
5.2. Total Synthesis of wTP3 93
5.3. Total Synthesis of Other Discovered Thiopeptides 98
6. Supplementary References 117
7. NMR spectra 120

1. General

All commercially available materials were used without further purification. Amino acids, coupling reagents, and resins were obtained from Sigma-Aldrich, TCI chemicals or Nacalai Tesque. All solvents were of reagent grade or HPLC grade (FUJIFILM Wako Pure Chemical Corporation). All anhydrous (H_2O ≤ 10 ppm) solvents were also obtained from FUJIFILM Wako Pure Chemical Corporation. Biotinylated TNIK (kinase domain, amino acids 1-314; NP_055843.1) was purchased from Carna Biosciences (Kobe, Japan). Magnetic beads with immobilized streptavidin were from Invitrogen (Dynabeads Streptavidin M280, #11206D). Chemical synthesis of thiopeptides TP1 and TP15 used for comparison in the metabolic stability studies was previously described.[1] Small molecule TNIK inhibitor control NCB0846 was from AdooQ BioScience (#A20535).

Oligonucleotides for library assembly (HPLC and OPC purification grade for regular and randomized sequences, respectively) were purchased from GeneDesign Inc. (Osaka, Japan). Other primers were from Eurofins Genomics (OPC purification grade). All oligonucleotides were used as received. PCR amplifications were conducted in a BioER TC-96GHBC thermal cycler. qPCR analysis was performed using a LightCycler Nano instrument (Roche) running on LightCycler Nano Software v.1.0.

Protein purification and synthesis of *Streptomyces lactacystinaeus* $tRNA^{Glu}$ were done as previously described.[2–4] Protein concentration measurements were carried out on a Nanodrop 2000c spectrophotometer (Thermo Scientific) equipped with a 5 mm pathlength quartz cuvette using extinction coefficient values calculated with ExPASy ProtParam tool (https://web.expasy.org/protparam). For LazF, a micro BCA assay (Thermo Fisher) was used instead. For thiopeptides, concentrations of DMSO stock solutions were determined spectrophotometrically using the extinction coefficients at 329 nm calculated from the contributions of the central heterocyclic core (ε^{329} = 10100 $M^{-1}cm^{-1}$; previously reported; DMSO)[1] and N^{Me}-nitrophenylalanine moieties (ε^{329} = 870 $M^{-1}cm^{-1}$; measured herein; DMSO).

Biochemical measurements (sections 2.8 – 2.11) were performed in triplicate or more. Most other experiments were reproduced to an analogous outcome at least once. In the cases where the determination of biophysical parameters involved non-linear regression (K_D, IC_{50}, $\tau_{1/2}$), reported are standard errors of the fit unless otherwise noted.

For organic synthesis, normal phase column chromatography was performed on Biotage Isolera™ Spektra flash purification system equipped with Biotage® SNAP Ultra and Sfär Silica HC D High-Capacity columns. ^{1}H and ^{13}C NMR spectra were recorded on a JEOL ECS400 MHz spectrometer at room temperature (~25°C). Chemical shifts for ^{1}H NMR are reported in parts per million (δ) relative to tetramethylsilane in deuterated solvent as the

internal standard. Chemical shifts for ^{13}C NMR are reported in parts per million (δ) relative to the solvent (e.g., $CDCl_3$, δ = 77.16). The following abbreviations are used for spin multiplicity: s = singlet, d = doublet, t = triplet, q = quadruplet, m = multiplet. High-resolution mass spectra were acquired on a Bruker MicrOTOF II TOF-MS mass spectrometer.

Reverse phase (RP) preparative HPLC separations utilized a mobile phase consisting of 0.1% trifluoroacetic acid (TFA) (v/v) in water (Solvent A) and 0.1% TFA (v/v) in acetonitrile (Solvent B) and were performed on a Prominence HPLC (Shimadzu) equipped with a Chromolith Prep RP-18 (100–25 mm) column (Merck) at a flow rate of 25 mL/min or using a Zorbax 300SB-C18 PrepHT (21.2 × 250 mm, 7 µm, 300 Å) column (Agilent) at 20 mL/min. Purification was performed following the method listed below.

RP-HPLC purification method

0 – 5min	20% B (10mL/min)
5 – 10 min	20% B (10 – 25mL/min)
10 – 90 min	20 – 55% B
90 – 100 min	55 – 95% B
100 – 110 min	95% B

For LC/MS monitoring of thiopeptide synthesis, separations utilized a mobile phase consisting of 0.1% formic acid (v/v) in water (Solvent A′) and 0.1% formic acid (v/v) in acetonitrile (Solvent B′). Analysis was performed on a Waters® SELECT SERIES H class/Cyclic IMS instrument equipped with ACQUITY UPLC® Peptide BEH C18 column (2.1 × 150 mm, 1.7 µm, 300 Å) at a flow rate of 0.3 mL/min and 60°C with one of the two methods listed below.

LC/MS method 1

0 – 2 min	10% B′
2 – 12 min	50 – 70% B′, linear ramp
12 – 13 min	95% B′
13 – 14 min	5% B′

LC/MS method 2

0 – 2 min	10% B′
2 – 12 min	25 – 65% B′, linear ramp
12 – 13 min	95% B′
13 – 14 min	5% B′

For analytical UPLC analysis, separations utilized a mobile phase consisting of Solvents A and B. Analysis was performed on a Shimadzu Nexera X2 UHPLC System equipped with ACQUITY UPLC® BEH C18 column (2.1 × 150 mm, 1.7 µm, 300 Å) at a flow rate of 0.5 mL/min and 60°C using the following method.

UPLC analysis method

0 – 2 min	10% B
2 –18 min	10 – 80% B
18 – 20 min	80 – 95% B
20 – 22 min	95% B
22 – 25 min	5% B

The purity of synthesized thiopeptides was quantified by integrating the areas under the peaks after baseline correction.

2. Methods

2.1. mRNA library construction

PCR assembly. v.t.4h and v.t.4w DNA libraries were de novo assembled by PCR from oligonucleotide primers. The final v.t.4h library contained a mixture of 9-, 10-, 11- and 12-mer random inserts, whereas v.t.4w consisted only of 12-mers. These 5 libraries were assembled individually, and the final v.t.4h library was constructed by mixing individual mRNAs as elaborated in the puromycin ligation subsection below.

PCR assembly schemes and primer sequences are summarized in Tables S1 and S2, respectively. First, overlapping forward and reverse primers were annealed and extended in the primer extension step. To this end, 150 µL of Platinum SuperFi DNA Polymerase solution [PCR buffer (1x), SuperFi Pol (1x), 250 µM each dNTP; **SF PCR mix**; buffer and enzyme from Thermo Fisher] containing 270 nM forward and 250 nM reverse primers was denatured at 95 °C for 60 s. Then, three cycles of annealing (50 °C for 30 s) and extension (72 °C for 30 s) were carried out. In the next step, the extension product (1 volume, 25–100 µL depending on the library diversity) was added to SF PCR mix (20 volumes) containing 500 nM forward and reverse primers, and five cycles of amplification ensued (three stage thermal cycling including a denaturing step at 95°C for 20 s, annealing at 57.2°C for 20 s, and extension at 72°C for 30 s). After, the mixture was further incubated at 72°C for 60 s, and cooled to 4°C. The outcomes were evaluated by TapeStation (Agilent Technologies). DNA was first extracted by phenol/chloroform/isoamyl alcohol (25:24:1, saturated with 10 mM Tris (pH 8.0), 1 mM EDTA), and then by chloroform/isoamyl alcohol (24:1).

Transcription. Extracted DNA was precipitated with ethanol, washed with 70% ethanol in water (v/v), dissolved in water, and added to the transcription reaction mix [40 mM Tris buffer (pH 8.0) supplemented with 20 mM $MgCl_2$, 10 mM DTT, 1 mM spermidine, 0.01% Triton X-100, 240 nM T7 RNA polymerase, 0.04 U/µL RNasin RNase inhibitor (Promega), and 3.75 mM each NTP; 0.5–2.0 mL total volume depending on the library diversity]. Transcription was allowed to proceed at 37 °C for 14 h, after which RQ1 RNase-free DNase (final concentration [**f.c.**]: 0.025 units/µL; Promega) was added, and the reactions were further incubated for 60 min at 37°C. Reactions were quenched by the addition of EDTA (f.c.: 67 mM) and NaCl (f.c.: 270 mM). The transcripts were precipitated with isopropanol, washed with 70% ethanol (v/v), redissolved in water, and purified by 6% polyacrylamide gel electrophoresis (PAGE) containing 6 M urea. RNA extracted from the gel with 300 mM NaCl were collected by ethanol precipitation, dissolved in water, and frozen at –80 °C for storage.

Starting with round 2 of selection, transcription was scaled down to a 25 µL reaction, and PAGE purification was not performed.

Puromycin ligation. Individual v.t.4h mRNA libraries (containing 9–12-mer random inserts) were mixed in a 5:10:15:30 molar ratio. The resulting mRNA libraries (v.t.4h and v.t.4w which consisted exclusively of the 12-mer insert) were attached to puromycin via a Y-ligation performed with the use of T4 ligase. The reaction containing 1 µM mRNA, 1.5 µM puromycin linker and 1 µM T4 ligase in ligation buffer (40 mM Tris, pH 7.8, 10 mM $MgCl_2$, 10 mM DTT, 0.5 mM ATP in a water/DMSO mixture [8:2, v/v]) was incubated at 25 °C for 45 min. Ligated mRNA was extracted with phenol/chloroform as described above, precipitated with ethanol, washed with 70% ethanol, and redissolved in water. The outcome of puromycin ligation was judged by PAGE (6%, 6 M urea). Ligation product was diluted with water to 6 µM and frozen at –20 °C for storage. This product was used for in vitro translation during selection (section 2.3).

2.2. Preparation of tRNA, flexizymes and aminoacylation

RNA preparation. tRNA ($tRNA^{Pro1E2}_{GUA}$, $tRNA^{Pro1E2}_{GAA}$, $tRNA^{Pro1E2}_{GAU}$, and $tRNA^{Pro1E2}_{GUG}$) and eFx were prepared by in vitro transcription with T7 RNA polymerase from DNA templates assembled by PCR. Forward and reverse primers were extended, and then further amplified by PCR under the standard conditions as described in section 2.5. Primer sequences and assembly schemes can be found in Tables S1 and S2. PCR products were extracted by phenol/chloroform/isoamyl, chloroform/isoamyl alcohol, and precipitated with ethanol (as in section 2.1). DNA were redissolved in water and added to the transcription reaction mix (composition as in section 2.1). For transcription of tRNA, 5 mM GMP was additionally supplied to the reaction mixture. Reactions were conducted at 37°C for 12–16 h on a 2 (tRNA) or 6 mL (eFx) scale. After, RQ1 RNase-free DNase (f.c.: 0.025 units/µL) was added, and the reactions were further incubated for 60 min at 37°C. The transcripts were precipitated with isopropanol, redissolved in water, and purified by 8% (tRNA) or 12% (eFx) polyacrylamide gel containing 6 M urea. RNA extracted from the gel with 300 mM NaCl were collected by ethanol precipitation followed by centrifugation (15300 g for 15 min, 25°C), and dissolved in water for storage (–20 °C).

Aminoacylation. tRNA were aminoacylated with eFx using appropriate activated amino acids as substrates as summarized in Table S3. The synthesis, aminoacylation, and ribosomal incorporation of N^{Me}-Gly, N^{Me}-Ala, N^{Me}-Nph and Tic^{OH} were previously reported.[5,6] To perform aminoacylation, tRNA (25 µM) and eFx (25 µM) were incubated with the appropriate amino acid substrate (5–20 mM) in HEPES-KOH buffer (50 mM; pH 7.5) containing 600 mM $MgCl_2$ on ice for 4 h. The reactions were stopped with the addition of 300 mM NaOAc (pH 5.2), and precipitated with ethanol. Precipitated RNA was recovered by centrifugation (15300 g for 15 min). The pellet was washed with 70% ethanol in water (v/v) containing 100 mM NaOAc (pH 5.2) and used in translation.

2.3. TNIK affinity selection

Translation and reverse transcription. An *in vitro* translation system was reconstituted by mixing purified ribosome, enzymes, and translation factors.[7,8] The final reaction mixture contained 50 mM HEPES-KOH (pH 7.6), 100 mM KOAc, 2 mM GTP, 2 mM ATP, 1 mM CTP, 1 mM UTP, 20 mM creatine phosphate, 12 mM $Mg(OAc)_2$, 2 mM spermidine, 2 mM DTT, 1.5 mg/mL *E. coli* total tRNA (Roche), 1.2 µM ribosome, 0.6 µM MTF, 2.7 µM prokaryotic IF1, 0.4 µM IF2, 1.5 µM IF3, 10 µM EF-Tu, 10 µM EF-Ts, 0.26 µM EF-G, 0.25 µM RF2, 0.17 µM RF3, 0.5 µM RRF, 4 µ/mL creatine kinase, 3 µg/mL myokinase, 0.1 µM pyrophosphatase, 0.1 µM nucleotide-diphosphatase kinase, 0.1 µM T7 RNA polymerase, 0.73 µM AlaRS, 0.03 µM ArgRS, 0.38 µM AsnRS, 0.13 µM AspRS, 0.02 µM CysRS, 0.06 µM GlnRS, 0.23 µM GluRS, 0.09 µM GlyRS, 0.02 µM HisRS, 0.4 µM IleRS, 0.04 µM LeuRS, 0.11 µM LysRS, 0.03 µM MetRS, 0.68 µM PheRS, 0.16 µM ProRS, 0.04 µM SerRS, 0.09 µM ThrRS, 0.03 µM TrpRS, 0.02 µM TyrRS, 0.02 µM ValRS; 500 µM each proteinogenic amino acid (except for Phe, Tyr, Lys, His and Ile which were omitted from the mixture), 100 µM 10-formyltetrahydrofolate (10-HCO-THF); 25 µM each aminoacylated tRNA (N^{Me}-Nph-$tRNA^{Pro1E2}_{GUA}$, Tic^{OH}-$tRNA^{Pro1E2}_{GAA}$, N^{Me}-Gly-$tRNA^{Pro1E2}_{GAU}$, and N^{Me}-Ala-$tRNA^{Pro1E2}_{GUG}$); and 1.2 µM puromycin-conjugated library v.t.4h or v.t.4w mRNA.

In round 1, translation was carried out on a 50 µL scale (60 pmol of mRNA; 3.6×10^{13} molecules). In round 2 and henceforth, translation was scaled down to a 5 µL scale.

The resulting solutions were first incubated at 37°C for 40 min, and then at 25°C for 10 min before adding EDTA (pH 8.0) to a final concentration of 17 mM. Quenched translation reactions were further incubated at 37°C for 10 min to ensure complete dissociation of mRNA from the ribosome. Reverse transcription was performed with MMLV (H–) reverse transcriptase (Promega) using v.t.4.RT.R33 primer (sequence in Table S2) at 42 °C for 60 min following manufacturer's protocol (Tris 50 mM, pH 8.3, 2 mM $MgCl_2$).

Maturation with Laz enzymes. To one volume of ice-cold reverse transcription product, 1 volume of LazDEF enzyme premix, which contained 3 µM LazD, 4 µM LazE and 4 µM LazF in 100 mM Tris buffer (pH 8.0) supplemented with 20 mM $MgCl_2$, 9 mM ATP, and 2 mM DTT, was added on ice. The mixture was transferred to a 25°C thermostat and incubated for 3 h. The tubes were then placed on ice, and a solution of iodoacetamide in water was added (f.c.: 5 mM). After 5 min on ice, 1 volume of LazBF enzyme premix [4 µM LazB, 2.2 µM LazF, 2 µM *S. lividans* GluRS, and 40 µM *S. lactacystinaeus* $tRNA^{Glu}$ in 50 mM Tris buffer (pH 8.0) supplemented with 10 mM $MgCl_2$, 4.5 mM ATP, and 0.5 mM glutamic acid] was added. After a 4 h incubation at 25°C, LazC (f.c.: 4 µM) was added directly to the mixture, and the reaction was allowed to proceed for an additional 3 h at 25°C.

TNIK affinity pulldown. The tubes were transferred back on ice, and 0.1 volume of 10x blocking buffer [TBS-T supplemented with 20 mg/mL bovine serum albumin and 20 mg/mL yeast total RNA, where TBS-T contained 50 mM Tris, 150 mM NaCl, 0.2% (v/v) tween-20, pH 7.6] was added. The resulting samples were subjected to a counterselection (not performed during the first round). A 1:1 mixture of M280 SAv Dynabeads and the same beads bound to D-biotin (prepared by incubating the beads with a saturating concentration of D-biotin at 4 °C for 10 min) was washed twice with TBS, once with 1x blocking buffer and added to the libraries (0.8 µL of 10 mg/mL bead slurry per pmol of library mRNA). Incubation at 4 °C for 30 min ensued, after which the supernatant was recovered and added to TNIK-immobilized M280 SAv Dynabeads (0.5 µL of 10 mg/mL bead slurry per pmol of library mRNA; prepared by agitating the beads with 1 µM TNIK in TBS-T at 4 °C for 10 min and washing thrice with TBS-T). The mixtures were nutated at 4 °C for 30 min, and after, the beads were washed thrice with TBS-T. Elution of captured cDNA was conducted by heating the beads suspended in elution buffer [SuperFi PCR buffer (1x), 250 µM each dNTP, 250 nM T7g10M.F46 and v.t.4.RT.R33 primers] at 95 °C for 5 min.

PCR amplification. Concentrations of recovered cDNA, as well as the cDNA recovery rate as reported in Fig. 3a were determined by qPCR. Recovered sample aliquots were amplified [10 mM Tris (pH 8.4), 50 mM KCl, 0.1% (v/v) Triton X-100, 2.5 mM $MgCl_2$, 250 µM each dNTP supplemented with 250 nM T7g10M.F46/ v.t.4.RT.R33 primers, Taq DNA polymerase (1x) and Sybr Green I (1x; Thermo Fisher)] and the outcomes were analyzed against a six-point calibration curve generated with a naïve v.t.4h library cDNA sample of known concentrations.

Thermal cycling to recover library DNA was performed based on the outcomes of qPCR so as to avoid cDNA overamplification. SuperFi Pol (f.c.: 1x) was added to the elution product, and three stage thermal cycling ensued (denaturing at 95°C for 15 s, annealing at 61°C for 15 s, and extension at 72°C for 15 s), after which DNA isolation, transcription and puromycin ligation steps were performed as described in section 2.1.

2.4. NGS and data analysis

NGS. Tailed PCR was used to install Rd1 and Rd2 adapter sequences to library 5' and 3'-ends, respectively. cDNA recovered after the sixth round of selection was PCR-amplified in the SF PCR mix with appropriate primers (primers lists can be found in Table S2). The product was carried forward to the second PCR step, which used SuperFi Polymerase and Nextera XT v2 Set primers (sequences from Illumina) to install sequencing barcodes. The success of PCR was evaluated by 3% agarose gel electrophoresis and TapeStation. After, PCR product was column-purified with a NucleoSpin kit (TaKaRa) adhering to

manufacturer's protocol. The concentration of the sample was measured with Qubit (Thermo Fisher) using the dsDNA BR kit. cDNA was then appropriately diluted and denatured with 200 mM NaOH per Illumina's protocol. Denatured library [10 pM containing 10–50% (mol/mol) PhiX Control v3 (Illumina)] was sequenced on Illumina's MiSeq instrument in the single read 1×151 cycle mode using v3 chip, collecting data as .fastq files.

.fastq parser. Original python code is at *https://github.com/avngrdv/clibas*. Briefly, .fastq data files containing NGS base calls were parsed to retrieve DNA sequences, which were in silico translated. The resulting peptide lists were filtered to discard sequences of incorrect length, ORFs missing stop codons, peptides containing ambiguous symbols, and overly mutated sequences (those which had the constant regions [leader peptide and the C-terminal region of core peptide] with more than 5 mutations). Finally, for each remaining entry, constant regions were trimmed (leaving only random insert sequences), and sequencing Q scores corresponding to the variable region were inspected, discarding reads containing any Q scores below 30.

Data analysis. Sequences in the resulting peptide lists were counted, and the top 100 most abundant entries (Table S4) were used for multiple sequence alignment. To visualize sequence convergence (Fig. 3b), the top 1000 peptides were represented as matrices of positionally encoded amino acid-wise extended connectivity fingerprints (ECFPs)[9,10] and embedded with uniform manifold approximation and projection (**umap**; https://umap-learn.readthedocs.io/en/latest/index.html).[11]

2.5. Preparation of synthetic DNA for in vitro translation

PCR. Linear double-stranded DNA containing a T7 promoter sequence and ORFs encoding individual v.t.4h or v.t.4w library precursor peptides were assembled by PCR from synthetic single-stranded DNA oligonucleotides using Taq polymerase. All PCR were performed in 10 mM Tris (pH 8.4), 50 mM KCl, 0.1% (v/v) Triton X-100, 2.5 mM $MgCl_2$, 250 µM each dNTP supplemented with 500 nM of appropriate primers and Taq DNA polymerase. Three stage thermal cycling included a denaturing step at 95°C for 40 s, annealing at 52°C for 40 s, and extension at 72°C for 40 s. The list of all oligonucleotides and assembly schemes can be found in Tables S1 and S2.

In a one-step PCR assembly, template DNA (1:20000 dilution) was amplified with appropriate primers in Taq PCR solution for 13 cycles.

In a two-step PCR assembly, template DNA (1:2000 dilution) was first amplified in Taq PCR solution for 9 cycles, followed by a 13-cycle amplification of the PCR product from the first step (1 µL in 200 µL Taq PCR solution). Assembly outcomes were analyzed by 3% agarose gel electrophoresis stained with ethidium bromide. DNA was isolated following

phenol/chloroform extraction and ethanol precipitation steps as in section 2.1. Templates prepared in this way were used for in vitro translation without concentration adjustment or further purification.

2.6. Enzymatic reactions and LC/MS analysis of their outcomes

In vitro translation and enzymatic reactions. Template DNA encoding individual library clones were added (20%, v/v) to the transcription-coupled in vitro translation premix described in section 2.3; for *lazA* variants bearing an amber stop codon (TAG), the reaction mixture was supplemented with 1 μM release factor-1 (RF1).

Translation was allowed to proceed at 37 °C for 50 min, after which the mixtures were transferred on ice and split in half. One half was subjected to the enzymatic treatment as follows. The three-step LazDEF/BF/C enzymatic treatment was identical to that described in section 2.3. The two-step reaction was performed as originally reported.[12] Namely, to 1 volume of the translation product on ice was added LazDEF enzyme premix [1 volume containing 3 μM LazD, 4 μM LazE and 4 μM LazF in 100 mM Tris buffer (pH 8.0) supplemented with 20 mM $MgCl_2$, 9 mM ATP, and 2 mM DTT]. The mixture was transferred to a 25°C thermostat and incubated for 3 h. The tubes were then placed on ice, and a solution of iodoacetamide in water was added (f.c.: 5 mM). After 5 min on ice, 1 volume of LazBCF enzyme premix [4 μM LazB, 8 μM LazC, 2.2 μM LazF, 2 μM *S. lividans* GluRS, and 40 μM *S. lactacystinaeus* $tRNA^{Glu}$ in 50 mM Tris buffer (pH 8.0) supplemented with 10 mM $MgCl_2$, 4.5 mM ATP, and 0.5 mM glutamic acid] was added, and the reactions were allowed to proceed for 7 h at 25°C. The second half of the translation product was diluted into reaction buffer lacking enzymes [100 mM Tris buffer (pH 8.0) supplemented with 20 mM $MgCl_2$ and 9 mM ATP, and 2 mM DTT] and incubated at 25°C for 10 h. Such no-enzyme controls were utilized to gauge the fidelity of the translation reaction.

To stop the reactions, the tubes were transferred on ice, and 1.1 volume of iodoacetamide in methanol (30 mM) was added. The mixtures were incubated on ice for 15 min, followed by a 25 °C incubation for another 10 min. The samples were then centrifuged (15300 g for 5 min), and the supernatant (2–8 μL) was analyzed by LC/MS.

LC/MS analysis. Reaction outcomes were analyzed using Waters Xevo G2-XS QTof instrument equipped with Acquity I-Class UPLC system. HPLC was done on an Acquity UPLC Peptide BEH C18 column [dimensions: 150 x 2.1 mm; pore size: 300Å ; particle size: 1.7 μm] or an analogous C4 column using solvents A′ and B′ (section 1). Analysis was performed at 60°C and 300 μL/min flow rate with one of two methods listed below.

**LC/MS method 3**		**LC/MS method 4**	
Comparison of maturation protocols		*Selection hit analysis*	
0 – 1 min	1% B	0 – 1 min	1% B
1 – 12 min	1 – 71% B, linear ramp	1 – 13 min	1 – 81% B, linear ramp
12 – 13 min	95% B	13 – 14 min	95% B
13 – 15 min	1% B	14 – 16 min	1% B

MS analysis was done in a positive polarity/high sensitivity mode with a 0.3 s scan time. Capillary voltage was set to 700 V; ESI source and desolvation temperatures were 120 and 400°C, respectively. Manufacturer-supplied Leu-enkephalin was used as a lockspray standard for continuous mass axis referencing, and the lockspray setup procedure was performed according to the manufacturer's instructions prior to every run.

For tandem mass spectrometry, a data-dependent acquisition method was used. Collision-induced dissociation was triggered in real time if detected ion intensity exceeded $4 \cdot 10^4$ and z = 4, 5 or 6. MS/MS spectra were acquired with a 2 s scan time and parameterized collision energy values. In method 1, collision energies were set to ramp from 6–8 to 15–20 eV over the range of acquired m/z values (200 to 2000); in method 2, these values were 6–8 to 22–28 eV, and in method 3, 6–8 to 30–40 eV. Samples subject to MS/MS were reanalyzed three times to acquire MS/MS spectra for each method. All acquired spectra were analyzed, but for brevity, reported are only the most informative ones.

LC/MS data analysis. LC/MS data was analyzed with MassLynx v.4.1. To analyze translation outcomes, as well as efficiency of macrocyclization and formation of various linear forms after an enzymatic treatment, broad range extracted ion current (EIC) chromatograms were generated as previously reported[2] with *m/z* ± 100 (±500 Da) or *m/z* ± 250 tolerance window. Briefly, despite methanol precipitation, significant interference by the FIT system-derived small molecules in total ion current chromatograms was frequently observed. However, in general, the interfering compounds had a low molecular weight (<1500 Da), and thus, little interference in the *m/z* region above 1000, where most studied peptides were detected (z=5 in most cases), was observed. Therefore, generating EIC chromatograms for translated peptides and their reaction products at *z*=5 with *m/z* ±100 or ±250 tolerance window enabled visualization of reaction outcomes with minimal interference from the translation components.

To analyze formation of thiopeptides, a list of molecular weights corresponding to plausible products was generated in each case. A combination of broad and narrow range EIC corresponding to the generated masses as well as biggest peak intensity (BPI) chromatograms were utilized in tandem. Reported are combined EIC chromatograms for

observed thiopeptides, each generated with target *m/z* ±3 value cutoff unless otherwise specified. Low abundance (≤5% total) thiopeptides stemming from the misincorporation of proteinogenic amino acids in place of npAAs during translation are generally not reported/annotated.

Thiopeptide product distributions reported in Fig. 2c, d were quantified by integrating the areas for the corresponding thiopeptide-derived peaks under the assumption that the ionization efficiencies of thiopeptides derived from a single LazA precursor are uniform. Such analysis should be treated as semi-quantitative because the above assumption may not be fully accurate in some cases. Individual compound areas were calculated by summing areas under EIC chromatogram peaks for complete charged series, i.e.:

$$A_{pep} = \sum_{z} A_z^{EIC}(pep) \quad (1)$$

MS/MS assignments were done manually. For a given peptide, a series of possible post-translational modification (**PTM**) patterns was generated, and for each of these patterns, ladders of *b*- and *y*-ion series were computed. Calculated ion distributions were compared against experimental spectra, and the ion ladder leading to the best match was used to make spectral assignments.

2.7. Determination of thiopeptide structures

In addition to the prerequisite PTMs needed for macrocyclization, Laz enzymes can also install various PTMs by modifying Cys/Ser/Thr residues inside random inserts (Fig. S1). LazBF optionally (as determined by the substrate preferences of the enzyme) converts Ser and Thr to dehydroalanine (**Dha**) and *z*-dehydrobutyrine (**Dhb**). Likewise, LazDE can modify Ser and Thr to oxazolines (**Oxn**) and 5-methyloxazolines (**Oxn^{Me}**), respectively, which are often but not always dehydrogenated to oxazoles (**Oxz**) and 5-methyloxazoles (**Oxz^{Me}**). LazDE can additionally convert a Cys to a thiazoline (**Thn**), which can be further dehydrogenated by LazF to a thiazole (**Thz**). Unmodified Cys residues are alkylated by iodoacetamide. With this setup, every PTM type is characterized by a unique mass shift which simplifies structural assignments from LC/MS data. However, in most cases, some Cys/Ser/Thr residues remain unmodified, and while mass shifts can point to the total number of PTMs in a thiopeptide, they can not be used to identify the *location* of the modifications. The available substrate specificity data for Laz enzymes[3,4,10,13] can be utilized to only weakly support the assignments, because the enzymatic preferences in the presence of multiple npAAs as well multiple other PTMs remain poorly understood.

To address the structural assignment challenge, we employed tandem mass spectrometry to directly probe the location of PTMs. Macrocyclic thiopeptides are not amenable to MS/MS

because they require two fragmentation events to generate peptidyl fragments of unique masses required to make structural annotations. Therefore, we devised a strategy where precursor peptides of interest are treated with LazDEF and LazBF but not LazC (section 2.6) to produce linear, but otherwise fully decorated structures for MS/MS. To facilitate the spectral assignments, precursor peptides lacking 8 C-terminal amino acids in the linker region, i. e., those not critical for the enzymatic modification, were generated and analyzed in this way. As a second method to probe location of PTMs, maturation of single point mutants (mostly Cys/Ser/Thr → Ala) with LazDEF/BF/C was employed. DNA encoding single point mutants of precursor peptides in question were assembled as specified in section 2.5, and the enzymatic treatment and LC/MS analysis was as in section 2.6.

In many cases, both strategies were required to confidently determine thiopeptide structures. Incomplete fragmentation ladders observed during MS/MS prevented unambiguous assignments for some PTMs, whereas single point mutations occasionally led to further structural changes which obfuscated the effects of the mutations. Nevertheless, the combination of the two approaches enabled the determination for 13 out of 15 attempted structures. The summary of the data is provided in Fig. S18–31.

2.8. Surface plasmon resonance

In the initial experiment, binding affinities between the thiopeptides and TNIK were measured with a Biacore T200 instrument (GE Healthcare) at 25 °C. The assay buffer contained 50 mM Tris (pH 7.5), 150 mM NaCl, 10 mM $MgCl_2$, 0.01% (v/v) tween-20 and 0.1% (v/v) DMSO. For wTP3, the assay buffer additionally contained ATP (f.c.: 1 mM).

Biotinylated TNIK was immobilized on a Biacore CAP chip (GE Healthcare) to a surface density of 950-1200 response units following the standard immobilization protocols provided by the manufacturer. The interaction sensorgrams were measured as single-cycle kinetics performed with five concentrations of the ligand sequentially flown over the chip at a flow rate of 15 µL/min allowing for 20 or 30 s association and 70 or 60 s for dissociation between the steps. The final dissociation step was allowed to proceed for 180 s.

Next, for the six compounds which showed nM affinities to TNIK (Fig. 5a), the experiment was repeated in triplicate on a Biacore 8K instrument (GE Healthcare) using a multi cycle kinetics format. The experimental details were as above, except ATP was not added to the buffer for wTP3. Kinetic parameters and affinities of the interaction were determined assuming the standard 1:1 binding model using the Biacore software (GE Healthcare). Reported K_D values were calculated from the average measured association and dissociation rates (Table S5).

2.9. Kinase inhibition assays with ADP-Glo

The ADP-Glo assay system was purchased from Promega. The kinase reaction buffer contained 50 mM Tris (pH 7.5), 10 mM $MgCl_2$ and 0.01% (v/v) triton X-100. TNIK (1.25 nM) was incubated with various concentrations of analyte inhibitors in kinase reaction buffer at 25 °C for 10 min. After, the reactions were initiated by the addition of ATP and substrate peptide (MSTtide, sequence: NH_2-NKGYNTLRRKK-$CONH_2$;[ref 14] synthesized using the standard Fmoc SPPS techniques). The final mixtures containing 0.5 nM TNIK, 25 µM ATP, 15 µM MSTtide and variable concentration of the inhibitors were incubated at 25 °C for 30 min. The reactions were stopped by the addition of ADP-Glo reagent (1 volume), and the luminescent signal was developed following the manufacturer's instructions. Luminesce was recorded on a Tecan M1000 PRO microplate reader (Tecan Group) with 1 s acquisition time and no signal attenuation. TNIK kinase activity was normalized to a no inhibitor control, and IC_{50} values were obtained from non-linear regression (scipy)[15] of experimentally measured values to the standard 4-parameter logistic curve model.

Kinase selectivity profiling. A STE-1 kinase selectivity profiling kit from Promega was utilized to assay the compounds. The experiment was conducted following the manufacturer's instructions, and the signal was developed using the ADP-Glo assay system as described above. All measurements were performed in triplicate. After background correction, the kinase activities were normalized to a no inhibitor control, and percent remaining activity values were calculated accordingly.

2.10. Thiopeptide metabolic stability experiments

Stability to trypsin. To a solution of the analyte thiopeptide (15 µM) and the internal standard peptide (5 µM NH_2-PEG_5-wstndwdtnd-PEG_5-$CONH_2$;[ref 16] lower case encoding is for D-amino acids; synthesized using the standard Fmoc SPPS techniques) in digestion buffer [50 mM tris, 100 mM NaCl, 10 mM EDTA, pH 7.9] was added agarose-immobilized TPCK-treated trypsin (Thermo Fisher; 1 µL of resin slurry; 100 µL final reaction volume) that was previously washed with digestion buffer thrice. The mixture was agitated at 22 °C, and at various timepoints (0.5, 1, 2, 4, and 6 h) aliquots were withdrawn and quenched with 14 volumes of water/acetonitrile mixture [30: 70 (v/v) water: acetonitrile with 0.1% (v/v) TFA]. The samples were centrifuged (15300 g for 5 min) to remove agarose beads, and the supernatant (1 µL) was analyzed by LC/MS (section 2.6) using LC/MS method 5 as detailed below.

LC/MS method 5

Protease digestion experiments

0 – 1 min	1% B
1 – 12 min	1 – 81% B, linear ramp
12 – 13 min	95% B
13 – 15 min	1% B

The ratio of areas under the peak for the intact analyte over the internal standard (EIC chromatograms generated with *m/z* ± 3 cutoff values) were used to calculate the extent of analyte degradation. Thiopeptide half-life values were obtained from non-linear regression (scipy)[15] of experimentally measured values to the standard first order kinetic decomposition model.

Serum stability assay. To a solution of analyte thiopeptide (13.3 µM) and the internal standard peptide in TBS [5 µM; buffer: 50 mM Tris (pH 7.6), 150 mM NaCl] human serum (0.33 volumes; Cosmo Bio) was added on ice. The solutions were mixed well and transferred to a 37 °C thermostat. At various timepoints, aliquots were withdrawn and quenched with 2.3 volumes of methanol containing 30 mM iodoacetamide. The mixtures were incubated on ice for 15 min and then at 25 °C for 30 min. The samples were then centrifuged (15300 g for 5 min), and the supernatant (0.5 µL) was analyzed by LC/MS (section 2.5) using method 6 as detailed below.

LC/MS method 6

Serum stability assays

0 – 1 min	1% B
1 – 10 min	1 – 81% B, linear ramp
10 – 11 min	95% B
11 – 13 min	1% B

Data analysis was conducted as for the trypsin digestion assays. Additionally, facile demethylation of the C-terminal methyl ester was observed for some peptides (particularly, for hTP8 and wTP12). Such demethylated but otherwise undigested thiopeptides were integrated as intact compounds.

Stability to glutathione. To a freshly prepared solution of glutathione (5 mM) in stability buffer [50 mM HEPES (pH 7.5), 100 mM NaCl] was added a solution of analyte (1.5 mM thiopeptide in DMSO; f. c.: 10 µM). The contents were mixed and transferred to a 37 °C thermostat. At various time points (0.5, 1, 2, 4, 6, and 10 h) aliquots were withdrawn and quenched with 2 volumes of methanol containing 30 mM iodoacetamide. The samples were

analyzed by LC/MS (0.3 µL; section 2.6) using method 3. Areas under the peak curve for all thiopeptide-derived products in the sample (TIC chromatograms) were used to calculate fractions of intact analyte. Thiopeptide half-life values were obtained from non-linear regression (scipy)[15] of experimentally measured values to the pseudo-first order approximation for the reaction kinetics which can be used since [glutathione] >> [analyte].

2.11. Cell assays

HCT116 cells were cultured in McCoy's 5A (Modified) Medium supplemented with 1% (v/v) penicillin-streptomycin and 10% (v/v) fetal bovine serum (all from Thermo Fisher) in a 37 °C/5% CO_2 incubator.

All assays were conducted using thiopeptide acetate salts prepared as follows. Strong anion resin (AG® 1–X8 acetate form, analytical grade, quaternary ammonium 10~50 mg) was first washed three times with 1.6 N acetic acid and three times with 0.16 N acetic acid. Then, a thiopeptide solution (0.5~3 mg, dissolved in water/acetonitrile [1:1, v/v; ~1 ml]) was added to the resin. After 2 h on a rotary shaker, the peptide-resin suspension was filtered and the liquid phase was recovered. The resin was washed twice with water/acetonitrile (1:1, v/v; 0.5 ml/wash), and the solutions were combined. Thiopeptide acetates were isolated by lyophilization.

Cell viability assays. HCT116 cells were grown to 40–50% confluency in 96-well plates and treated with test thiopeptides, positive control (NCB0846, 10 µM) or DMSO, ensuring that the final DMSO concentration is 1% (v/v) for every sample. In the first experiment (Fig. S44a), the cells were grown for 24 h, and the incubation time was further extended to 48 h in the dose-dependent assay with hTP8 (Fig. S44b). In both cases, the cell viability was directly assayed with Cell Counting Kit-8 (Dojindo) following the manufacturer's instructions allowing 4 h to develop the signal. Absorbance at 450 nm was measured on a Tecan M1000 PRO microplate reader (Tecan Group), and live cell counts were normalized to the DMSO control. All experiments were performed in triplicate, and in most cases, replicated at least once to analogous outcomes.

RT-qPCR. HCT116 cells seeded in 12-well plates were treated as described above for 24 h. Subsequently, the medium was aspirated, and the cells were washed with cold PBS. Total cellular RNA was isolated using an RNeasy kit (Qiagen) following the manufacturer's instructions. RNA concentration was determined using a Nanodrop 2000c spectrophotometer (Thermo Scientific), and the quality of isolates was confirmed by TapeStation (RNA ScreenTape, Agilent Technologies; RIN^e values above 9.5 for every sample). Reverse transcription was performed with MMLV (H–) reverse transcriptase (Promega) and t20_anc.rt primer (sequence in Table S2) at 42 °C for 60 min following the

manufacturer's protocol. qPCR was performed using Platinum SuperFi II DNA Polymerase [SF PCR mix (section 2.3) supplemented with Sybr Green I (1x; Thermo Fisher) and 250 nM appropriate primers (Table S2)]. Thermal cycling conditions were as follows: denaturation at 95 °C for 15 s, annealing at 52 °C for 15 s, and extension at 72 °C for 30 s (40 cycles). Tm calling was conducted to confirm the homogeneity of the resulting amplicons in every case. The results were quantified using the standard ΔΔCq method using *GAPDH* as the reference gene and DMSO-treated samples as the reference samples. All experiments were performed in triplicate.

2.12. X-ray crystallography

TNIK expression and purification. For the structural study, TNIK (residues 11-314; Table S6) was expressed with an N-terminal Twin-Strep-His6 tag and a cleavage site for HRV 3C protease using a modified pET vector (Novagen) in E. coli BL21 (DE3) pLysS cells and purified by methods described previously.[12] After purification, the proteins in stock buffer (20 mM Tris-HCl pH 7.5, 150 mM NaCl, 0.05 mM DTT, 10% glycerol) were concentrated to 0.14 mM using VIVASPIN 15R, MWCO (molecular weight cut off) 10,000 (Sartorius).

Crystallization, X-ray data collection and structure determination. The sitting-drop vapor diffusion method was used for crystallization. For the crystallization of the TNIK·AMPPNP·wTP3 complex, purified TNIK (0.13 mM) in stock buffer was mixed with AMPPNP, $MgCl_2$, and wTP3 to final concentrations of 1 mM, 1 mM, and 0.26 mM, respectively. White precipitation, which formed after the addition of wTP3, was removed by centrifugation. The supernatant (100 nL) was mixed with 100 nL of a reservoir solution containing 13% Tacsimate pH 6.0 (Hampton Research) and incubated at 10 °C. Thin rod-like crystals, which grew in a few days, were cryoprotected with the reservoir solution supplemented with 30% Glycerol, 1 mM AMPPNP, 1 mM $MgCl_2$, 0.26 mM wTP3, and flash-frozen with liquid nitrogen.

The X-ray diffraction data were collected at SPring-8 BL32XU beamline with an automatic data collection system, ZOO.[17] Data sets from sixteen crystals of the TNIK·AMPPNP·wTP3 complex were automatically processed and merged using the programs XDS (version Jan10, 2022)[18] and KAMO.[19] The initial phases were calculated by the molecular replacement method using the program phaser 2.8.3[ref 20] with the TNIK structure (pdb 7xzq) as a search model.[12] Model building and refinement were carried out with the programs Coot 0.8.9[ref 21] and REFMAC 5.8[ref 22] implemented in CCP4 7.1.[ref 23] AceDRG[24] and eLBOW[25] were used to create the parameter files of the peptide for the refinement. The omit maps of the peptide and ligand were calculated using Phenix 1.19.2.[ref 26] Although AMPPNP was used during crystallization, the electron density at the ATP site was only visible for the adenosine moiety

(Fig. S46). Therefore, we modeled the bound molecule as adenosine. Structural figures were prepared with the program PyMOL 2.5.0 (Schrödinger, Inc.). A summary of data collection and refinement statistics is provided in Table S7.

3. Supplementary Figures

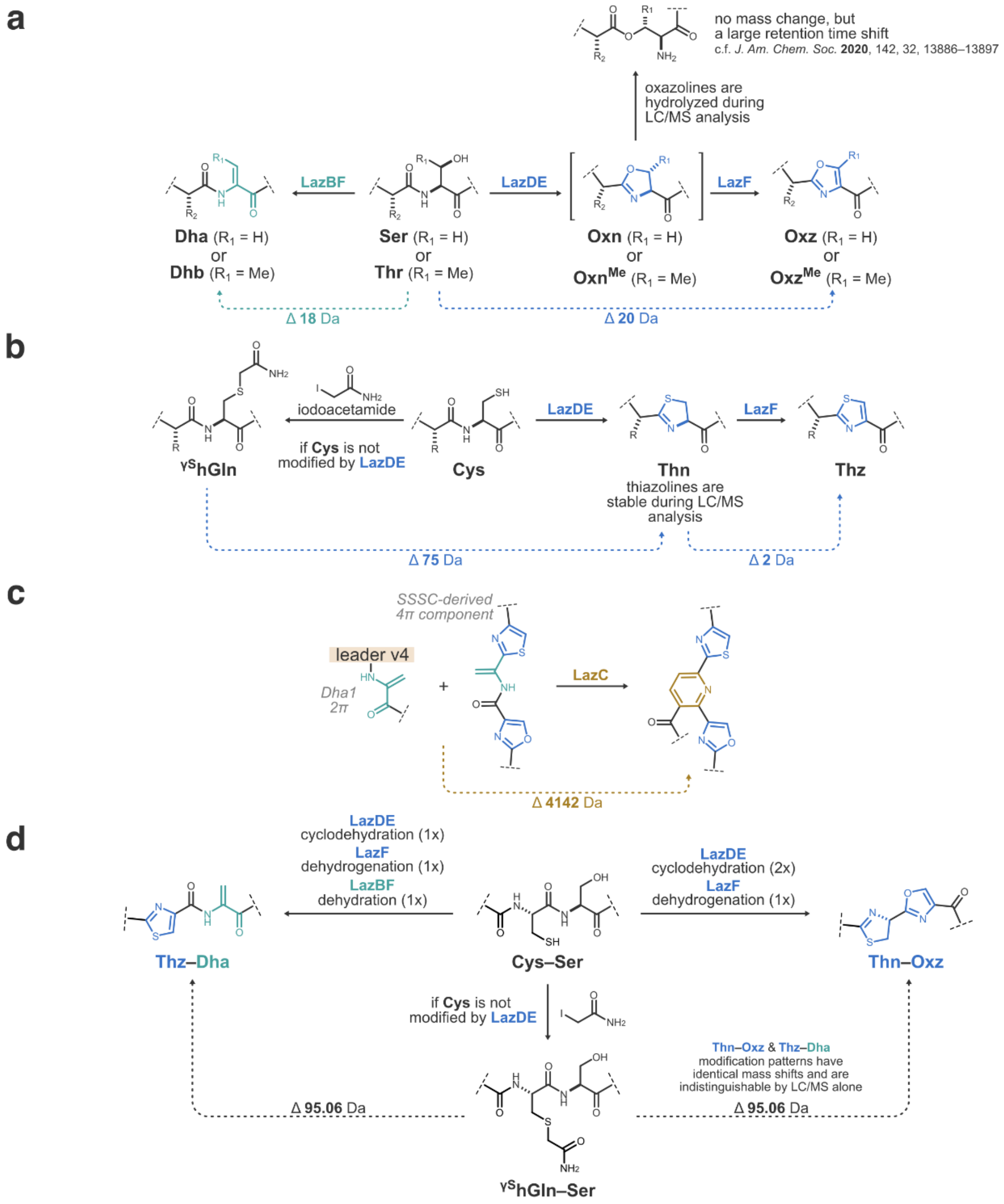

Figure S1. Post-translation modifications (**PTM**s) in the FIT-Laz system. **a**) Possible products of Ser and Thr modification by LazBF and LazDEF. **b**) Possible products of Cys modification by LazDEF. **c**) Pyridine formation catalyzed by LazC. Molecular weight shifts associated with each PTM type shown below the corresponding reactions. In the FIT-Laz system, every PTM type has a unique chemical mass shift which simplifies structural assignments for product thiopeptides. However, as shown in panel **d**), when multiple PTMs are present, unambiguous structural assignments are not always possible.

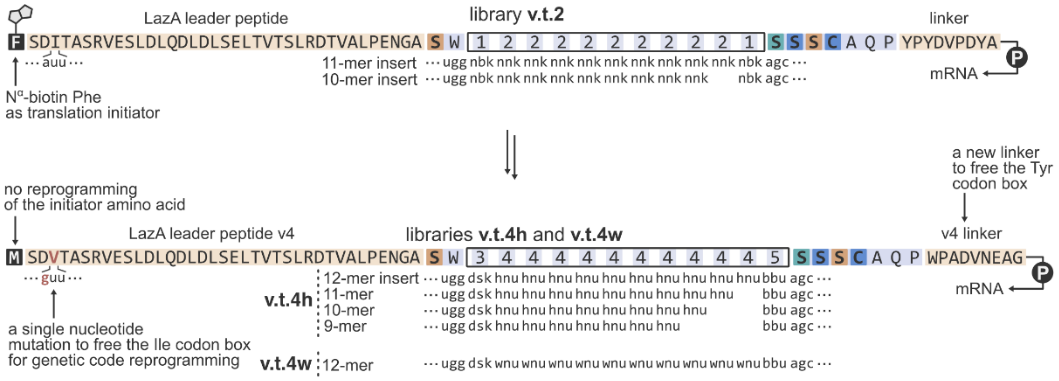

Figure S2. Design specifications for v.t.4 mRNA LazA-based libraries using the previously reported v.t.2 as the starting point. The main changes included mutating the leader peptide and C-terminal linker as well as changing the composition of the random insert. See also Fig. 1 and Table S1 for additional details.

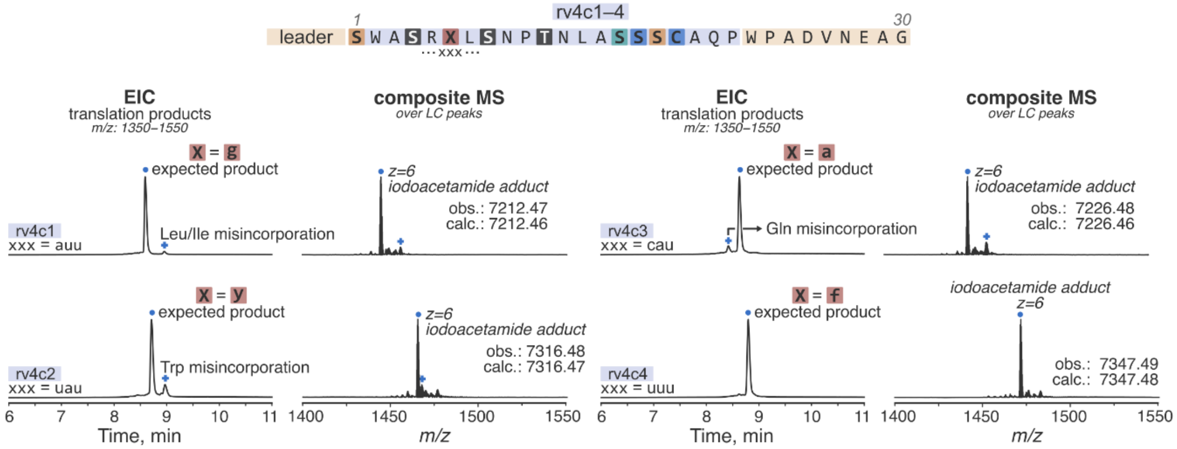

Figure S3. In vitro translation of v.t.4 precursor peptides with genetic code reprogramming: evaluating the incorporation efficiency of individual npAAs in model templates. Peptides *rv4c1-4* were expressed with the FIT system using the reprogrammed genetic code as described in section 2.6; translation outcomes were analyzed by LC/MS. Displayed are extracted ion current (EIC) chromatograms (*m/z*: 1350-1550) and composite MS spectra integrated over peptide-derived peaks (*m/z*: 1400-1550, i. e., the region corresponding to the LC peaks in the chromatograms on the left). Clean incorporation of all chosen amino acids was observed. Minor misincorporation of npAAs into various proteinogenic amino acids was apparent in three out of the four cases.

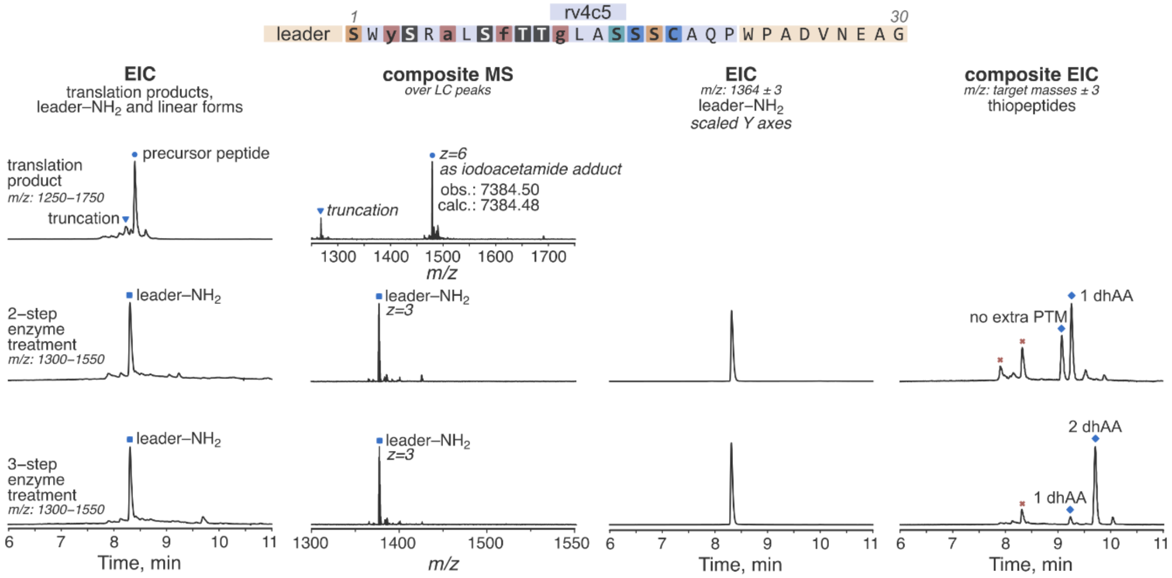

Figure S4. Maturation of random v.t.4 precursor peptides using the original two-step and the reengineered three-step enzymatic treatment protocols. Peptide *rv4c5* was expressed with the FIT system using the reprogrammed genetic code as described and treated with Laz enzymes as described in section 2.6; the outcomes were analyzed by LC/MS. Displayed are extracted ion current (EIC) chromatograms [*m/z*: 1250-1750 (visualizes translation products) or 1300-1550 (shows the accumulation of linear forms and leader-NH_2)]; composite MS spectra integrated over substrate-derived peaks (*m/z*: 1300-1550, i. e., the region corresponding to the LC peaks in the chromatograms on the left); EIC chromatograms for leader-NH_2 (*m/z*: 1362.7 ± 3; scaled Y-axes to enable relative quantification of macrocyclization yields); and composite EICs for all detected thiopeptide products (scaled Y-axes). The results for the original two-step (LazDEF/LazBC) and reengineered (LazDEF/LazBF/LazC) treatments are stacked for comparison. In the EIC chromatograms for thiopeptides, the peaks labelled with a red cross correspond to thiopeptide-unrelated compounds.

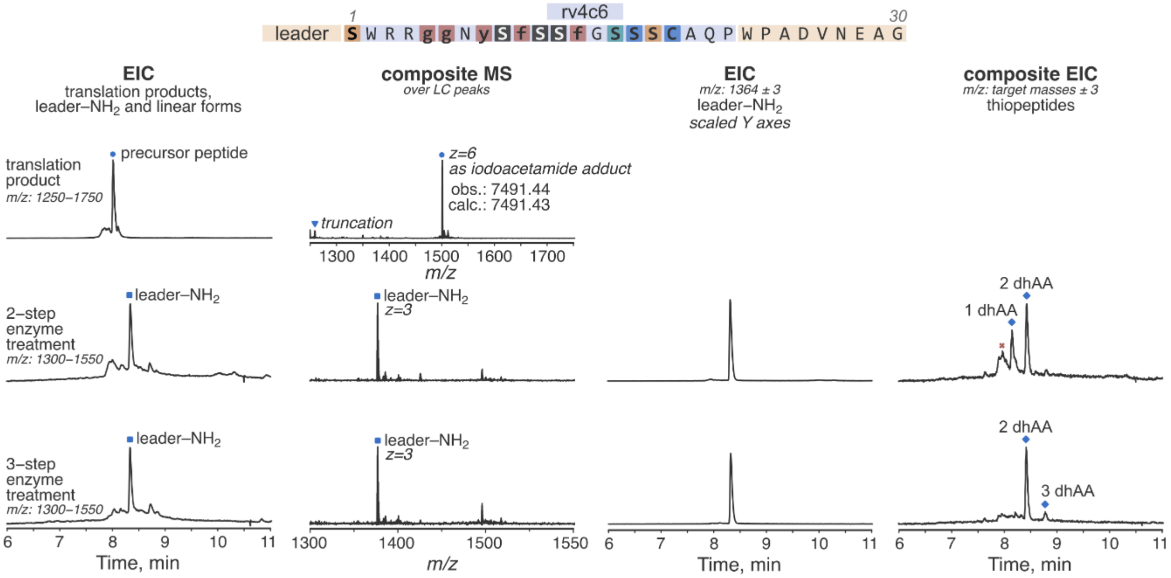

Figure S5. Maturation of random v.t.4 precursor peptides using the original two-step and the reengineered three-step enzymatic treatment protocols. Peptide *rv4c6* was expressed with the FIT system using the reprogrammed genetic code as described and treated with Laz enzymes as described in section 2.6; the outcomes were analyzed by LC/MS. Displayed are extracted ion current (EIC) chromatograms [*m/z*: 1250-1750 (visualizes translation products) or 1300-1550 (shows the accumulation of linear forms and leader-NH_2)]; composite MS spectra integrated over substrate-derived peaks (*m/z*: 1300-1550, i. e., the region corresponding to the LC peaks in the chromatograms on the left); EIC chromatograms for leader-NH_2 (*m/z*: 1362.7 ± 3; scaled Y-axes to enable relative quantification of macrocyclization yields); and composite EICs for all detected thiopeptide products (scaled Y-axes). The results for the original two-step (LazDEF/LazBC) and reengineered (LazDEF/LazBF/LazC) treatments are stacked for comparison. In the EIC chromatograms for thiopeptides, the peaks labelled with a red cross correspond to thiopeptide-unrelated compounds.

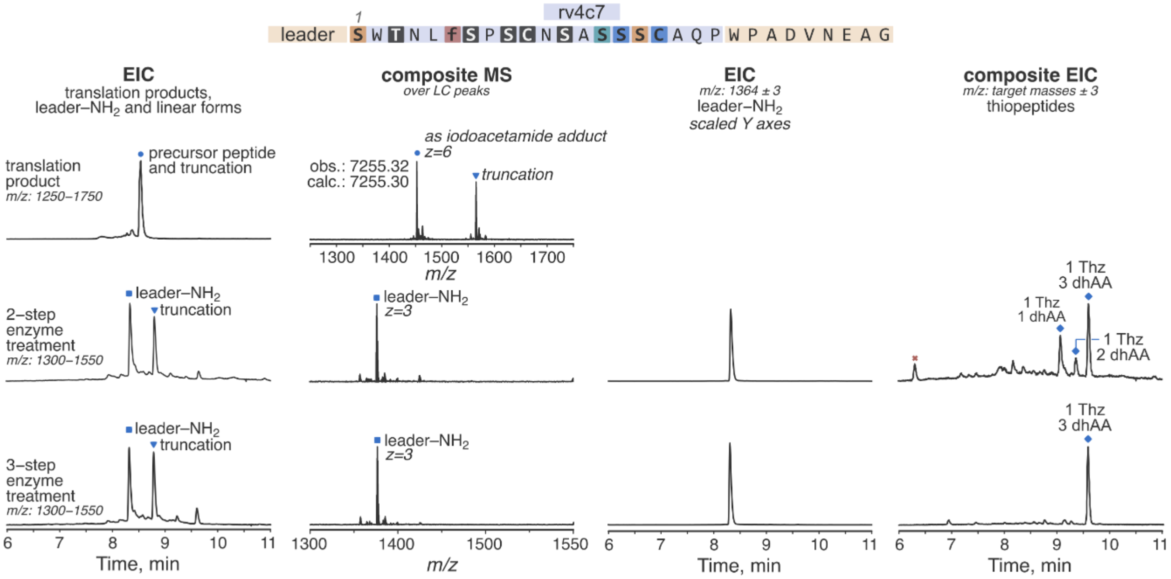

Figure S6. Maturation of random v.t.4 precursor peptides using the original two-step and the reengineered three-step enzymatic treatment protocols. Peptide *rv4c5* was expressed with the FIT system using the reprogrammed genetic code as described and treated with Laz enzymes as described in section 2.6; the outcomes were analyzed by LC/MS. Displayed are extracted ion current (EIC) chromatograms [*m/z*: 1250-1750 (visualizes translation products) or 1300-1550 (shows the accumulation of linear forms and leader-NH_2)]; composite MS spectra integrated over substrate-derived peaks (*m/z*: 1300-1550, i. e., the region corresponding to the LC peaks in the chromatograms on the left); EIC chromatograms for leader-NH_2 (*m/z*: 1362.7 ± 3; scaled Y-axes to enable relative quantification of macrocyclization yields); and composite EICs for all detected thiopeptide products (scaled Y-axes). The results for the original two-step (LazDEF/LazBC) and reengineered (LazDEF/LazBF/LazC) treatments are stacked for comparison. In the EIC chromatograms for thiopeptides, the peaks labelled with a red cross correspond to thiopeptide-unrelated compounds.

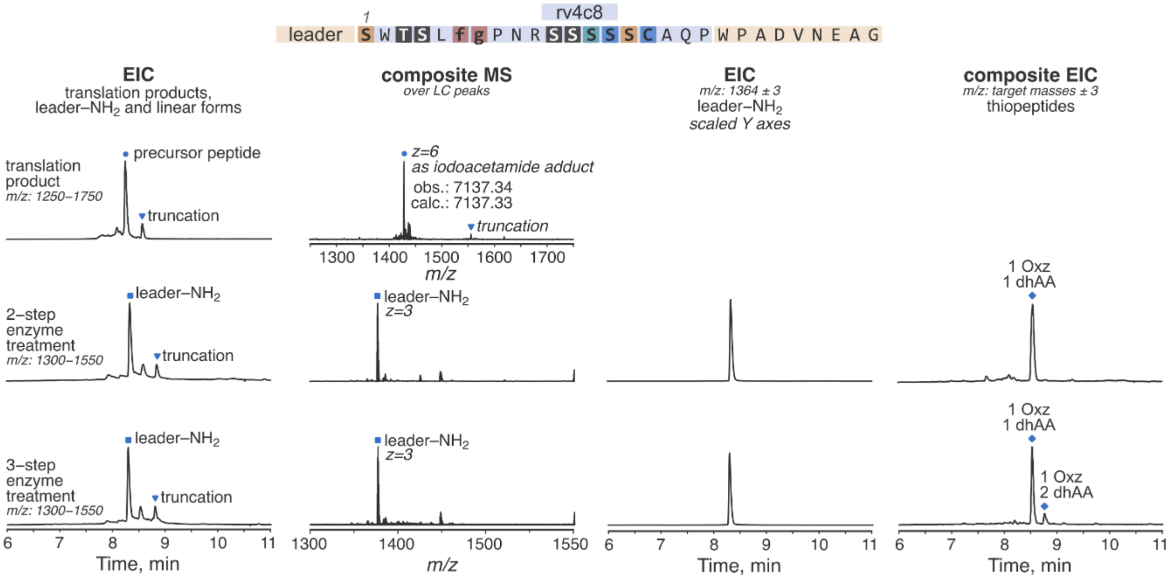

Figure S7. Maturation of random v.t.4 precursor peptides using the original two-step and the reengineered three-step enzymatic treatment protocols. Peptide *rv4c8* was expressed with the FIT system using the reprogrammed genetic code as described and treated with Laz enzymes as described in section 2.6; the outcomes were analyzed by LC/MS. Displayed are extracted ion current (EIC) chromatograms [*m/z*: 1250-1750 (visualizes translation products) or 1300-1550 (shows the accumulation of linear forms and leader-NH_2)]; composite MS spectra integrated over substrate-derived peaks (*m/z*: 1300-1550, i. e., the region corresponding to the LC peaks in the chromatograms on the left); EIC chromatograms for leader-NH_2 (*m/z*: 1362.7 ± 3; scaled Y-axes to enable relative quantification of macrocyclization yields); and composite EICs for all detected thiopeptide products (scaled Y-axes). The results for the original two-step (LazDEF/LazBC) and reengineered (LazDEF/LazBF/LazC) treatments are stacked for comparison.

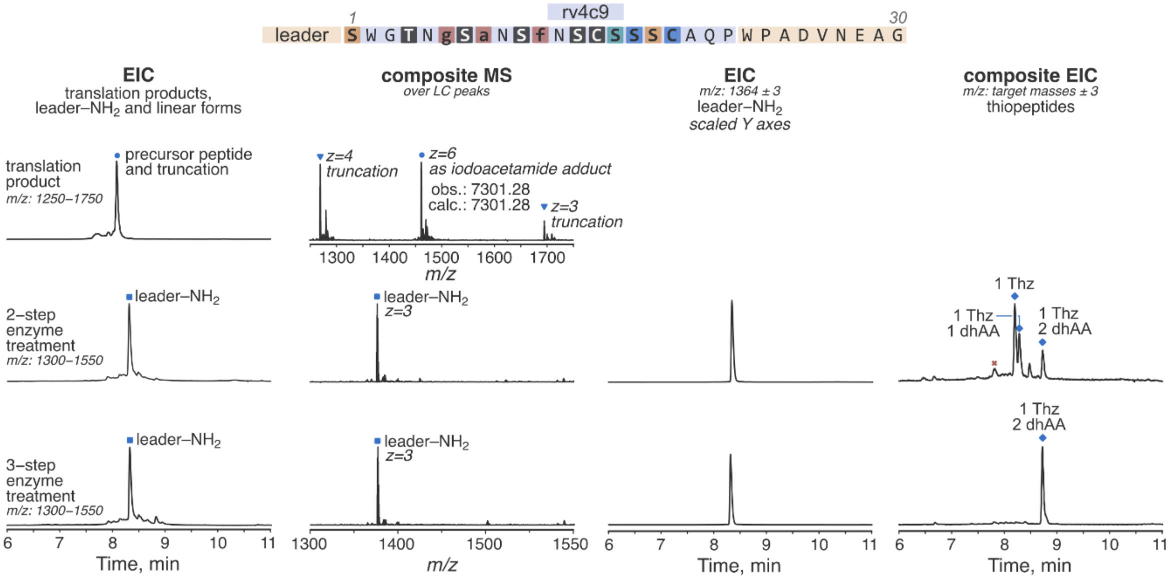

Figure S8. Maturation of random v.t.4 precursor peptides using the original two-step and the reengineered three-step enzymatic treatment protocols. Peptide *rv4c9* was expressed with the FIT system using the reprogrammed genetic code as described and treated with Laz enzymes as described in section 2.6; the outcomes were analyzed by LC/MS. Displayed are extracted ion current (EIC) chromatograms [*m/z*: 1250-1750 (visualizes translation products) or 1300-1550 (shows the accumulation of linear forms and leader-NH_2)]; composite MS spectra integrated over substrate-derived peaks (*m/z*: 1300-1550, i. e., the region corresponding to the LC peaks in the chromatograms on the left); EIC chromatograms for leader-NH_2 (*m/z*: 1362.7 ± 3; scaled Y-axes to enable relative quantification of macrocyclization yields); and composite EICs for all detected thiopeptide products (scaled Y-axes). The results for the original two-step (LazDEF/LazBC) and reengineered (LazDEF/LazBF/LazC) treatments are stacked for comparison. In the EIC chromatograms for thiopeptides, the peaks labelled with a red cross correspond to thiopeptide-unrelated compounds.

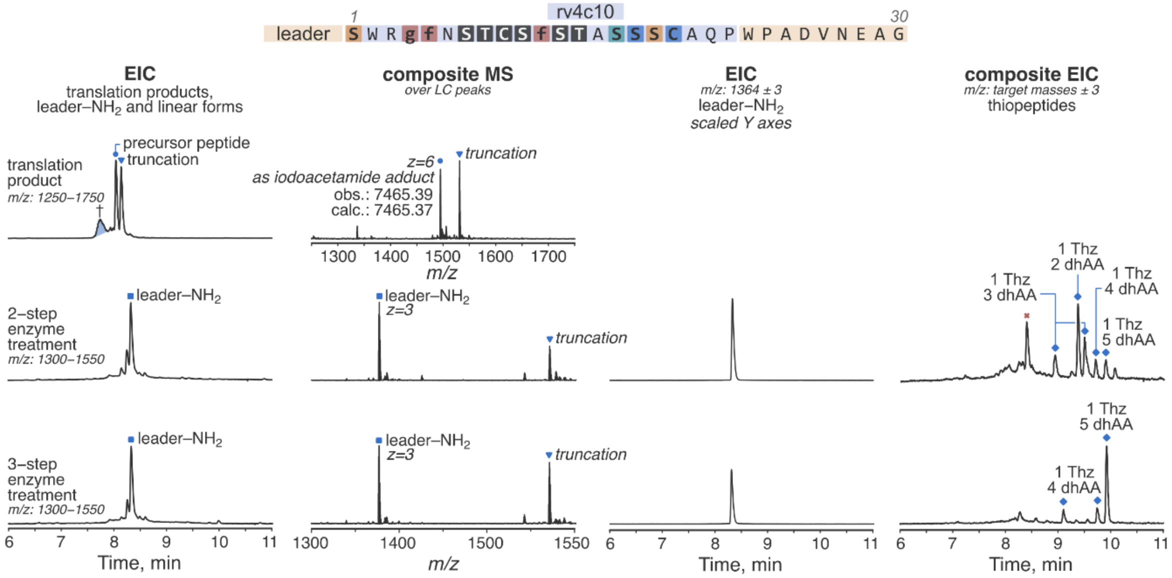

Figure S9. Maturation of random v.t.4 precursor peptides using the original two-step and the reengineered three-step enzymatic treatment protocols. Peptide *rv4c10* was expressed with the FIT system using the reprogrammed genetic code as described and treated with Laz enzymes as described in section 2.6; the outcomes were analyzed by LC/MS. Displayed are extracted ion current (EIC) chromatograms [*m/z*: 1250-1750 (visualizes translation products) or 1300-1550 (shows the accumulation of linear forms and leader-NH_2)]; composite MS spectra integrated over substrate-derived peaks (*m/z*: 1300-1550, i. e., the region corresponding to the LC peaks in the chromatograms on the left); EIC chromatograms for leader-NH_2 (*m/z*: 1362.7 ± 3; scaled Y-axes to enable relative quantification of macrocyclization yields); and composite EICs for all detected thiopeptide products (scaled Y-axes). The results for the original two-step (LazDEF/LazBC) and reengineered (LazDEF/LazBF/LazC) treatments are stacked for comparison. In the EIC chromatograms for thiopeptides, the peaks labelled with a red cross correspond to thiopeptide-unrelated compounds. †: peak derived from the carryover of translation proteins.

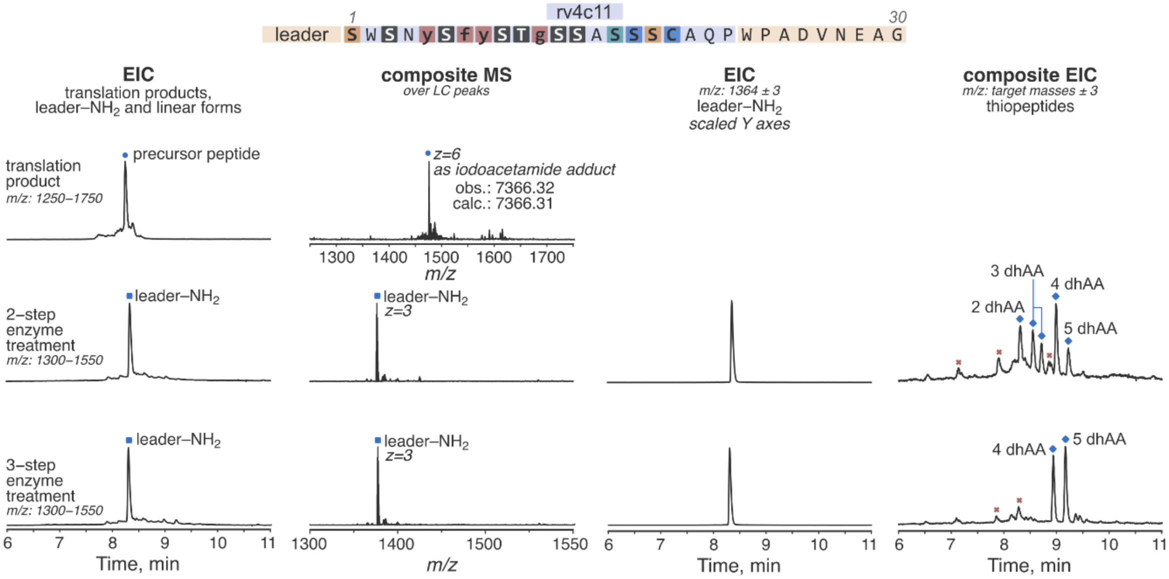

Figure S10. Maturation of random v.t.4 precursor peptides using the original two-step and the reengineered three-step enzymatic treatment protocols. Peptide *rv4c11* was expressed with the FIT system using the reprogrammed genetic code as described and treated with Laz enzymes as described in section 2.6; the outcomes were analyzed by LC/MS. Displayed are extracted ion current (EIC) chromatograms [*m/z*: 1250-1750 (visualizes translation products) or 1300-1550 (shows the accumulation of linear forms and leader-NH_2)]; composite MS spectra integrated over substrate-derived peaks (*m/z*: 1300-1550, i. e., the region corresponding to the LC peaks in the chromatograms on the left); EIC chromatograms for leader-NH_2 (*m/z*: 1362.7 ± 3; scaled Y-axes to enable relative quantification of macrocyclization yields); and composite EICs for all detected thiopeptide products (scaled Y-axes). The results for the original two-step (LazDEF/LazBC) and reengineered (LazDEF/LazBF/LazC) treatments are stacked for comparison. In the EIC chromatograms for thiopeptides, the peaks labelled with a red cross correspond to thiopeptide-unrelated compounds.

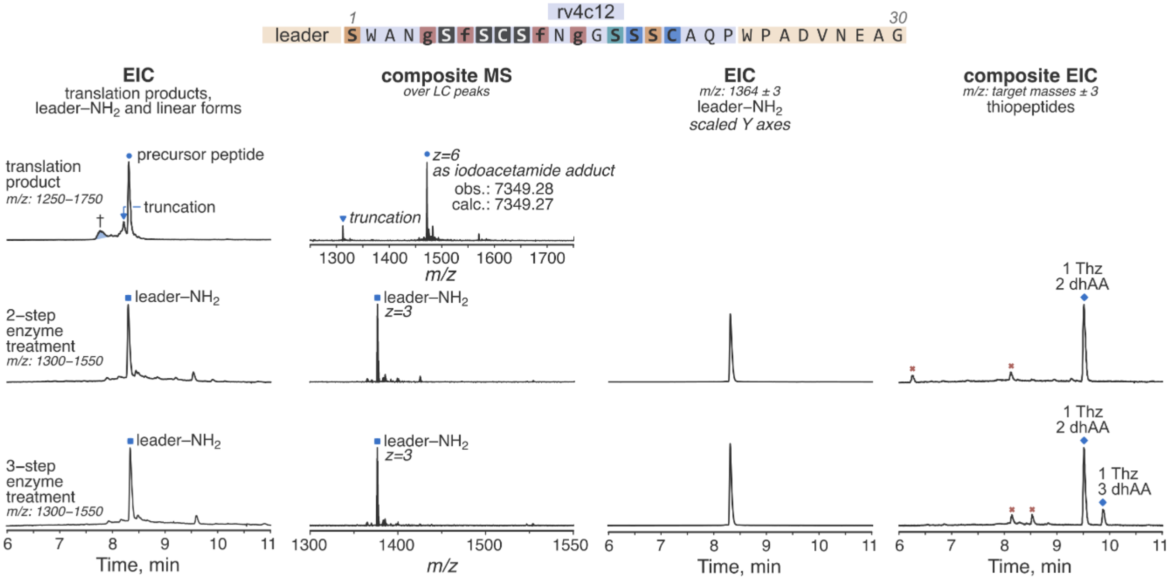

Figure S11. Maturation of random v.t.4 precursor peptides using the original two-step and the reengineered three-step enzymatic treatment protocols. Peptide *rv4c12* was expressed with the FIT system using the reprogrammed genetic code as described and treated with Laz enzymes as described in section 2.6; the outcomes were analyzed by LC/MS. Displayed are extracted ion current (EIC) chromatograms [*m/z*: 1250-1750 (visualizes translation products) or 1300-1550 (shows the accumulation of linear forms and leader-NH_2)]; composite MS spectra integrated over substrate-derived peaks (*m/z*: 1300-1550, i. e., the region corresponding to the LC peaks in the chromatograms on the left); EIC chromatograms for leader-NH_2 (*m/z*: 1362.7 ± 3; scaled Y-axes to enable relative quantification of macrocyclization yields); and composite EICs for all detected thiopeptide products (scaled Y-axes). The results for the original two-step (LazDEF/LazBC) and reengineered (LazDEF/LazBF/LazC) treatments are stacked for comparison. In the EIC chromatograms for thiopeptides, the peaks labelled with a red cross correspond to thiopeptide-unrelated compounds. †: peak derived from the carryover of translation proteins.

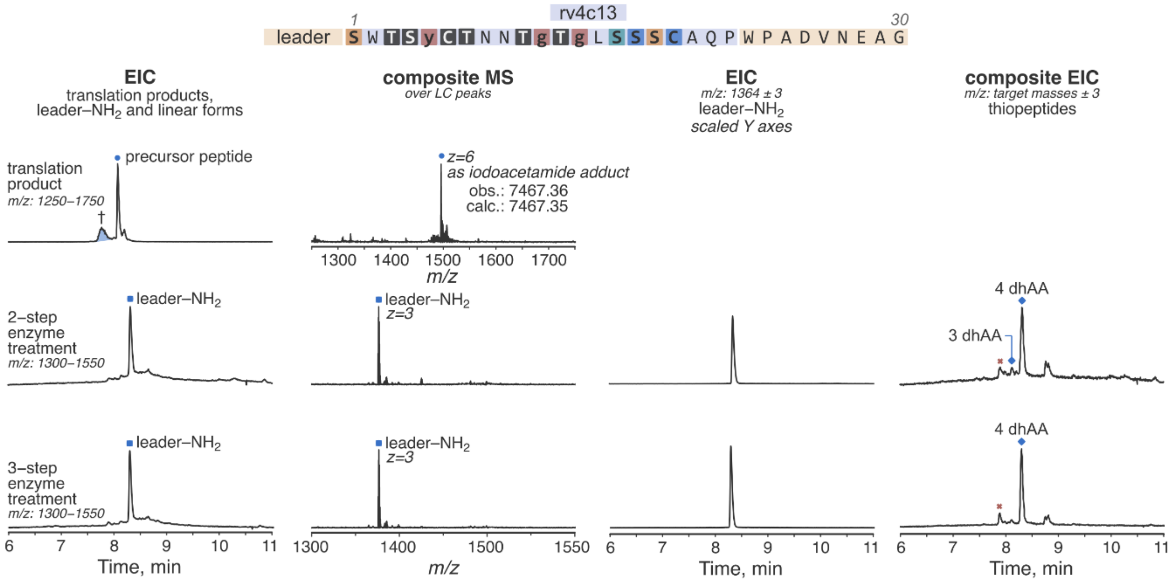

Figure S12. Maturation of random v.t.4 precursor peptides using the original two-step and the reengineered three-step enzymatic treatment protocols. Peptide *rv4c13* was expressed with the FIT system using the reprogrammed genetic code as described and treated with Laz enzymes as described in section 2.6; the outcomes were analyzed by LC/MS. Displayed are extracted ion current (EIC) chromatograms [*m/z*: 1250-1750 (visualizes translation products) or 1300-1550 (shows the accumulation of linear forms and leader-NH_2)]; composite MS spectra integrated over substrate-derived peaks (*m/z*: 1300-1550, i. e., the region corresponding to the LC peaks in the chromatograms on the left); EIC chromatograms for leader-NH_2 (*m/z*: 1362.7 ± 3; scaled Y-axes to enable relative quantification of macrocyclization yields); and composite EICs for all detected thiopeptide products (scaled Y-axes). The results for the original two-step (LazDEF/LazBC) and reengineered (LazDEF/LazBF/LazC) treatments are stacked for comparison. In the EIC chromatograms for thiopeptides, the peaks labelled with a red cross correspond to thiopeptide-unrelated compounds. †: peak derived from the carryover of translation proteins.

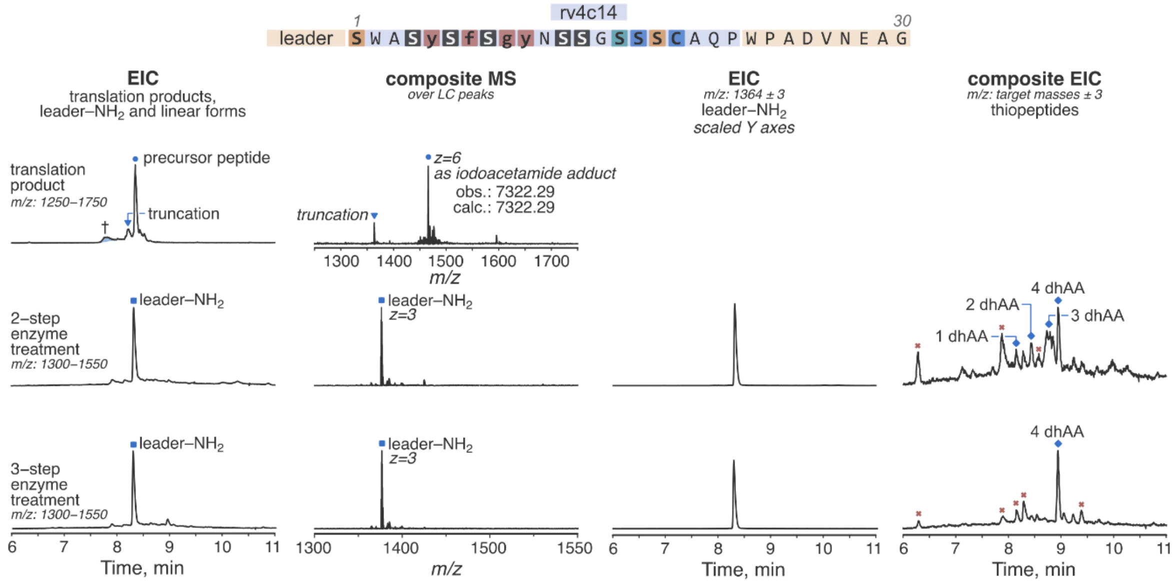

Figure S13. Maturation of random v.t.4 precursor peptides using the original two-step and the reengineered three-step enzymatic treatment protocols. Peptide *rv4c14* was expressed with the FIT system using the reprogrammed genetic code as described and treated with Laz enzymes as described in section 2.6; the outcomes were analyzed by LC/MS. Displayed are extracted ion current (EIC) chromatograms [*m/z*: 1250-1750 (visualizes translation products) or 1300-1550 (shows the accumulation of linear forms and leader-NH_2)]; composite MS spectra integrated over substrate-derived peaks (*m/z*: 1300-1550, i. e., the region corresponding to the LC peaks in the chromatograms on the left); EIC chromatograms for leader-NH_2 (*m/z*: 1362.7 ± 3; scaled Y-axes to enable relative quantification of macrocyclization yields); and composite EICs for all detected thiopeptide products (scaled Y-axes). The results for the original two-step (LazDEF/LazBC) and reengineered (LazDEF/LazBF/LazC) treatments are stacked for comparison. In the EIC chromatograms for thiopeptides, the peaks labelled with a red cross correspond to thiopeptide-unrelated compounds. †: peak derived from the carryover of translation proteins.

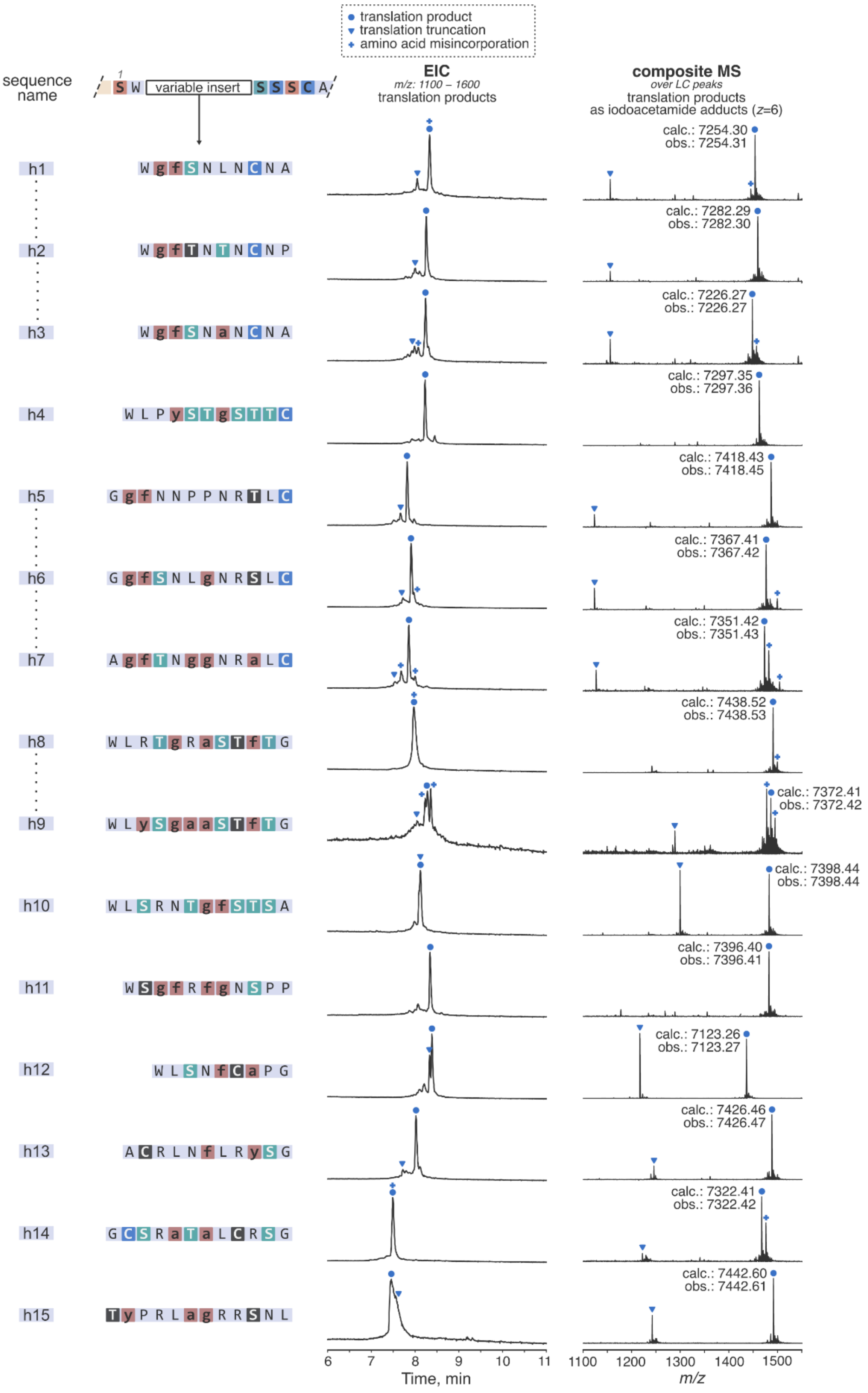

translation product
translation truncation
amino acid misincorporation
sequence name
variable insert
EIC
m/z: 1100 – 1600
translation products
composite MS
over LC peaks
translation products
as iodoacetamide adducts (z=6)
h1 W g f S N L N C N A
calc.: 7254.30
obs.: 7254.31
h2 W g f T N T N C N P
calc.: 7282.29
obs.: 7282.30
h3 W g f S N a N C N A
calc.: 7226.27
obs.: 7226.27
h4 W L P y S T g S T T C
calc.: 7297.35
obs.: 7297.36
h5 G g f N N P P N R T L C
calc.: 7418.43
obs.: 7418.45
h6 G g f S N L g N R S L C
calc.: 7367.41
obs.: 7367.42
h7 A g f T N g g N R a L C
calc.: 7351.42
obs.: 7351.43
h8 W L R T g R a S T f T G
calc.: 7438.52
obs.: 7438.53
h9 W L y S g a a S T f T G
calc.: 7372.41
obs.: 7372.42
h10 W L S R N T g f S T S A
calc.: 7398.44
obs.: 7398.44
h11 W S g f R f g N S P P
calc.: 7396.40
obs.: 7396.41
h12 W L S N f C a P G
calc.: 7123.26
obs.: 7123.27
h13 A C R L N f L R y S G
calc.: 7426.46
obs.: 7426.47
h14 G C S R a T a L C R S G
calc.: 7322.41
obs.: 7322.42
h15 T y P R L a g R R S N L
calc.: 7442.60
obs.: 7442.61
Time, min
m/z

Figure S14. In vitro translation of v.t.4h selection hits (precursor peptides) with genetic code reprogramming. Peptides *h1–15* were expressed with the FIT system using the reprogrammed genetic code as described in section 2.6; translation outcomes were analyzed by LC/MS. Displayed are extracted ion current (EIC) chromatograms (*m/z*: 1100-1600) and composite MS spectra integrated over peptide-derived peaks (*m/z*: 1100-1550, i. e., the region corresponding to the LC peaks in the chromatograms on the left). Expected translation products were observed in every case; translational truncation products were the main source of heterogeneity; major misincorporation of proteinogenic amino acids in place of npAA was also observed in several cases.

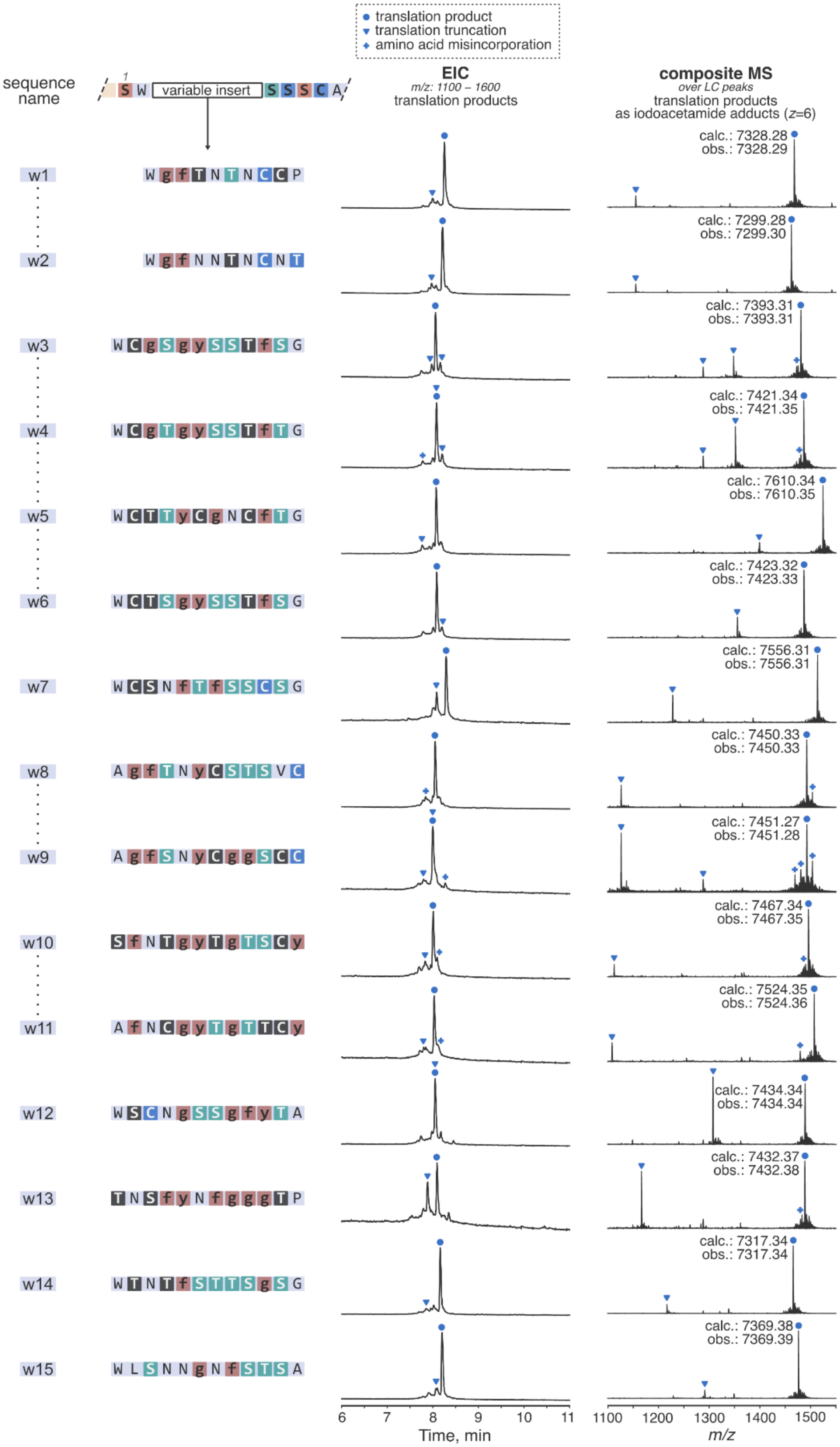
translation product
translation truncation
amino acid misincorporation
sequence name
1
S W variable insert S S S C A
EIC
m/z: 1100 – 1600
translation products
composite MS
over LC peaks
translation products
as iodoacetamide adducts (z=6)
w1 W g f T N T N C C P
calc.: 7328.28
obs.: 7328.29
w2 W g f N N T N C N T
calc.: 7299.28
obs.: 7299.30
w3 W C g S g y S S T f S G
calc.: 7393.31
obs.: 7393.31
w4 W C g T g y S S T f T G
calc.: 7421.34
obs.: 7421.35
w5 W C T T y C g N C f T G
calc.: 7610.34
obs.: 7610.35
w6 W C T S g y S S T f S G
calc.: 7423.32
obs.: 7423.33
w7 W C S N f T f S S C S G
calc.: 7556.31
obs.: 7556.31
w8 A g f T N y C S T S V C
calc.: 7450.33
obs.: 7450.33
w9 A g f S N y C g g S C C
calc.: 7451.27
obs.: 7451.28
w10 S f N T g y T g T S C y
calc.: 7467.34
obs.: 7467.35
w11 A f N C g y T g T T C y
calc.: 7524.35
obs.: 7524.36
w12 W S C N g S S g f y T A
calc.: 7434.34
obs.: 7434.34
w13 T N S f y N f g g g T P
calc.: 7432.37
obs.: 7432.38
w14 W T N T f S T T S g S G
calc.: 7317.34
obs.: 7317.34
w15 W L S N N g N f S T S A
calc.: 7369.38
obs.: 7369.39
6 7 8 9 10 11
Time, min
1100 1200 1300 1400 1500
m/z

Figure S15. In vitro translation of v.t.4w TNIK selection hits (precursor peptides) with genetic code reprogramming. Peptides *w1–15* were expressed with the FIT system using the reprogrammed genetic code as described in section 2.6; translation outcomes were analyzed by LC/MS. Displayed are extracted ion current (EIC) chromatograms (*m/z*: 1100-1600) and composite MS spectra integrated over peptide-derived peaks (*m/z*: 1100-1550, i. e., the region corresponding to the LC peaks in the chromatograms on the left). Expected translation products were observed in every case; translational truncation products were the main source of heterogeneity; minor misincorporation of proteinogenic amino acids in place of npAA was also observed in most cases.

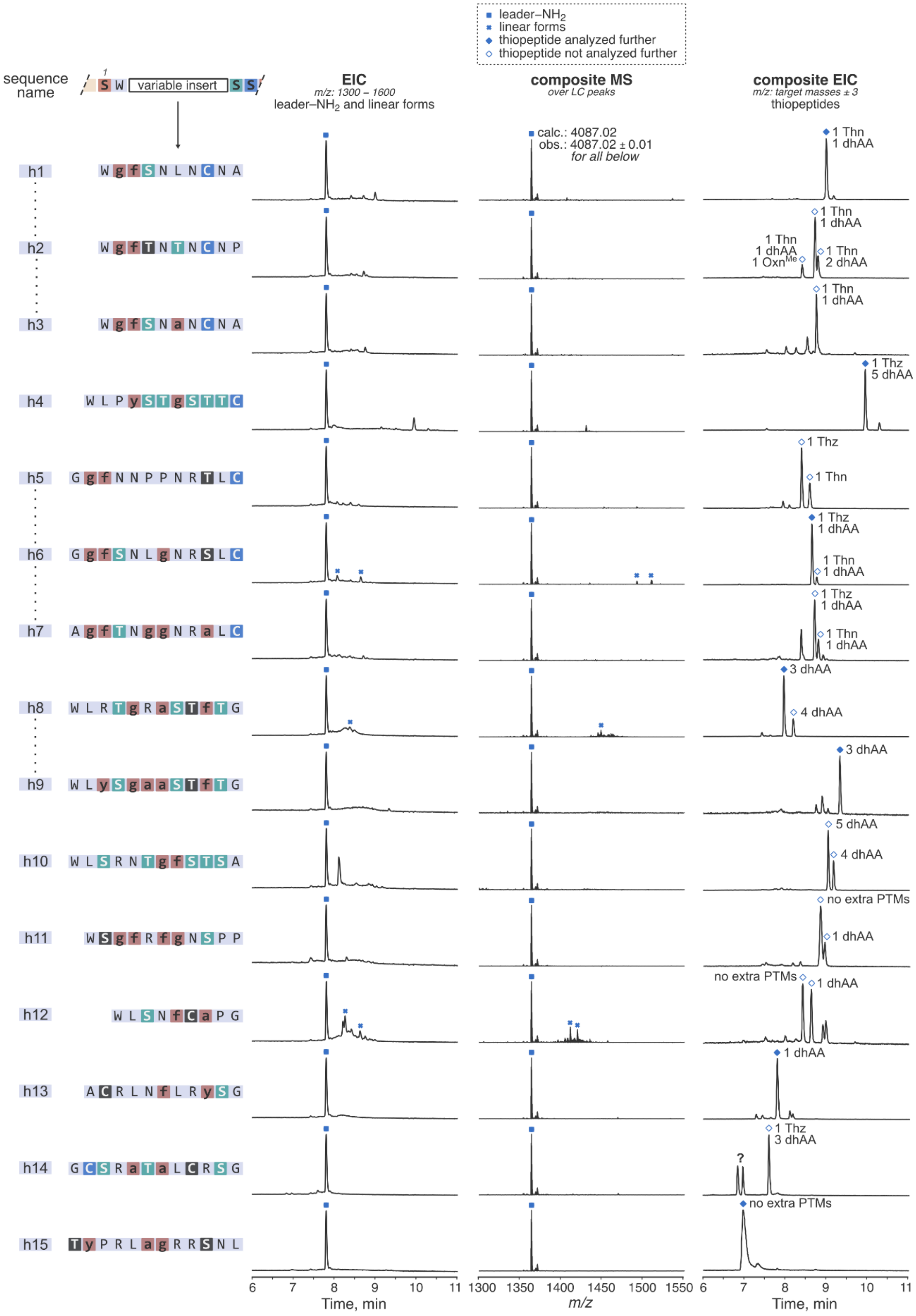
leader–NH2
linear forms
thiopeptide analyzed further
thiopeptide not analyzed further
sequence name
variable insert
EIC
m/z: 1300 – 1600
leader–NH2 and linear forms
composite MS
over LC peaks
composite EIC
m/z: target masses ± 3
thiopeptides
calc.: 4087.02
obs.: 4087.02 ± 0.01
for all below
h1
h2
h3
h4
h5
h6
h7
h8
h9
h10
h11
h12
h13
h14
h15
1 Thn
1 dhAA
1 Oxn
2 dhAA
1 Thz
5 dhAA
3 dhAA
4 dhAA
no extra PTMs
Time, min
m/z

Figure S16. Maturation of v.t.4h TNIK selection hits using the three-step enzymatic treatment protocol. Precursor peptides *h1–15* expressed with the FIT system as shown in Fig. S14 were treated with Laz enzymes as described in section 2.6; the outcomes were analyzed by LC/MS. Displayed are extracted ion current (EIC) chromatograms (*m/z*: 1300-1600 to visualize the accumulation of linear forms and leader-NH_2); composite MS spectra integrated over substrate-derived peaks (*m/z*: 1300-1550, i. e., the region corresponding to the LC peaks in the chromatograms on the left); and composite EICs for all detected thiopeptide products (scaled Y-axes). In the EIC chromatograms for thiopeptides, thiopeptide-unrelated compounds as well as minor thiopeptides stemming from the misincorporation of proteinogenic amino acids in place of npAAs are unannotated. Maturation of h14 led to the formation of two unidentified products labelled with a "?" mark. Clean conversion of precursor peptides into thiopeptides was observed in 14 out of the 15 cases.

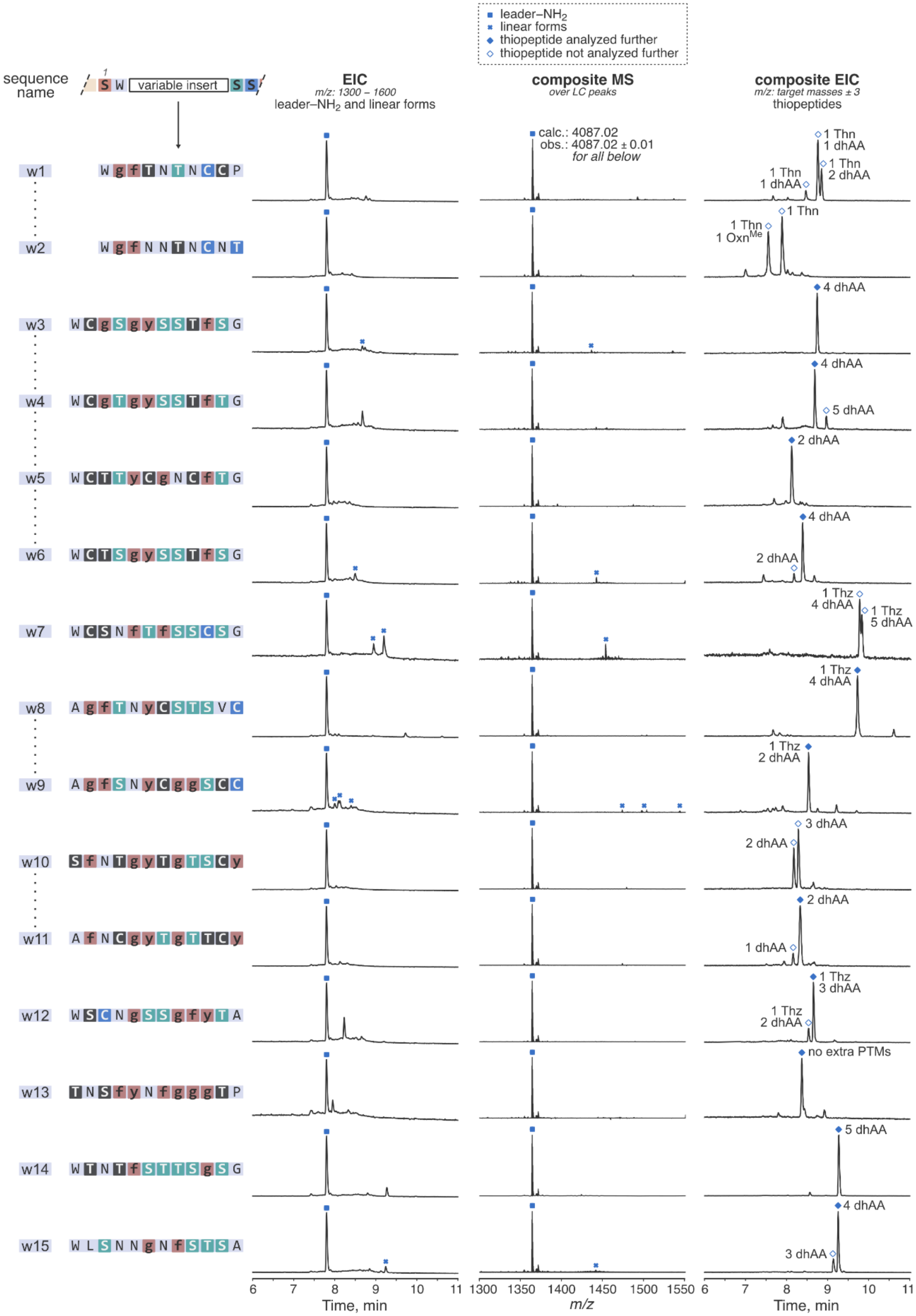
leader–NH2
linear forms
thiopeptide analyzed further
thiopeptide not analyzed further
sequence name
variable insert
EIC
m/z: 1300 – 1600
leader–NH2 and linear forms
composite MS
over LC peaks
composite EIC
m/z: target masses ± 3
thiopeptides
calc.: 4087.02
obs.: 4087.02 ± 0.01
for all below
w1
w2
w3
w4
w5
w6
w7
w8
w9
w10
w11
w12
w13
w14
w15
1 Thn
1 dhAA
2 dhAA
1 OxnMe
4 dhAA
5 dhAA
3 dhAA
1 Thz
no extra PTMs
Time, min
m/z

Figure S17. Maturation of v.t.4w TNIK selection hits using the three-step enzymatic treatment protocol. Precursor peptides *w1–15* expressed with the FIT system as shown in Fig. S15 were treated with Laz enzymes as described in section 2.6; the outcomes were analyzed by LC/MS. Displayed are extracted ion current (EIC) chromatograms (*m/z*: 1300-1600 to visualize the accumulation of linear forms and leader-NH_2); composite MS spectra integrated over substrate-derived peaks (*m/z*: 1300-1550, i. e., the region corresponding to the LC peaks in the chromatograms on the left); and composite EICs for all detected thiopeptide products (scaled Y-axes). In the EIC chromatograms for thiopeptides, thiopeptide-unrelated compounds as well as minor thiopeptides stemming from the misincorporation of proteinogenic amino acids in place of npAAs are unannotated. Clean conversion of precursor peptides into thiopeptides was observed in all cases.

a

b

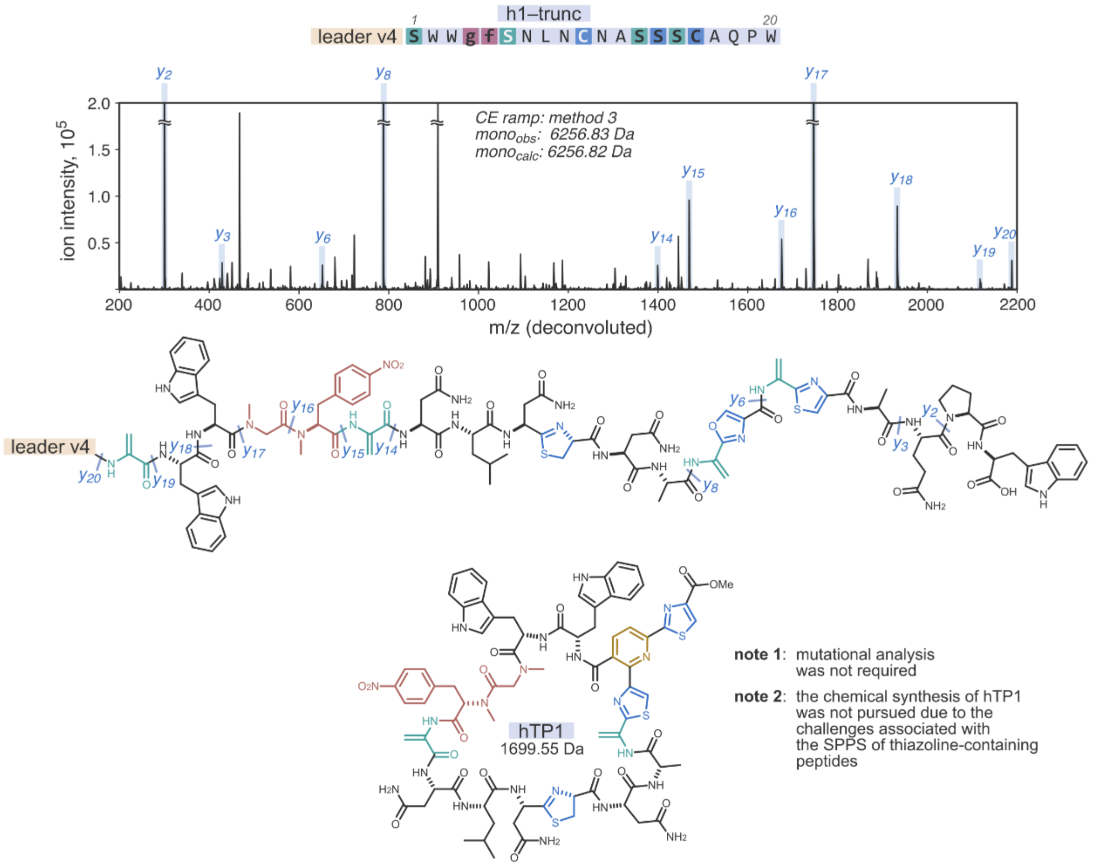

Figure S18. Structure determination studies for thiopeptide hTP1. **a**) MS/MS analysis of the insert modification pattern in C-terminally truncated *h1* precursor peptide. Peptide *h1–trunc* expressed with the FIT system using the reprogrammed genetic code as described and treated with Laz enzymes as described in section 2.6 and 2.7 (LazDEF/LazBF treatment skipping LazC); the major product was analyzed by LC/MS/MS. Shown is a zoomed-in section of a charge-deconvoluted CID fragmentation spectrum for the modified *h1–trunc*; b-ion assignments and neutral molecule losses are omitted for clarity. Fragmentation assignments are mapped onto the suggested chemical structure of the modified *h1–trunc* shown below. **b**) The chemical structure of the target hTP1 thiopeptide. In this case, mutational analysis was not required.

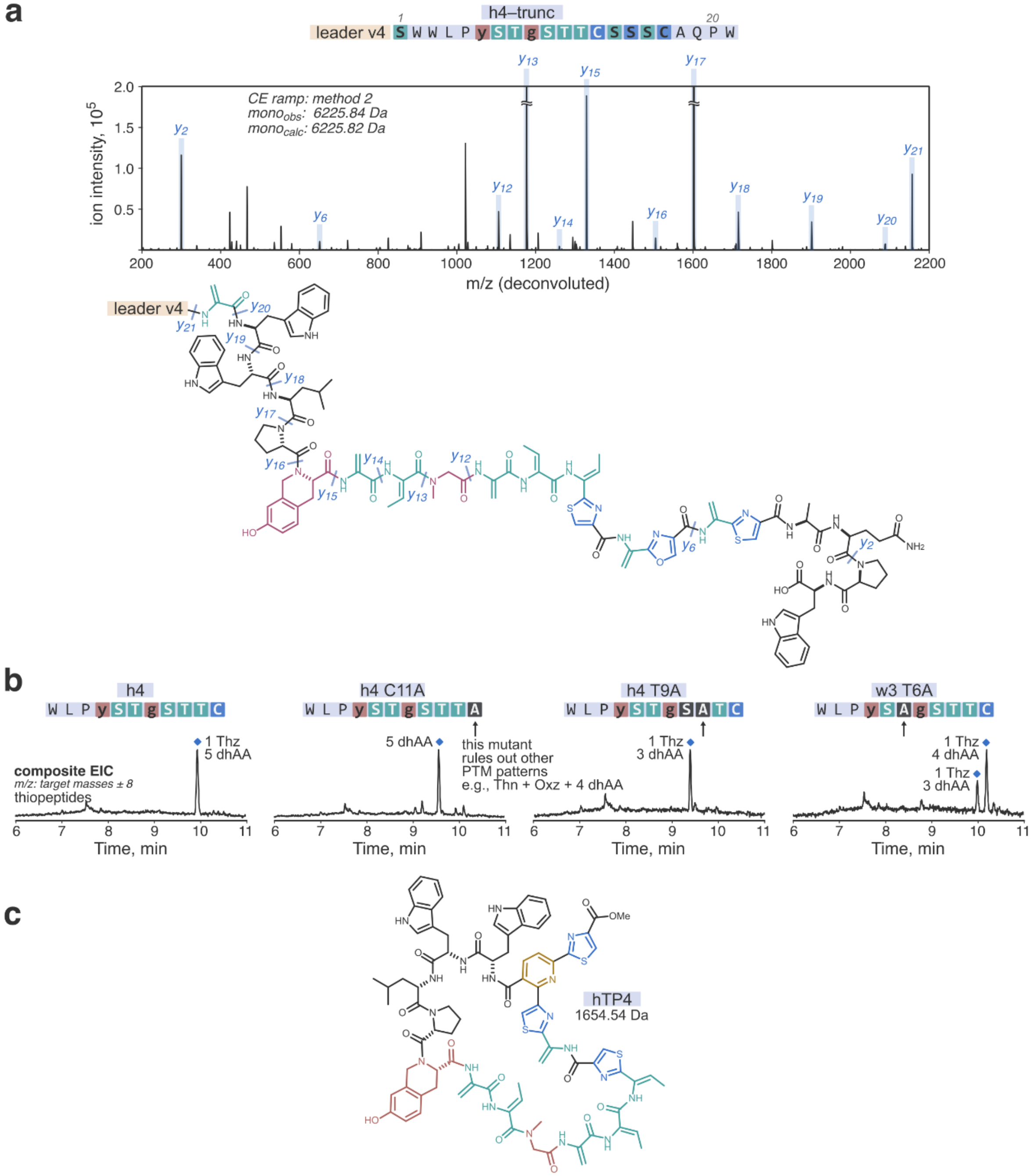

Figure S19. Structure determination studies for thiopeptide hTP4. **a**) MS/MS analysis of the insert modification pattern in C-terminally truncated *h4* precursor peptide. Peptide *h4–trunc* expressed with the FIT system using the reprogrammed genetic code as described and treated with Laz enzymes as described in section 2.6 and 2.7 (LazDEF/LazBF treatment skipping LazC); the major product was analyzed by LC/MS/MS. Shown is a zoomed-in section of a charge-deconvoluted CID fragmentation spectrum for the modified *h4–trunc*; b-ion assignments and neutral molecule losses are omitted for clarity. Fragmentation assignments are mapped onto the suggested chemical structure of the modified *h4–trunc* shown below. **b**) Mutational analysis results. The specified *h4* mutants were

expressed with the FIT system using the reprogrammed genetic code as described and treated with Laz enzymes as described in section 2.6, and the outcomes were analyzed by LC/MS. Displayed are composite extracted ion current chromatograms for all detected thiopeptide products (scaled Y-axes). **c**) The chemical structure of the target hTP4 thiopeptide.

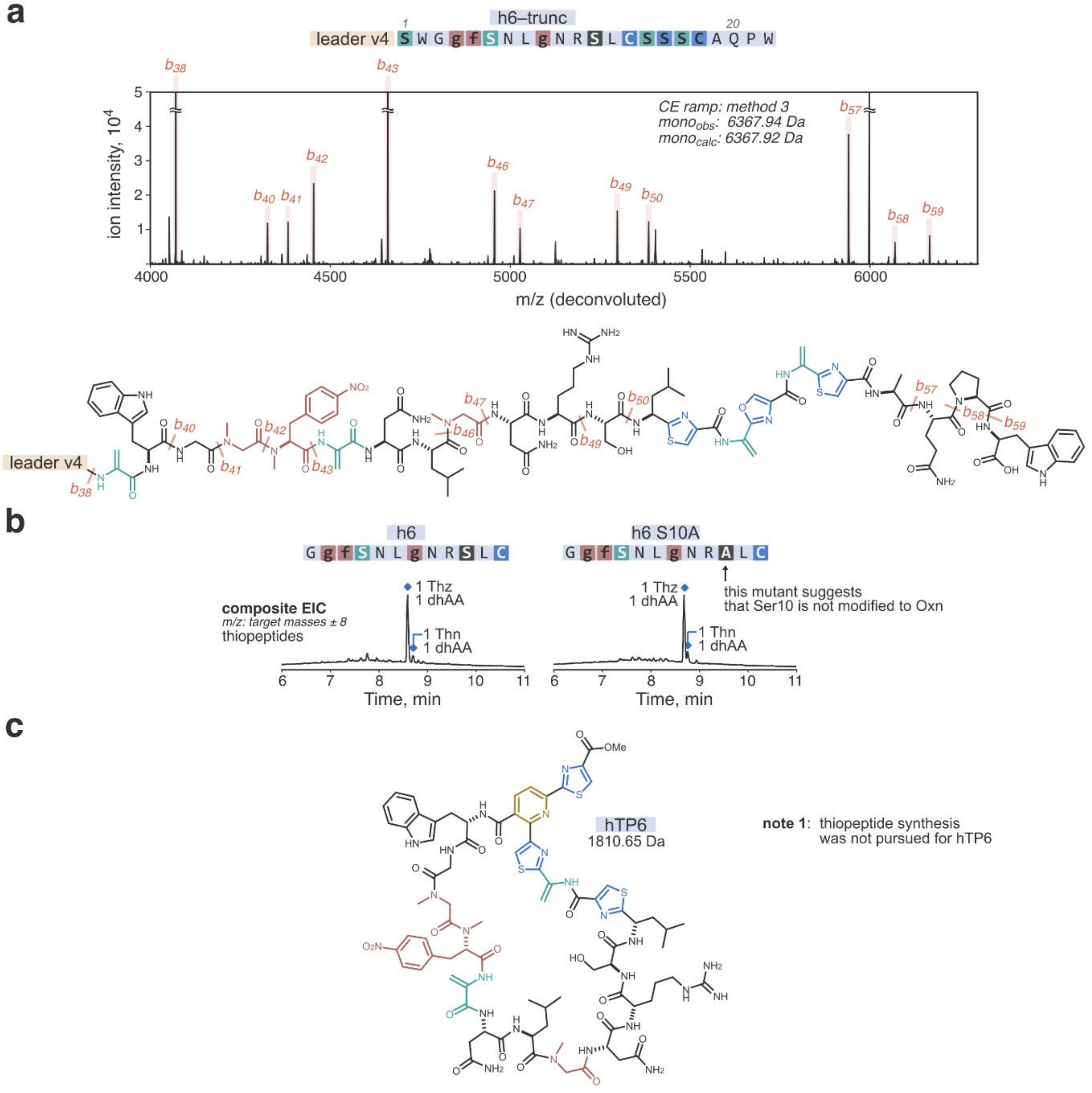

Figure S20. Structure determination studies for thiopeptide hTP6. **a**) MS/MS analysis of the insert modification pattern in C-terminally truncated *h6* precursor peptide. Peptide *h6–trunc* expressed with the FIT system using the reprogrammed genetic code as described and treated with Laz enzymes as described in section 2.6 and 2.7 (LazDEF/LazBF treatment skipping LazC); the major product was analyzed by LC/MS/MS. Shown is a zoomed-in section of a charge-deconvoluted CID fragmentation spectrum for the modified *h6–trunc*; y-ion assignments and neutral molecule losses are omitted for clarity. Fragmentation assignments are mapped onto the suggested chemical structure of the modified *h6–trunc* shown below. **b**) Mutational analysis results. The specified *h6* mutant was expressed with the FIT system using the reprogrammed genetic code as described and treated with Laz enzymes as described in section 2.6, and the outcomes were analyzed by LC/MS. Displayed are composite extracted ion current chromatograms for all detected thiopeptide products (scaled Y-axes). **c**) The chemical structure of the target hTP6 thiopeptide.

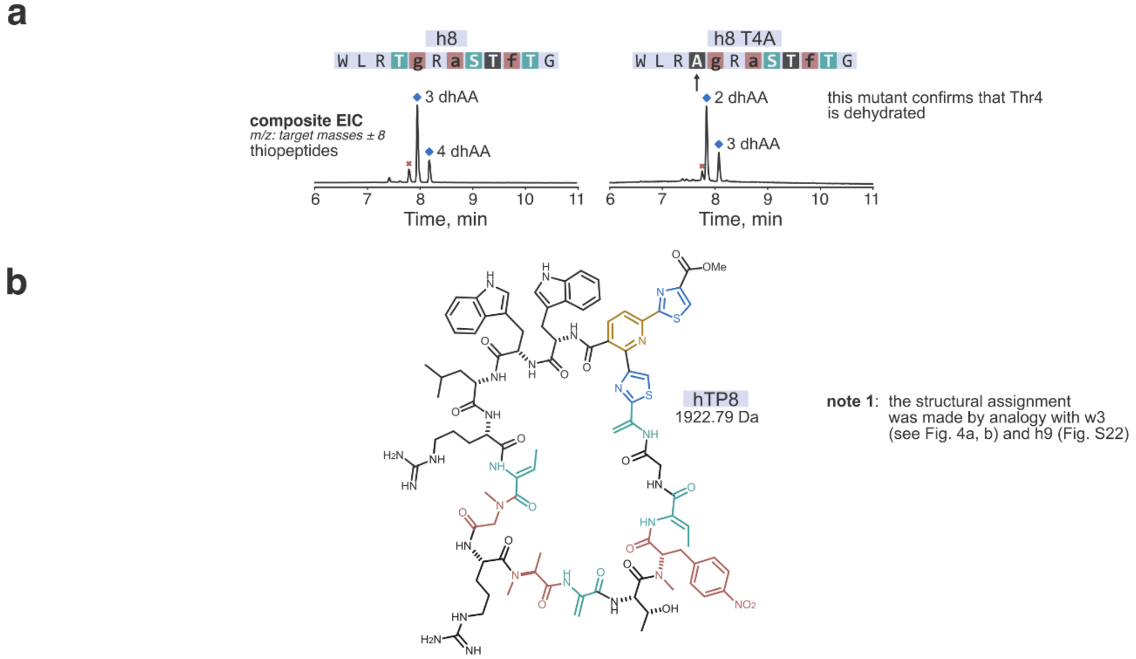

Figure S21. Structure determination studies for thiopeptide hTP8. **a**) Mutational analysis results. The specified *h8* mutant was expressed with the FIT system using the reprogrammed genetic code as described and treated with Laz enzymes as described in section 2.6, and the outcomes were analyzed by LC/MS. Displayed are composite extracted ion current chromatograms for all detected thiopeptide products (scaled Y-axes). Peaks labelled with red crosses are not thiopeptide-derived. **b**) The chemical structure of the target hTP8 thiopeptide. In this case, MS/MS analysis was not required, because the structural assignment was made by analogy to wTP3 and hTP9.

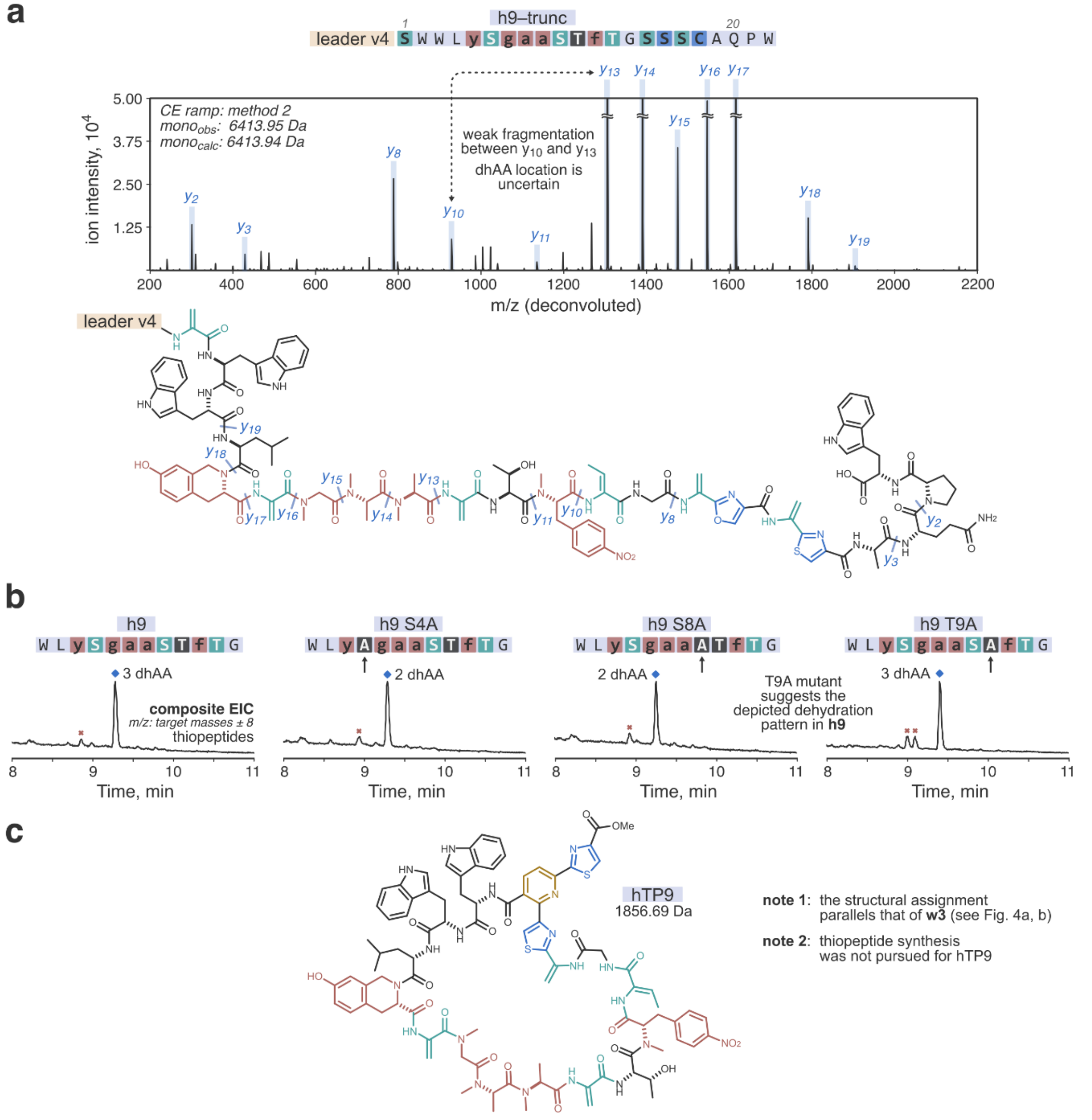

Figure S22. Structure determination studies for thiopeptide hTP9. **a**) MS/MS analysis of the insert modification pattern in C-terminally truncated *h9* precursor peptide. Peptide *h9–trunc* expressed with the FIT system using the reprogrammed genetic code as described and treated with Laz enzymes as described in section 2.6 and 2.7 (LazDEF/LazBF treatment skipping LazC); the major product was analyzed by LC/MS/MS. Shown is a zoomed-in section of a charge-deconvoluted CID fragmentation spectrum for the modified *h9–trunc*; y-ion assignments and neutral molecule losses are omitted for clarity. Fragmentation assignments are mapped onto the suggested chemical structure of the modified *h9–trunc* shown below. **b**) Mutational analysis results. The specified *h9* mutants were expressed with the FIT system using the reprogrammed genetic code as described and treated with Laz enzymes as described in section 2.6, and the outcomes were analyzed by LC/MS. Displayed are composite extracted ion current chromatograms for all detected thiopeptide products (scaled Y-axes). **c**) The chemical structure of the target hTP9 thiopeptide.

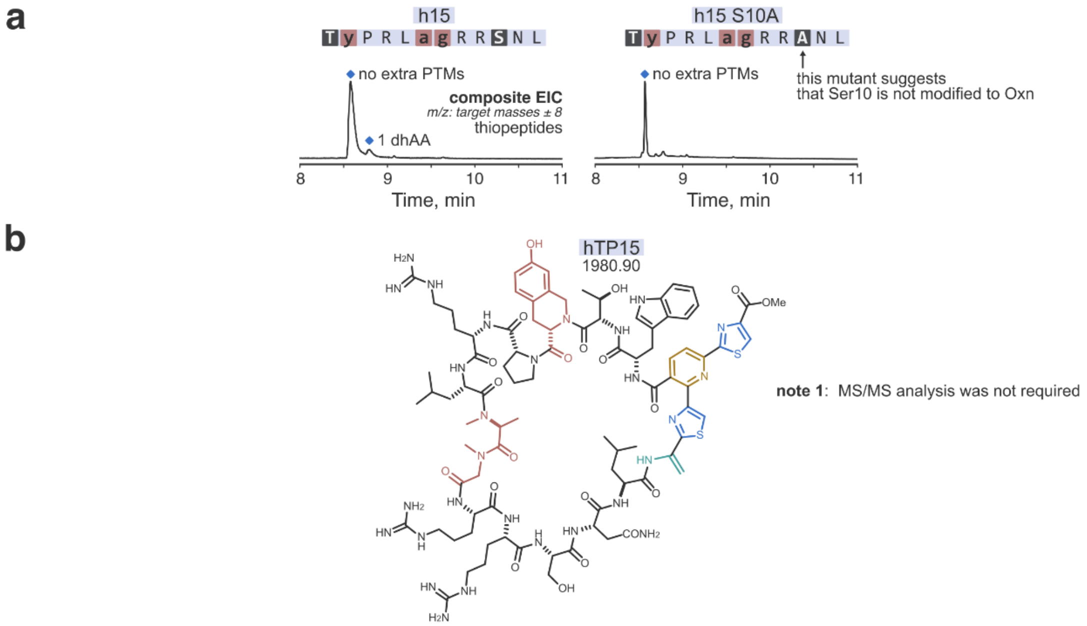

Figure S23. Structure determination studies for thiopeptide hTP15. **a**) Mutational analysis results. The specified *h15* mutant was expressed with the FIT system using the reprogrammed genetic code as described and treated with Laz enzymes as described in section 2.6, and the outcomes were analyzed by LC/MS. Displayed are composite extracted ion current chromatograms for all detected thiopeptide products (scaled Y-axes). **c**) The chemical structure of the target hTP15 thiopeptide. In this case, MS/MS analysis was not required.

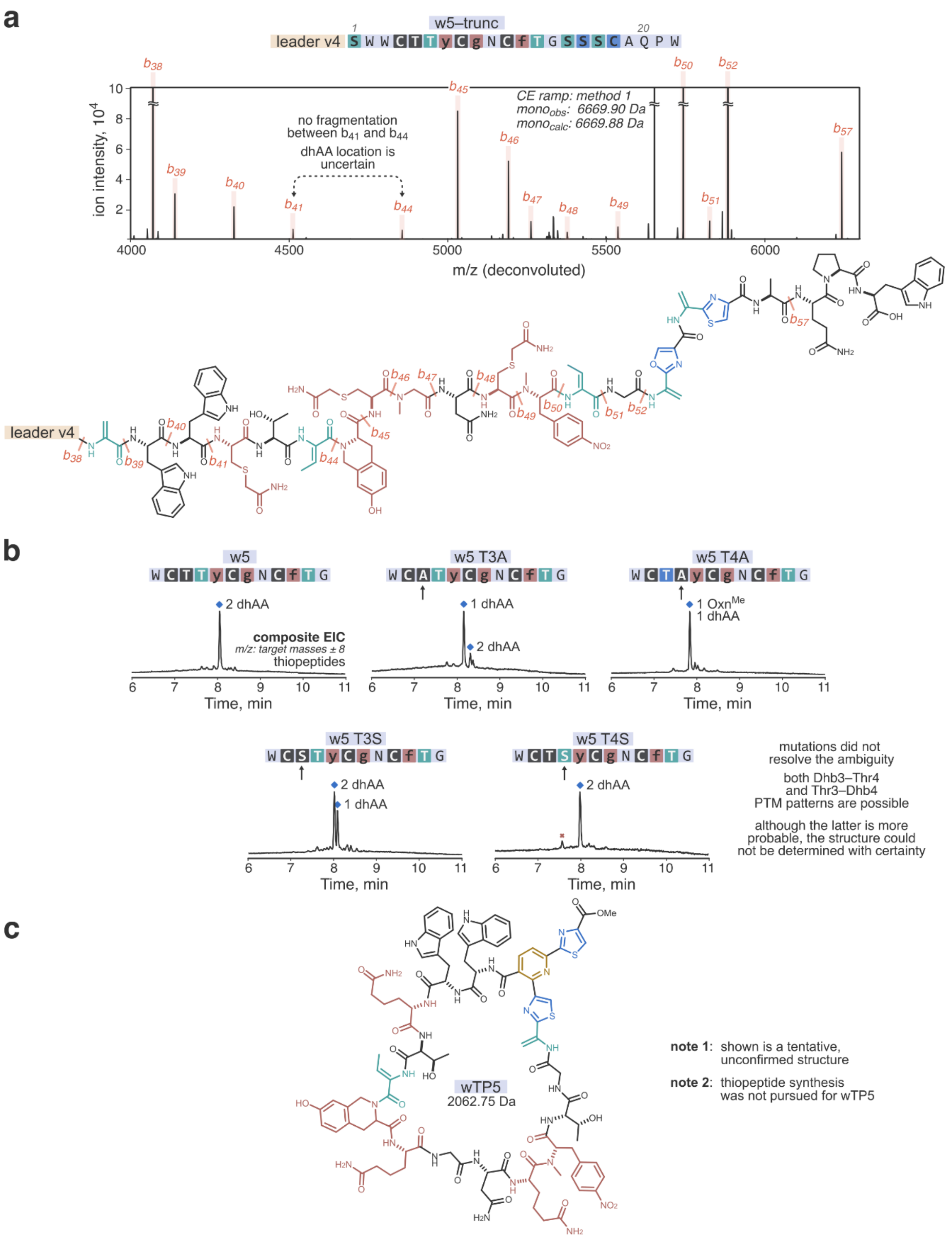

Figure S24. Structure determination studies for thiopeptide wTP5. **a**) MS/MS analysis of the insert modification pattern in C-terminally truncated *w5* precursor peptide. Peptide *w5–trunc* expressed with

the FIT system using the reprogrammed genetic code as described and treated with Laz enzymes as described in section 2.6 and 2.7 (LazDEF/LazBF treatment skipping LazC); the major product was analyzed by LC/MS/MS. Shown is a zoomed-in section of a charge-deconvoluted CID fragmentation spectrum for the modified *w5–trunc*; y-ion assignments and neutral molecule losses are omitted for clarity. Fragmentation assignments are mapped onto the suggested chemical structure of the modified *w5–trunc* shown below. **b**) Mutational analysis results. The specified *w5* mutants were expressed with the FIT system using the reprogrammed genetic code as described and treated with Laz enzymes as described in section 2.6, and the outcomes were analyzed by LC/MS. Displayed are composite extracted ion current chromatograms for all detected thiopeptide products (scaled Y-axes). **c**) The tentative chemical structure of the target wTP5 thiopeptide. In this case, neither MS/MS nor mutational analyses could unambiguously determine the structure.

a

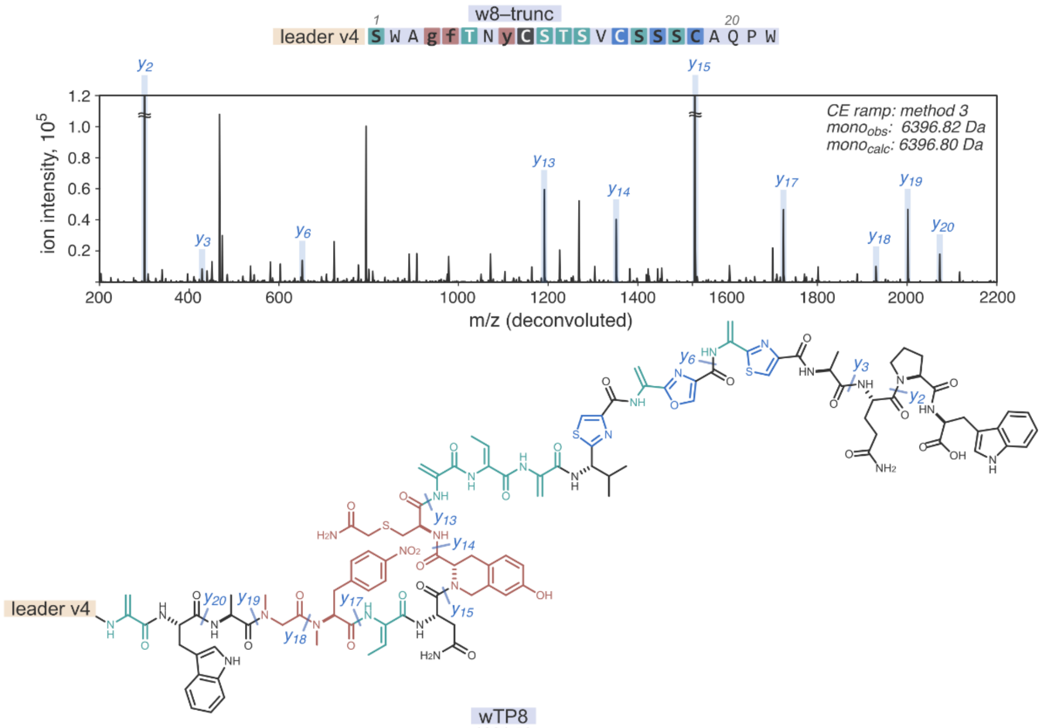

b

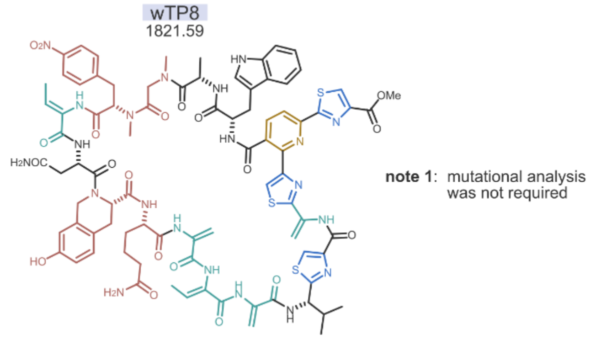

Figure S25. Structure determination studies for thiopeptide wTP8. **a**) MS/MS analysis of the insert modification pattern in C-terminally truncated *w8* precursor peptide. Peptide *w8–trunc* expressed with the FIT system using the reprogrammed genetic code as described and treated with Laz enzymes as described in section 2.6 and 2.7 (LazDEF/LazBF treatment skipping LazC); the major product was analyzed by LC/MS/MS. Shown is a zoomed-in section of a charge-deconvoluted CID fragmentation spectrum for the modified *w8–trunc*; b-ion assignments and neutral molecule losses are omitted for clarity. Fragmentation assignments are mapped onto the suggested chemical structure of the modified *w8–trunc* shown below. **b**) The chemical structure of the target wTP8 thiopeptide. In this case, mutational analysis was not required.

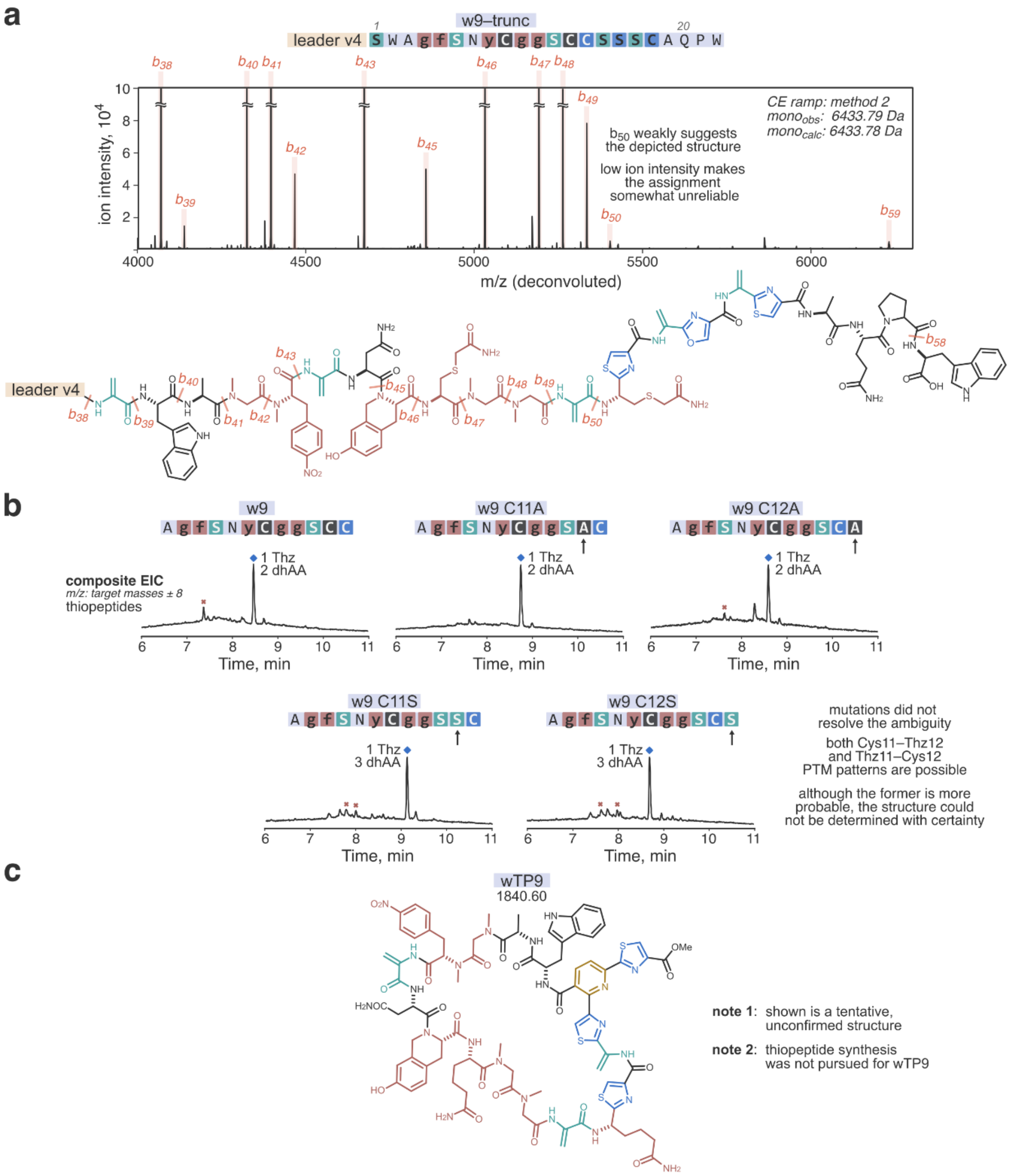

Figure S26. Structure determination studies for thiopeptide wTP9. **a**) MS/MS analysis of the insert modification pattern in C-terminally truncated *w9* precursor peptide. Peptide *w9–trunc* expressed with the FIT system using the reprogrammed genetic code as described and treated with Laz enzymes as described in section 2.6 and 2.7 (LazDEF/LazBF treatment skipping LazC); the major product was analyzed by LC/MS/MS. Shown is a zoomed-in section of a charge-deconvoluted CID fragmentation spectrum for the modified *w9–trunc*; y-ion assignments and neutral molecule losses are omitted for clarity. Fragmentation assignments are mapped onto the suggested chemical structure of the

modified *w9–trunc* shown below. **b**) Mutational analysis results. The specified *w9* mutants were expressed with the FIT system using the reprogrammed genetic code as described and treated with Laz enzymes as described in section 2.6, and the outcomes were analyzed by LC/MS. Displayed are composite extracted ion current chromatograms for all detected thiopeptide products (scaled Y-axes). **c**) The tentative chemical structure of the target wTP9 thiopeptide. In this case, neither MS/MS nor mutational analyses could unambiguously determine the structure.

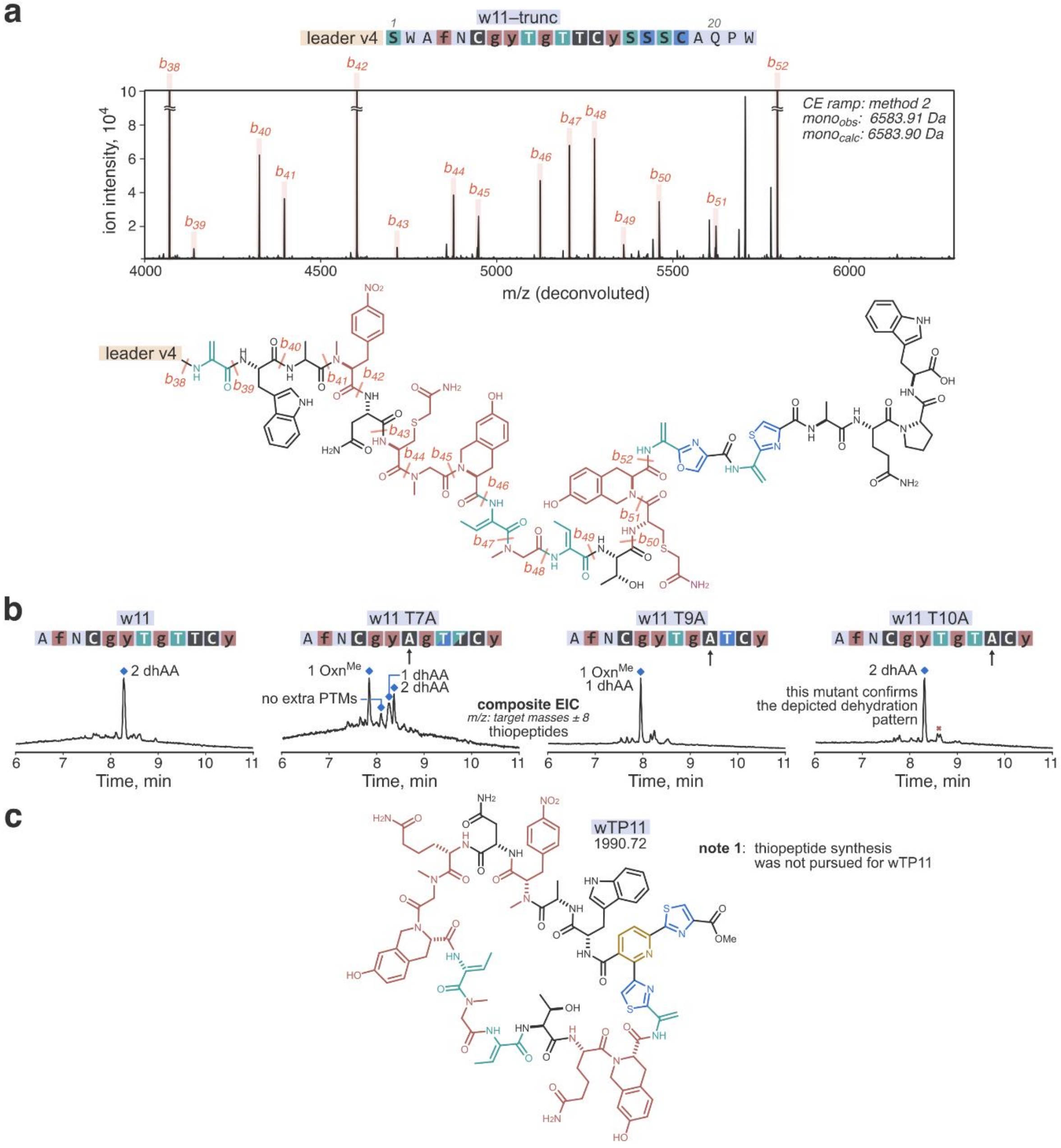

Figure S27. Structure determination studies for thiopeptide wTP11. **a**) MS/MS analysis of the insert modification pattern in C-terminally truncated *w11* precursor peptide. Peptide *w11–trunc* expressed with the FIT system using the reprogrammed genetic code as described and treated with Laz enzymes as described in section 2.6 and 2.7 (LazDEF/LazBF treatment skipping LazC); the major product was analyzed by LC/MS/MS. Shown is a zoomed-in section of a charge-deconvoluted CID fragmentation spectrum for the modified *w11–trunc*; y-ion assignments and neutral molecule losses are omitted for clarity. Fragmentation assignments are mapped onto the suggested chemical structure of the modified *w11–trunc* shown below. **b**) Mutational analysis results. The specified *w11* mutants were expressed with the FIT system using the reprogrammed genetic code as described and treated with Laz enzymes as described in section 2.6, and the outcomes were analyzed by LC/MS. Displayed

are composite extracted ion current chromatograms for all detected thiopeptide products (scaled Y-axes). **c**) The chemical structure of the target wTP11 thiopeptide.

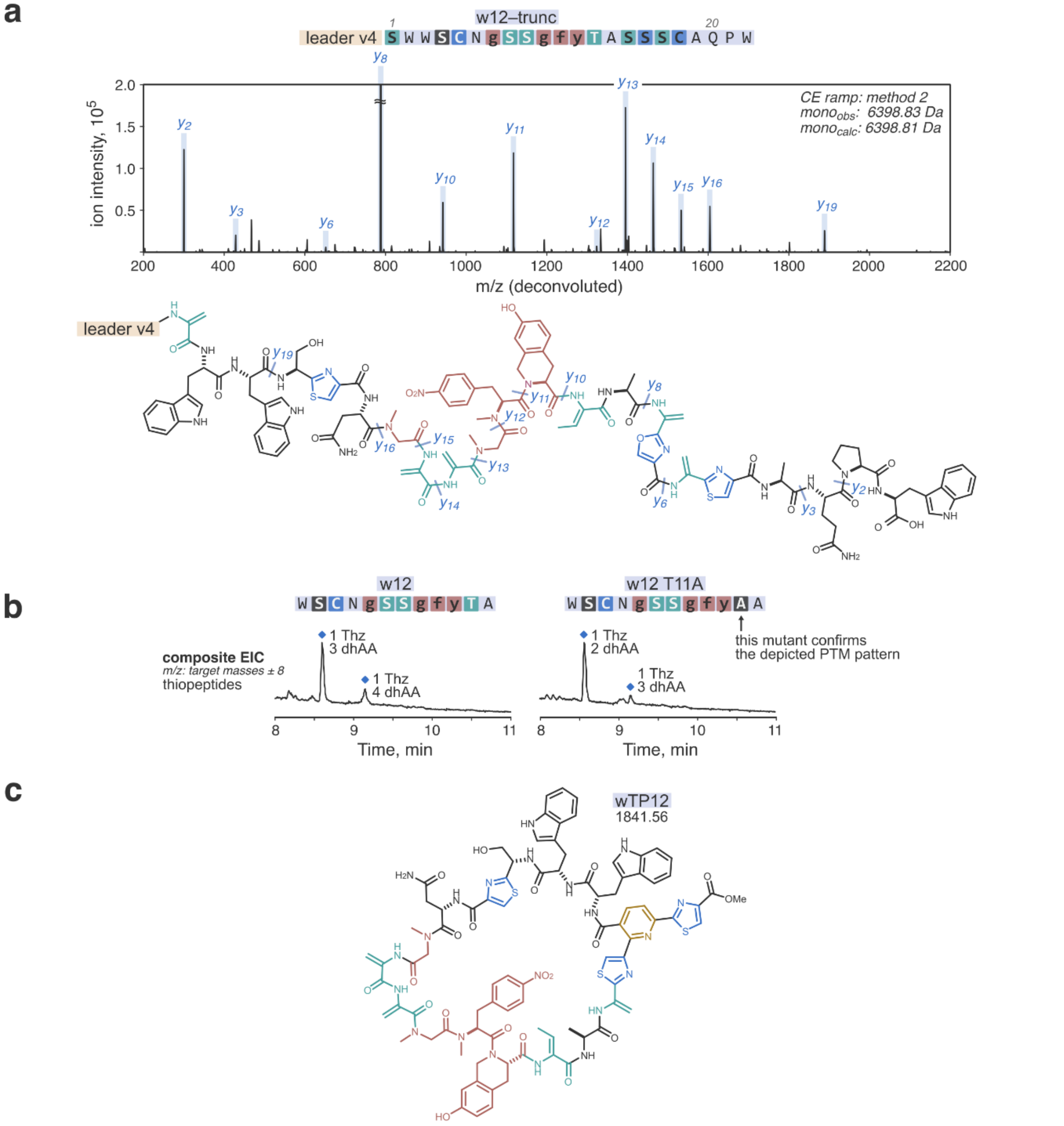

Figure S28. Structure determination studies for thiopeptide wTP12. **a**) MS/MS analysis of the insert modification pattern in C-terminally truncated *w12* precursor peptide. Peptide *w12–trunc* expressed with the FIT system using the reprogrammed genetic code as described and treated with Laz enzymes as described in section 2.6 and 2.7 (LazDEF/LazBF treatment skipping LazC); the major product was analyzed by LC/MS/MS. Shown is a zoomed-in section of a charge-deconvoluted CID fragmentation spectrum for the modified *w12–trunc*; y-ion assignments and neutral molecule losses are omitted for clarity. Fragmentation assignments are mapped onto the suggested chemical structure of the modified *w12–trunc* shown below. **b**) Mutational analysis results. The specified *w12* mutant was expressed with the FIT system using the reprogrammed genetic code as described and treated with Laz enzymes as described in section 2.6, and the outcomes were analyzed by LC/MS. Displayed

are composite extracted ion current chromatograms for all detected thiopeptide products (scaled Y-axes). **c**) The tentative chemical structure of the target wTP12 thiopeptide.

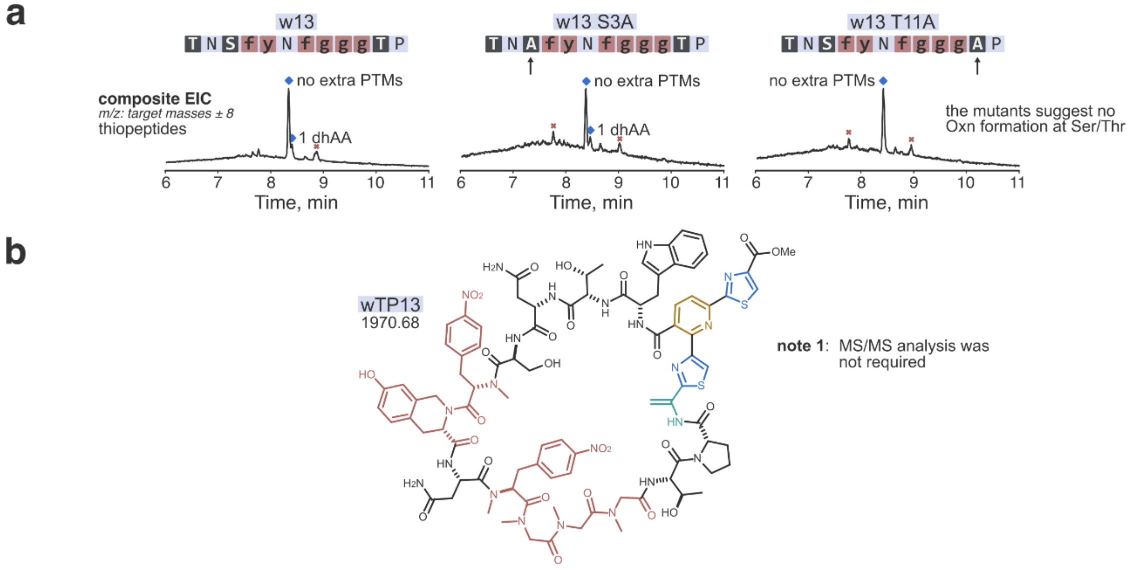

Figure S29. Structure determination studies for thiopeptide wTP13. **a**) Mutational analysis results. The specified *w13* mutants were expressed with the FIT system using the reprogrammed genetic code as described and treated with Laz enzymes as described in section 2.6, and the outcomes were analyzed by LC/MS. Displayed are composite extracted ion current chromatograms for all detected thiopeptide products (scaled Y-axes). **b**) The chemical structure of the target wTP13 thiopeptide. In this case, MS/MS analysis was not required.

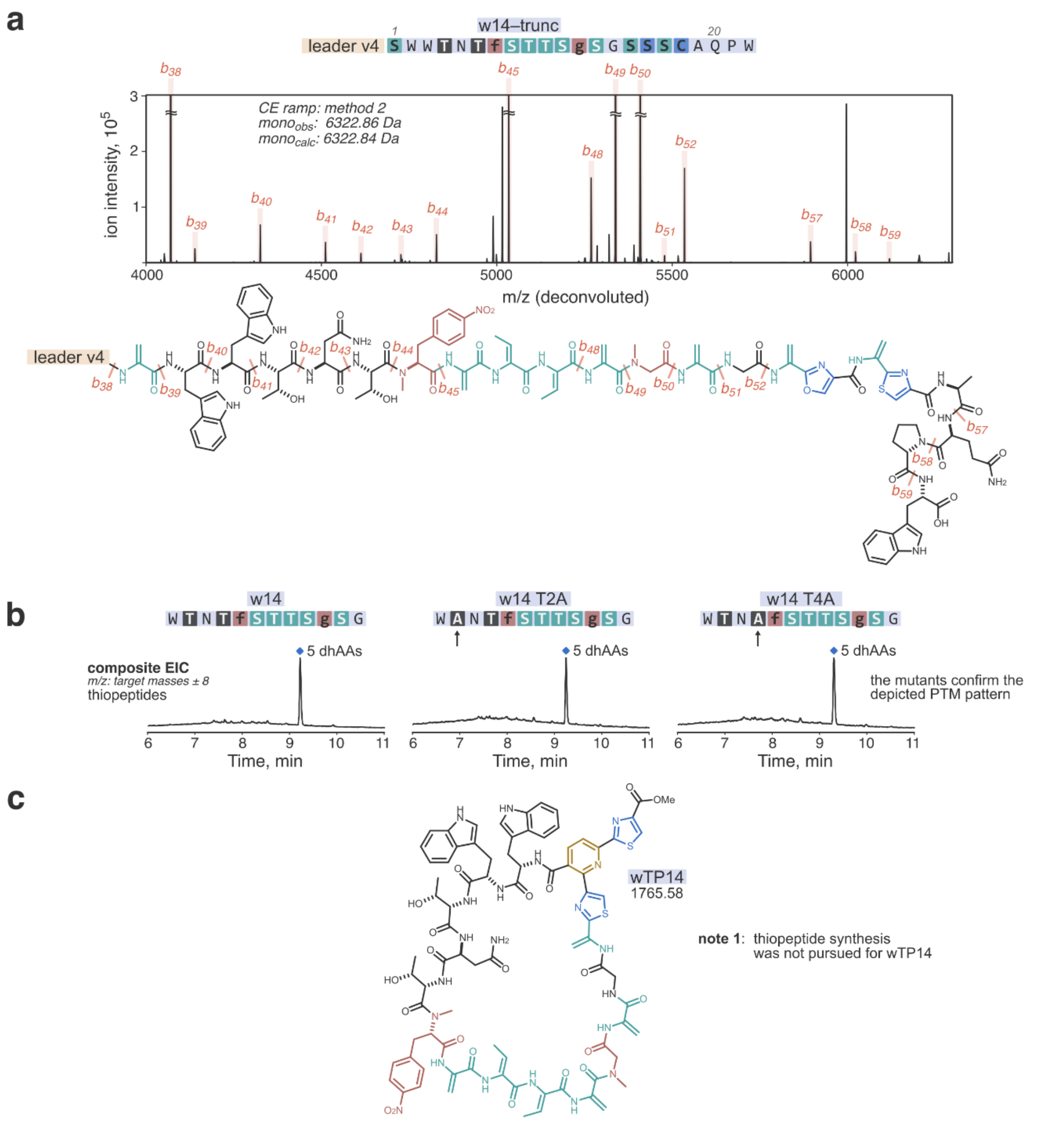

Figure S30. Structure determination studies for thiopeptide wTP14. **a**) MS/MS analysis of the insert modification pattern in C-terminally truncated *w14* precursor peptide. Peptide *w14–trunc* expressed with the FIT system using the reprogrammed genetic code as described and treated with Laz enzymes as described in section 2.6 and 2.7 (LazDEF/LazBF treatment skipping LazC); the major product was analyzed by LC/MS/MS. Shown is a zoomed-in section of a charge-deconvoluted CID fragmentation spectrum for the modified *w14–trunc*; y-ion assignments and neutral molecule losses are omitted for clarity. Fragmentation assignments are mapped onto the suggested chemical structure of the modified *w14–trunc* shown below. **b**) Mutational analysis results. The specified *w14* mutants were expressed with the FIT system using the reprogrammed genetic code as described and treated with Laz enzymes as described in section 2.6, and the outcomes were analyzed by LC/MS. Displayed

are composite extracted ion current chromatograms for all detected thiopeptide products (scaled Y-axes). **c**) The chemical structure of the target wTP14 thiopeptide.

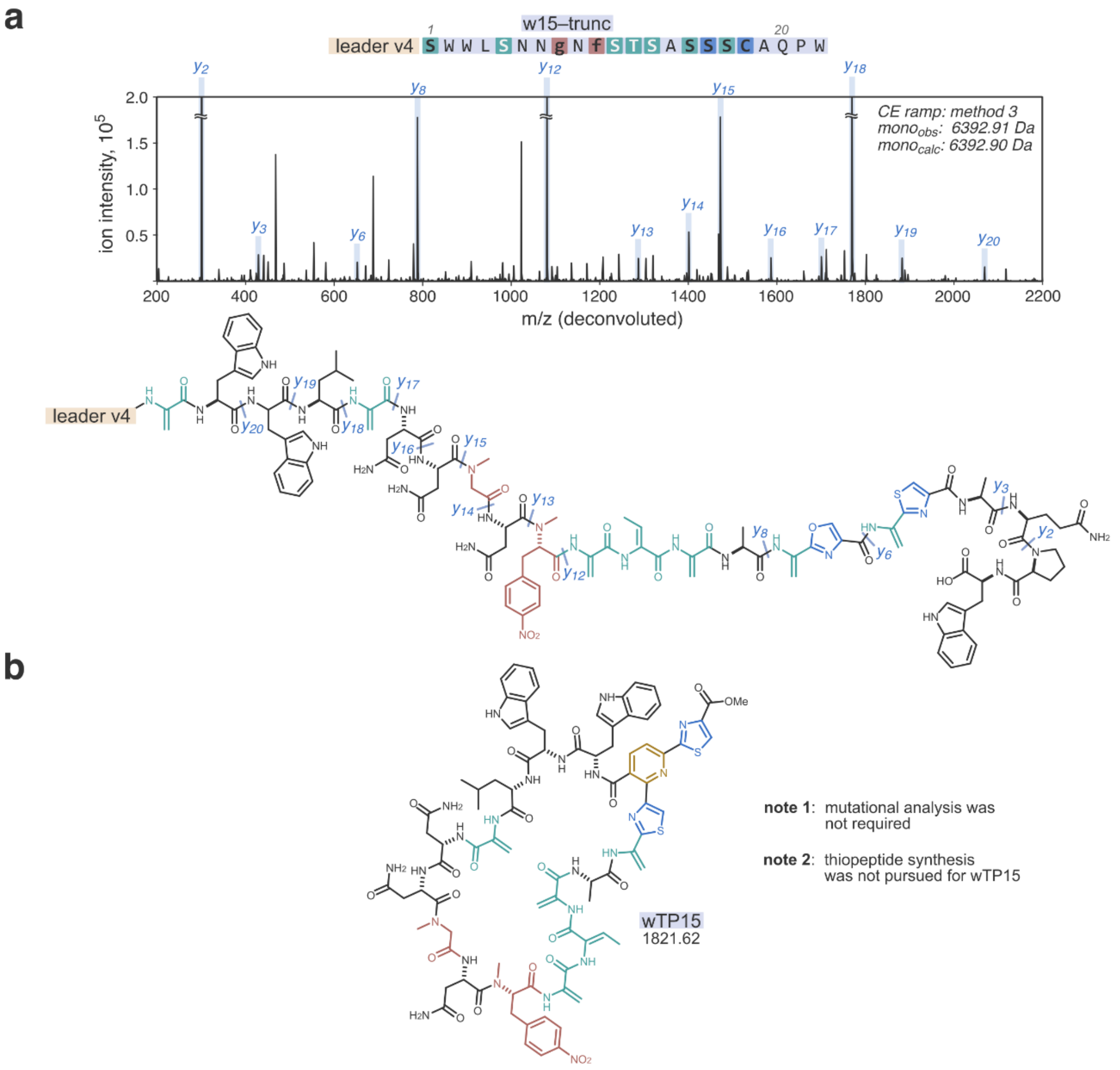

Figure S31. Structure determination studies for thiopeptide wTP15. **a**) MS/MS analysis of the insert modification pattern in C-terminally truncated *w15* precursor peptide. Peptide *w15–trunc* expressed with the FIT system using the reprogrammed genetic code as described and treated with Laz enzymes as described in section 2.6 and 2.7 (LazDEF/LazBF treatment skipping LazC); the major product was analyzed by LC/MS/MS. Shown is a zoomed-in section of a charge-deconvoluted CID fragmentation spectrum for the modified *w15–trunc*; y-ion assignments and neutral molecule losses are omitted for clarity. Fragmentation assignments are mapped onto the suggested chemical structure of the modified *w15–trunc* shown below. **b**) The chemical structure of the target wTP15 thiopeptide. In this case, mutational analysis was not required.

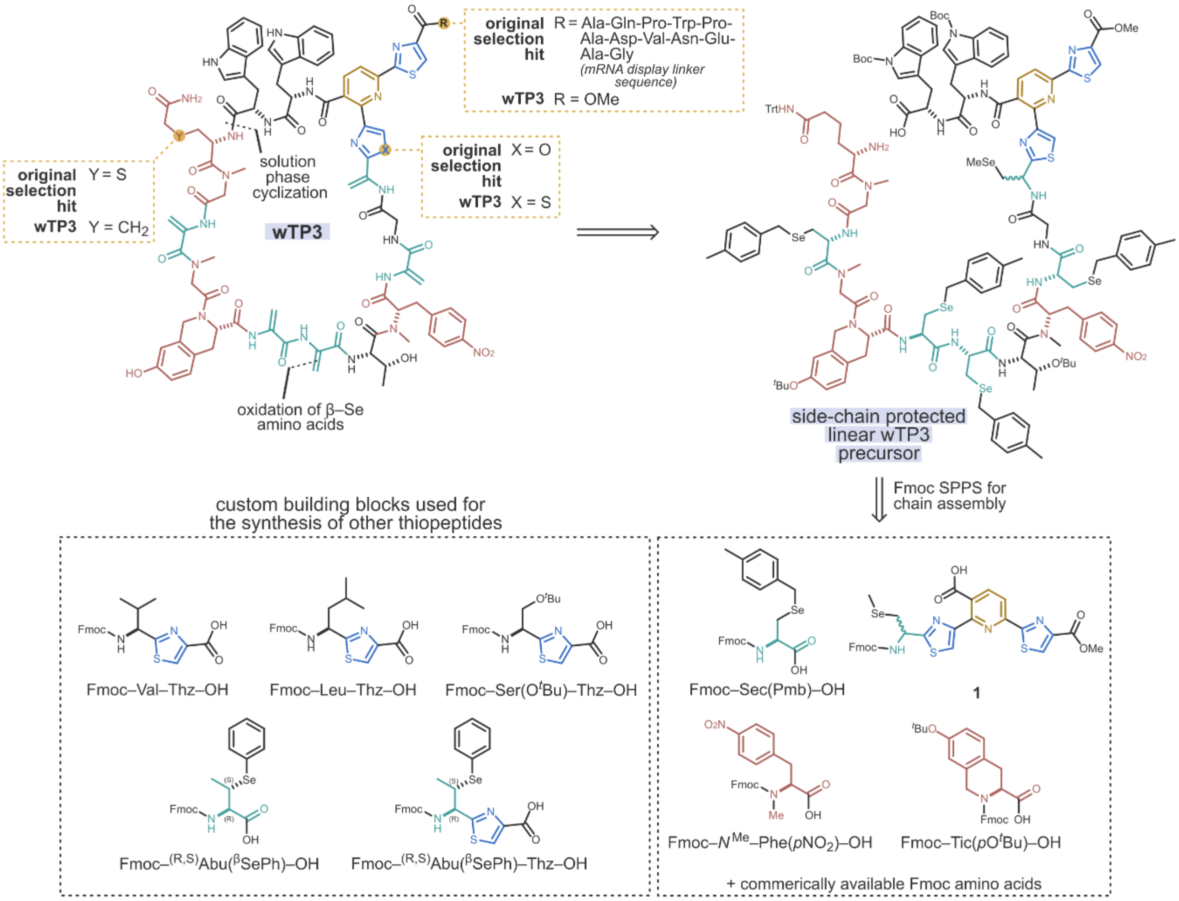

Figure S32. Chemical structure of wTP3 and its retrosynthetic analysis. The differences between the original selection hit and the synthesized wTP3 structures are highlighted. The details of the custom building block and thiopeptide syntheses are provided in sections 4 and 5, respectively.

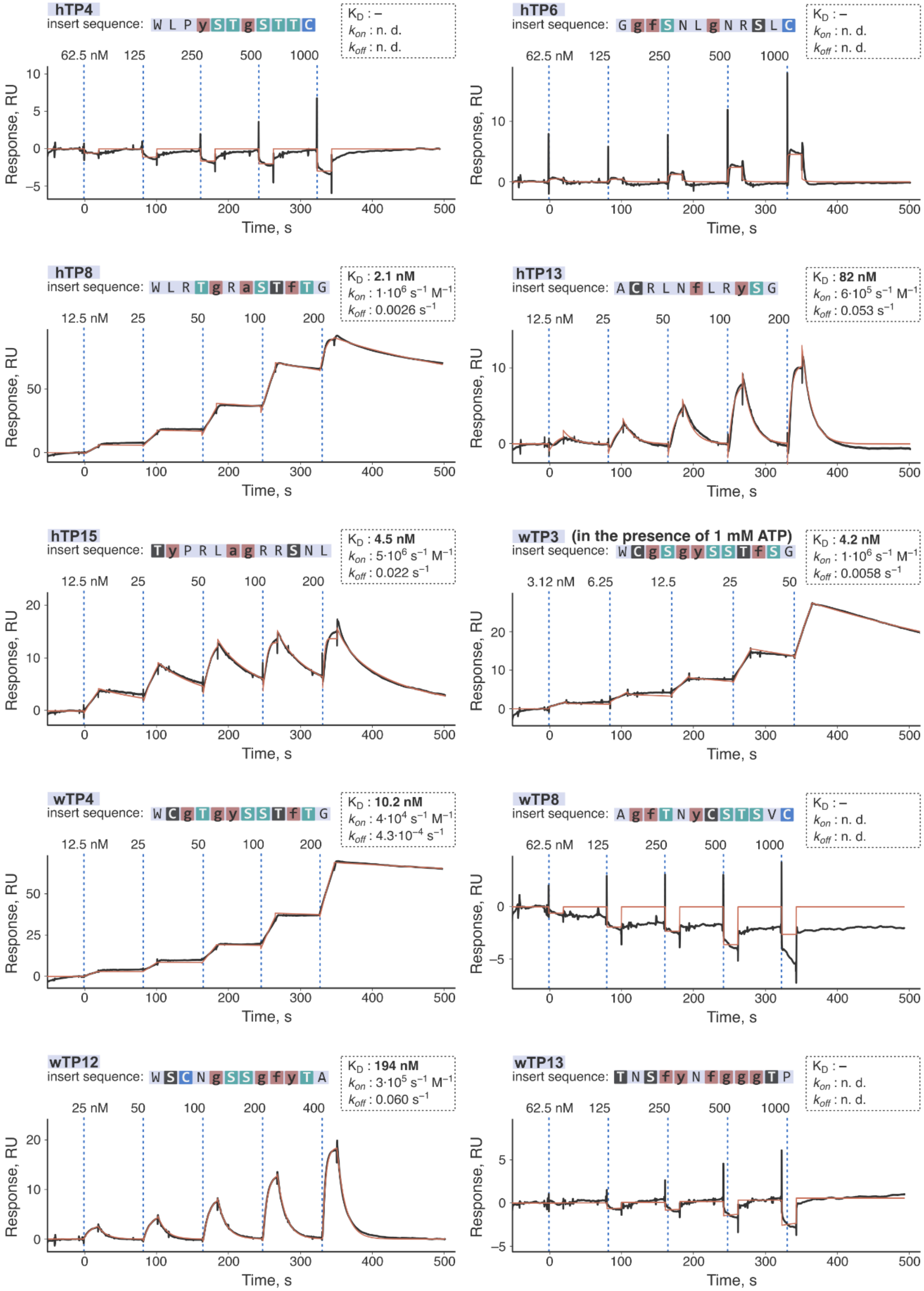
hTP4
insert sequence: W L P y S T g S T T C
K_D : –
k_{on} : n. d.
k_{off} : n. d.
62.5 nM 125 250 500 1000
Response, RU
Time, s
hTP6
insert sequence: G g f S N L g N R S L C
K_D : –
k_{on} : n. d.
k_{off} : n. d.
62.5 nM 125 250 500 1000
Response, RU
Time, s
hTP8
insert sequence: W L R T g R a S T f T G
K_D : 2.1 nM
k_{on} : $1 \cdot 10^6$ s^{-1} M^{-1}
k_{off} : 0.0026 s^{-1}
12.5 nM 25 50 100 200
Response, RU
Time, s
hTP13
insert sequence: A C R L N f L R y S G
K_D : 82 nM
k_{on} : $6 \cdot 10^5$ s^{-1} M^{-1}
k_{off} : 0.053 s^{-1}
12.5 nM 25 50 100 200
Response, RU
Time, s
hTP15
insert sequence: T y P R L a g R R S N L
K_D : 4.5 nM
k_{on} : $5 \cdot 10^6$ s^{-1} M^{-1}
k_{off} : 0.022 s^{-1}
12.5 nM 25 50 100 200
Response, RU
Time, s
wTP3 (in the presence of 1 mM ATP)
insert sequence: W C g S g y S S T f S G
K_D : 4.2 nM
k_{on} : $1 \cdot 10^6$ s^{-1} M^{-1}
k_{off} : 0.0058 s^{-1}
3.12 nM 6.25 12.5 25 50
Response, RU
Time, s
wTP4
insert sequence: W C g T g y S S T f T G
K_D : 10.2 nM
k_{on} : $4 \cdot 10^4$ s^{-1} M^{-1}
k_{off} : $4.3 \cdot 10^{-4}$ s^{-1}
12.5 nM 25 50 100 200
Response, RU
Time, s
wTP8
insert sequence: A g f T N y C S T S V C
K_D : –
k_{on} : n. d.
k_{off} : n. d.
62.5 nM 125 250 500 1000
Response, RU
Time, s
wTP12
insert sequence: W S C N g S S g f y T A
K_D : 194 nM
k_{on} : $3 \cdot 10^5$ s^{-1} M^{-1}
k_{off} : 0.060 s^{-1}
25 nM 50 100 200 400
Response, RU
Time, s
wTP13
insert sequence: T N S f y N f g g g T P
K_D : –
k_{on} : n. d.
k_{off} : n. d.
62.5 nM 125 250 500 1000
Response, RU
Time, s

Figure S33. Surface plasmon resonance sensorgram curves (single cycle kinetics) for the synthesized thiopeptides. See section 2.8 for experimental details. Experimental data are shown as black lines, and best fit in orange. Insert sequence color coding is as in Fig. 3.

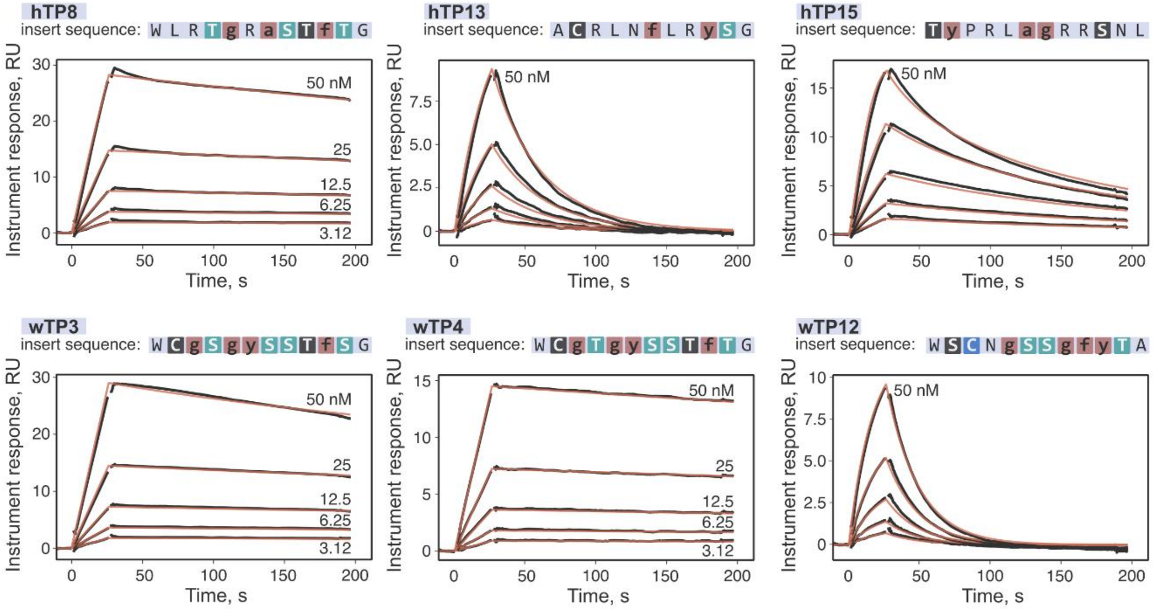

Figure S34. Representative surface plasmon resonance sensorgram curves (multi cycle kinetics) for six TNIK ligands with nM affinities. See section 2.8 for experimental details. Experimental data are shown as black lines, and best fit in orange. Insert sequence color coding is as in Fig. 3. Kinetic parameters determined from these experiments are summarized in Table S5.

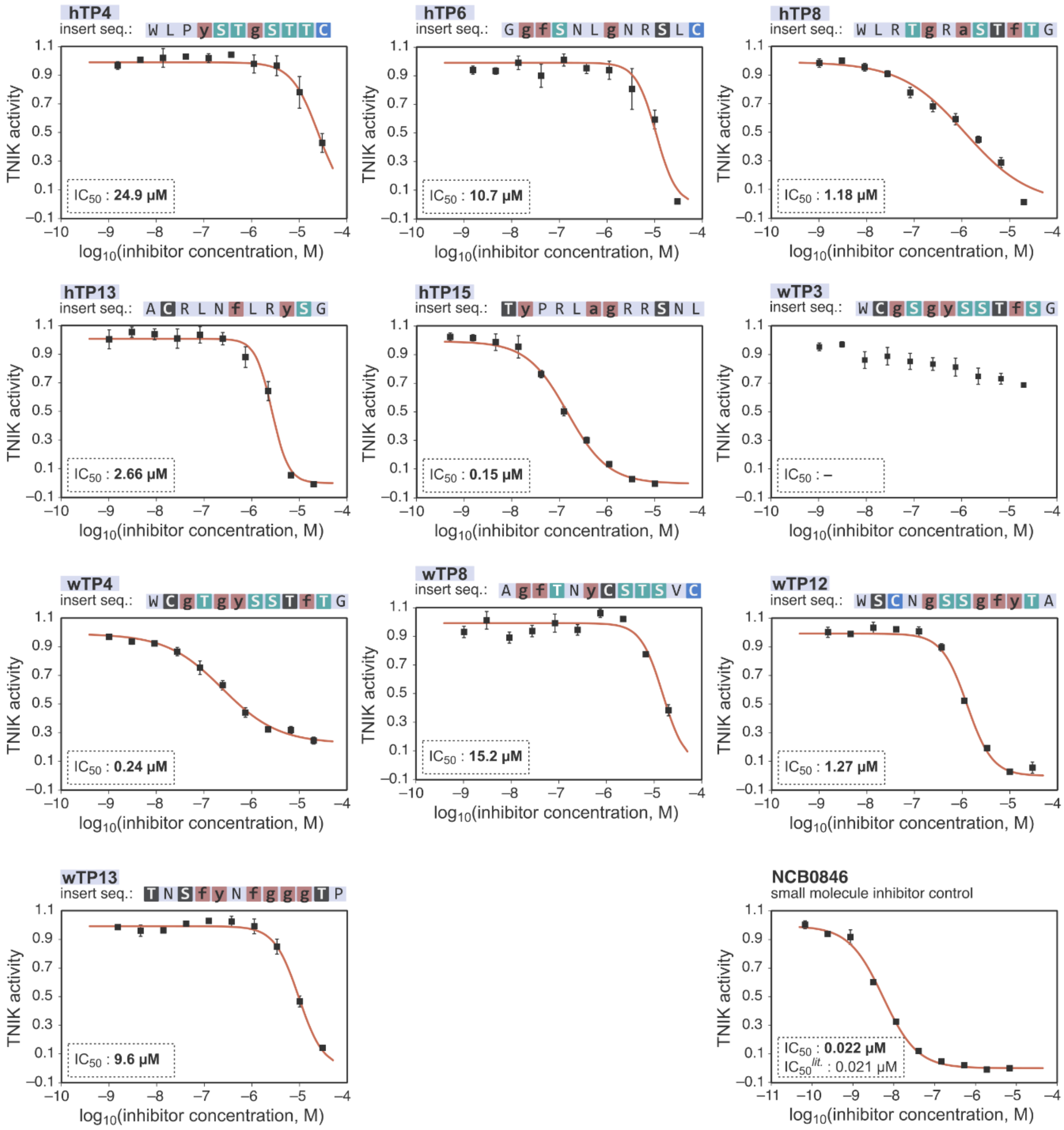

Figure S35. TNIK inhibition curves for the synthesized compounds and NCB0846 small molecule inhibitor control.[27] See section 2.9 for experimental details. Experimental data are shown in black, and best fit in orange. Insert sequence color coding is as in Fig. 3.

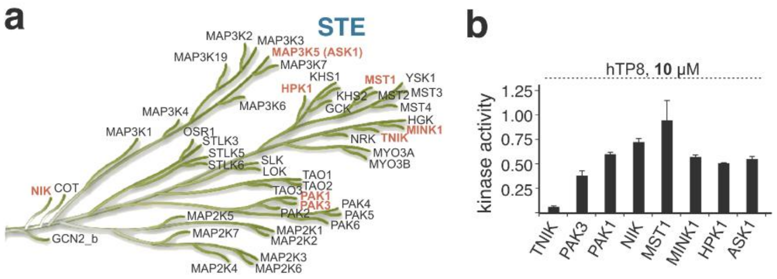

Figure S36. Kinase selectivity profiling outcomes. **a**) The phylogenetic tree showing the relationship between the Ste-20 family kinases. The assayed proteins are indicated in orange. Illustration reproduced courtesy of Cell Signaling Technology, Inc. (www.cellsignal.com). **b**) Kinase selectivity profiling outcomes for hTP8, tested at 10 μM thiopeptide concentration.

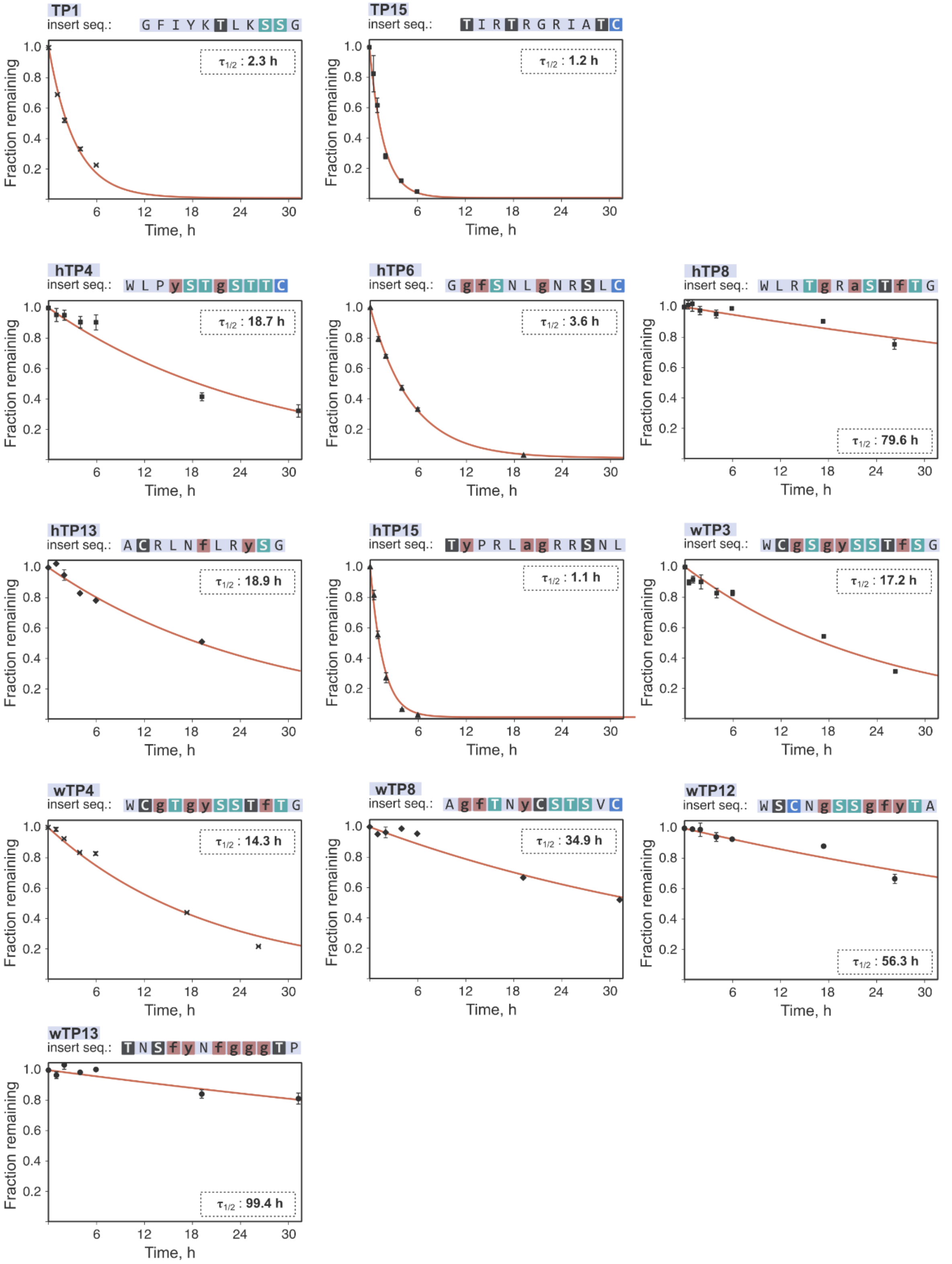

Figure S37. Decomposition of the synthesized compounds and previously reported TP1/TP15[12] in

human serum. See section 2.10 for experimental details. Experimental data are shown in black, and best fit in orange. Insert sequence color coding is as in Fig. 3.

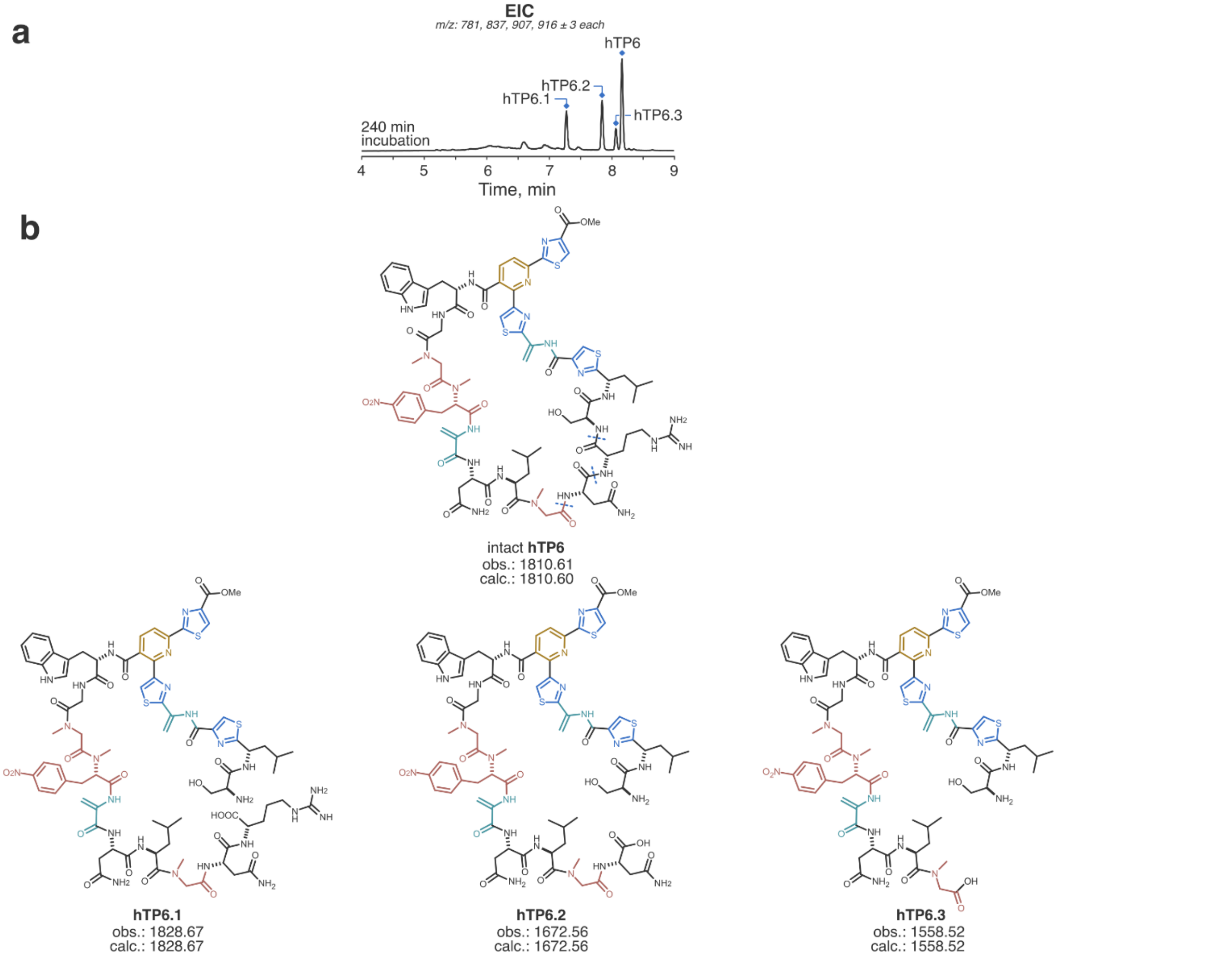

Figure S38. Analysis of hTP6 degradation products in human serum. The compound was incubated in human serum and at various timepoints the results were analyzed by LC/MS. See section 2.10 for experimental details. **a**) Composite EICs for intact hTP6 and some of its degradation products detected after a 240 min incubation with human serum. **b**) Assigned chemical structures of the degradation products indicated in panel a).

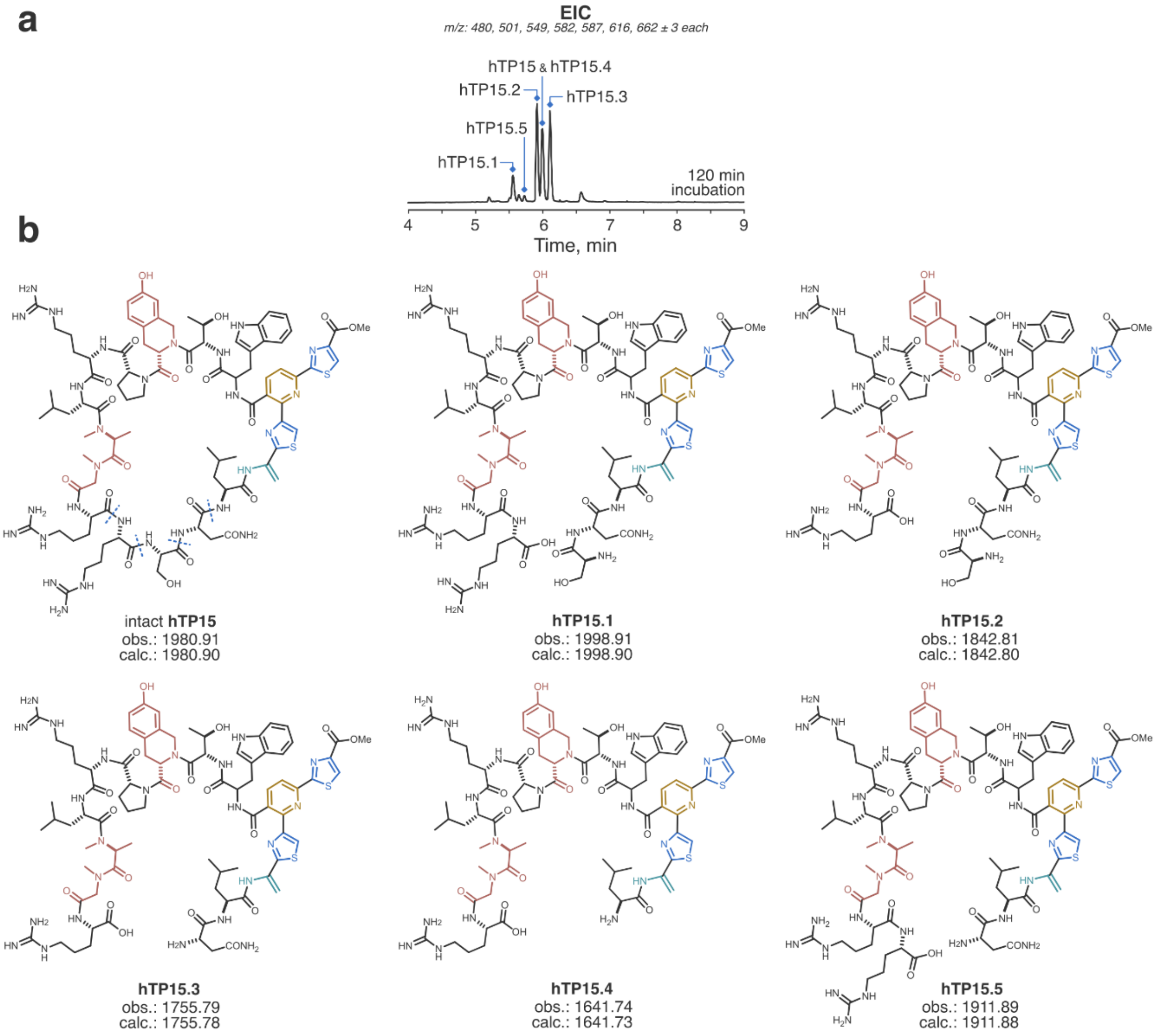

Figure S39. Analysis of hTP15 degradation products in human serum. The compound was incubated in human serum and at various timepoints the results were analyzed by LC/MS. See section 2.10 for experimental details. **a**) Composite EICs for intact hTP15 and some of its degradation products detected after a 120 min incubation with human serum. **b**) Assigned chemical structures of the degradation products indicated in panel a).

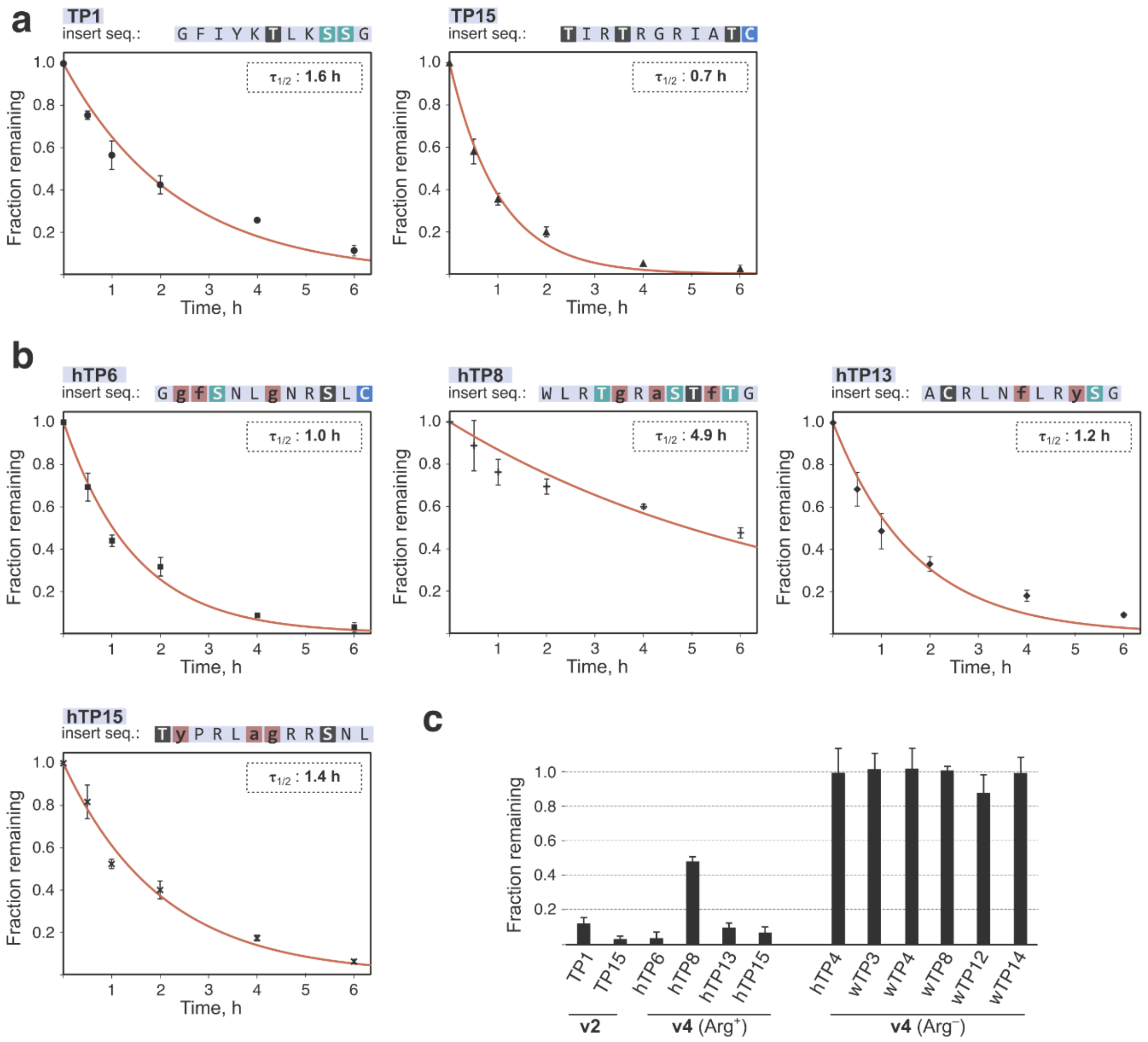

Figure S40. Digestion of the previously reported TP1/TP15[12] (panel **a**) and synthesized compounds (panel **b**) with agarose-immobilized trypsin. See section 2.10 for experimental details. Experimental data are shown in black, and best fit in orange. Insert sequence color coding is as in Fig. 3. Peptides hTP4, wTP3, wTP4, wTP8, wTP12 and wTP14 did not degrade, and are not plotted. **c**) Fraction thiopeptide remaining after a 6 h incubation with trypsin. As expected, the degradation is only observed for Arg or Lys-containing peptides.

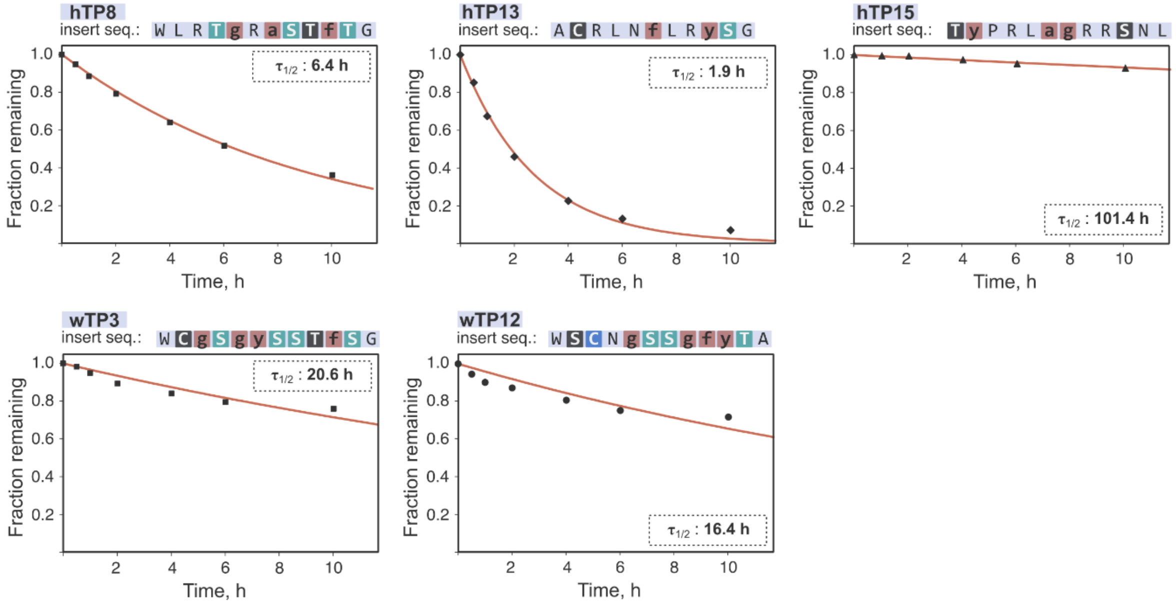

Figure S41. Degradation of hTP8, hTP13, hTP15, wTP3, and wTP12 in the presence of glutathione. See section 2.10 for experimental details. Experimental data are shown in black, and best fit in orange. Insert sequence color coding is as in Fig. 3d.

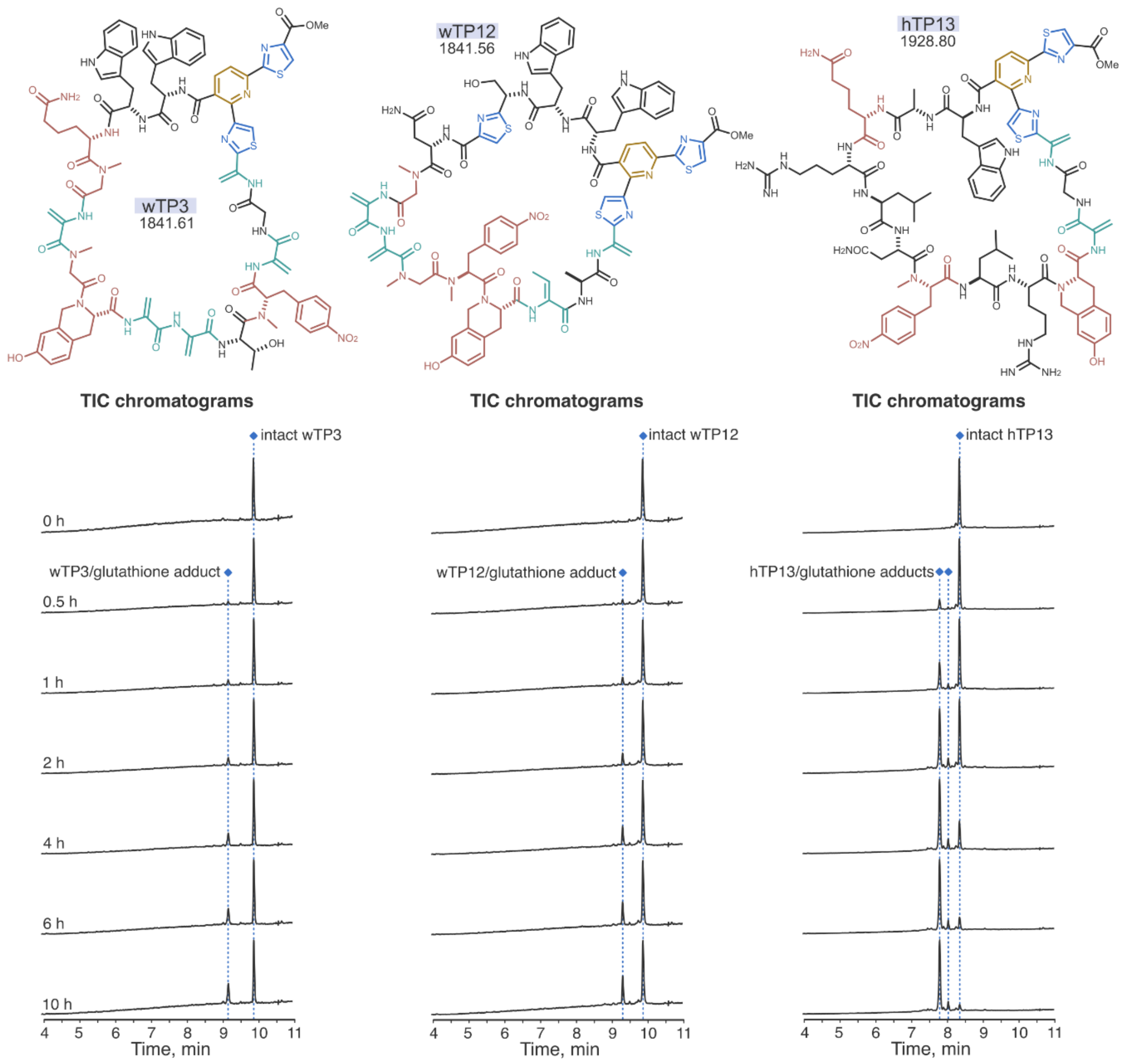

Figure S42. Degradation of wTP3, wTP12, and hTP13 in the presence of glutathione. See section 2.10 for experimental details. Shown are total ion current chromatograms.

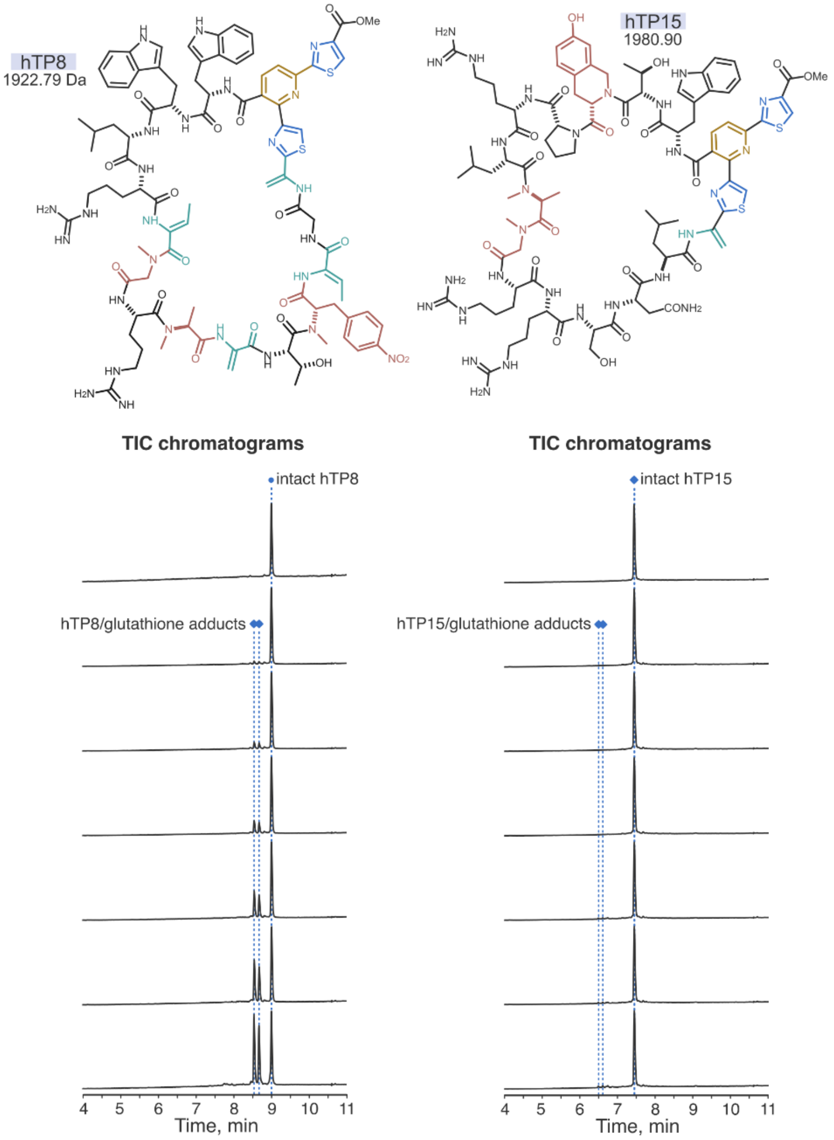

Figure S43. Degradation of hTP8 and hTP15 in the presence of glutathione. See section 2.10 for experimental details. Shown are total ion current chromatograms.

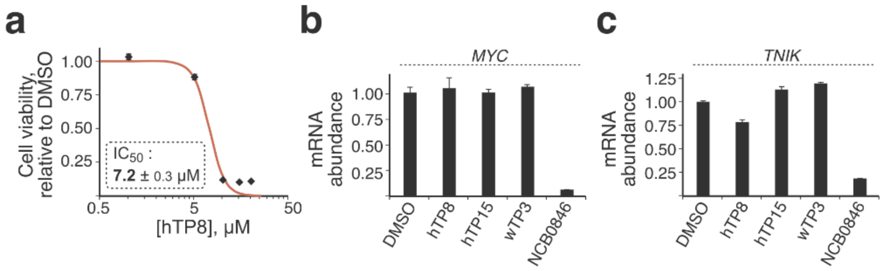

Figure S44. Activity of the discovered thiopeptides in HCT116 cells. See section 2.11 for experimental details. **a**) Dose-dependent cytotoxicity of hTP8 in HCT116 cells. The compound is strongly cytotoxic at concentrations above 5 μM. **c, d**) In contrast to NCB0846, hTP8 and other tested compounds do not significantly downregulate the overall level of MYC and TNIK mRNA in HCT116 cells according to RT-qPCR.

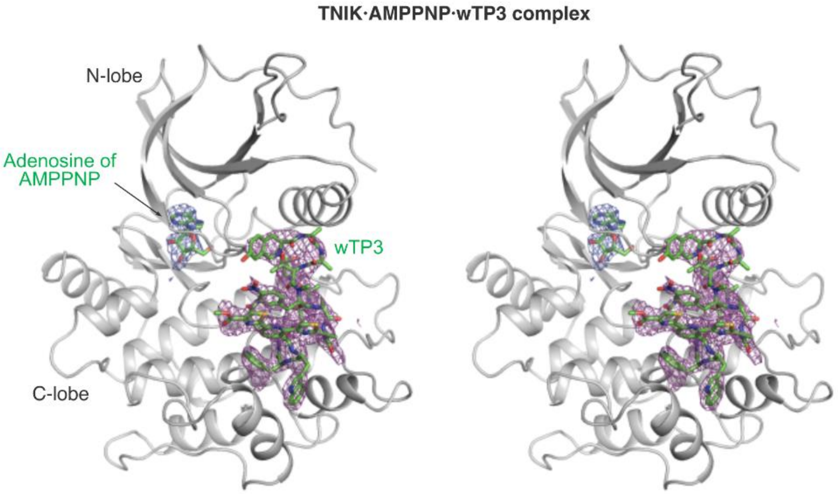

Figure S45. Stereo view of the F_o-F_c omit maps of wTP3 (magenta) and adenosine (blue), both contoured at 2.5 sigma level.

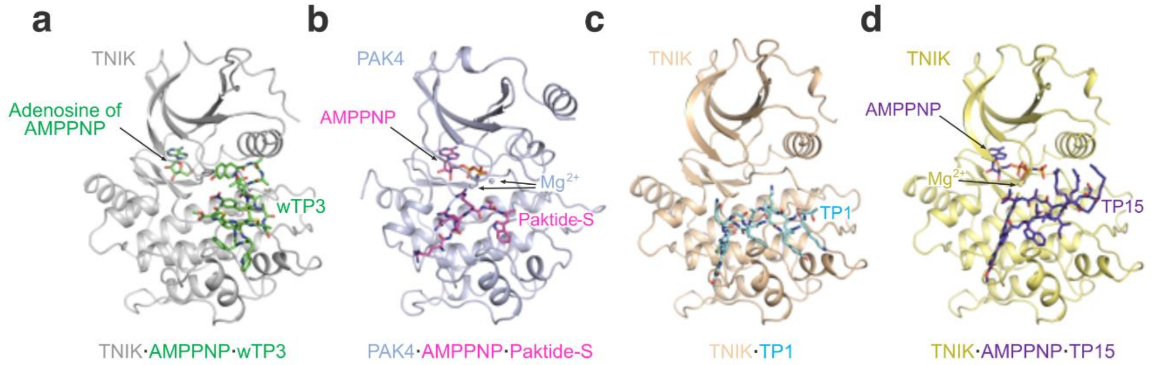

Figure S46. Structural comparison between the TNIK·AMPPNP·wTP3 complex (panel **a**, pdb 8wm0), the PAK4·AMPPNP·Paktide-S complex[28] (panel **b**, pdb 4jdi), the TNIK·TP1 complex[12] (panel **c**, pdb 7xzq) and the TNIK·AMPPNP·TP15 complex[12] (panel **d**, pdb 7xzr).

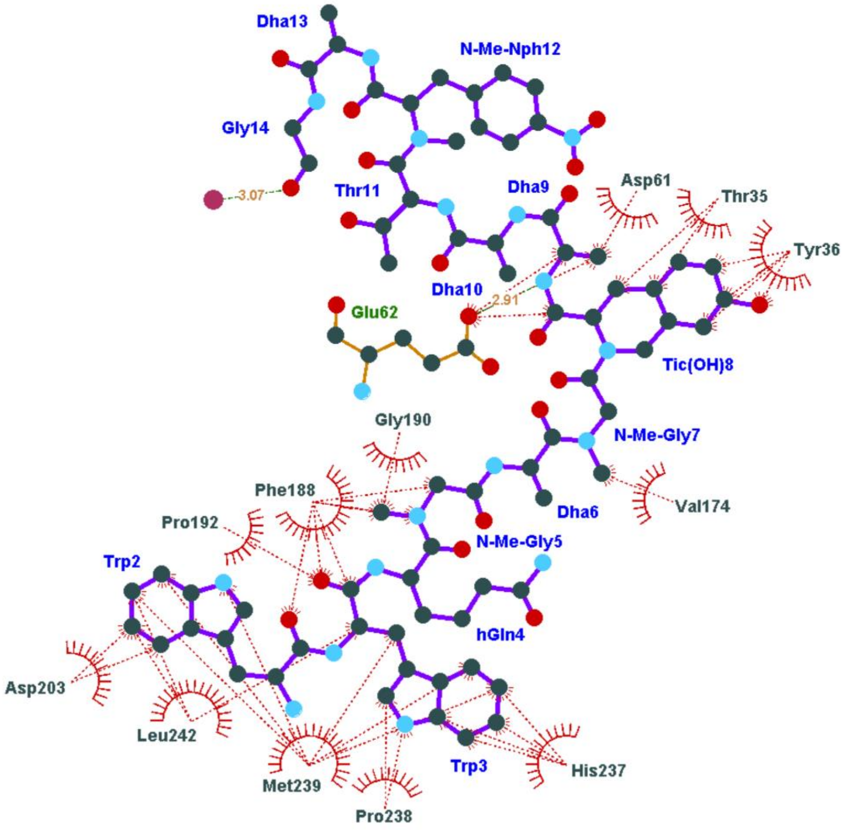

Figure S47. A ligand interaction diagram for wTP3 bound to TNIK. The pyridine-diazole scaffold, which does not contact the protein, is omitted for clarity. The analysis clearly shows the predominantly hydrophobic nature of the interaction. The diagram was generated with LigPlot+ v.2.2.[29]

4. Synthesis of amino acids

The known compound Fmoc–$^{(R,S)}$Abu($^{\beta}$SePh)–OH was prepared according to a published procedure.[30] The syntheses of pyridine-bisazole-containing amino acid **1** (Fig. S32) and Fmoc–Sec(Pmb)–OH were also previously described.[1]

4.1. Synthesis of Fmoc–N^{Me}–Phe(*p*NO_2)–OH

Fmoc–N^{Me}–Phe(*p*NO_2)–OH [(*S*)-2-((((9*H*-fluoren-9-yl)methoxy)carbonyl)(methyl)amino)-3-(4-nitrophenyl)propanoic acid]

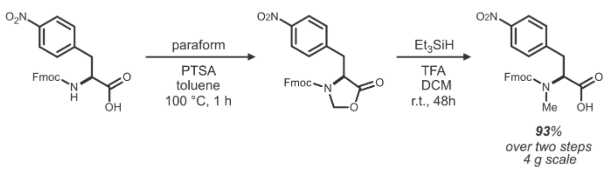

The compound was synthesized by modifying a published general procedure.[31]

Step 1. A Dean-Stark apparatus was charged with Fmoc-^{L}Phe(*p*NO2)-OH (4.32 g; 10 mmol), paraformaldehyde (2 g; 65 mmol), dry toluene (200 mL) and *p*-toluenesulfonic acid monohydrate (PTSA; 200 mg; 1.05 mmol). The mixture was heated (oil bath temperature: 103 ˚C) with constant stirring for 1 h, after which TLC indicated the full consumption of the starting material. The flask was cooled down to room temperature, after which the solution was washed with saturated aqueous sodium bicarbonate (3 × 75 mL) and dried over sodium sulfate. Upon filtering and evaporating the solvent under reduced pressure, the intermediate oxazolidine was obtained as a yellow solid (4.46 g; 10 mmol; quant) that was immediately used in the following reaction.

Step 2. A flame-dried round bottom flask charged with the intermediate oxazolidine (4.44 g; 10 mmol) and a magnetic stirrer was filled with nitrogen and placed under a slight positive pressure of nitrogen. Dry dichloromethane (25 mL), trifluoroacetic acid (25 mL) and triethylsilane (3.49 g; 30 mmol) were sequentially added, and the mixture was stirred at room temperature for 50 h. After, the solvent was evaporated under reduced pressure. The mixture was coevaporated with dichloromethane three more times to remove as much trifluoroacetic acid as possible. The residue was dissolved in a minimal amount of dichloromethane and purified by flash column chromatography using dichloromethane / methanol as the solvent system. The product Fmoc–N^{Me}–Phe(*p*NO_2)–OH was isolated as a light yellow, foamy solid (4.17 g, 9.34 mmol, 93% over two steps).

^{1}H NMR (400 MHz, DMSO-*d*$_6$) δ 8.10 (d, *J* = 18.5, 8.6 Hz, 2H), 7.88 (q, *J* = 5.5 Hz, 2H), 7.51 (d, *J* = 8.4 Hz, 2H), 7.45 – 7.38 (m, 2H), 7.32 – 7.23 (m, 2H), 4.90 (dd, *J* = 11.2, 5.0 Hz,

1H), 4.54 (ddd, J = 101.8, 10.6, 5.2 Hz, 1H), 4.28 – 4.16 (m, 3H), 3.39 – 2.91 (m, 2H), 2.66 (s, 3H).

^{13}C NMR (101 MHz, DMSO-d_6) δ 172.13, 156.22, 146.72, 144.16, 141.27, 130.72, 128.15, 127.54, 125.40, 123.82, 120.63, 67.31, 60.29, 55.43, 46.97, 34.34, 32.38.

TOF-HRMS (*m/z*): calcd for $C_{25}H_{22}N_2O_6Na^+$ (M+Na) = 469.1370; found: 469.1386.

4.2. Synthesis of Fmoc–Tic(pO^{t}Bu)–OH

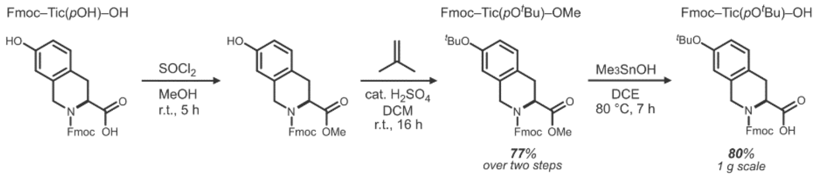

Fmoc–Tic(pO^{t}Bu)–OMe [2-((9*H*-fluoren-9-yl)methyl) 3-methyl (*S*)-7-(tert-butoxy)-3,4-dihydroisoquinoline-2,3(1H)-dicarboxylate]

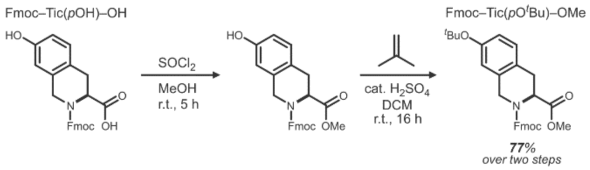

The compound was synthesized by modifying a published procedure for the synthesis of Fmoc-protected Tyr analogs.[32]

Step 1. A flame-dried round bottom flask was charged with Fmoc–Tic(*p*OH)–OH (1.58 g; 3.8 mmol), dry methanol (7.6 mL) and a magnetic stirrer. The flask was filled with nitrogen and placed under a slight positive pressure of nitrogen. Thionyl chloride (551 µL; 7.6 mmol) was added dropwise to the mixture via a syringe with constant stirring at room temperature. The reaction was allowed to proceed at this temperature and with constant stirring for 7 h, after which ethyl acetate (50 mL) and water (20 mL) were added. The organic layer was further washed with saturated aqueous sodium bicarbonate (2 × 15 mL), brine (15 mL) and dried over sodium sulfate. Upon filtering and evaporating the solvent under reduced pressure, the intermediate Fmoc–Tic(*p*OH)–OMe was obtained as a white solid (1.76 g; >100%) that was immediately used in the following reaction.

Step 2. A flame-dried round bottom flask was charged with Fmoc–Tic(*p*OH)–OMe (1.63 g; 3.8 mmol), dry dichloromethane (30 ml), sulfuric acid (103 µL; 1.9 mmol) and a magnetic stirrer. The flask was first purged with isobutylene gas and then placed under a slight positive pressure of the same gas (~5 psi). The reaction was allowed to proceed at room temperature and with constant stirring for 16 h, after which the flask was depressurized. Saturated aqueous sodium bicarbonate (50 mL) and ethyl acetate (100 mL) were added, and the

organic layer was further washed water (30 mL), saturated aqueous sodium bicarbonate (30 mL), brine (30 mL) and dried over sodium sulfate. Upon filtering and evaporating the solvent under reduced pressure, the compound was purified by flash column chromatography using *n*-hexane / ethyl acetate as the solvent system. The product Fmoc–Tic(*p*O^{t}Bu)–OMe was isolated as a white, foamy solid (1.41 g, 2.9 mmol, 76% over two steps).

^{1}H NMR (400 MHz, Chloroform-*d*) δ 7.77 (t, *J* = 7.6 Hz, 2H), 7.68 – 7.51 (m, 2H), 7.45 – 7.26 (m, 4H), 7.04 (t, *J* = 7.5 Hz, 1H), 6.89 – 6.73 (m, 2H), 5.01 (ddd, *J* = 105.1, 5.8, 3.7 Hz, 1H), 4.75 (dd, *J* = 16.3, 6.2 Hz, 1H), 4.61 – 4.50 (m, 2H), 4.44 (dt, *J* = 10.9, 6.1 Hz, 1H), 4.30 (dt, *J* = 41.4, 6.8 Hz, 1H), 3.60 (s, 2H), 3.27 – 3.06 (m, 2H), 1.34 (dd, *J* = 12.4, 0.9 Hz, 9H).

^{13}C NMR (101 MHz, Chloroform-*d*) δ 171.78, 156.19, 154.31, 143.95, 133.03, 128.99, 127.84, 127.21, 126.54, 125.21, 125.00, 122.91, 121.63, 120.12, 78.61, 68.10, 53.51, 52.46, 47.31, 44.54, 30.57, 28.97.

TOF-HRMS (*m/z*): calcd for $C_{30}H_{31}NO_5Na^+$ (M+Na) = 508.2095; found: 508.2147.

Fmoc–Tic(*p*O^{t}Bu)–OH [(*S*)-2-(((9*H*-fluoren-9-yl)methoxy)carbonyl)-7-(tert-butoxy)-1,2,3,4-tetrahydroisoquinoline-3-carboxylic acid]

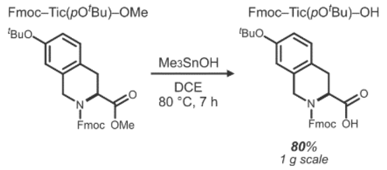

The compound was synthesized by modifying a published general procedure.[33]

A flame-dried two-necked round bottom flask equipped with a magnetic stirring bar and a reflux condenser was charged with Fmoc–Tic(*p*O^{t}Bu)–OMe (1.51 g; 3.11 mmol) and trimethyltin hydroxide (1.69 g; 9.33 mmol), and filled with nitrogen. Anhydrous dichloroethane (21 mL) was added via syringe and the mixture was heated to 80 °C. After 7 h of stirring at this temperature, TLC indicated full consumption of the starting material. The mixture was cooled down to room temperature and the solvent was evaporated under reduced pressure. The residue was redissolved in ethyl acetate (50 mL), extracted with aqueous solution of potassium hydrogen sulfate (5% w/v, 30 mL x 4), washed with brine (20 mL) and dried over sodium sulfate. Upon filtering and evaporating the solvent under reduced pressure, the compound was purified by flash column chromatography using dichloromethane / methanol as the solvent system. The product Fmoc–Tic(*p*O^{t}Bu)–OH was isolated as a white solid (1.18 g, 2.5 mmol, 80%).

^{1}H NMR (400 MHz, Chloroform-*d*) δ 7.80 – 7.71 (m, 2H), 7.60 (dd, *J* = 11.9, 7.5 Hz, 2H), 7.38 (tt, *J* = 7.6, 3.8 Hz, 2H), 7.31 (q, *J* = 7.5 Hz, 3H), 7.13 (t, *J* = 7.4 Hz, 1H), 7.03 (d, *J* =

8.2 Hz, 1H), 6.80 (ddd, J = 16.2, 8.2, 2.1 Hz, 2H), 5.11 (dd, J = 6.2, 3.5 Hz, 1H), 4.69 (d, J = 16.6 Hz, 2H), 4.52 (dt, J = 13.0, 6.4 Hz, 2H), 4.31 (t, J = 7.1 Hz, 1H), 3.28 – 3.14 (m, 1H), 3.14 – 3.01 (m, 2H), 1.33 (s, 9H).

^{13}C NMR (101 MHz, Chloroform-*d*) δ 176.59, 155.51, 154.37, 143.89, 141.47, 132.89, 129.07, 127.88, 127.25, 125.22, 124.86, 123.03, 121.63, 120.13, 78.75, 68.22, 53.32, 47.30, 44.49, 30.26, 28.97.

TOF-HRMS (*m/z*): calcd for $C_{29}H_{29}NO_5Na^+$ (M+Na) = 494.1938; found: 494.1972.

4.3. Synthesis of Fmoc–Leu–Thz–OH

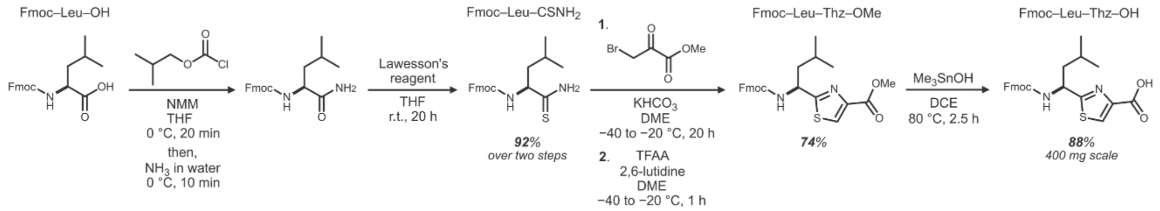

Fmoc–Leu–CSNH₂ [(9H-fluoren-9-yl)methyl (S)-(1-amino-4-methyl-1-thioxopentan-2-yl)carbamate]

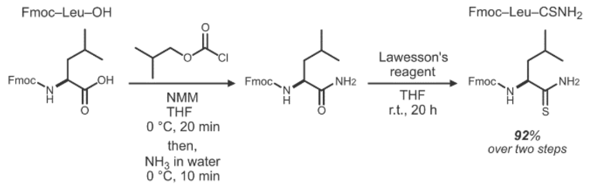

The known compound[34] was synthesized according to a published procedure.[1]

Step 1. A flame-dried three-necked round bottom flask equipped with a magnetic stirring bar was charged with Fmoc-^{L}Leu-OH (1.06 g, 3 mmol) and filled with nitrogen. Anhydrous THF (15 mL) was added via syringe and the mixture was cooled down in an ice bath. To the resulting solution was added *N*-methylmorpholine (0.363 mL, 3.3 mmol), and then isobutyl chloroformate was added dropwise (0.428 mL, 3.3 mmol). The mixture was stirred for 20 min at which point TLC indicated full consumption of the starting material. Then, ammonia (28% solution in water) (1.02 mL, 15 mmol) was added dropwise to the ice-cold, vigorously stirred mixture. After completion of the reaction (5–10 min), the solvent was reduced under reduced pressure to ca. 5 mL, and the mixture was partitioned between water (20 mL) and ethyl acetate (30 mL). The organic layer was washed with saturated aqueous solution of ammonium chloride (2 × 20 mL), brine (80 mL) and then dried over anhydrous sodium sulfate. Upon filtering and evaporating the solvent under reduced pressure, the intermediate Fmoc-^{L}Leu-$CONH_2$ was obtained as a white solid (1.06 g; 3 mmol; quant.) that was immediately used in the following reaction.

Step 2. A flame-dried round bottom flask equipped with a magnetic stirring bar was charged with Fmoc-^{L}Leu-$CONH_2$ (1.05 g, 3 mmol) and Lawesson's reagent (607 mg, 1.5 mmol). The flask was filled with nitrogen and anhydrous THF (60 mL) was added via syringe at room temperature. The resulting mixture was stirred for 16 h at the same temperature, after which the solvent was removed under reduced pressure. The residue was redissolved in a minimal amount of DCM and purified by flash column chromatography using *n*-hexane / ethyl acetate as the solvent system. The product Fmoc–Leu–$CSNH_2$ was isolated as a white, foamy solid (1.02 g, 2.77 mmol, 92% over two steps).

^{1}H NMR (400 MHz, Chloroform-*d*) δ 7.89 (s, 1H), 7.75 (d, *J* = 7.5 Hz, 2H), 7.62 (s, 1H), 7.57 (dd, *J* = 7.4, 2.7 Hz, 2H), 7.39 (t, *J* = 7.4 Hz, 2H), 7.30 (td, *J* = 7.5, 1.2 Hz, 2H), 5.50 (d, *J* = 8.4 Hz, 1H), 4.53 (s, 1H), 4.39 (t, *J* = 6.9 Hz, 2H), 4.19 (t, *J* = 6.8 Hz, 1H), 1.66 (s, 2H), 0.91 (s, 6H).

^{13}C NMR (101 MHz, Chloroform-*d*) δ 210.22, 156.52, 143.65, 141.33, 127.86, 127.26, 125.16, 120.11, 67.23, 58.47, 47.14, 44.59, 24.91, 22.93, 22.04.

TOF-HRMS (*m/z*): calcd for $C_{21}H_{24}N_2O_2SNa^+$ (M+Na) = 391.1451; found: 391.1469.

Fmoc–Leu–Thz–OMe [methyl (S)-2-(1-((((9H-fluoren-9-yl)methoxy)carbonyl)amino)-3-methylbutyl)thiazole-4-carboxylate]

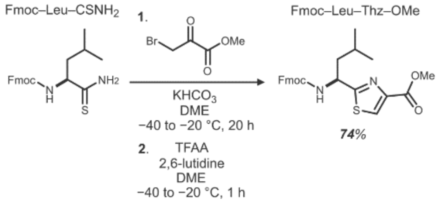

The compound[34] was synthesized by modifying a published procedure.[1]

A flame-dried round bottom flask equipped with a magnetic stirring bar was charged with Fmoc–Leu–$CSNH_2$ (800 mg, 2.17 mmol) and anhydrous potassium bicarbonate (435 mg, 4.35 mmol, oven-dried at 90 °C overnight). The flask was filled with nitrogen and, after adding anhydrous dimethoxyethane (5.1 mL), was cooled to –40 °C. To the resulting mixture, methyl 3-bromopyruvate (386 µL, 3.62 mmol) was added in one portion, and the bath temperature was adjusted to –20 °C. The reaction was then allowed to proceed at the same temperature for 20 h with continuous stirring. Solids were filtered, and the solvent was removed under reduced pressure. The residue was redissolved in dimethoxyethane (2.5 mL) and cooled to –40 °C. To the resulting solution, a freshly prepared mixture of trifluoroacetic acid anhydride (755 µL, 5.43 mmol) and 2,6-lutidine (1.68 mL, 14.5 mmol) in dimethoxyethane (1.75 mL) was added dropwise under nitrogen, and the bath temperature was adjusted to –20 °C. After stirring for 1 h at this temperature, the flask was warmed up

to room temperature and the solvent was removed under reduced pressure. The residue was redissolved in diethyl ether (30 mL) and washed with aqueous solution of citric acid (10%; 2 × 20 mL), saturated aqueous solution of sodium bicarbonate (20 mL) and brine (20 mL). The organic layer was dried over anhydrous sodium sulfate, and the compound was further purified by flash column chromatography using ethyl acetate / *n*-hexane as the solvent system. The product Fmoc–Leu–Thz–OMe was isolated as a yellow, foamy solid (720 mg, 1.6 mmol, 74%).

^{1}H NMR (400 MHz, Chloroform-*d*) δ 8.07 (s, 1H), 7.74 (d, *J* = 7.6 Hz, 2H), 7.61 – 7.48 (m, 2H), 7.38 (t, *J* = 7.4 Hz, 2H), 7.29 (t, *J* = 7.4 Hz, 2H), 5.45 (d, *J* = 8.4 Hz, 1H), 5.12 (q, *J* = 8.9 Hz, 1H), 4.47 (dt, *J* = 15.1, 7.5 Hz, 2H), 4.19 (t, *J* = 6.5 Hz, 1H), 3.93 (s, 3H), 1.98 – 1.87 (m, 1H), 1.85 – 1.56 (m, 2H), 0.99 – 0.84 (m, 6H).

^{13}C NMR (101 MHz, Chloroform-*d*) δ 173.87, 161.85, 155.78, 147.07, 143.73, 141.42, 127.78, 127.42, 127.14, 125.06, 120.07, 66.89, 52.54, 51.79, 47.32, 44.45, 24.95, 23.00, 21.79.

TOF-HRMS (*m/z*): calcd for $C_{25}H_{26}N_2O_4SNa^+$ (M+Na) = 473.1506; found: 473.1537.

Fmoc–Leu–Thz–OH [(S)-2-(1-((((9H-fluoren-9-yl)methoxy)carbonyl)amino)-3-methyl-butyl)thiazole-4-carboxylic acid]

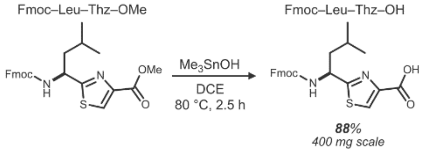

The compound[34] was synthesized by modifying a published procedure.[1]

A flame-dried two-necked round bottom flask equipped with a magnetic stirring bar and a reflux condenser was charged with Fmoc–Leu–Thz–OMe (541 mg, 1.2 mmol) and trimethyltin hydroxide (651 mg, 3.6 mmol), and filled with nitrogen. Anhydrous dichloroethane (7.5 mL) was added via syringe and the mixture was then heated to 80°C in an oil bath. After 2.5 h of stirring at this temperature, TLC indicated full consumption of the starting material. The mixture was cooled down to room temperature and the solvent was reduced under reduced pressure. The residue was redissolved in ethyl acetate (60 mL), extracted with aqueous solution of potassium hydrogen sulfate (5% w/v, 4 × 40 mL), washed with brine (20 mL) and dried over anhydrous sodium sulfate. Upon filtering and evaporating the solvent under reduced pressure, the compound was purified by flash column chromatography using dichloromethane / methanol as the solvent system. The product Fmoc–Leu–Thz–OH was isolated as an off-white solid (460 mg, 1.05 mmol, 88%).

^{1}H NMR (400 MHz, Chloroform-*d*) δ 8.18 (s, 1H), 7.74 (d, J = 7.4 Hz, 2H), 7.57 (d, J = 6.5 Hz, 2H), 7.37 (t, J = 7.2 Hz, 2H), 7.28 (t, J = 7.3 Hz, 2H), 5.70 (d, J = 7.1 Hz, 1H), 5.13 (q, J = 8.3 Hz, 1H), 4.45 (p, J = 10.4 Hz, 2H), 4.19 (t, J = 6.3 Hz, 1H), 1.95 – 1.76 (m, 2H), 1.66 (dt, J = 14.9, 8.5 Hz, 1H), 0.95 (d, J = 6.5 Hz, 6H).

^{13}C NMR (101 MHz, Chloroform-*d*) δ 174.22, 164.17, 155.95, 146.73, 143.86, 143.78, 141.47, 128.59, 127.85, 127.20, 125.16, 120.12, 66.97, 51.64, 47.36, 44.36, 25.00, 22.98.

TOF-HRMS (*m/z*): calcd for $C_{24}H_{24}N_2O_4SNa^+$ (M+Na) = 459.1349; found: 459.1389.

4.4. Synthesis of Fmoc–Val–Thz–OH

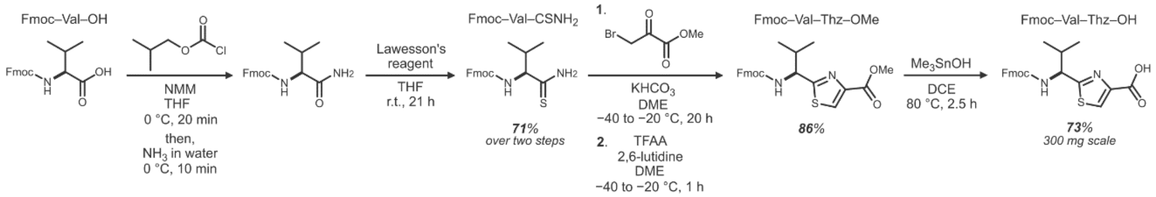

Fmoc–Val–CSNH₂ [(9*H*-fluoren-9-yl)methyl (*S*)-(1-amino-3-methyl-1-thioxobutan-2-yl)carbamate]

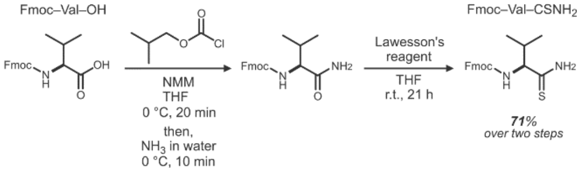

The known compound[35] was synthesized following the procedure for Fmoc–Leu–$CSNH_2$ (section 4.3) using Fmoc-^{L}Val-OH (1.02 g, 3 mmol) as the starting material. The product Fmoc–Val–$CSNH_2$ was isolated as a white foamy solid (750 mg, 2.11 mmol, 70%).

^{1}H NMR (400 MHz, Chloroform-*d*) δ 7.86 (s, 1H), 7.76 (d, *J* = 7.5 Hz, 2H), 7.65 – 7.52 (m, 3H), 7.40 (t, *J* = 7.5 Hz, 2H), 7.31 (tdd, *J* = 7.4, 2.2, 1.1 Hz, 2H), 5.64 (d, *J* = 8.1 Hz, 1H), 4.37 (d, *J* = 6.2 Hz, 2H), 4.21 (t, *J* = 6.9 Hz, 1H), 2.22 – 2.11 (m, 1H), 1.07 – 0.87 (m, 6H).

^{13}C NMR (101 MHz, Chloroform-*d*) δ 208.99, 156.63, 143.75, 141.43, 127.95, 127.33, 125.19, 120.19, 67.31, 65.67, 47.27, 33.40, 19.69, 18.28.

TOF-HRMS (*m/z*): calcd for $C_{20}H_{22}N_2O_2SNa^+$ (M+Na) = 377.1294; found: 377.1296.

Fmoc–Val–Thz–OMe [methyl (S)-2-(1-((((9H-fluoren-9-yl)methoxy)carbonyl)amino)-2-methylpropyl)thiazole-4-carboxylate]

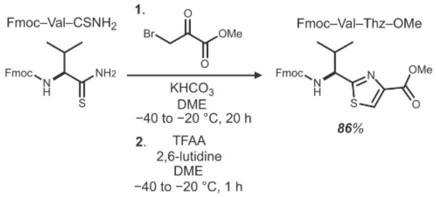

The compound was synthesized following the procedure for Fmoc–Leu–Thz–OMe (section 4.3) using Fmoc–Val–$CSNH_2$ (720 mg, 2 mmol) as the starting material. The product Fmoc–Val–Thz–OMe was isolated as a yellow foamy solid (752 mg, 1.72 mmol, 86%).

^{1}H NMR (400 MHz, Chloroform-*d*) δ 8.08 (s, 1H), 7.74 (d, *J* = 7.4 Hz, 2H), 7.58 (dd, *J* = 7.7, 3.8 Hz, 2H), 7.38 (t, *J* = 7.5 Hz, 2H), 7.29 (t, *J* = 7.5 Hz, 2H), 5.63 (d, *J* = 9.1 Hz, 1H), 4.93 (dd, *J* = 9.1, 6.1 Hz, 1H), 4.44 (d, *J* = 6.7 Hz, 2H), 4.21 (t, *J* = 6.6 Hz, 1H), 3.93 (s, 3H), 2.42 (dq, *J* = 13.0, 6.3 Hz, 1H), 1.00 – 0.88 (m, 6H).

^{13}C NMR (101 MHz, Chloroform-*d*) δ 172.49, 161.82, 156.11, 147.11, 143.75, 141.39, 127.77, 127.29, 127.12, 125.06, 120.05, 66.98, 58.68, 52.50, 47.29, 33.41, 19.50, 17.66.

TOF-HRMS (*m/z*): calcd for $C_{24}H_{25}N_2O_4S^+$ (M+H) = 437.1529; found: 437.1539.

Fmoc–Val–Thz–OH [(S)-2-(1-((((9H-fluoren-9-yl)methoxy)carbonyl)amino)-2-methyl-propyl)thiazole-4-carboxylic acid]

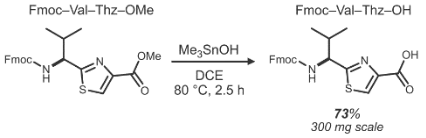

The known compound[36] was synthesized following the procedure for Fmoc–Leu–Thz–OH (section 4.3) using Fmoc–Val–Thz–OMe (537 mg, 1.23 mmol) as the starting material. The product Fmoc–Val–Thz–OH was isolated as an off-white solid (379 mg, 0.90 mmol, 73%).

^{1}H NMR (400 MHz, Chloroform-*d*) δ 9.41 (brs, 1H), 8.22 (s, 1H), 7.74 (d, *J* = 7.5 Hz, 2H), 7.60 (t, *J* = 7.3 Hz, 2H), 7.37 (t, *J* = 6.7 Hz, 2H), 7.31 – 7.26 (m, 2H), 5.90 (d, *J* = 9.2 Hz, 1H), 4.93 (t, *J* = 7.8 Hz, 1H), 4.44 (d, *J* = 6.6 Hz, 2H), 4.20 (t, *J* = 6.6 Hz, 1H), 2.42 (q, *J* = 6.8 Hz, 1H), 0.95 (t, *J* = 7.3 Hz, 6H).

^{13}C NMR (101 MHz, Chloroform-d) δ 173.00, 164.24, 156.29, 146.76, 143.87, 141.46, 128.66, 127.84, 127.19, 125.14, 120.11, 67.08, 58.68, 47.34, 33.40, 19.64, 18.00.

TOF-HRMS (m/z): calcd for $C_{23}H_{23}N_2O_4S$+ (M+H) = 423.1373; found: 423.1384.

4.5. Synthesis of Fmoc–Ser(O^{t}Bu)–Thz–OH

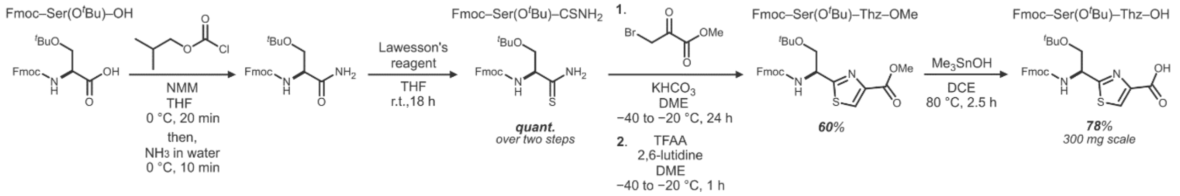

Fmoc–Ser(O^{t}Bu)–CSNH$_2$ [(9*H*-fluoren-9-yl)methyl (*S*)-(1-amino-3-(tert-butoxy)-1-thioxopropan-2-yl)carbamate]

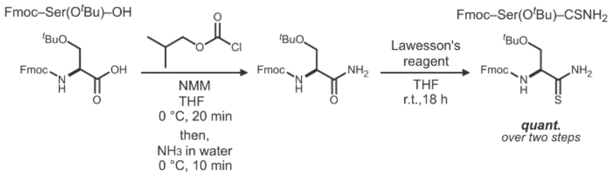

The known compound[34,37,38] was synthesized following the procedure for Fmoc–Leu–CSNH$_2$ (section 4.3) using Fmoc–^{L}Ser(O^{t}Bu)-OH (2.3 g, 6 mmol) as the starting material. The product Fmoc–Ser(O^{t}Bu)–CSNH$_2$ was isolated as a white foamy solid (2.43 g, 6 mmol, quant).

^{1}H NMR (400 MHz, Chloroform-*d*) δ 7.97 (s, 1H), 7.76 (d, *J* = 7.5 Hz, 2H), 7.65 – 7.55 (m, 2H), 7.39 (t, *J* = 7.5 Hz, 2H), 7.31 (td, *J* = 7.4, 1.1 Hz, 2H), 6.03 (s, 1H), 4.53 – 4.35 (m, 3H), 4.23 (t, *J* = 7.0 Hz, 1H), 3.85 (dd, *J* = 8.8, 4.0 Hz, 1H), 3.47 (s, 1H), 1.19 (s, 9H).

^{13}C NMR (101 MHz, Chloroform-d) δ 206.30, 155.85, 143.84, 141.44, 127.87, 127.21, 125.28, 125.20, 120.13, 77.36, 74.50, 63.99, 47.27, 27.55.

TOF-HRMS (*m/z*): calcd for $C_{22}H_{26}N_2O_3SNa^+$ (M+Na) = 421.1556; found: 421.1576.

Fmoc–Ser(O^{t}Bu)–Thz–OMe [methyl (*S*)-2-(1-((((9*H*-fluoren-9-yl)methoxy)carbonyl)amino)-2-(tert-butoxy)ethyl)thiazole-4-carboxylate]

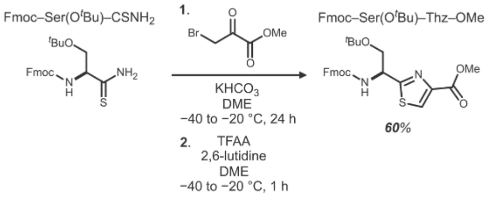

The compound was synthesized following the procedure for Fmoc–Leu–Thz–OMe (section 4.3) using Fmoc–Ser(O^{t}Bu)–CSNH$_2$ (2.39 g, 6 mmol) as the starting material. The product Fmoc–Ser(O^{t}Bu)–Thz–OMe was isolated as a yellow foamy solid (1.73 g, 3.6 mmol, 60%).

^{1}H NMR (400 MHz, Chloroform-*d*) δ 8.10 (s, 1H), 7.76 (d, *J* = 7.5 Hz, 2H), 7.61 (s, 2H), 7.46 – 7.27 (m, 4H), 5.95 (d, *J* = 7.8 Hz, 1H), 5.22 (s, 1H), 4.45 (dq, *J* = 17.6, 10.1 Hz, 2H), 4.24 (t, *J* = 6.8 Hz, 1H), 3.94 (s, 3H), 3.70 (dd, *J* = 9.0, 4.7 Hz, 1H), 1.10 (s, 9H).

^{13}C NMR (101 MHz, Chloroform-*d*) δ 172.39, 161.93, 155.91, 146.64, 143.80, 141.38, 127.87, 127.78, 127.12, 125.07, 120.07, 73.99, 67.18, 63.58, 54.13, 52.47, 47.25, 27.37.

TOF-HRMS (*m/z*): calcd for $C_{26}H_{28}N_2O_5SNa^+$ (M+Na) = 503.1611; found: 503.1659.

Fmoc–Ser(O^{t}Bu)–Thz–OH [(*S*)-2-(1-((((9*H*-fluoren-9-yl)methoxy)carbonyl)amino)-2-(tert-butoxy)ethyl)thiazole-4-carboxylic acid]

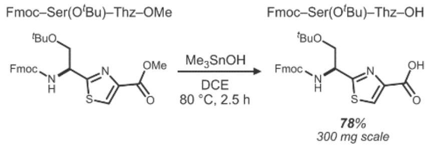

The known compound[34,37,38] was synthesized following the procedure for Fmoc–Leu–Thz–OH (section 4.3) using Fmoc–Ser(O^{t}Bu)–Thz–OMe (481 mg, 1 mmol) as the starting material. The product Fmoc–Ser(O^{t}Bu)–Thz–OH was isolated as a white solid (365 mg, 0.78 mmol, 78%).

^{1}H NMR (400 MHz, Chloroform-*d*) δ 10.93 (brs, 1H), 8.21 (s, 1H), 7.75 (d, *J* = 7.6 Hz, 2H), 7.60 (s, 2H), 7.43 – 7.26 (m, 4H), 6.08 (d, *J* = 6.3 Hz, 1H), 5.26 (s, 1H), 4.56 – 4.35 (m, 2H), 4.23 (t, *J* = 7.2 Hz, 1H), 3.90 (d, *J* = 9.0 Hz, 1H), 3.71 (s, 1H), 1.11 (s, 9H).

^{13}C NMR (101 MHz, Chloroform-*d*) δ 172.56, 164.53, 156.06, 146.17, 143.75, 141.33, 129.17, 127.76, 127.11, 125.08, 120.03, 74.09, 67.21, 63.50, 53.97, 47.19, 27.32.

TOF-HRMS (*m/z*): calcd for $C_{25}H_{27}N_2O_5S^+$ (M+H) = 467.1635; found: 467.1678.

4.6. Synthesis of Fmoc–$^{(R,S)}$Abu($^{\beta}$SePh)–Thz–OH

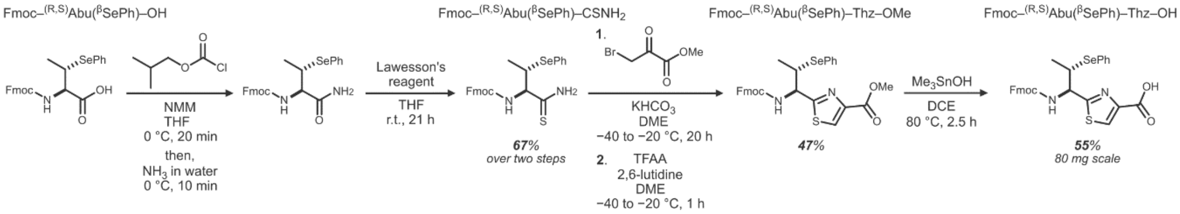

Fmoc–$^{(R,S)}$Abu($^{\beta}$SePh)–CSNH$_2$ [(9*H*-fluoren-9-yl)methyl ((2*R*,3*S*)-1-amino-3-(phenyl selanyl)-1-thioxobutan-2-yl)carbamate]

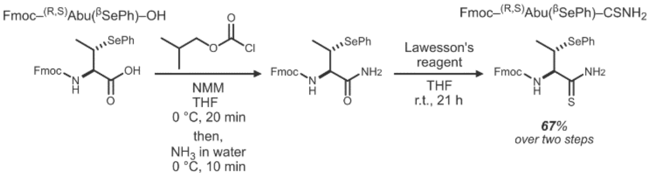

The compound was synthesized following the procedure for Fmoc–Leu–CSNH$_2$ (section 4.3) using Fmoc–$^{(R,S)}$Abu($^{\beta}$SePh)–OH (480 mg, 1 mmol; synthesized according to a published procedure[30]) as the starting material. The product Fmoc–$^{(R,S)}$Abu($^{\beta}$SePh)–CSNH$_2$ was isolated as a white foamy solid (367 mg, 0.74 mmol, 74%).

^{1}H NMR (400 MHz, Chloroform-d) δ 7.76 (d, *J* = 7.6 Hz, 2H), 7.61 – 7.54 (m, 4H), 7.40 (td, *J* = 7.4, 3.0 Hz, 2H), 7.32 (ddd, *J* = 7.5, 4.7, 1.2 Hz, 2H), 7.29 – 7.26 (m, 2H), 7.25 (s, 1H), 5.69 (s, 1H), 4.42 (d, *J* = 6.8 Hz, 2H), 4.20 (s, 1H), 3.84 (d, *J* = 6.7 Hz, 1H), 1.39 (d, *J* = 3.8 Hz, 3H), 0.93 (d, *J* = 6.7 Hz, 1H).

^{13}C NMR (101 MHz, Chloroform-*d*) δ 205.93, 156.33, 143.67, 143.62, 141.43, 135.13, 129.37, 128.32, 127.93, 127.30, 127.26, 125.19, 125.12, 120.17, 67.50, 47.21, 19.09.

TOF-HRMS (*m/z*): calcd for $C_{25}H_{24}N_2O_2SSeNa^+$ (M+Na) = 519.06159; found: 519.0634.

Fmoc–$^{(R,S)}$Abu($^{\beta}$SePh)–Thz–OMe [methyl 2-((1*R*,2*S*)-1-((((9*H*-fluoren-9-yl)methoxy) carbonyl)amino)-2-(phenylselanyl)propyl)thiazole-4-carboxylate]

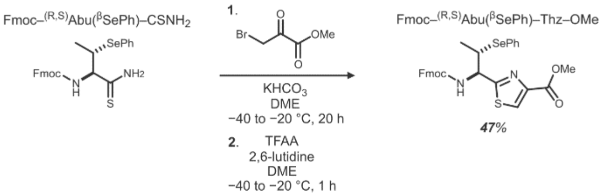

The compound was synthesized following the procedure for Fmoc–Leu–Thz–OMe (section 4.3) using Fmoc–$^{(R,S)}$Abu($^{\beta}$SePh)–CSNH$_2$ (302 mg, 0.61 mmol) as the starting material. The product Fmoc–$^{(R,S)}$Abu($^{\beta}$SePh)–Thz–OMe was isolated as a yellow foamy solid (165 mg, 0.28 mmol, 47%).

^{1}H NMR (400 MHz, Chloroform-*d*) δ 8.08 (s, 1H), 7.76 (d, *J* = 7.9 Hz, 2H), 7.65 – 7.49 (m, 4H), 7.40 (t, *J* = 6.8 Hz, 3H), 7.35 – 7.26 (m, 3H), 7.24 (s, 1H), 7.15 (dd, *J* = 16.4, 8.4 Hz, 1H), 5.75 (d, *J* = 8.5 Hz, 1H), 5.27 (s, 1H), 4.44 (d, *J* = 6.8 Hz, 2H), 4.28 – 4.20 (m, 1H), 3.92 (d, *J* = 11.4 Hz, 3H), 1.48 – 1.42 (m, 3H), 0.93 (d, *J* = 6.7 Hz, 1H).

^{13}C NMR (101 MHz, Chloroform-*d*) δ 161.76, 147.23, 143.82, 141.50, 135.41, 129.35, 128.31, 127.91, 127.83, 127.25, 125.17, 120.18, 67.35, 57.59, 52.61, 52.49, 47.32, 43.23, 28.07, 19.14, 18.15.

TOF-HRMS (*m/z*): calcd for $C_{29}H_{26}N_2O_4SSeNa^+$ (M+Na) = 601.0670; found: 601.0677.

Fmoc–Ser(O^{t}Bu)–Thz–OH [2-((1*R*,2*S*)-1-((((9*H*-fluoren-9-yl)methoxy)carbonyl)amino)-2-(phenylselanyl)propyl)thiazole-4-carboxylic acid]

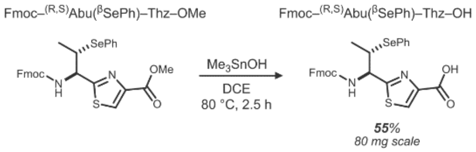

The compound was synthesized following the procedure for Fmoc–Leu–Thz–OH (section 4.3) using Fmoc–$^{(R,S)}$Abu($^{\beta}$SePh)–Thz–OMe (150 mg, 0.26 mmol) as the starting material. The product Fmoc–$^{(R,S)}$Abu($^{\beta}$SePh)–Thz–OH was isolated as a white solid (81 mg, 0.14 mmol, 55%).

^{1}H NMR (400 MHz, Chloroform-*d*) δ 9.22 (s, 1H), 8.17 (s, 1H), 7.76 (t, *J* = 8.6 Hz, 2H), 7.58 (d, *J* = 8.1 Hz, 2H), 7.52 (dd, *J* = 7.8, 1.8 Hz, 2H), 7.38 (t, *J* = 7.3 Hz, 3H), 7.27 (d, *J* = 8.6 Hz, 2H), 7.23 – 7.20 (m, 1H), 7.15 (dd, *J* = 13.6, 6.4 Hz, 1H), 5.98 (d, *J* = 8.8 Hz, 1H), 5.27 (t, *J* = 7.3 Hz, 1H), 4.44 (d, *J* = 6.9 Hz, 2H), 4.22 (dd, *J* = 14.6, 6.6 Hz, 1H), 3.87 (d, *J* = 6.7 Hz, 1H), 1.46 (d, *J* = 7.3 Hz, 3H), 0.93 (d, *J* = 6.6 Hz, 1H).

^{13}C NMR (101 MHz, Chloroform-*d*) δ 171.33, 164.16, 155.93, 146.65, 143.73, 141.43, 135.31, 129.32, 129.11, 128.31, 127.87, 127.21, 125.13, 120.13, 71.73, 67.32, 57.54, 47.25, 27.99, 19.07.

TOF-HRMS (*m/z*): calcd for $C_{28}H_{24}N_2O_4SSeNa^+$ (M+Na) = 587.0514; found: 587.0534.

5. Synthesis of thiopeptides

5.1. General procedures

Fmoc solid-phase peptide synthesis (SPPS). Synthesis was performed manually on 2-chlorotrityl chloride resin (100 – 200 mesh, 1% DVB; Novabiochem®, Merck; loading: 1.00 – 1.80 mmol/g). First, anhydrous dichloromethane (DCM; 1.5 mL) was used to swell the resin for 15 min. To the swollen resin, a solution of the first amino acid (4 eq. w.r.t. the initial resin loading) and *N,N*-diisopropylethylamine (DIPEA; 8 eq.) in anhydrous DCM (1 – 1.5 mL) was added. The reaction mixture was shaken at room temperature for 2 h. After aspiration, the resin was washed with DCM (3 × 2 mL) and DMF (3 × 2 mL). The resin was subsequently submitted to iterative peptide assembly via the Fmoc-SPPS protocol described below.

The iterative amino acids coupling to the pre-loaded resin was performed using a mixture of an Fmoc-amino acid (4.5 eq.), 1-[bis(dimethylamino)methylene]-1*H*-1,2,3triazolo[4,5-b]pyridinium 3-oxide hexafluorophosphate (HATU; 4.0 eq.) and *N,N*-diisopropylethylamine (DIPEA; 8.0 eq.) in DMF (1 mL) for 1 hour at room temperature. The resin was then washed with DCM and DMF, and subjected to Fmoc deprotection (15 ~ 20 min; 20% piperidine in DMF).

For the coupling of amino acids or building blocks that come immediately after the *N*-methyl amino acids, a solution of Fmoc-amino acid (4 eq. to the resin loading), (1-cyano-2-ethoxy-2-oxoethylidenaminooxy)dimethylamino-morpholino-carbenium hexafluorophosphate (COMU; 4 eq.), DIPEA (8.0 eq.) in anhydrous DMF (0.4 – 0.6 mL) was gently agitated on a shaker with the resin at room temperature for 2 h. The coupling was repeated once for 2 h or overnight. The resin was then washed with DMF (3 × 2 mL) and DCM (2 mL). Before Fmoc-deprotection, the residual unreacted amino group on the resin was capped using the DCM : pyridine : acetic anhydride (1 mL; 2 : 1 : 1, *v : v : v*) mixture for 30 min at room temperature.

For the coupling of central amino acid **1**, a solution of **1** (1.2 eq. w.r.t. the resin loading), benzotriazol-1-yloxytripyrrolidinophosphonium hexafluorophosphate (PyBOP) (1.2 eq.), DIPEA (2.4 eq.) in anhydrous DMF (0.4 – 0.6 mL) was gently agitated on shaker with the resin at room temperature for 12 h. The resin was then washed with DMF (3 × 2 mL) and DCM (2 mL). Residual unreacted amine was capped using the DCM : pyridine : acetic anhydride (1 mL; 2 : 1 : 1, *v : v : v*) mixture for 30 min at room temperature, after which the solution was aspirated and resin was washed by DMF (5 × 2 mL). Fmoc deprotection after the coupling of Se-containing building blocks was shortened to 2 × 3 min treatments (20% piperidine in DMF, 2 mL each time). Additionally, Fmoc deprotection for *N*-methyl amino acids was changed to 3 × 3 min treatments (20% piperidine in DMF, 2 mL each time). After

Fmoc deprotection, the resin was washed with DMF (3 × 2 mL), DCM (3 × 2 mL) and DMF (3 × 2 mL), and the next amino acid was coupled as described above.

In-solution macrocyclization and global deprotection. The resin was washed with DCM and treated with a mild acidic cleavage cocktail consisting of DCM/AcOH/TFE (8 : 1 : 1, *v : v : v*) for 1.5 hour at room temperature, with the process repeated twice. *(note: if cleavage is incomplete at this stage, an additional treatment of DCM/HFIP (4 : 1, v/v) for 0.5–1 hour is recommended)*. The cleavage solution was collected, combined and concentrated under reduced pressure. The residue was co-evaporated with *n*-hexane (4 × 50 mL) to remove any residual acetic acid, yielding the crude protected peptide bearing the free carboxylic acid at the C-terminus as white to off-white solid. The crude compound was further dried under vacuum overnight before proceeding to macrocyclization.

Crude protected linear peptide (1 eq.) was dissolved in anhydrous DCM (0.2 mM) and cooled in an ice bath. A mixture of DIPEA (12 eq.), 1-hydroxy-7-azabenzotriazole (HOAt; 6 eq.) and ethyl cyano(hydroxyimino)acetate (OxymaPure; 6 eq.) in anhydrous DCM/DMF was added to the reaction solution, and the contents were stirred at 0°C for 10 min. Then, 1-[bis(dimethylamino)methylene]-*H*-1,2,3-triazolo[4,5-b]pyridinium 3-oxide hexafluoro-phosphate (HATU; 10 eq.) was added in one-portion, and the solution was slowly warmed to room temperature and stirred for 20 h. After, the solvent was removed under reduced pressure and the cyclic peptide was further dried under vacuum.

A freshly prepared global cleavage cocktail (TFA : TIPS : H_2O = 92.5 : 5 : 2.5, *v : v : v*) was added to the crude cyclic peptide residue obtained from the previous step at 0°C, and the resulting mixture was warmed up and vigorously stirred at room temperature for another 2 ~ 3 hours. The solution was concentrated and the crude peptide was precipitated with ice-cold diethyl ether. The macrocyclic thiopeptide precursor was dried under vacuum overnight to remove residual solvent.

General procedure for oxidative elimination. **Method 1**. The crude macrocyclic thiopeptide precursor was dissolved in dimethyl sulfoxide (DMSO; 0.1 volume of the final reaction solution) and diluted with 40% acetonitrile in phosphate buffer (pH 8; 1 mM). Hydrogen peroxide (70 wt.% in water) was then added to achieve a final concentration of 100 to 500 mM, and the reaction was shaken at room temperature or 37°C until completion, as determined by LC/MS (section 1). Upon full conversion, the solution was acidified with 2% AcOH in acetonitrile/water, filtered, purified by preparative RP-HPLC, and lyophilized to yield the final thiopeptide as a white solid.

General procedure for oxidative elimination. **Method 2**. Sodium periodate was suspended in phosphate buffer (100 mM phosphate, pH 8; ca. 25–80 mM $NaIO_4$, freshly prepared), and the mixture was briefly sonicated. Next, a solution of a macrocyclic

thiopeptide precursor in DMSO (0.4 volume of the sodium periodate solution) was added at room temperature. The reaction mixture was agitated at room temperature or 37°C on an orbital shaker until the oxidation reaction was complete, as determined by LC/MS (section 1). The suspension was diluted with 1% AcOH in acetonitrile to a final concentration of 40% (*v/v*). The solution slightly sonicated and filtered, purified by preparative RP-HPLC, and lyophilized to yield the final thiopeptide as a white solid.

5.2. Total Synthesis of wTP3

Step1: Synthesis of a linear wTP3 precursor by Fmoc SPPS

Following the above general procedure (section 5.1), a fully protected linear wTP3 precursor was obtained as a white solid. The crude linear precursor was used in the following macrocyclization without further purification. (The deprotected linear wTP3 precursor was analyzed by LC/MS, refer to section 1 for a detailed analysis method.)

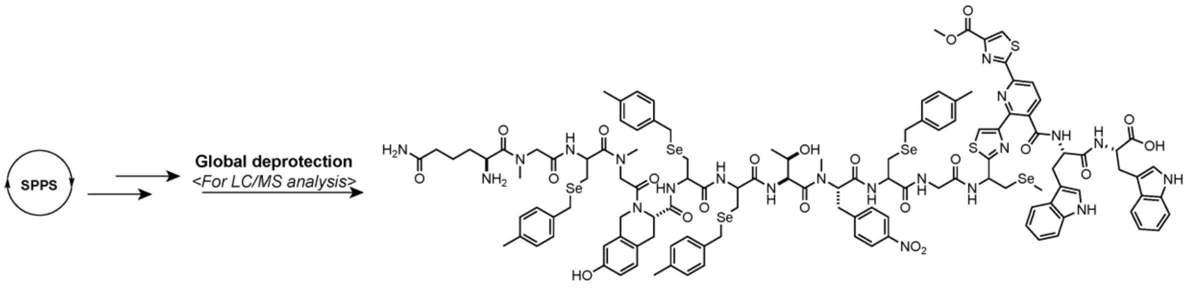

wTP3 (Linear precursor)

TIC chromatogram

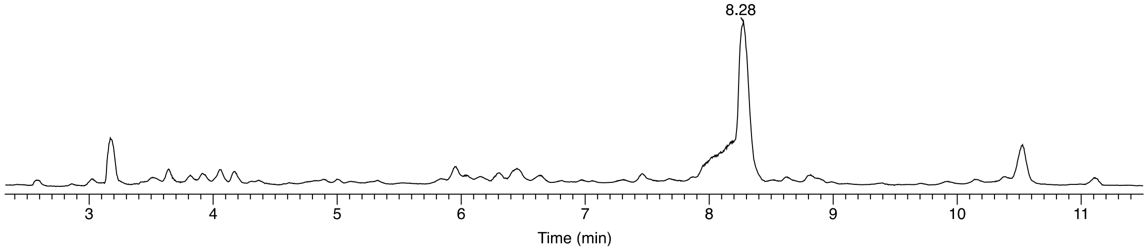

Combined MS
integrated over product peaks

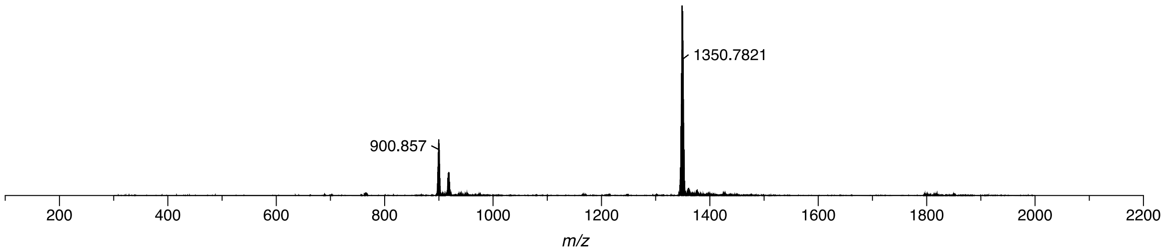

Figure S48. TIC chromatogram and mass spectra from LC/MS analysis of deprotected linear wTP3 precursor after the SPPS using method 1. ESI-MS (*m/z*): calcd for $C_{121}H_{137}N_{21}O_{22}S_2Se_5$ $[M+2H]^{2+}$ *m/z* = 1350.7836, found 1350.7821; $[M+3H]^{3+}$ *m/z* = 900.8583, found 900.8570.

Step 2: Macrocyclization and oxidative elimination to yield wTP3

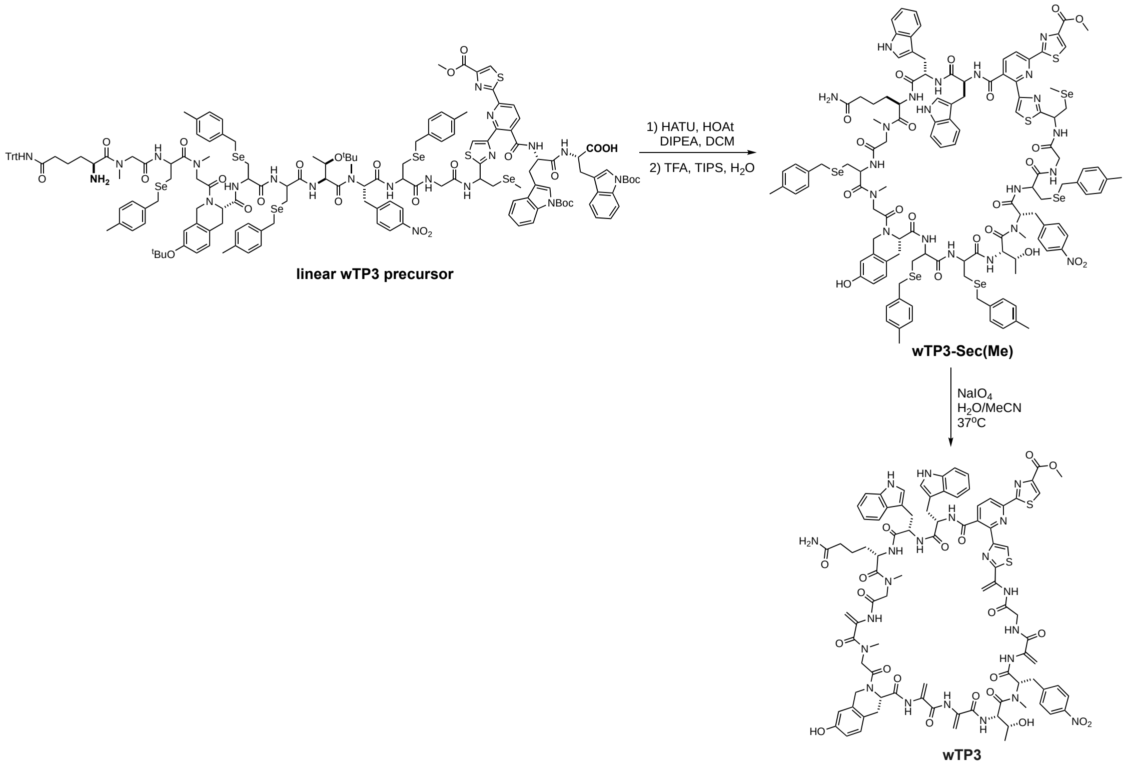

After the in-solution peptide macrocyclization and global deprotection (refer to section 5.1), the crude cyclic precursor wTP3-Sec(Me) was precipitated and dried under vacuum. The crude cyclic precursor was subsequently utilized in the final oxidative elimination without purification, following the general procedure outlined in section 5.1. The reaction employed 80 mM $NaIO_4$ as the oxidizing agent at 37°C.

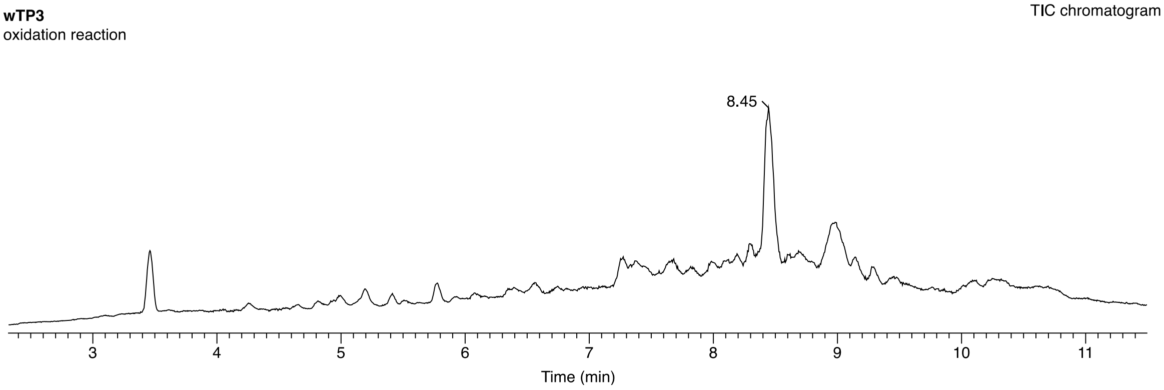

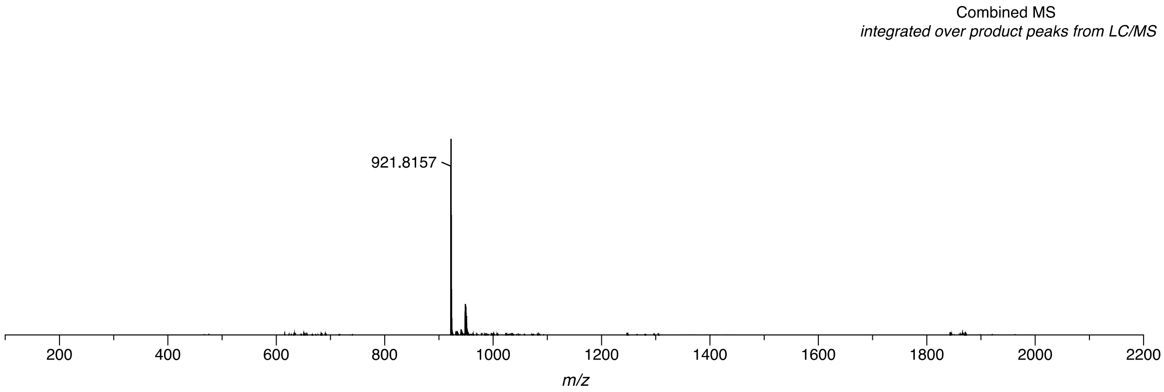

Figure S49. TIC chromatogram and mass spectra from LC/MS analysis of crude oxidation reaction of wTP3 using method 2. ESI-MS (*m/z*): calcd for $C_{88}H_{91}N_{21}O_{21}S_2$ $[M+2H]^{2+}$ *m/z* = 921.8148, found 921.8157.

Final purified wTP3

The final product was purified by RP-HPLC using the HPLC method as described in general remarks (section 1) and lyophilized as a white solid (8 mg, 5% overall yield based on the resin loading). The purity was determined to be 98% by analytical UPLC analysis.

^{1}H NMR (400 MHz, DMSO-D_6) δ 10.88 – 10.58 (m, 2H), 9.77 – 9.43 (m, 1H), 9.43 – 9.18 (m, 4H), 9.01 – 8.98 (m, 1H), 8.79 – 8.52 (m, 3H), 8.21 – 8.02 (m, 2H), 7.98 (d, *J* = 8.2 Hz, 2H), 7.92 – 7.76 (m, 2H), 7.61 – 7.48 (m, 2H), 7.39 (d, *J* = 8.4 Hz, 2H), 7.32 – 7.24 (m, 2H), 7.17 (s, 1H), 7.08 – 6.96 (m, 4H), 6.95 – 6.85 (m, 3H), 6.70 (s, 1H), 6.65 – 6.46 (m, 2H), 5.99 (s, 1H), 5.84 (s, 1H), 5.79 – 5.60 (m, 2H), 5.56 (s, 1H), 5.52 – 5.20 (m, 5H), 4.99 – 4.81 (m, 1H), 4.74 – 4.18 (m, 10H), 4.06 – 3.90 (m, 3H), 3.86 (s, 3H), 3.13 – 2.82 (m, 13H), 2.80 – 2.60 (m, 4H), 1.97 (s, 3H), 1.62 – 1.34 (m, 5H), 0.96 (s, 3H), 0.84 – 0.55 (m, 2H).

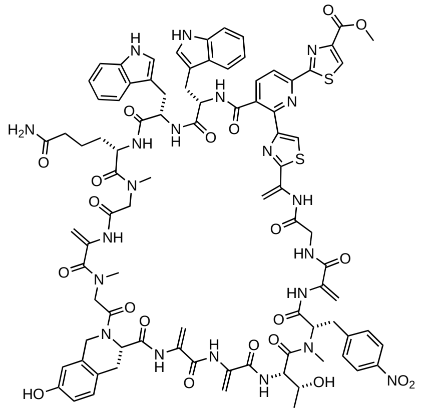

wTP3

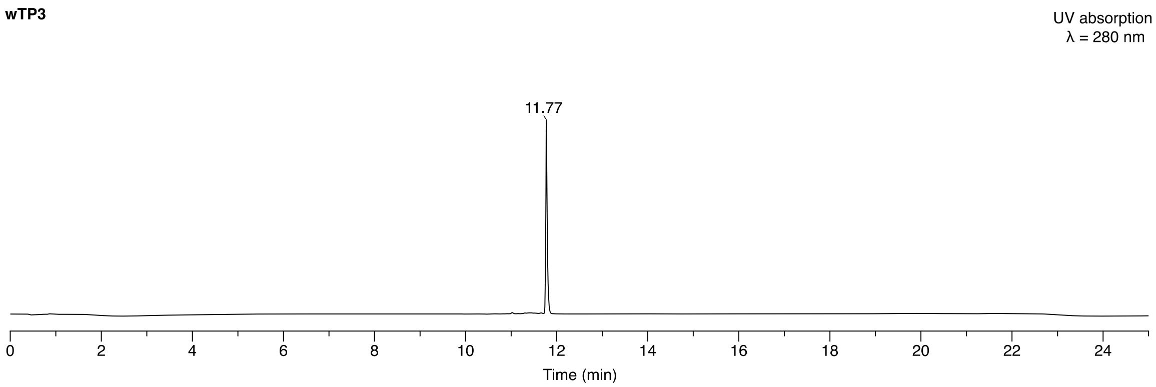

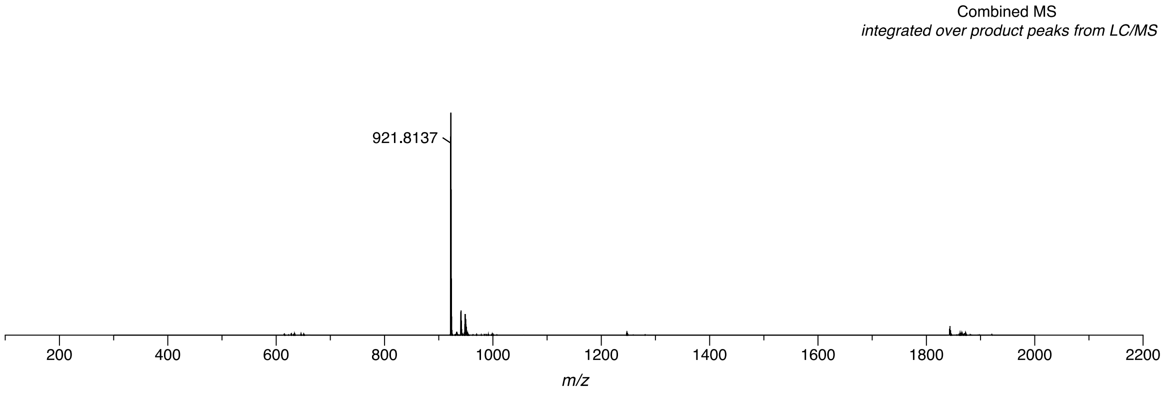

Figure S50. UV chromatogram and mass spectra of the purified wTP3. The product was analyzed by UPLC (λ = 280 nm) and LC/MS (method 2), see section 1 for details. ESI-MS (*m/z*): calcd for wTP3: $C_{88}H_{91}N_{21}O_{21}S_2$ $[M+2H]^{2+}$ *m/z* = 921.8148, found 921.8137.

5.3. Total Synthesis of Other Discovered Thiopeptides

1. Synthesis of hTP4

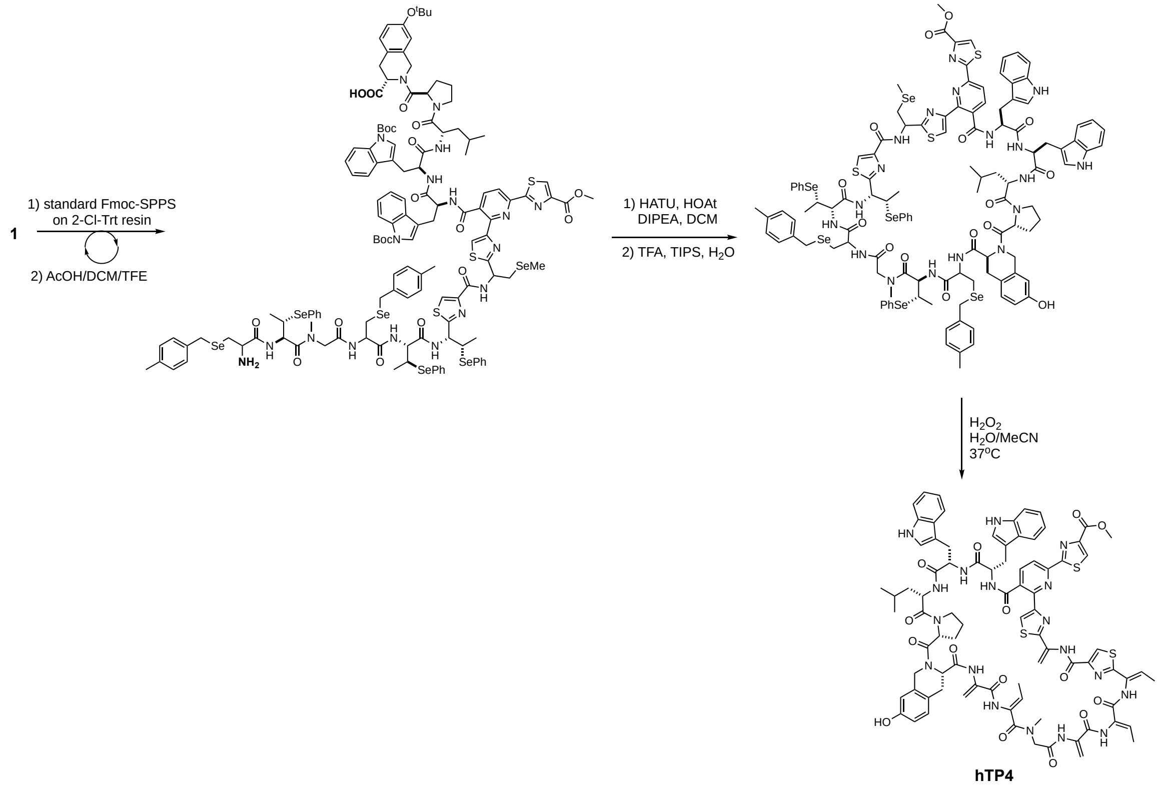

The title thiopeptide was synthesized following the general procedures described above (section 5.1). The oxidative elimination was performed at 37°C using 300 mM H_2O_2 or at

room temperature using 50 mM $NaIO_4$. The final product was purified by RP-HPLC using the HPLC method as described in general remarks and lyophilized as a white solid (1 mg, 2% overall yield based on the resin loading). The purity was determined to be 96% by analytical UPLC analysis.

^{1}H NMR (400 MHz, DMSO-D_6) δ 10.89 – 10.75 (m, 2H), 10.42 (s, 1H), 9.54 (s, 2H), 9.41 – 9.13 (m, 4H), 8.85 (s, 2H), 8.66 (d, *J* = 14.7 Hz, 2H), 8.28 (s, 1H), 8.08 (d, *J* = 8.2 Hz, 2H), 7.44 (d, *J* = 7.8 Hz, 2H), 7.38 – 7.23 (m, 4H), 7.10 – 6.84 (m, 8H), 6.74 – 6.55 (m, 4H), 6.50 (s, 2H), 6.31 – 6.22 (m, 2H), 5.70 (s, 1H), 5.63 – 5.42 (m, 4H), 5.27 (s, 2H), 4.94 – 4.71 (m, 4H), 4.48 (s, 5H), 4.15 (d, *J* = 12 Hz, 2H), 3.86 (s, 3H), 3.03 – 2.81 (m, 8H), 2.63 (s, 1H), 2.29 (s, 1H), 1.67 (d, *J* = 7.0 Hz, 3H), 1.47 (s, 3H), 0.85 – 0.76 (m, 2H), 0.65 – 0.39 (m, 5H).

Final purified hTP4

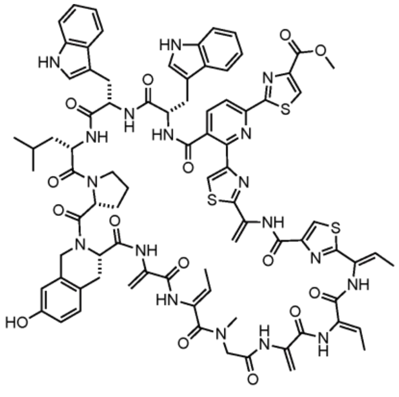

hTP4

hTP4

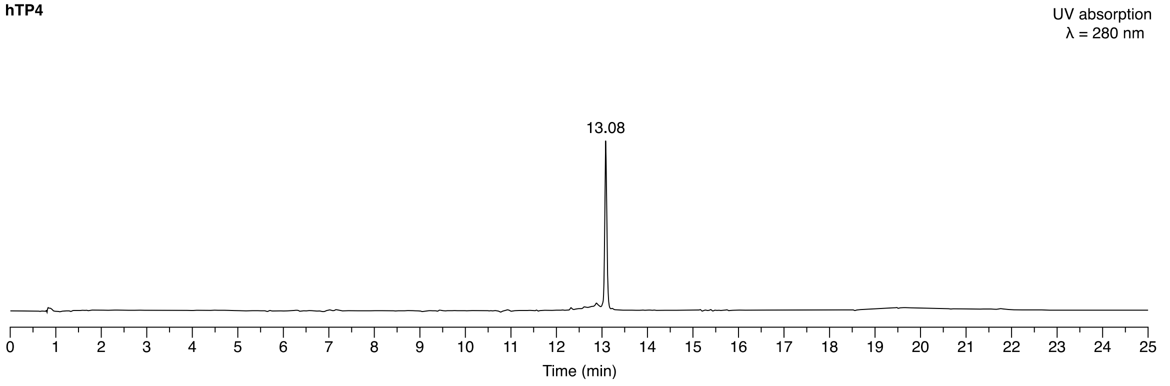

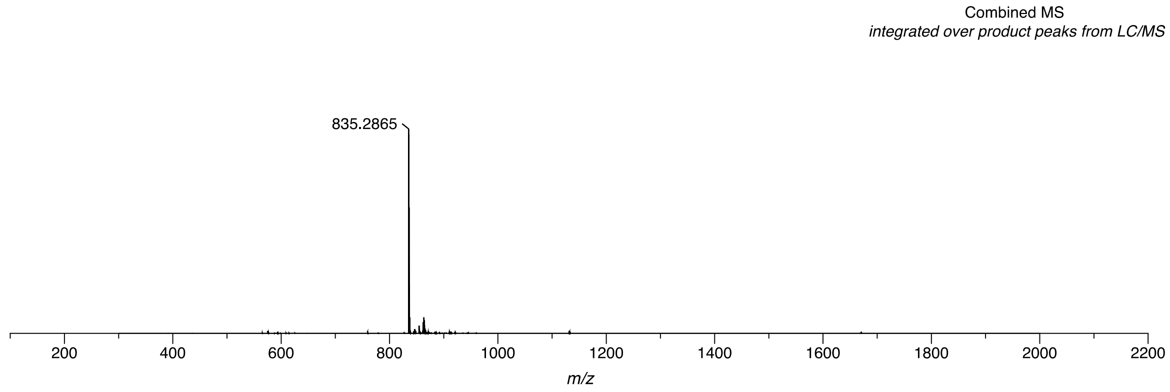

Figure S51. UV chromatogram and mass spectra of the purified hTP4. The product was analyzed by UPLC (λ = 280 nm) and LC/MS (method 2), see section 1 for details. ESI-MS (*m/z*): calcd for hTP4: $C_{83}H_{84}N_{18}O_{15}S_3$ $[M+2H]^{2+}$ *m/z* = 835.2841, found 835.2865.

2. Synthesis of hTP6

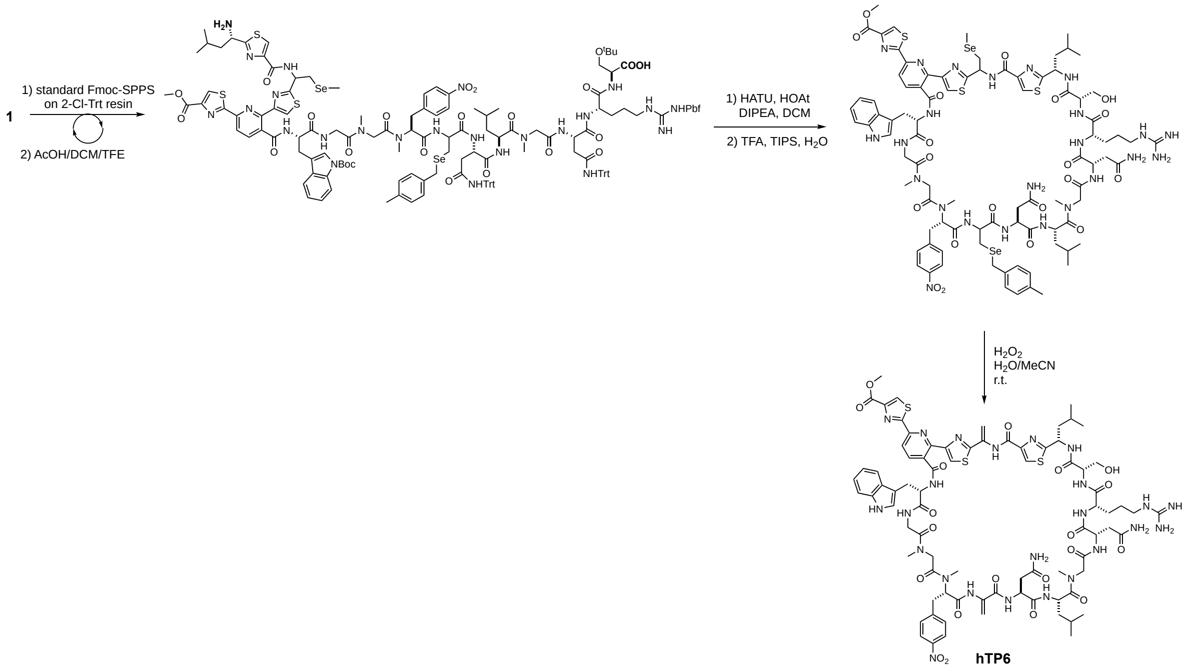

The title thiopeptide was synthesized following the general procedures described above (section 5.1). The oxidative elimination was performed at room temperature using 100 mM H_2O_2 as the oxidizing agent. The final product was purified by RP-HPLC using the HPLC method as described in general remarks and lyophilized as a white solid (5.6 mg, 12% overall yield based on the resin loading). The purity was determined to be 99% by analytical UPLC analysis.

^{1}H NMR (400 MHz, DMSO-D_6) δ 10.86 – 10.73 (m, 1H), 10.23 – 10.05 (m, 1H), 9.39 – 9.16 (m, 1H), 8.88 – 8.69 (m, 1H), 8.67 – 8.61 (m, 1H), 8.54 – 8.40 (m, 2H), 8.33 (s, 1H), 8.21 – 8.08 (m, 4H), 8.03 (s, 1H), 7.90 (m, 2H), 7.82 – 7.65 (m, 2H), 7.65 – 7.49 (m, 4H), 7.44 – 7.23 (m, 4H), 7.16 – 7.01 (m, 4H), 6.98 – 6.81 (m, 3H), 6.71 (s, 1H), 6.39 – 6.24 (m, 1H), 6.10 – 5.90 (m, 1H), 5.51 (d, *J* = 10.5 Hz, 2H), 5.30 (s, 1H), 5.09 – 4.95 (m, 2H), 4.84 – 4.67 (m, 2H), 4.64 – 4.41 (m, 3H), 4.30 – 4.15 (m, 3H), 4.11 – 3.91 (m, 3H), 3.85 (s, 3H), 3.82 – 3.75 (m, 1H), 3.63 (s, 3H), 3.20 – 3.11 (m, 2H), 3.02 (d, *J* = 6.5 Hz, 3H), 2.91 (m, 2H), 2.90 – 2.81 (m, 3H), 2.80 – 2.76 (m, 1H), 2.73 – 2.65 (m, 2H), 2.62 – 2.52 (m, 3H), 2.38 – 2.36 (m, 1H), 1.81 – 1.67 (m, 2H), 1.66 – 1.43 (m, 6H), 1.37 (s, 3H), 0.80 (q, *J* = 6.4 Hz, 4H), 0.76 – 0.64 (m, 2H), 0.56 – 0.26 (m, 6H).

Final purified hTP6

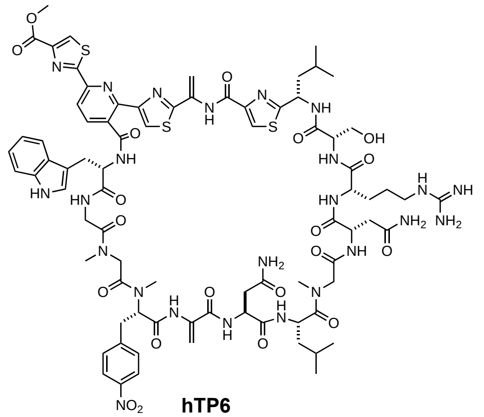

hTP6

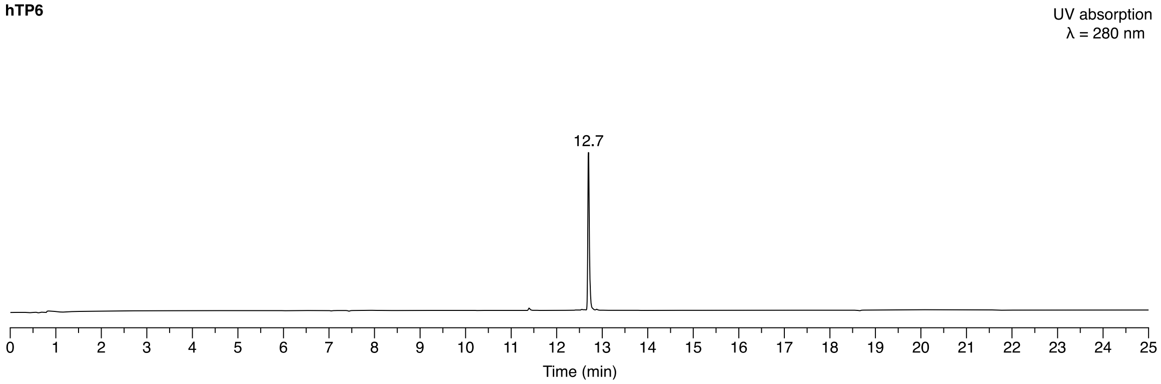

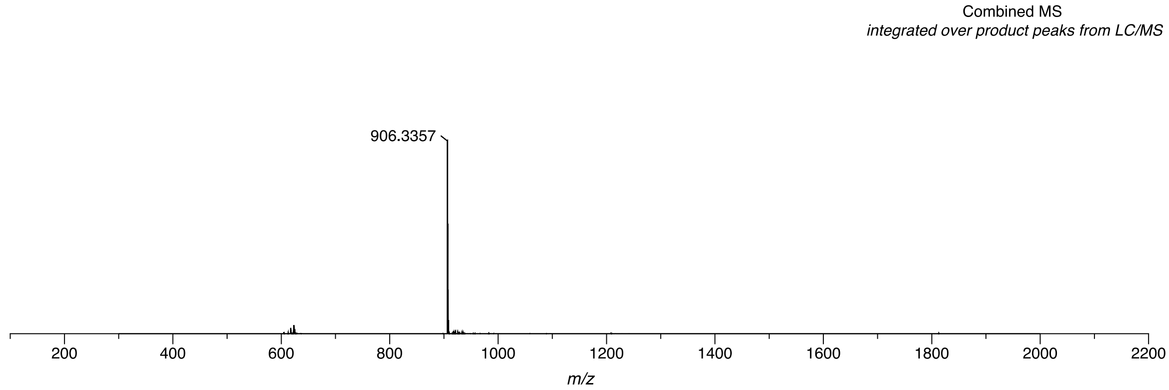

Figure S52. UV chromatogram and mass spectra of the purified hTP6. The product was analyzed by UPLC (λ = 280 nm) and LC/MS (method 2), see section 1 for details. ESI-MS (*m/z*): calcd for hTP6: $C_{80}H_{98}N_{24}O_{20}S_3$ $[M+2H]^{2+}$ *m/z* = 906.3354, found 906.3357.

3. Synthesis of hTP8

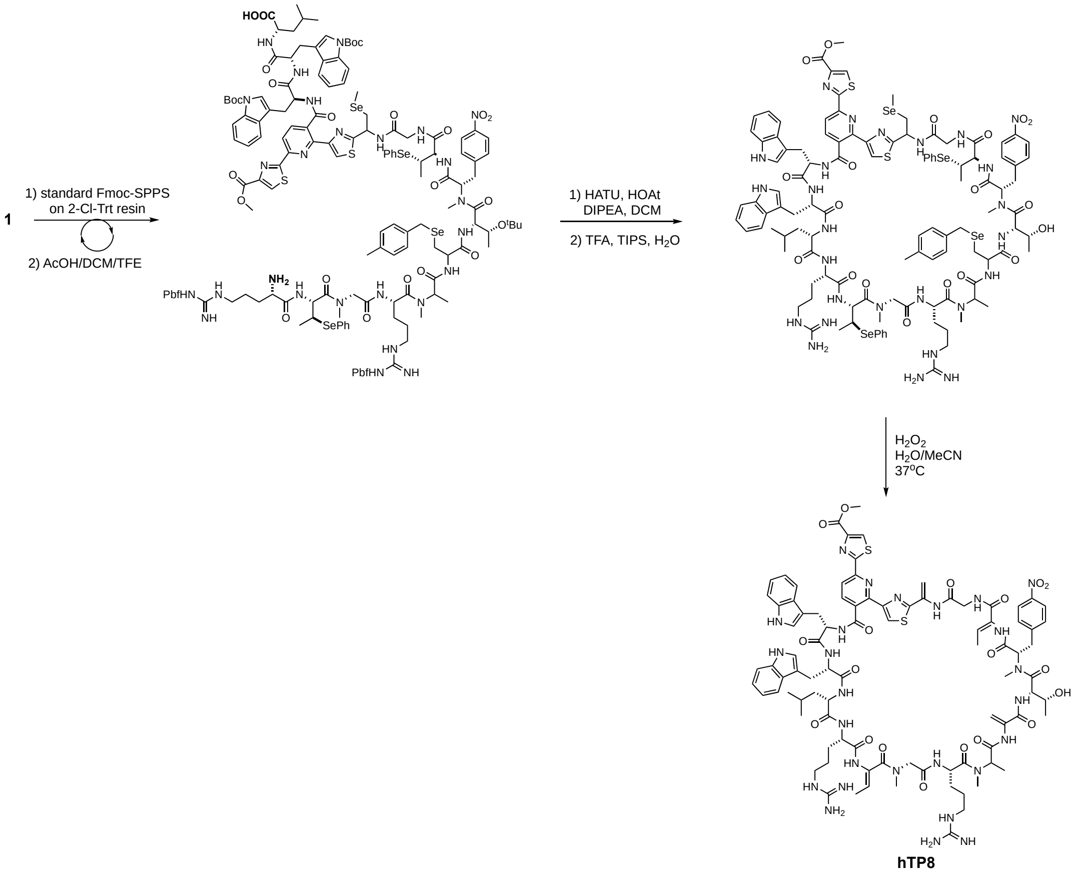

The title thiopeptide was synthesized following the general procedures described above (section 5.1). The oxidative elimination was performed at 37°C using 250 mM H_2O_2 as the oxidizing agent. The final product was purified by RP-HPLC using the HPLC method as described in general remarks and lyophilized as a white solid (5.4 mg, 5% overall yield based on the resin loading). The purity was determined to be 95% by analytical UPLC analysis.

^{1}H NMR (400 MHz, DMSO-D_6) δ 10.92 – 10.64 (m, 2H), 9.68 (S, 1H), 9.42 – 9.11 (m, 2H), 9.10 – 8.89 (m, 2H), 8.70 (s, 2H), 8.26 – 7.62 (m, 10H), 7.50 – 7.30 (m, 7H), 7.14 (s, 4H), 7.02 (t, J = 7.1 Hz, 4H), 6.98 – 6.81 (m, 5H), 6.36 (s, 2H), 5.49 (s, 4H), 5.26 – 5.06 (m, 2H), 4.90 – 4.17 (m, 10H), 4.02 (brs, 2H), 3.87 (s, 3H), 3.78 – 3.72 (m, 2H), 3.17 – 2.91 (m, 11H), 2.90 – 2.81 (m, 3H), 6.04 – 5.85 (m, 2H), 2.76 – 2.61 (m, 3H), 1.77 – 1.34 (m, 14 H), 1.32 – 1.22 (m, 4H), 1.12 – 0.78 (m, 4H), 0.72 – 0.41 (m, 6H).

Final purified hTP8

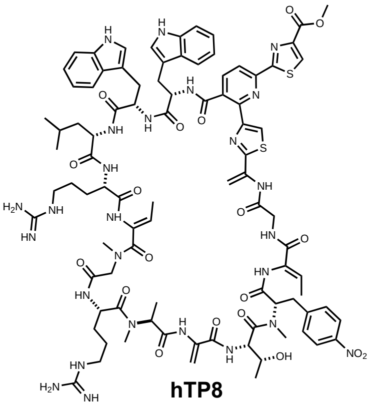

hTP8

hTP8

UV absorption
λ = 280 nm

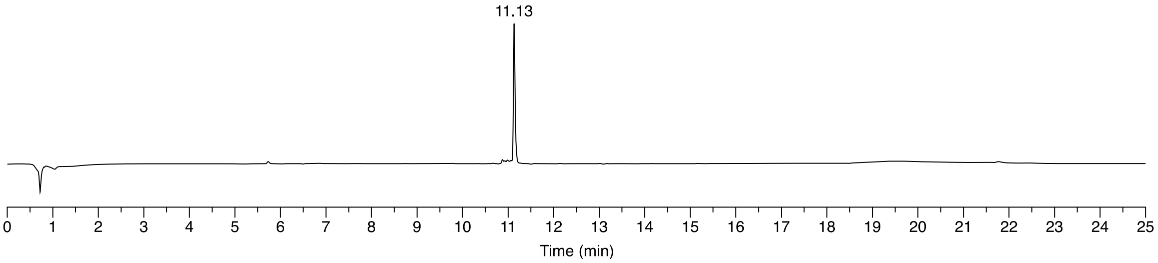

Combined MS
integrated over product peaks from LC/MS

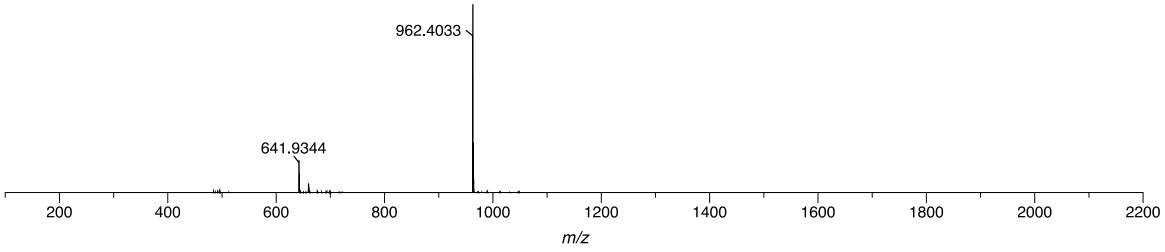

Figure S53. UV chromatogram and mass spectra of the purified hTP8. The product was analyzed by UPLC (λ = 280 nm) and LC/MS (method 2), see section 1 for details. ESI-MS (*m/z*): calcd for hTP8: $C_{90}H_{110}N_{26}O_{19}S_2$ $[M+2H]^{2+}$ *m/z* = 962.4019, found 962.4033; $[M+3H]^{3+}$ *m/z* = 641.9372, found 641.9344.

4. Synthesis of hTP13

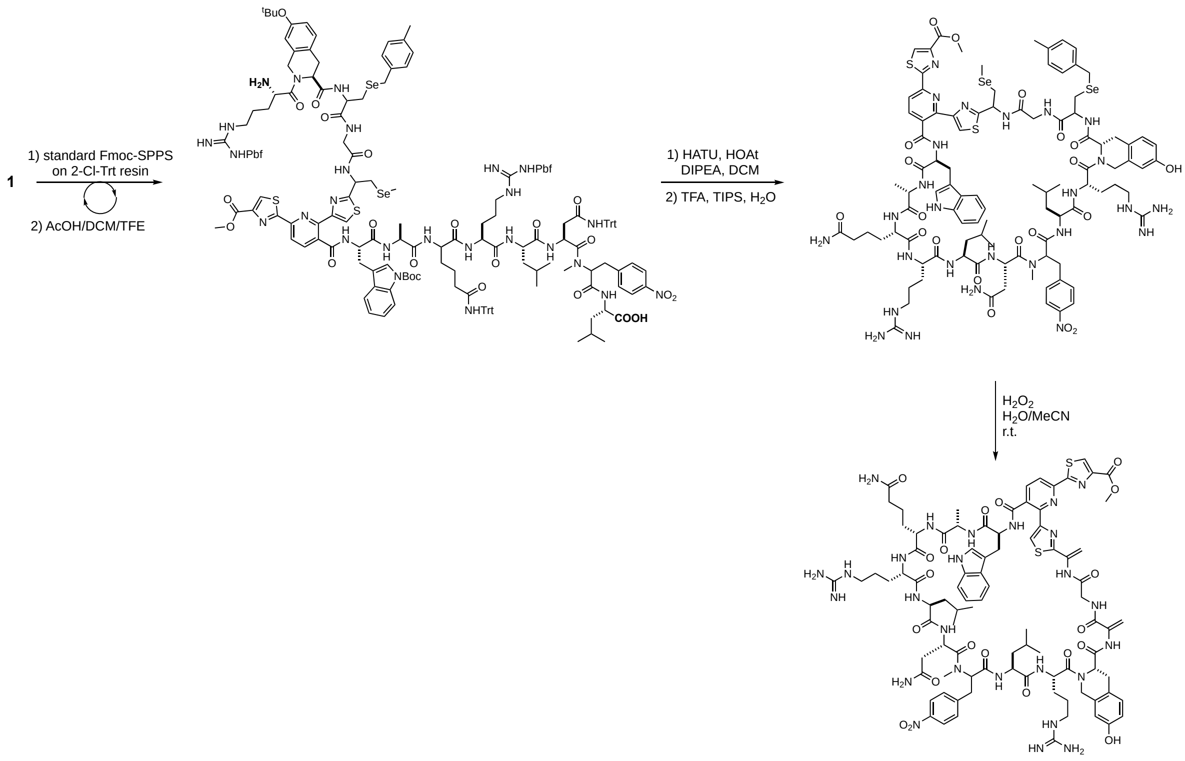

hTP13

The title thiopeptide was synthesized following the general procedures described above (section 5.1). The oxidative elimination was performed at room temperature using 100 mM H_2O_2 as the oxidizing agent. The final product was purified by RP-HPLC using the HPLC method as described in general remarks and lyophilized as a white solid (2 mg, 4% overall yield based on the resin loading). The purity was determined to be 96% by analytical UPLC analysis.

^{1}H NMR (400 MHz, DMSO-D_6) δ 10.75 (s, 1H), 9.42 – 9.28 (m, 2H), 9.26 – 9.11 (m, 2H), 8.77 – 8.64 (m, 2H), 8.47 – 8.27 (m, 3H), 8.19 (s, 1H), 8.06 (m, 4H), 7.91 – 7.76 (m, 2H), 7.65 – 7.51 (m, 5H), 7.40 (d, *J* = 8.6 Hz, 1H), 7.31 – 7.28 (m, 1H), 7.26 – 7.18 (m, 2H), 7.15 (s, 1H), 7.08 (t, *J* = 3.5 Hz, 1H), 7.02 (d, *J* = 7.4 Hz, 2H), 6.93 (m, 3H), 6.85 (d, *J* = 8.0 Hz, 1H), 6.72 (d, *J* = 13.6 Hz, 2H), 6.65 (s, 1H), 6.62 – 6.53 (m, 1H), 6.50 (d, *J* = 7.8 Hz, 1H), 6.14 (s, 1H), 5.93 (s, 1H), 5.62 – 5.49 (m, 2H), 5.25 (s, 1H), 5.14 – 4.95 (m, 2H), 4.93 – 4.71 (m, 5H), 4.59 – 4.46 (m, 1H), 4.43 – 4.26 (m, 3H), 4.26 – 4.16 (m, 2H), 4.05 – 3.93 (m, 2H), 3.86 (s, 3H), 3.16 – 2.95 (m, 9H), 2.90 (d, *J* = 10.2 Hz, 2H), 2.81 (s, 1H), 2.70 (s, 1H), 2.67 – 2.62 (m, 1H), 2.31 – 2.23 (m, 1H), 2.04 – 1.91 (m, 3H), 1.64 – 1.49 (m, 7H), 1.47 – 1.33 (m, 7H), 1.22 – 1.17 (m, 3H), 1.14 (d, *J* = 6.8 Hz, 2H), 0.84 – 0.66 (m, 13H).

Final purified hTP13

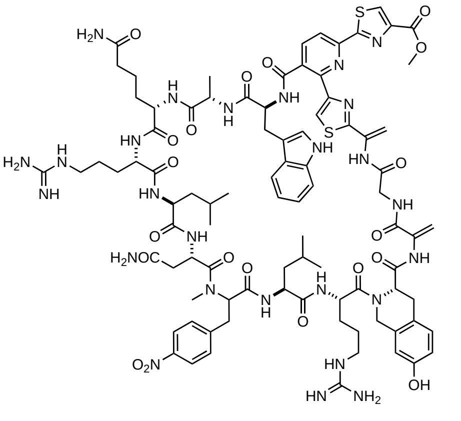

hTP13

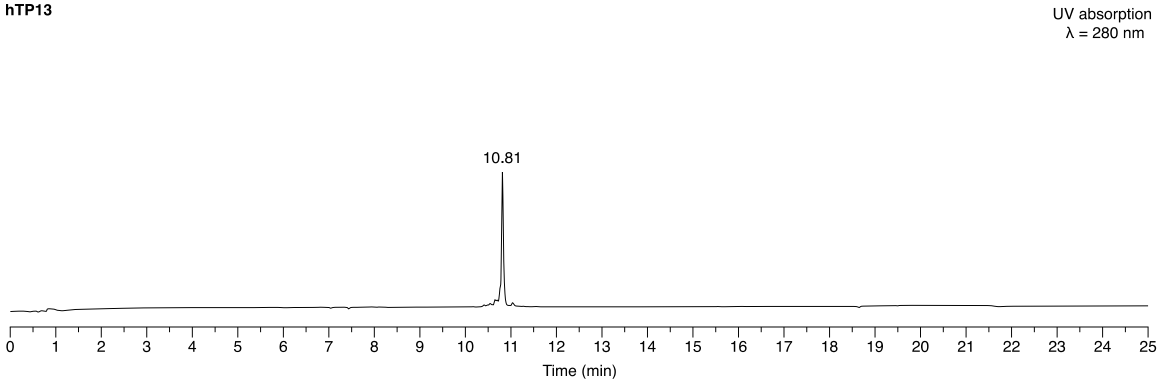

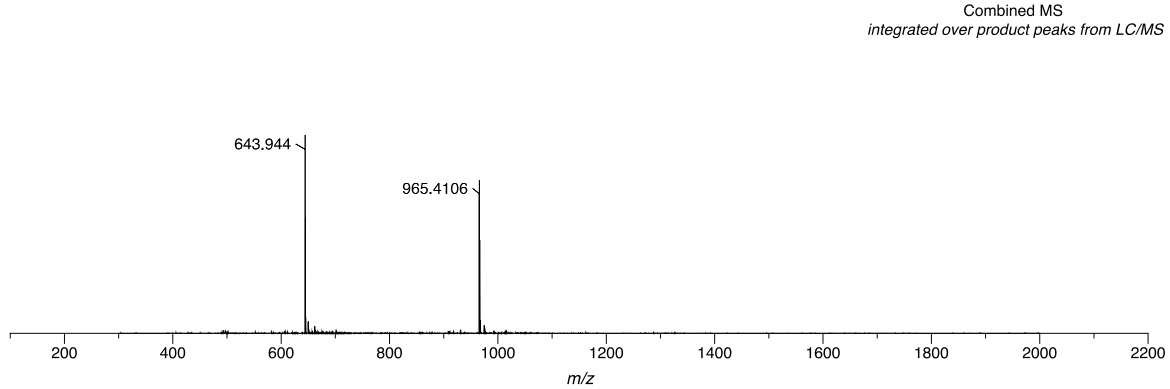

Figure S54. UV chromatogram and mass spectra of the purified hTP13. The product was analyzed by UPLC (λ = 280 nm) and LC/MS (method 2), see section 1 for details. ESI-MS (*m/z*): calcd for hTP13: $C_{89}H_{112}N_{26}O_{20}S_2$ $[M+2H]^{2+}$ *m/z* = 965.4072, found 965.4106; $[M+3H]^{3+}$ *m/z* = 643.9407, found 643.9440.

5. Synthesis of hTP15

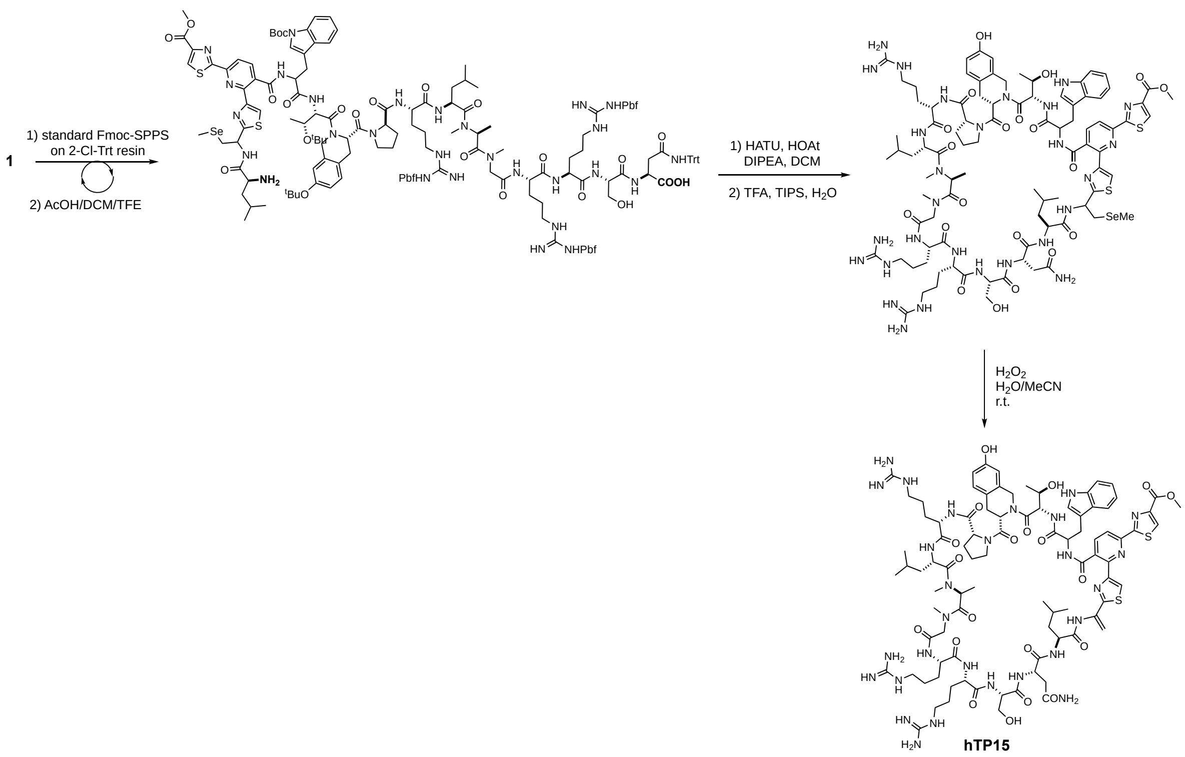

The title thiopeptide was synthesized following the general procedures described above (section 5.1). The oxidative elimination was performed at room temperature using 100 mM H_2O_2 as the oxidizing agent. The final product was purified by RP-HPLC using the HPLC method as described in general remarks and lyophilized as a white solid (1.5 mg, 3% overall yield based on the resin loading). The purity was determined to be 98% by analytical UPLC analysis.

hTP15

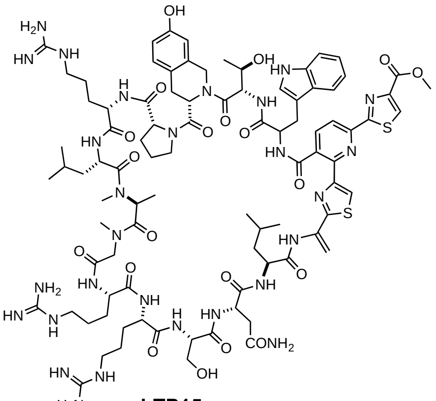

hTP15

hTP15

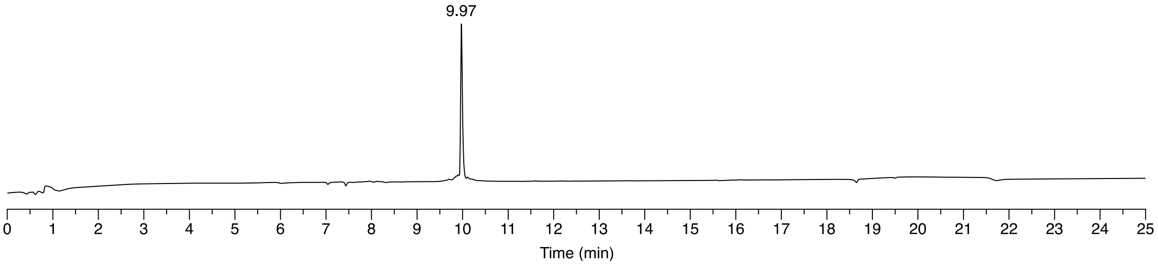

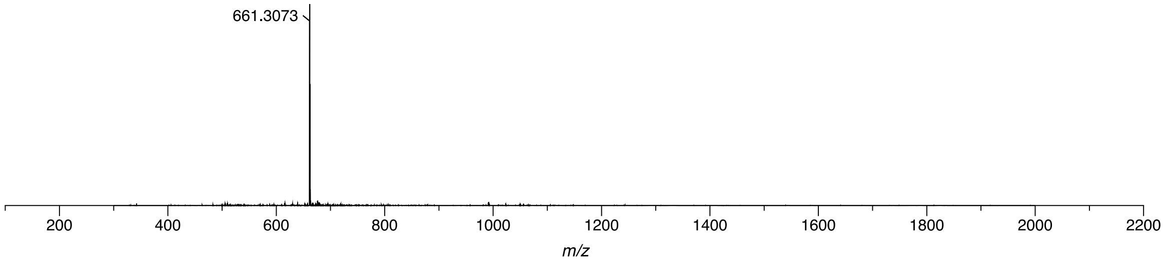

Figure S55. UV chromatogram and mass spectra of the purified hTP15. The product was analyzed by UPLC (λ = 280 nm) and LC/MS (method 2), see section 1 for details. ESI-MS (*m/z*): calcd for hTP15: $C_{90}H_{124}N_{28}O_{20}S_2$ $[M+3H]^{3+}$ *m/z* = 661.3074, found 661.3073.

6. Synthesis of wTP4

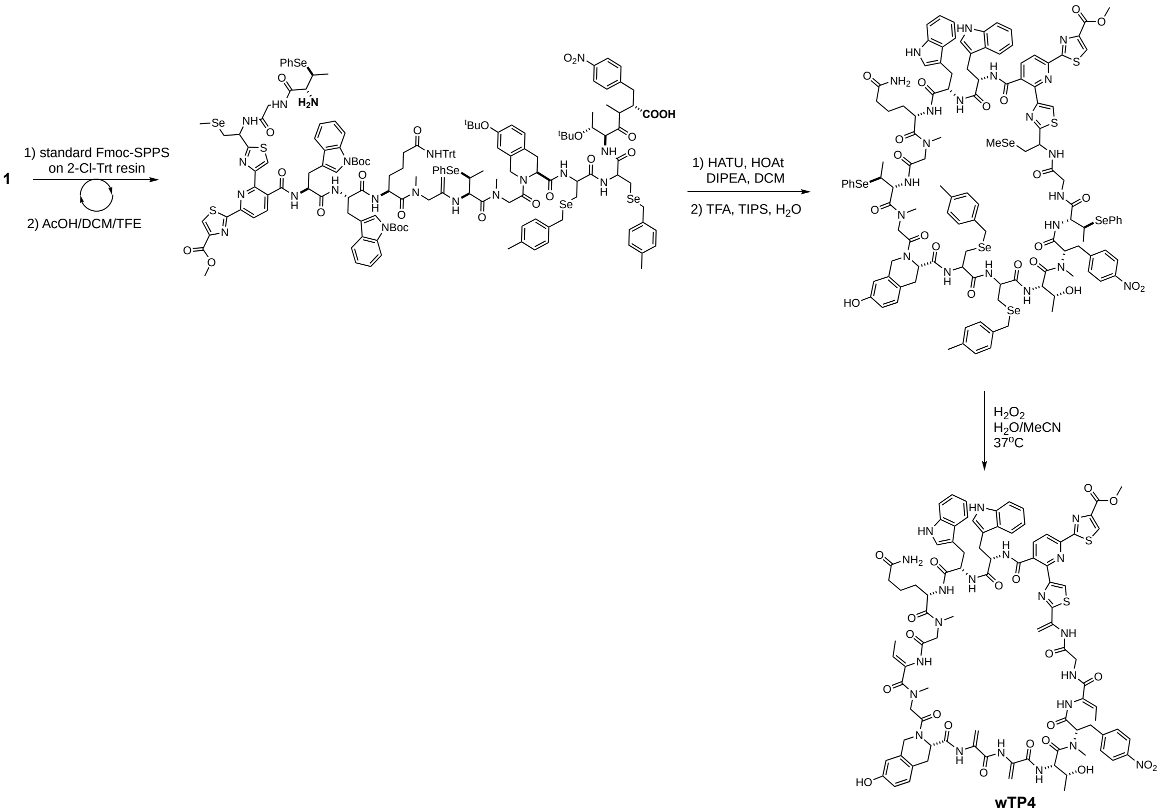

The title thiopeptide was synthesized following the general procedures described above (section 5.1). The oxidative elimination was performed at 37°C using 200 mM H_2O_2 as the oxidizing agent. The final product was purified by RP-HPLC using the HPLC method as described in general remarks and lyophilized as a white solid (1.9 mg, 6% overall yield based on the resin loading). The purity was determined to be 94% by analytical UPLC analysis.

Final purified wTP4

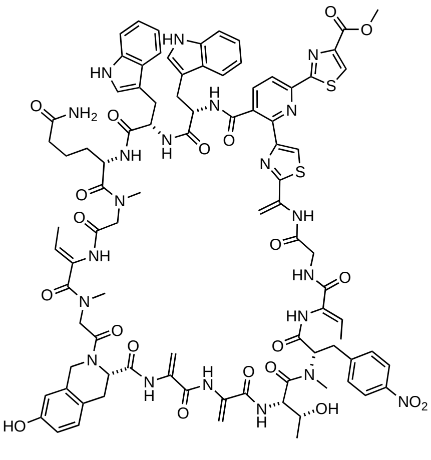

wTP4

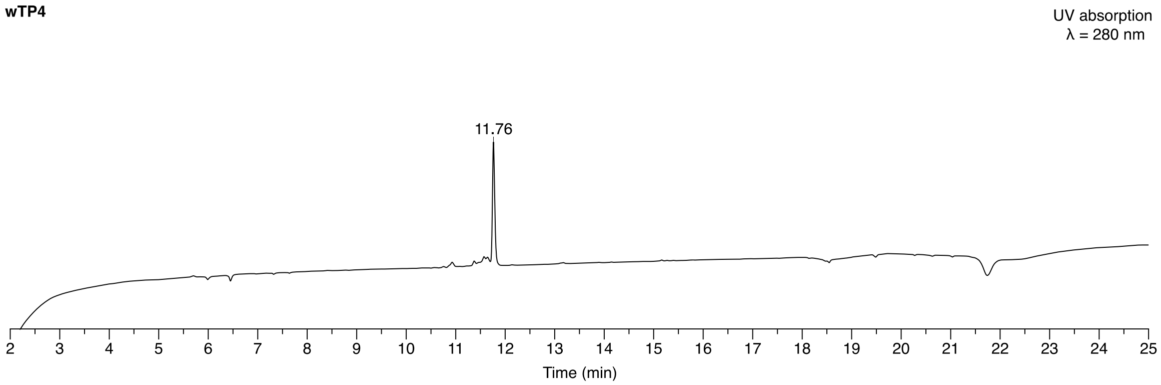

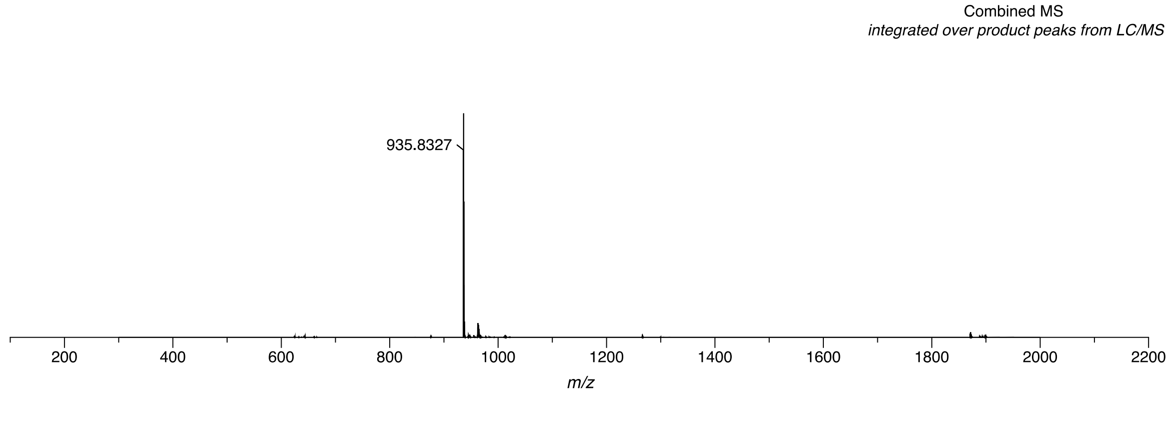

Figure S56. UV chromatogram and mass spectra of the purified wTP4. The product was analyzed by UPLC (λ = 280 nm) and LC/MS (method 2), see section 1 for details. ESI-MS (*m/z*): calcd for wTP4: $C_{90}H_{95}N_{21}O_{21}S_2$ $[M+2H]^{2+}$ *m/z* = 935.8306, found 935.8327.

7. Synthesis of wTP8

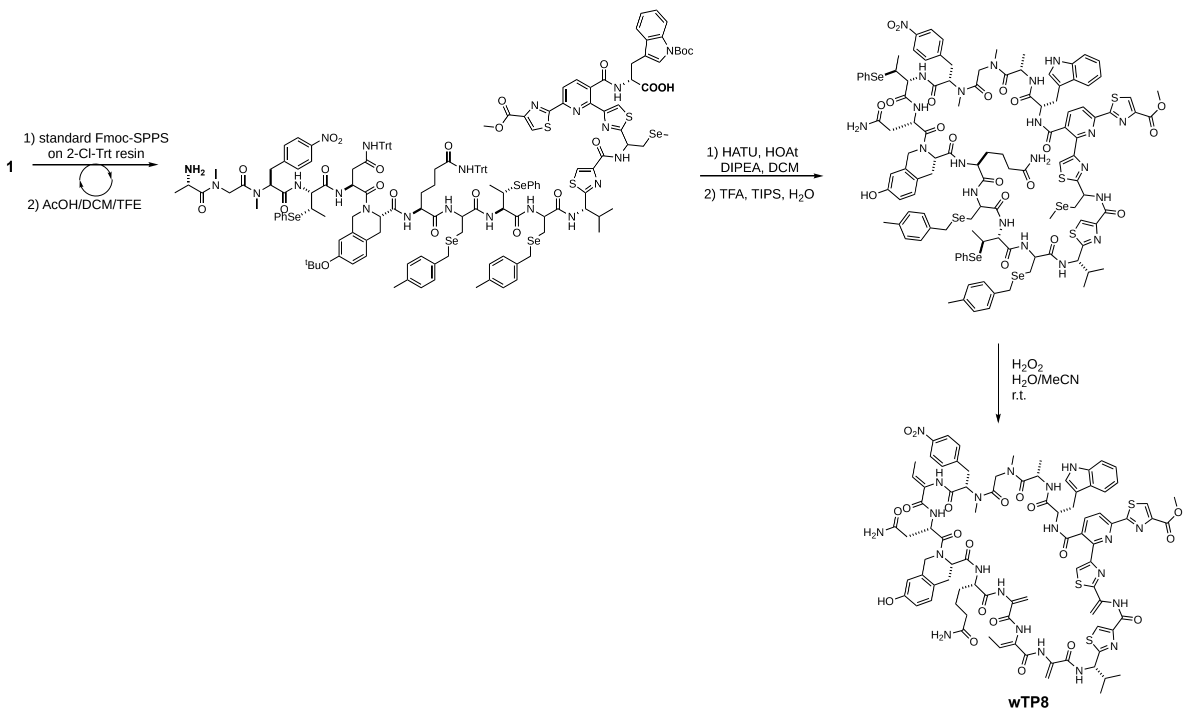

The title thiopeptide was synthesized following the general procedures described above (section 5.1). The oxidative elimination was performed at room temperature using 500 mM H_2O_2 as the oxidizing agent. The final product was purified by RP-HPLC using the HPLC method as described in general remarks and lyophilized as a white solid (0.7 mg, 3% overall yield based on the resin loading). The purity was determined to be 95% by analytical UPLC analysis.

Final purified wTP8

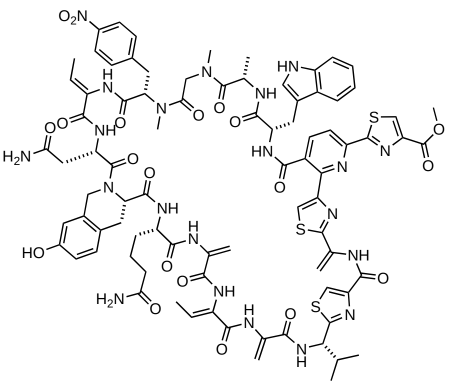

wTP8

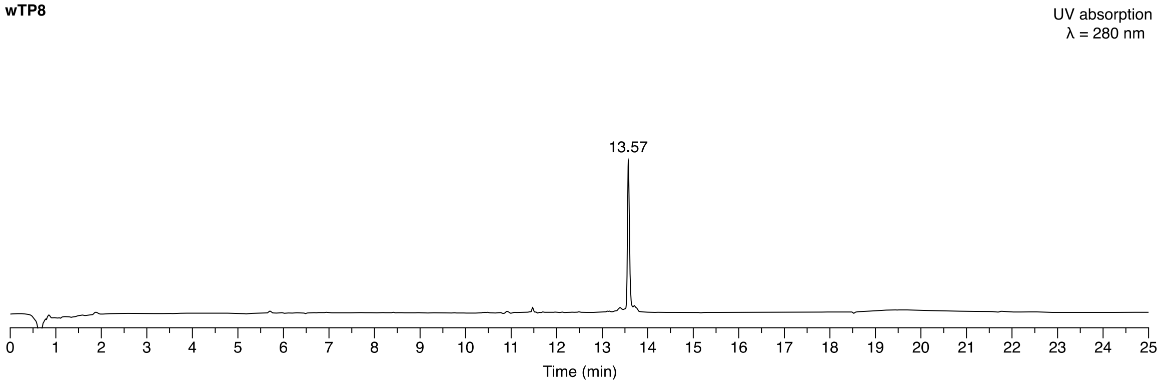

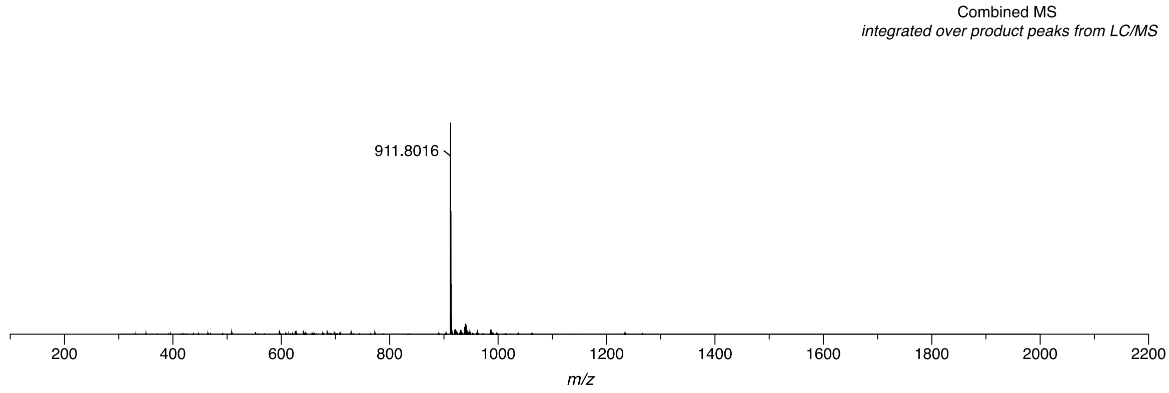

Figure S57. UV chromatogram and mass spectra of the purified wTP8. The product was analyzed by UPLC (λ = 280 nm) and LC/MS (method 2), see section 1 for details. ESI-MS (*m/z*): calcd for wTP8: $C_{85}H_{91}N_{21}O_{20}S_3$ $[M+2H]^{2+}$ *m/z* = 911.8034, found 911.8016.

8. Synthesis of wTP12

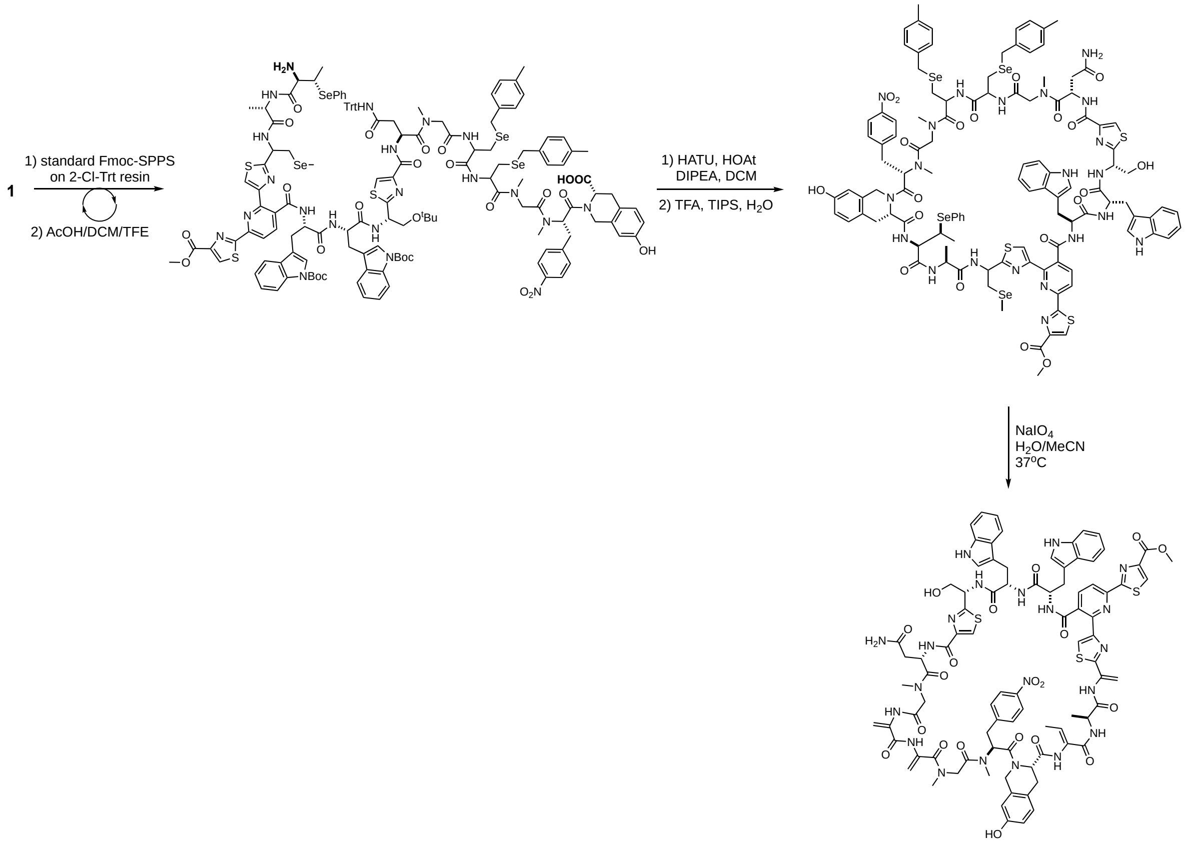

wTP12

The title thiopeptide was synthesized following the general procedures described above (section 5.1). The oxidative elimination was performed at 37°C using 50mM $NaIO_4$ as the oxidizing agent. The final product was purified by RP-HPLC using the HPLC method as described in general remarks and lyophilized as a white solid (2.4 mg, 5% overall yield based on the resin loading). The purity was determined to be 96% by analytical UPLC analysis.

^{1}H NMR (400 MHz, DMSO-D_6) δ 10.85 – 10.64 (m, 2H), 9.91 (s, 1H), 9.37 – 9.09 (m, 3H), 8.68 (s, 2H), 8.57 (s, 1H), 8.20 – 7.94 (m, 6H), 7.79 (s, 1H), 7.64 – 7.21 (m, 9H), 7.10 – 6.97 (m, 4H), 6.96 – 6.84 (m, 4H), 6.62 – 6.30 (m, 4H), 5.90 – 5.78 (m, 1H), 5.61 – 5.44 (m, 2H), 5.44 – 5.33 (m, 1H), 5.25 – 5.16 (m, 1H), 5.09 – 5.01 (m, 1H), 4.75 – 4.63 (m, 3H), 4.44 – 4.24 (m, 4H), 4.22 – 4.03 (m, 3H), 3.86 (s, 3H), 3.68 (s, 3H), 3.18 – 3.05 (m, 5H), 3.05 – 2.72 (m, 10H), 2.64 (d, J = 8.7 Hz, 5H), 2.51 (s, 2H), 1.39 – 1.30 (m, 3H), 1.16 – 1.06 (m, 2H), 0.81 (d, J = 6.5 Hz, 1H).

Final purified wTP12

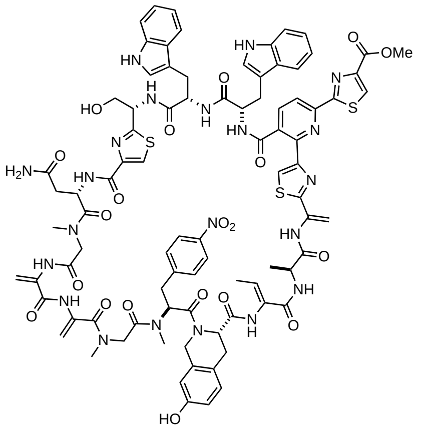

wTP12

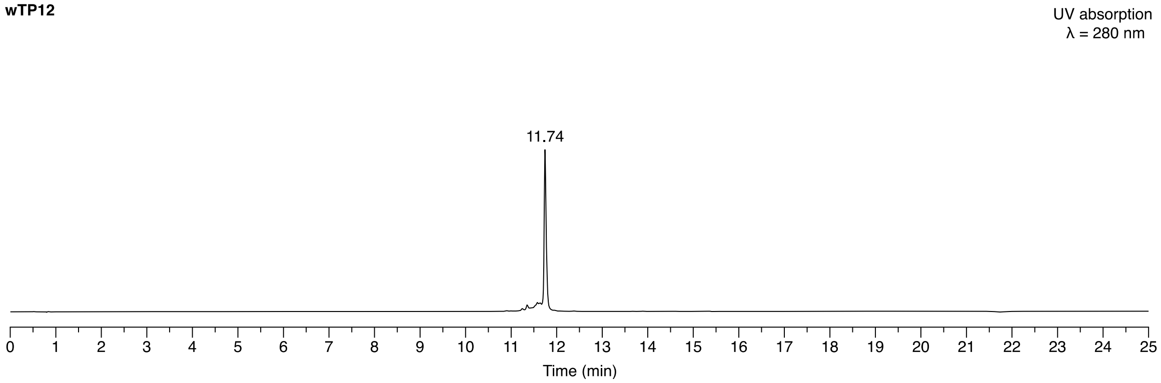

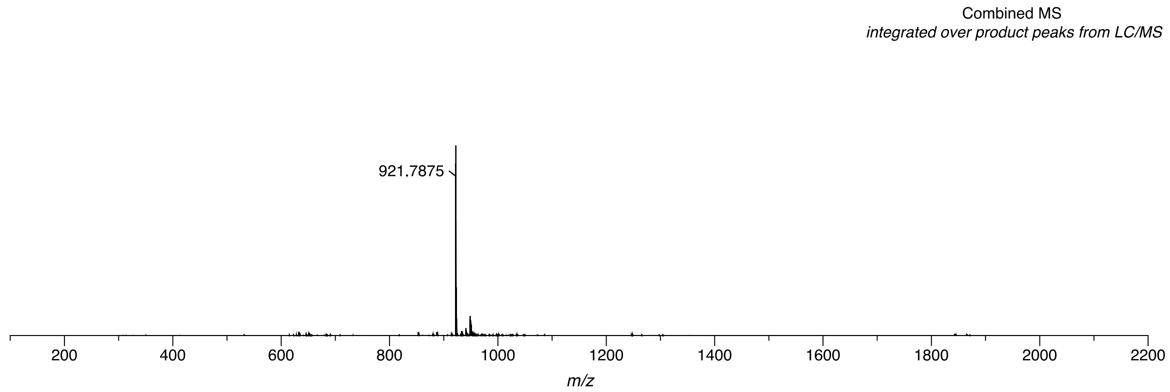

Figure S58. UV chromatogram and mass spectra of the purified wTP12. The product was analyzed by UPLC (λ = 280 nm) and LC/MS (method 2), see section 1 for details. ESI-MS (*m/z*): calcd for wTP12: $C_{87}H_{87}N_{21}O_{20}S_3$ $[M+2H]^{2+}$ *m/z* = 921.7877, found 921.7875.

9. Synthesis of wTP13

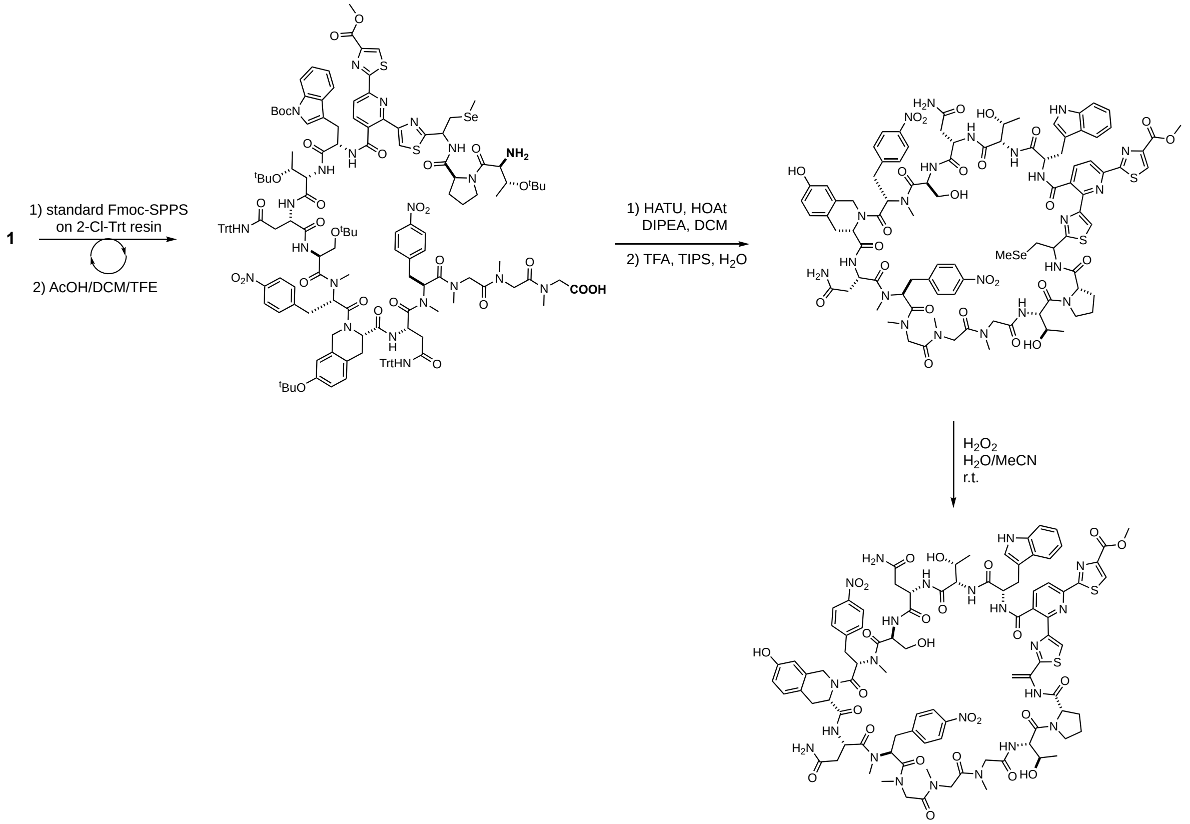

wTP13

The title thiopeptide was synthesized following the general procedures described above (section 5.1). The oxidative elimination was performed at room temperature using 100mM H_2O_2 as the oxidizing agent. The final product was purified by RP-HPLC using the HPLC method as described in general remarks and lyophilized as a white solid (1.6 mg, 3% overall yield based on the resin loading). The purity was determined to be 93% by analytical UPLC analysis.

Purified wTP13

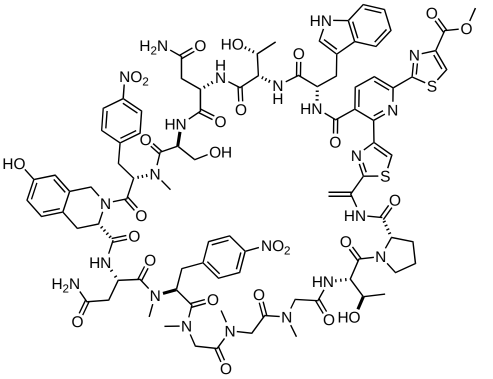

wTP13

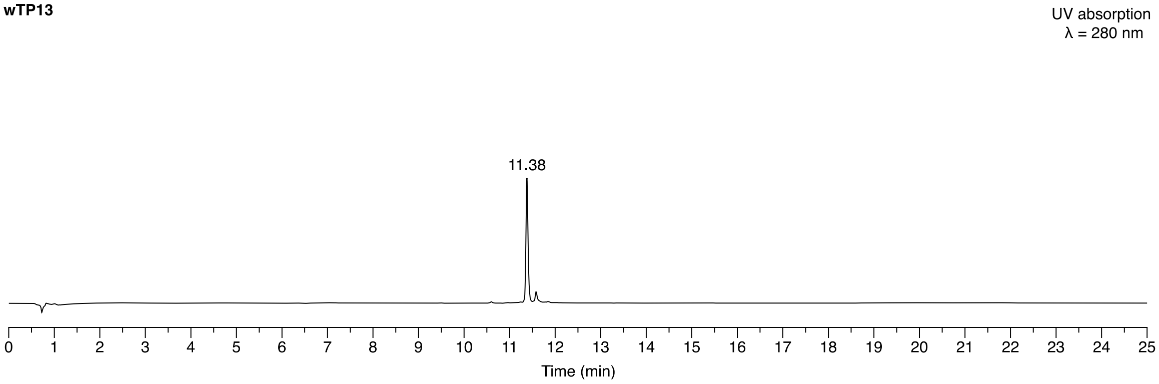

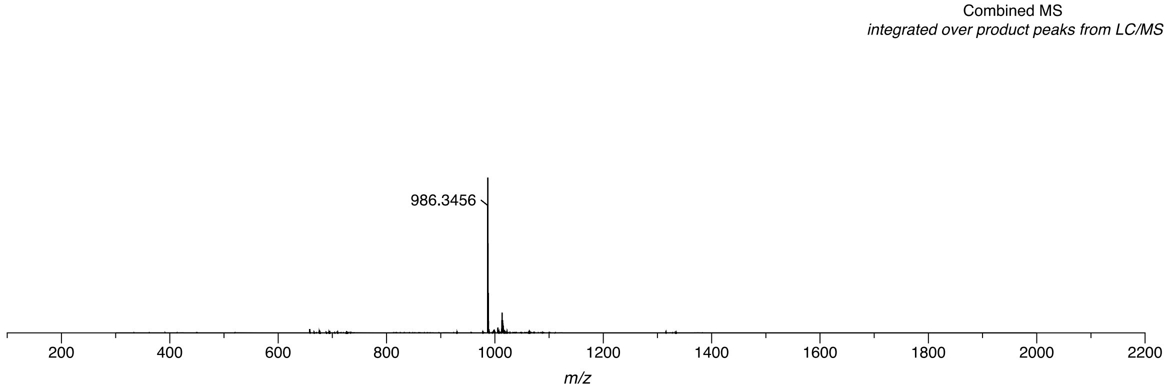

Figure S59. UV chromatogram and mass spectra of the purified wTP13. The product was analyzed by UPLC (λ = 280 nm) and LC/MS (method 2), see section 1 for details. ESI-MS (*m/z*): calcd for wTP13: $C_{90}H_{102}N_{22}O_{26}S_2$ [M+2H]$^{2+}$ *m/z* = 986.3467, found 986.3456.

6. Supplementary References

(1) Zhang, Y.; Vinogradov, A. A.; Chang, J. S.; Goto, Y.; Suga, H. Solid-Phase-Based Synthesis of Lactazole-Like Thiopeptides. Org. Lett. **2022**, 24, 7894–7899.

(2) Vinogradov, A. A.; Shimomura, M.; Goto, Y.; Sugai, Y.; Suga, H.; Onaka, H. Minimal Lactazole Scaffold for in Vitro Thiopeptide Bioengineering. Nat. Commun. **2020**, 11, 2272.

(3) Vinogradov, A. A.; Nagai, E.; Chang, J. S.; Narumi, K.; Onaka, H.; Goto, Y.; Suga, H. Accurate Broadcasting of Substrate Fitness for Lactazole Biosynthetic Pathway from Reactivity-Profiling mRNA Display. J. Am. Chem. Soc. **2020**, 142, 20329–20334.

(4) Vinogradov, A. A.; Nagano, M.; Goto, Y.; Suga, H. Site-Specific Nonenzymatic Peptide S/O-Glutamylation Reveals the Extent of Substrate Promiscuity in Glutamate Elimination Domains. J. Am. Chem. Soc. **2021**, 143, 13358–13369.

(5) Kawakami, T.; Murakami, H.; Suga, H. Messenger RNA-Programmed Incorporation of Multiple N-Methyl-Amino Acids into Linear and Cyclic Peptides. Chem. Biol. **2008**, 15, 32–42.

(6) Kawakami, T.; Ishizawa, T.; Murakami, H. Extensive Reprogramming of the Genetic Code for Genetically Encoded Synthesis of Highly N-Alkylated Polycyclic Peptidomimetics. J. Am. Chem. Soc. **2013**, 135, 12297–12304.

(7) Shimizu, Y.; Inoue, A.; Tomari, Y.; Suzuki, T.; Yokogawa, T.; Nishikawa, K.; Ueda, T. Cell-Free Translation Reconstituted with Purified Components. Nat. Biotechnol. **2001**, 19, 751–755.

(8) Goto, Y.; Katoh, T.; Suga, H. Flexizymes for Genetic Code Reprogramming. Nat. Protoc. **2011**, 6, 779–790.

(9) Rogers, D.; Hahn, M. Extended-Connectivity Fingerprints. J. Chem. Inf. Model. **2010**, 50, 742–754.

(10) Vinogradov, A. A.; Chang, J. S.; Onaka, H.; Goto, Y.; Suga, H. Accurate Models of Substrate Preferences of Post-Translational Modification Enzymes from a Combination of mRNA Display and Deep Learning. ACS Cent. Sci. **2022**, 8, 814–824.

(11) Sainburg, T.; McInnes, L.; Gentner, T. Q. Parametric UMAP Embeddings for Representation and Semisupervised Learning. Neural Comput. **2021**, 33, 2881–2907.

(12) Vinogradov, A. A.; Zhang, Y.; Hamada, K.; Chang, J. S.; Okada, C.; Nishimura, H.; Terasaka, N.; Goto, Y.; Ogata, K.; Sengoku, T.; Onaka, H.; Suga, H. De Novo Discovery of Thiopeptide Pseudo-Natural Products Acting as Potent and Selective TNIK Kinase Inhibitors. J. Am. Chem. Soc. **2022**, 144, 20332–20341.

(13) Vinogradov, A. A.; Shimomura, M.; Kano, N.; Goto, Y.; Onaka, H.; Suga, H. Promiscuous Enzymes Cooperate at the Substrate Level En Route to Lactazole A. J. Am. Chem. Soc. **2020**, 142, 13886–13897.

(14) Miller, C. J.; Lou, H. J.; Simpson, C.; Van De Kooij, B.; Hak Ha, B.; Fisher, O. S.; Pirman, N. L.; Boggon, T. J.; Rinehart, J.; Yaffe, M. B.; Linding, R.; Turk, B. E. Comprehensive Profiling of the STE20 Kinase Family Defines Features Essential for Selective Substrate Targeting and Signaling Output. PLoS Biol. **2019**, 17, e2006540.

(15) Virtanen, P.; Gommers, R.; Oliphant, T. E.; Haberland, M.; Reddy, T.; Cournapeau, D.; Burovski, E.; Peterson, P.; Weckesser, W.; Bright, J.; van der Walt, S. J.; Brett, M.; Wilson, J.; Millman, K. J.; Mayorov, N.; Nelson, A. R. J.; Jones, E.; Kern, R.; Larson, E.; Carey, C. J.; Polat, İ.; Feng, Y.; Moore, E. W.; VanderPlas, J.; Laxalde, D.; Perktold, J.; Cimrman, R.; Henriksen, I.; Quintero, E. A.; Harris, C. R.; Archibald, A. M.; Ribeiro, A. H.; Pedregosa, F.; van Mulbregt, P.; Vijaykumar, A.; Bardelli, A. Pietro; Rothberg, A.; Hilboll, A.; Kloeckner, A.; Scopatz, A.; Lee, A.; Rokem, A.; Woods, C. N.; Fulton, C.; Masson, C.; Häggström, C.;

Fitzgerald, C.; Nicholson, D. A.; Hagen, D. R.; Pasechnik, D. V.; Olivetti, E.; Martin, E.; Wieser, E.; Silva, F.; Lenders, F.; Wilhelm, F.; Young, G.; Price, G. A.; Ingold, G. L.; Allen, G. E.; Lee, G. R.; Audren, H.; Probst, I.; Dietrich, J. P.; Silterra, J.; Webber, J. T.; Slavič, J.; Nothman, J.; Buchner, J.; Kulick, J.; Schönberger, J. L.; de Miranda Cardoso, J. V.; Reimer, J.; Harrington, J.; Rodríguez, J. L. C.; Nunez-Iglesias, J.; Kuczynski, J.; Tritz, K.; Thoma, M.; Newville, M.; Kümmerer, M.; Bolingbroke, M.; Tartre, M.; Pak, M.; Smith, N. J.; Nowaczyk, N.; Shebanov, N.; Pavlyk, O.; Brodtkorb, P. A.; Lee, P.; McGibbon, R. T.; Feldbauer, R.; Lewis, S.; Tygier, S.; Sievert, S.; Vigna, S.; Peterson, S.; More, S.; Pudlik, T.; Oshima, T.; Pingel, T. J.; Robitaille, T. P.; Spura, T.; Jones, T. R.; Cera, T.; Leslie, T.; Zito, T.; Krauss, T.; Upadhyay, U.; Halchenko, Y. O.; Vázquez-Baeza, Y. SciPy 1.0: Fundamental Algorithms for Scientific Computing in Python. Nat. Methods **2020**, 17, 261–272.

(16) Katoh, T.; Sengoku, T.; Hirata, K.; Ogata, K.; Suga, H. Ribosomal Synthesis and de Novo Discovery of Bioactive Foldamer Peptides Containing Cyclic β-Amino Acids. Nat. Chem. **2020**, 12, 1081–1088.

(17) Hirata, K.; Yamashita, K.; Ueno, G.; Kawano, Y.; Hasegawa, K.; Kumasaka, T.; Yamamoto, M. Zoo: An Automatic Data-Collection System for High-Throughput Structure Analysis in Protein Microcrystallography. Acta Crystallogr. Sect. D Struct. Biol. **2019**, 75, 138–150.

(18) Kabsch, W. XDS. Acta Crystallogr. Sect. D Struct. Biol. **2010**, 66, 125–132.

(19) Yamashita, K.; Hirata, K.; Yamamoto, M. KAMO: Towards Automated Data Processing for Microcrystals. Acta Crystallogr. Sect. D Struct. Biol. **2018**, 74, 441–449.

(20) McCoy, A. J.; Grosse-Kunstleve, R. W.; Adams, P. D.; Winn, M. D.; Storoni, L. C.; Read, R. J. Phaser Crystallographic Software. J. Appl. Crystallogr. **2007**, 40, 658–674.

(21) Emsley, P.; Lohkamp, B.; Scott, W. G.; Cowtan, K. Features and Development of Coot. Acta Crystallogr. Sect. D Biol. Crystallogr. **2010**, 66, 486–501.

(22) Murshudov, G. N.; Skubák, P.; Lebedev, A. A.; Pannu, N. S.; Steiner, R. A.; Nicholls, R. A.; Winn, M. D.; Long, F.; Vagin, A. A. REFMAC5 for the Refinement of Macromolecular Crystal Structures. Acta Crystallogr. Sect. D Biol. Crystallogr. **2011**, 67, 355–367.

(23) Collaborative Computational Project, N. 4. The CCP4 Suite: Programs for Protein Crystallography. Acta Crystallogr. Sect. D Biol. Crystallogr. **1994**, 50, 760–763.

(24) Long, F.; Nicholls, R. A.; Emsley, P.; Gražulis, S.; Merkys, A.; Vaitkus, A.; Murshudov, G. N. AceDRG: A Stereochemical Description Generator for Ligands. Acta Crystallogr. Sect. D Struct. Biol. **2017**, 73, 112–122.

(25) Moriarty, N. W.; Grosse-Kunstleve, R. W.; Adams, P. D. Electronic Ligand Builder and Optimization Workbench (ELBOW): A Tool for Ligand Coordinate and Restraint Generation. Acta Crystallogr. Sect. D Biol. Crystallogr. **2009**, 65, 1074–1080.

(26) Liebschner, D.; Afonine, P. V.; Baker, M. L.; Bunkoczi, G.; Chen, V. B.; Croll, T. I.; Hintze, B.; Hung, L. W.; Jain, S.; McCoy, A. J.; Moriarty, N. W.; Oeffner, R. D.; Poon, B. K.; Prisant, M. G.; Read, R. J.; Richardson, J. S.; Richardson, D. C.; Sammito, M. D.; Sobolev, O. V.; Stockwell, D. H.; Terwilliger, T. C.; Urzhumtsev, A. G.; Videau, L. L.; Williams, C. J.; Adams, P. D. Macromolecular Structure Determination Using X-Rays, Neutrons and Electrons: Recent Developments in Phenix. Acta Crystallogr. Sect. D Struct. Biol. **2019**, 75, 861–877.

(27) Masuda, M.; Uno, Y.; Ohbayashi, N.; Ohata, H.; Mimata, A.; Kukimoto-Niino, M.; Moriyama, H.; Kashimoto, S.; Inoue, T.; Goto, N.; Okamoto, K.; Shirouzu, M.; Sawa, M.; Yamada, T. TNIK Inhibition Abrogates Colorectal Cancer Stemness. Nat. Commun. **2016**, 7, 12586.

(28) Chen, C.; Ha, B. H.; Thévenin, A. F.; Lou, H. J.; Zhang, R.; Yip, K. Y.; Peterson, J. R.; Gerstein, M.; Kim, P. M.; Filippakopoulos, P.; Knapp, S.; Boggon, T. J.; Turk, B. E. Identification of a Major Determinant for Serine-Threonine Kinase Phosphoacceptor Specificity. Mol. Cell **2014**, 53, 140–147.

(29) Laskowski, R. A.; Swindells, M. B. LigPlot+: Multiple Ligand-Protein Interaction Diagrams for Drug Discovery. J. Chem. Inf. Model. **2011**, 51, 2778–2786.

(30) Zhou, H.; van der Donk, W. A. Biomimetic Stereoselective Formation of Methyllanthionine. Org. Lett. **2002**, 4, 1335–1338.

(31) Freidinger, R. M.; Hinkle, J. S.; Perlow, D. S.; Arison, B. H. Synthesis of 9-Fluorenylmethyloxycarbonyl-Protected N-Alkyl Amino Acids by Reduction of Oxazolidinones. J. Org. Chem. **1983**, 48, 77–81.

(32) Harrison, B. A.; Pasternak, G. W.; Verdine, G. L. 2,6-Dimethyltyrosine Analogues of a Stereodiversified Ligand Library: Highly Potent, Selective, Non-Peptidic μ Opioid Receptor Agonists. J. Med. Chem. **2003**, 46, 677–680.

(33) Nicolaou, K. C.; Estrada, A. A.; Zak, M.; Lee, S. H.; Safina, B. S. A Mild and Selective Method for the Hydrolysis of Esters with Trimethyltin Hydroxide. Angew. Chemie - Int. Ed. **2005**, 44, 1378–1382.

(34) Aihara, K.; Inokuma, T.; Jichu, T.; Lin, Z.; Fu, F.; Yamaoka, K.; Shigenaga, A.; Hutchins, D. A.; Schmidt, E. W.; Otaka, A. Cysteine-Free Intramolecular Ligation of N -Sulfanylethylanilide Peptide Using 4-Mercaptobenzylphosphonic Acid: Synthesis of Cyclic Peptide Trichamide. Synlett **2017**, 28, 1944–1949.

(35) Wang, C.; Han, C.; Yang, J.; Zhang, Z.; Zhao, Y.; Zhao, J. Ynamide-Mediated Thioamide and Primary Thioamide Syntheses. J. Org. Chem. **2022**, 87, 5617–5629.

(36) Wever, W. J.; Bogart, J. W.; Baccile, J. A.; Chan, A. N.; Schroeder, F. C.; Bowers, A. A. Chemoenzymatic Synthesis of Thiazolyl Peptide Natural Products Featuring an Enzyme-Catalyzed Formal [4 + 2] Cycloaddition. J. Am. Chem. Soc. **2015**, 137, 3494–3497.

(37) Bogart, J. W.; Bowers, A. A. Thiopeptide Pyridine Synthase TbtD Catalyzes an Intermolecular Formal Aza-Diels–Alder Reaction. J. Am. Chem. Soc. **2019**, 141, 1842–1846.

(38) Wang, B.; Lamattina, J. W.; Marshall, S. L.; Booker, S. J. Capturing Intermediates in the Reaction Catalyzed by NosN, a Class C Radical S-Adenosylmethionine Methylase Involved in the Biosynthesis of the Nosiheptide Side-Ring System. J. Am. Chem. Soc. **2019**, 141, 5788–5797.

7. NMR spectra

^{1}H NMR of Fmoc–N^{Me}–Phe(pNO$_2$)–OH (400 MHz, DMSO-d_6)

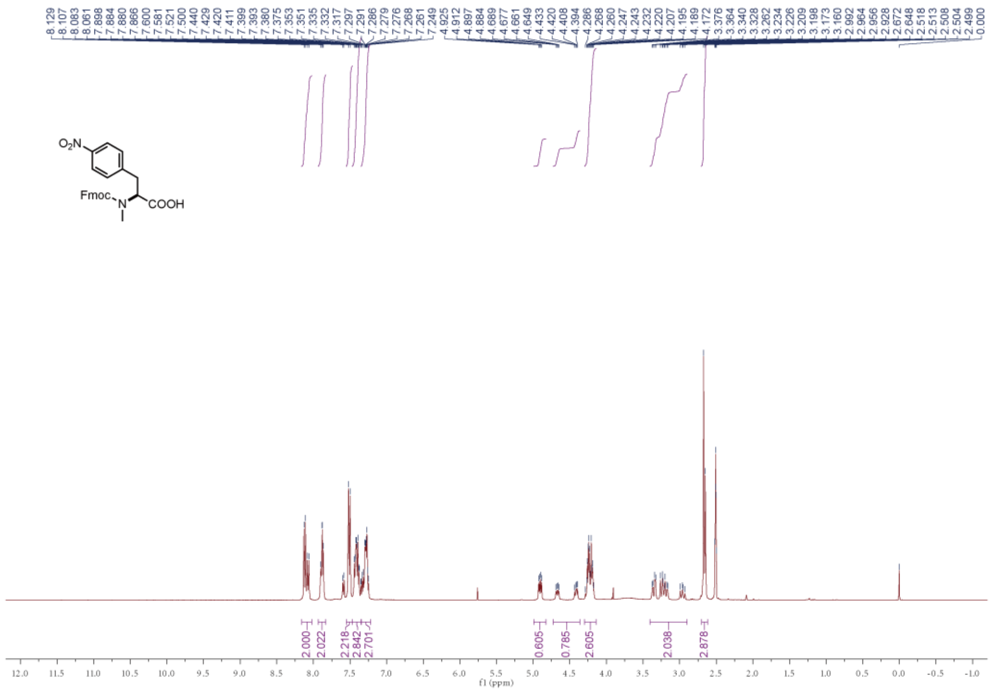

^{13}C NMR of Fmoc–N^{Me}–Phe(pNO$_2$)–OH (101 MHz, DMSO-d_6)

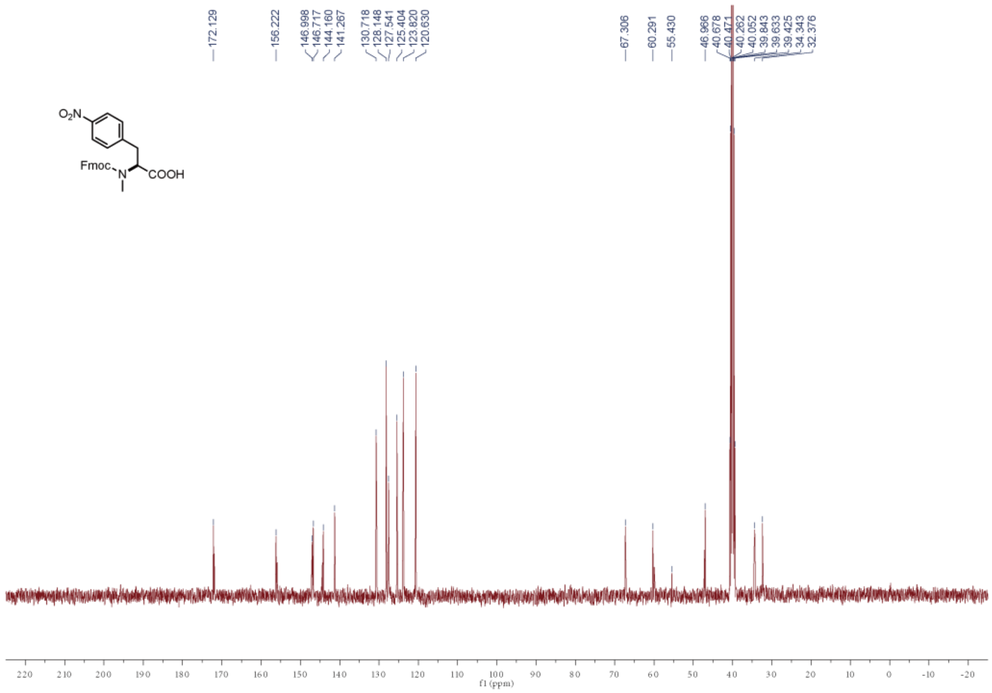

^{1}H NMR of Fmoc–Tic(pO^{t}Bu)–OMe (400 MHz, CDCl$_3$)

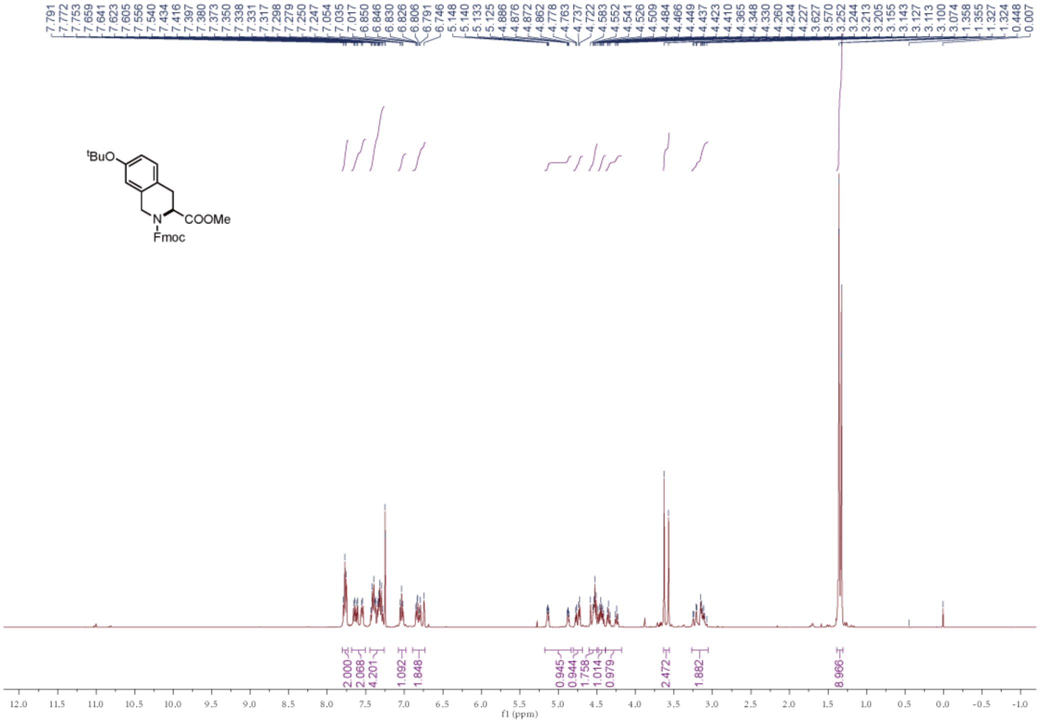

^{13}C NMR of Fmoc–Tic(pO^{t}Bu)–OMe (101 MHz, CDCl$_3$)

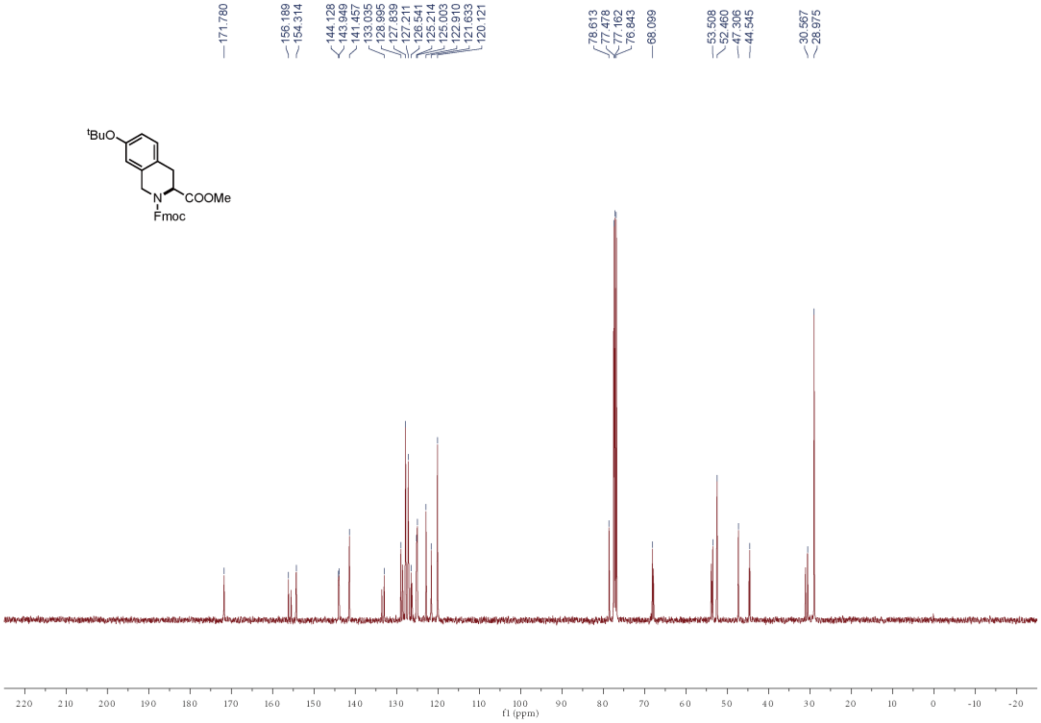

^{1}H NMR of Fmoc–Tic(*p*O^{t}Bu)–OH (400 MHz, $CDCl_3$)

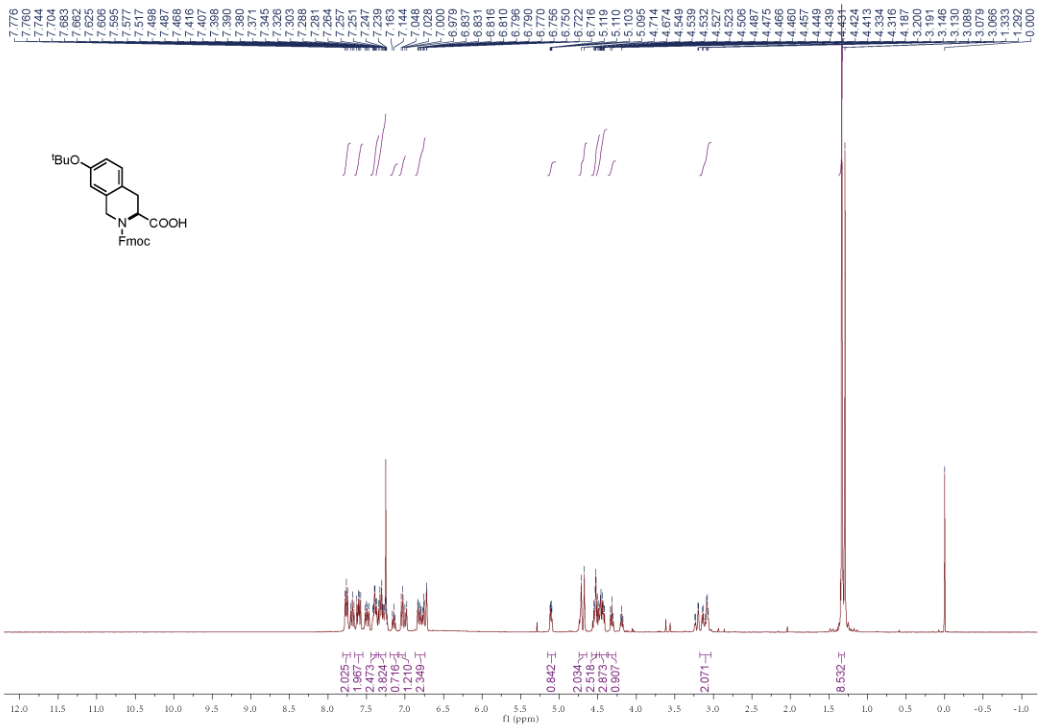

^{13}C NMR of Fmoc–Tic(*p*O^{t}Bu)–OH (101 MHz, $CDCl_3$)

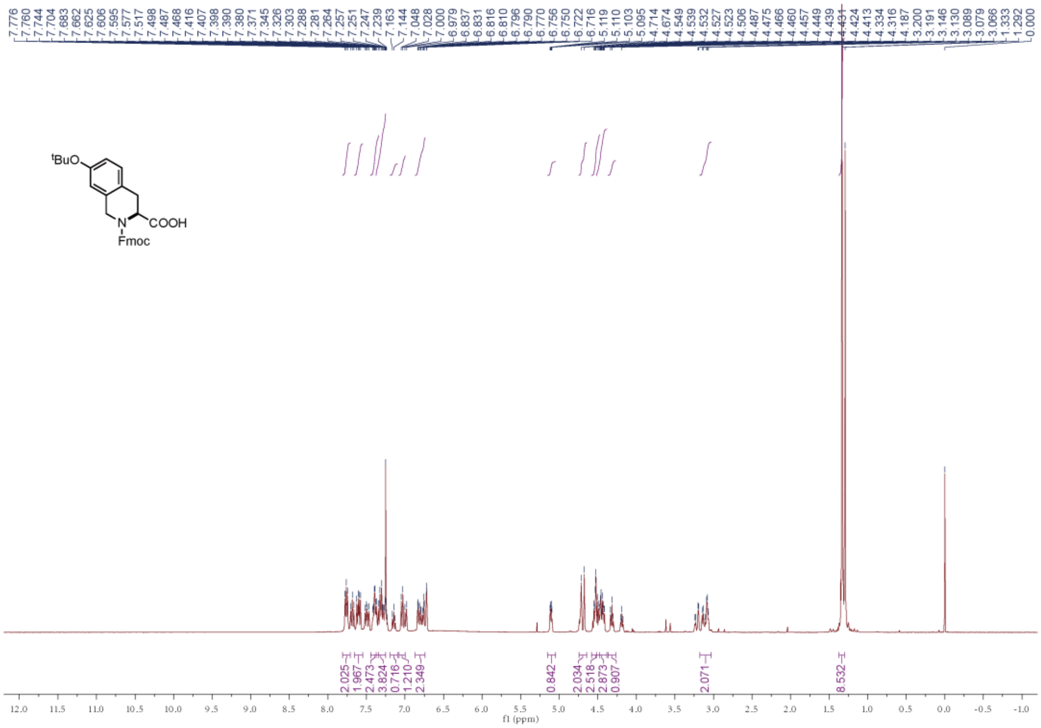

^{1}H NMR of Fmoc–Leu–$CSNH_2$ (400 MHz, $CDCl_3$)

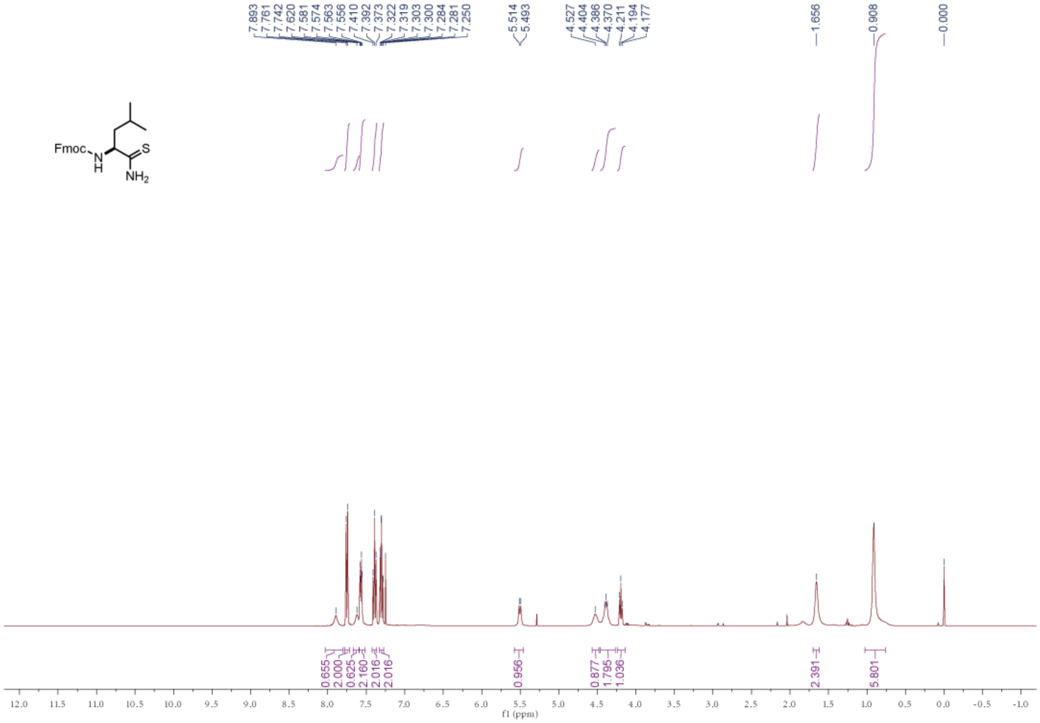

^{13}C NMR of Fmoc–Leu–$CSNH_2$ (101 MHz, $CDCl_3$)

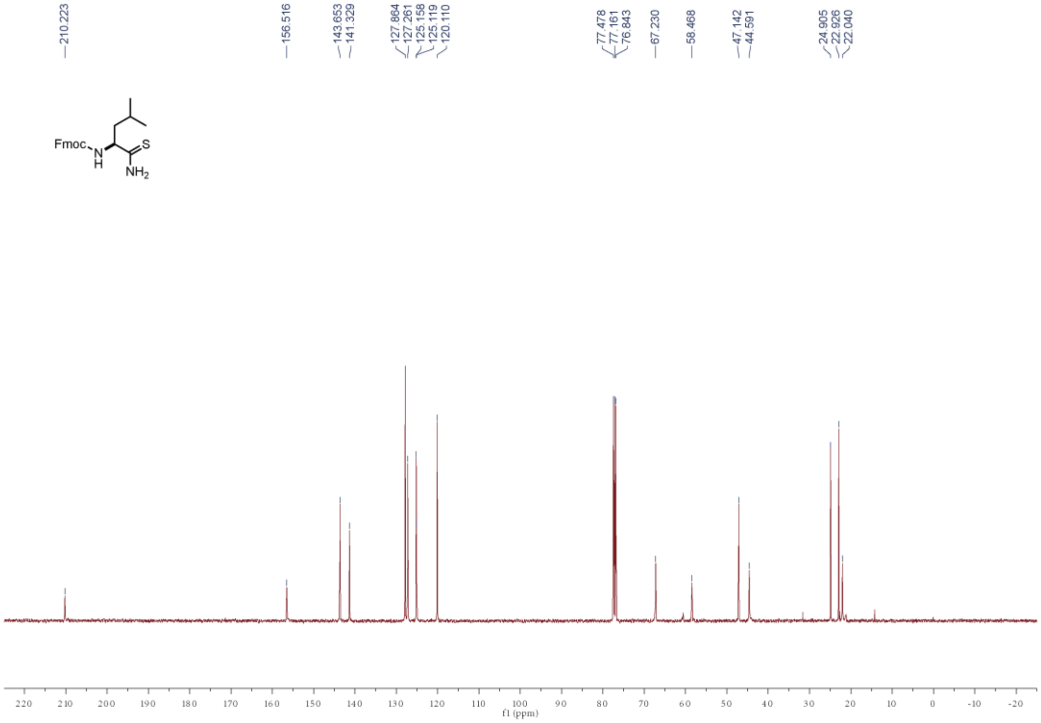

^{1}H NMR of Fmoc–Leu–Thz–OMe (400 MHz, $CDCl_3$)

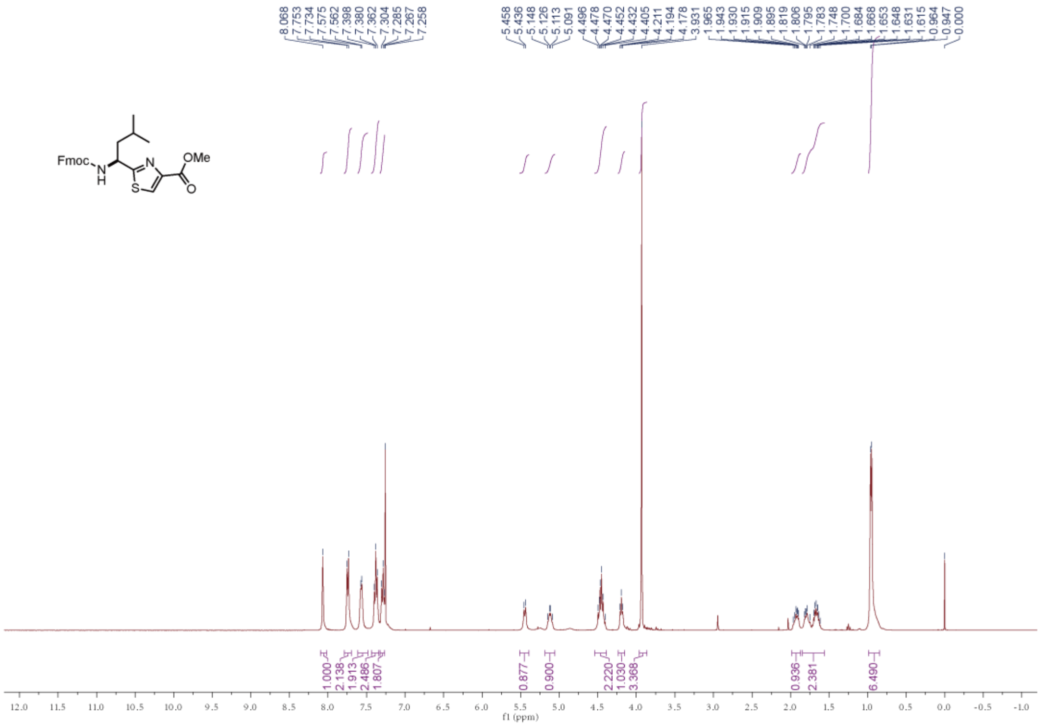

^{13}C NMR of Fmoc–Leu–Thz–OMe (101 MHz, $CDCl_3$)

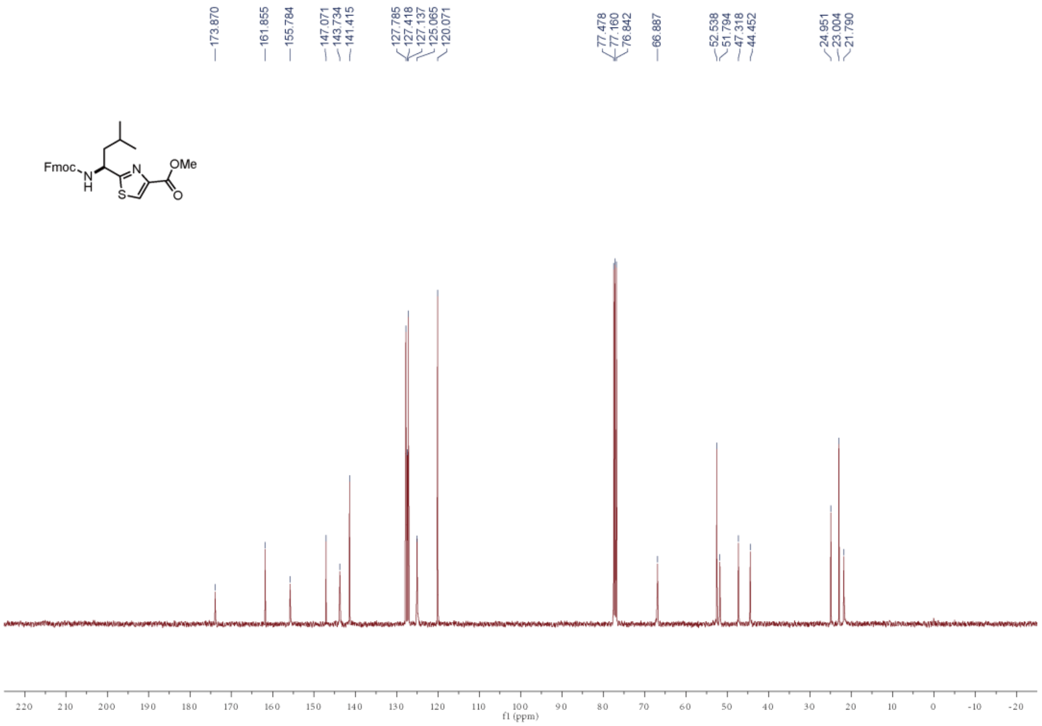

^{1}H NMR of Fmoc–Leu–Thz–OH (400 MHz, $CDCl_3$)

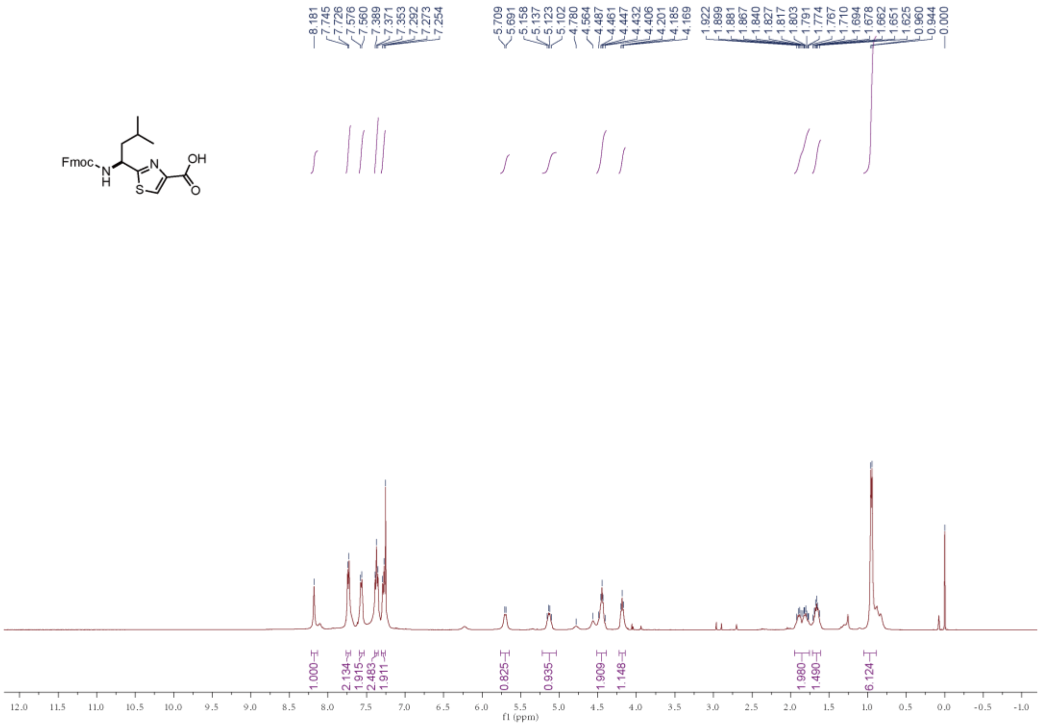

^{13}C NMR of Fmoc–Leu–Thz–OH (101 MHz, $CDCl_3$)

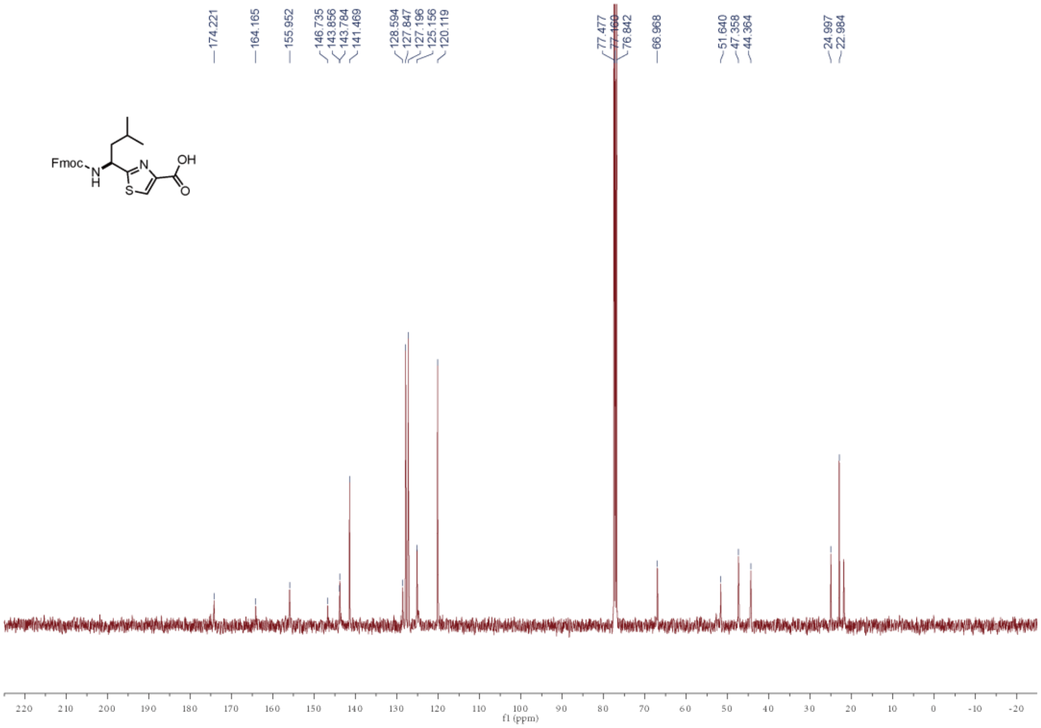

^{1}H NMR of Fmoc–Val–$CSNH_2$ (400 MHz, $CDCl_3$)

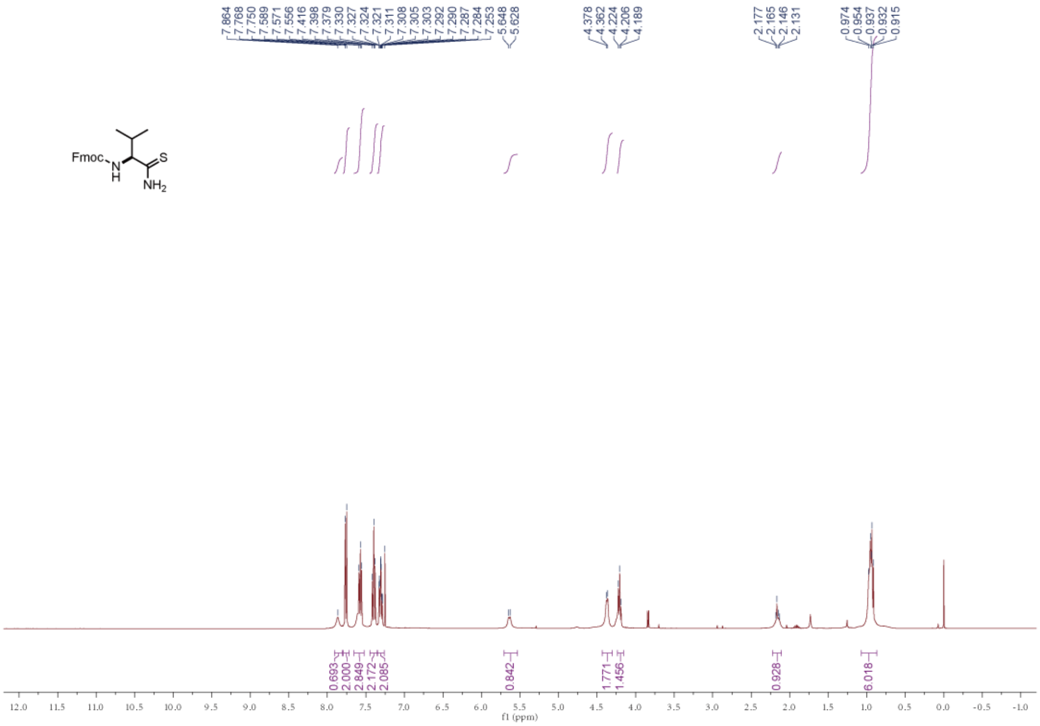

^{13}C NMR of Fmoc–Val–$CSNH_2$ (101 MHz, $CDCl_3$)

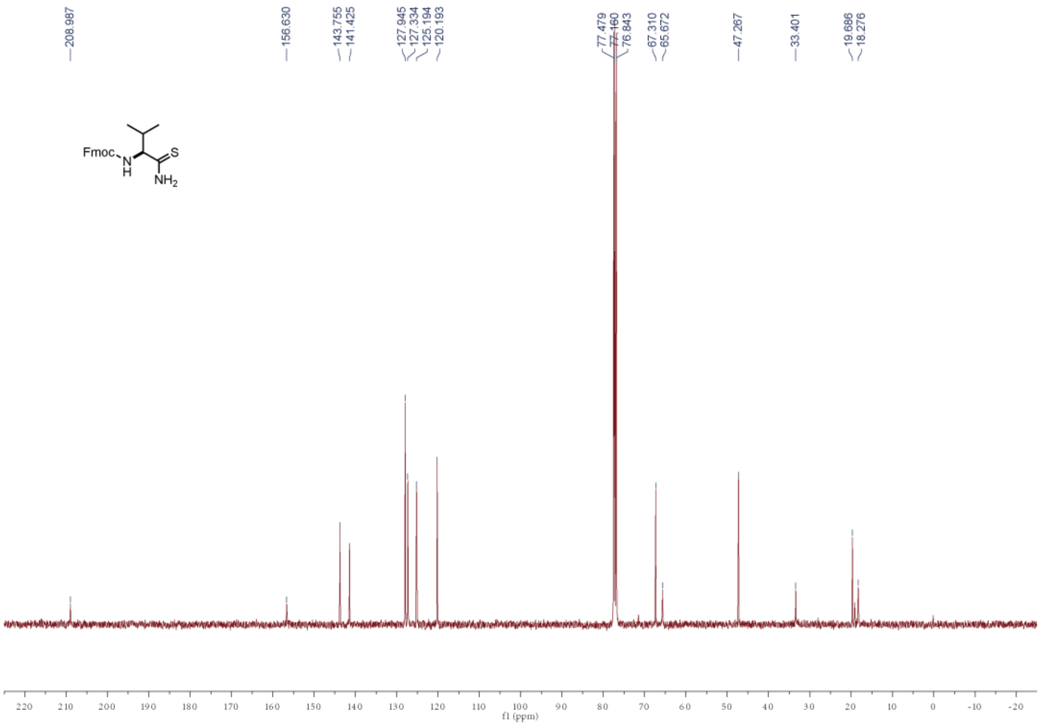

^{1}H NMR of Fmoc–Val–Thz–OMe (400 MHz, $CDCl_3$)

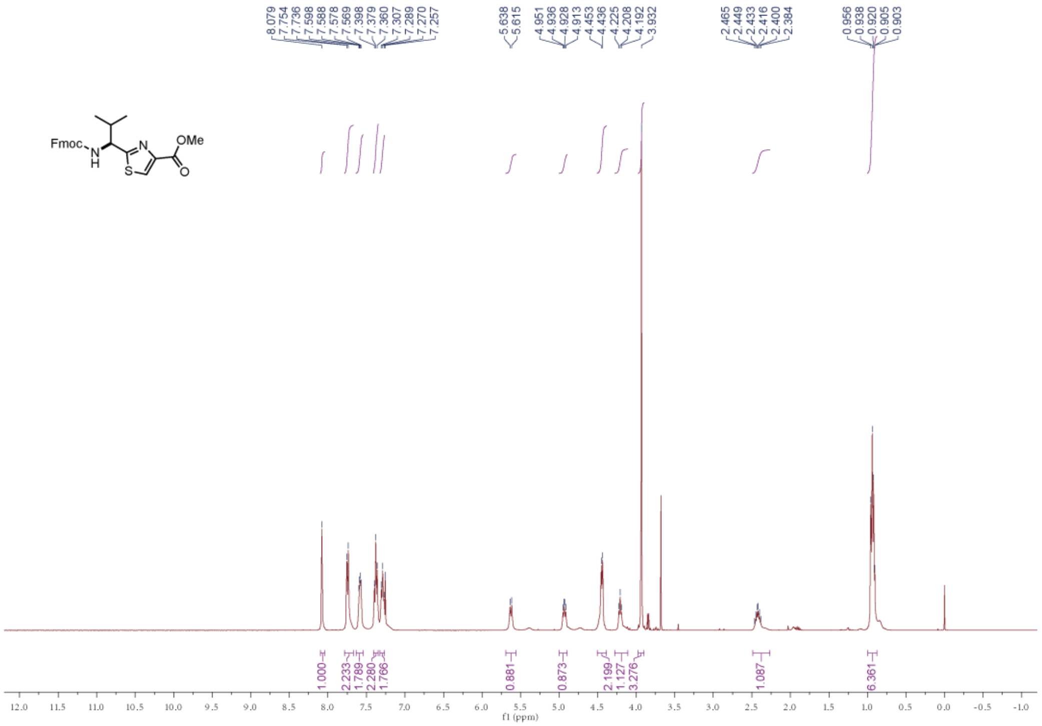

^{13}C NMR of Fmoc–Val–Thz–OMe (101 MHz, $CDCl_3$)

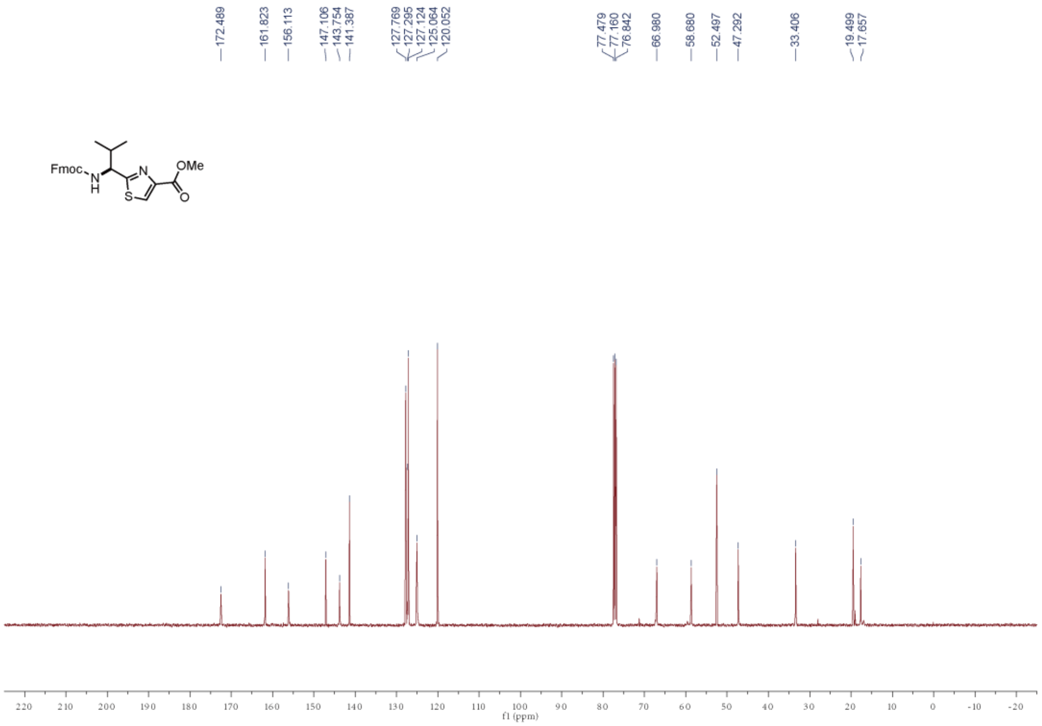

^{1}H NMR of Fmoc–Val–Thz–OH (400 MHz, $CDCl_3$)

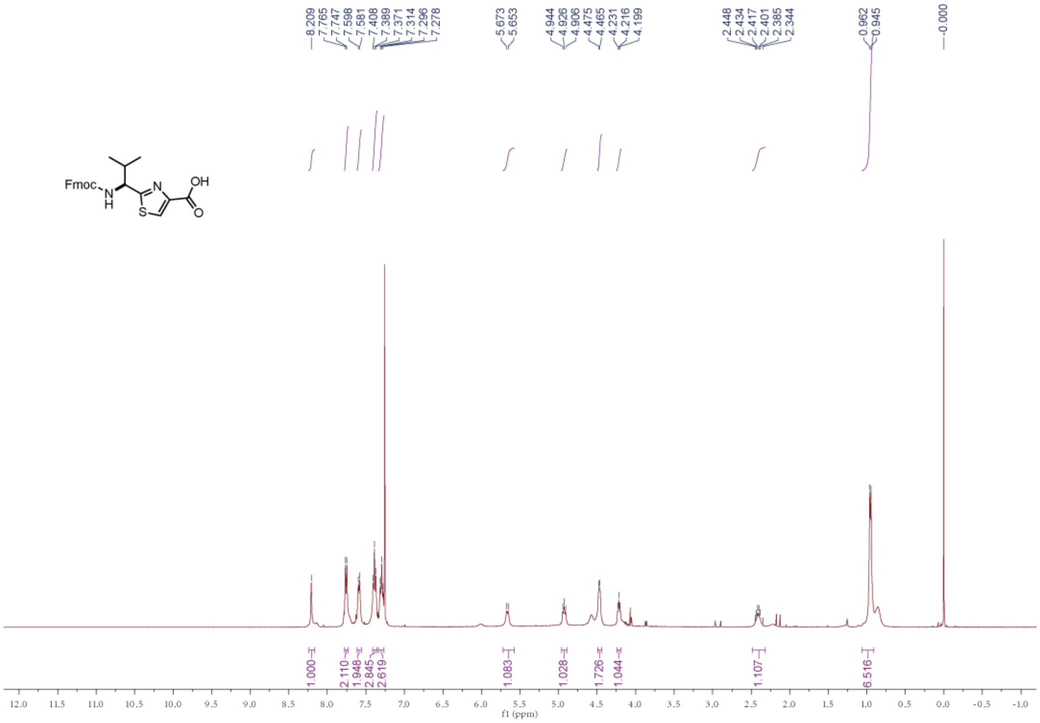

^{13}C NMR of Fmoc–Val–Thz–OH (101 MHz, $CDCl_3$)

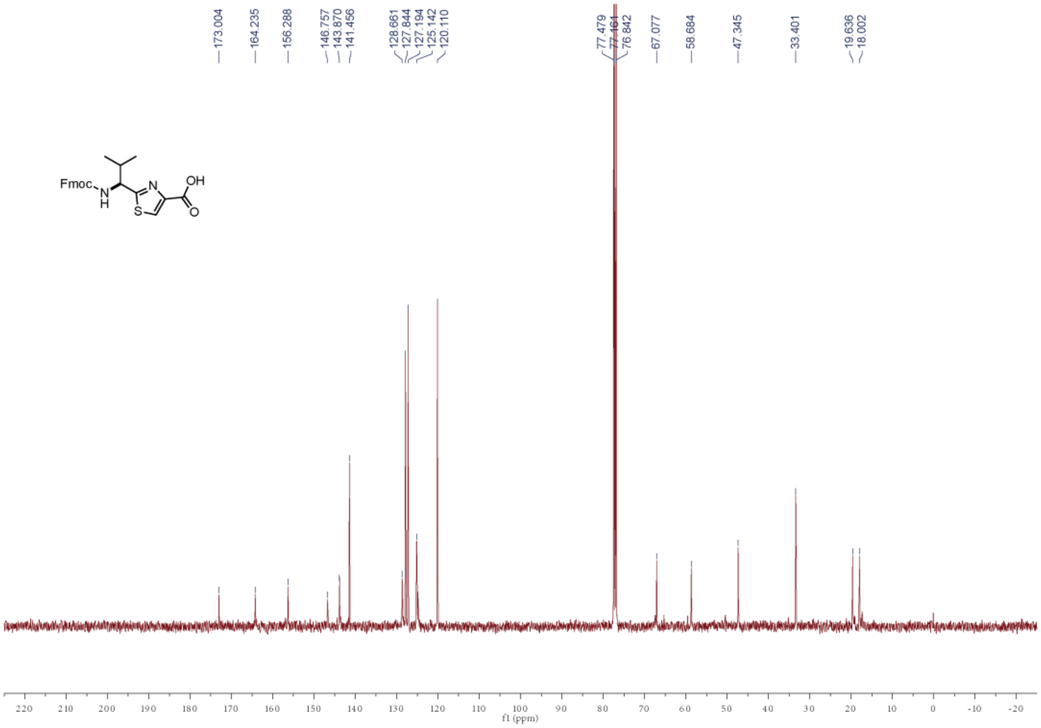

^{1}H NMR of Fmoc–Ser(O^{t}Bu)–$CSNH_2$ (400 MHz, $CDCl_3$)

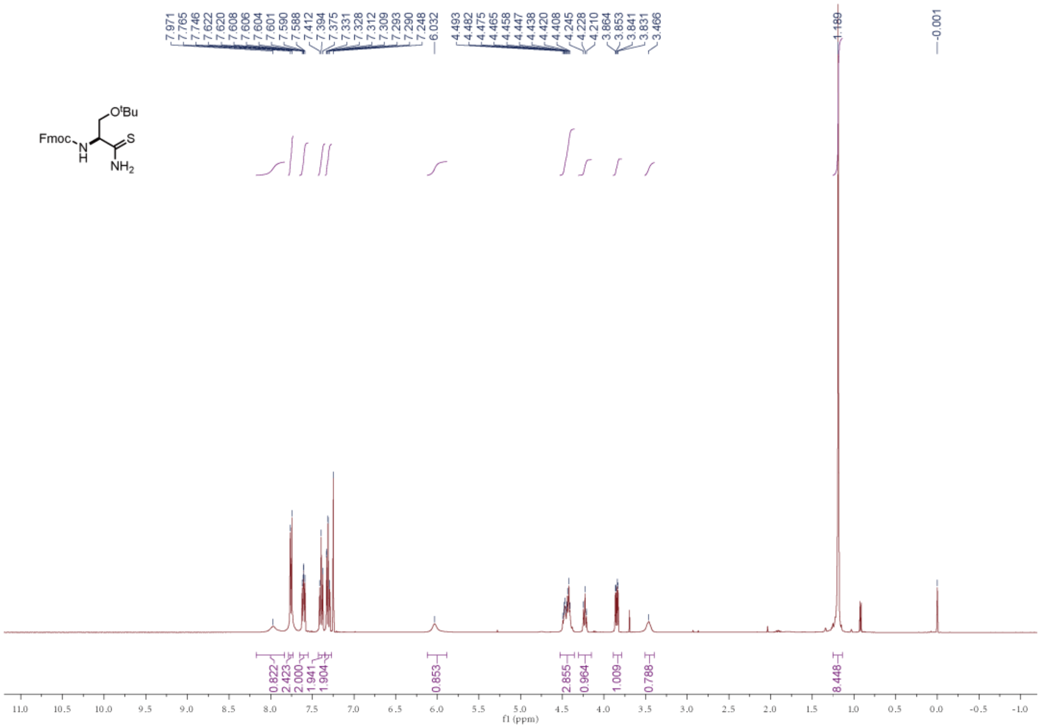

^{13}C NMR of Fmoc–Ser(O^{t}Bu)–$CSNH_2$ (101 MHz, $CDCl_3$)

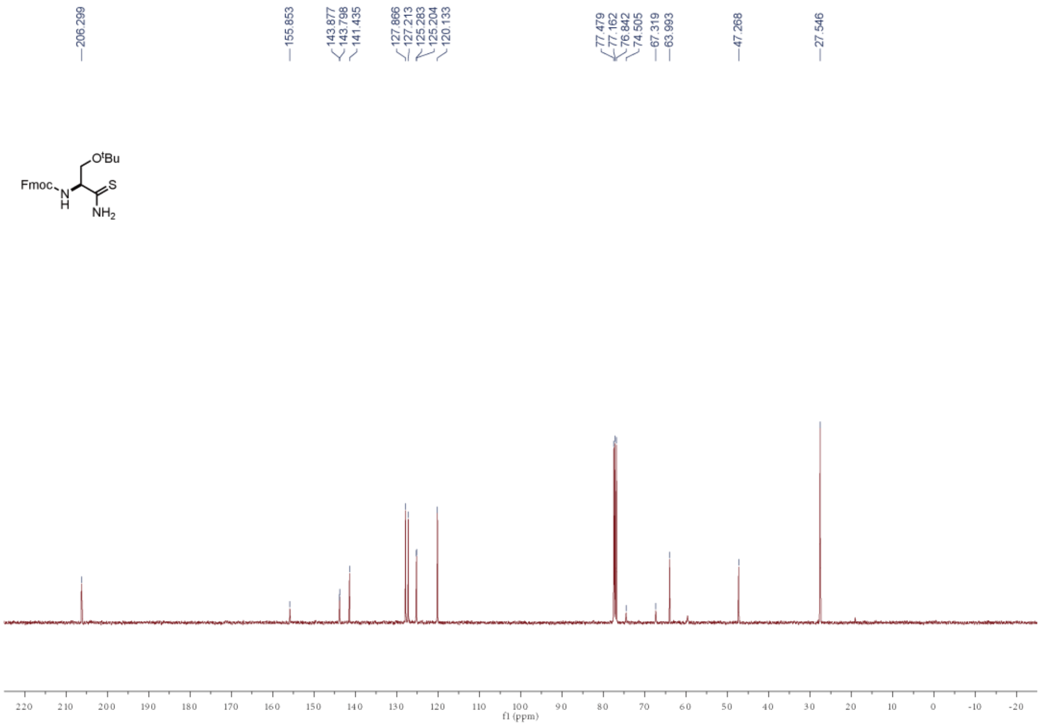

^{1}H NMR of Fmoc–Ser(O^{t}Bu)–Thz–OMe (400 MHz, $CDCl_3$)

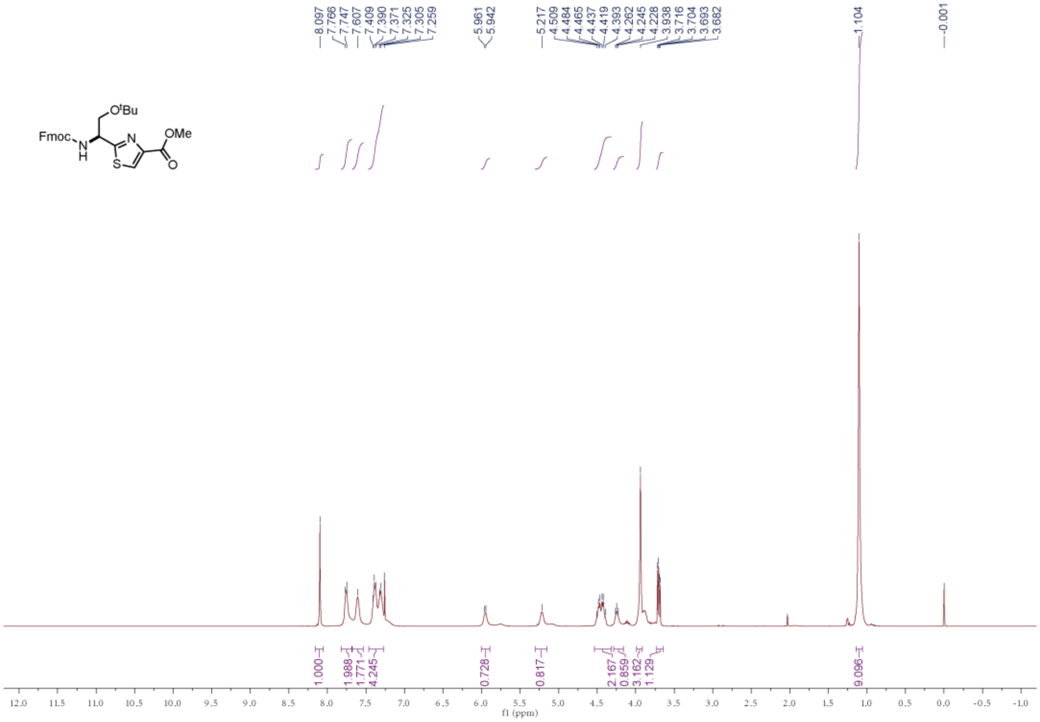

^{13}C NMR of Fmoc–Ser(O^{t}Bu)–Thz–OMe (101 MHz, $CDCl_3$)

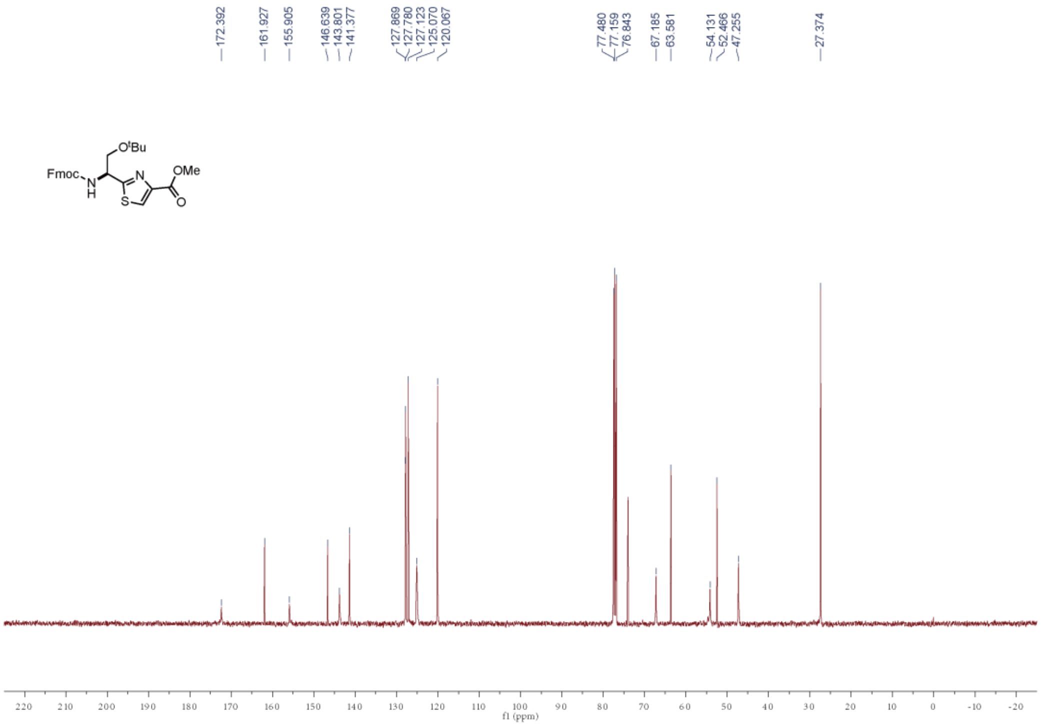

^{1}H NMR of Fmoc–Ser(O^{t}Bu)–Thz–OH (400 MHz, $CDCl_3$)

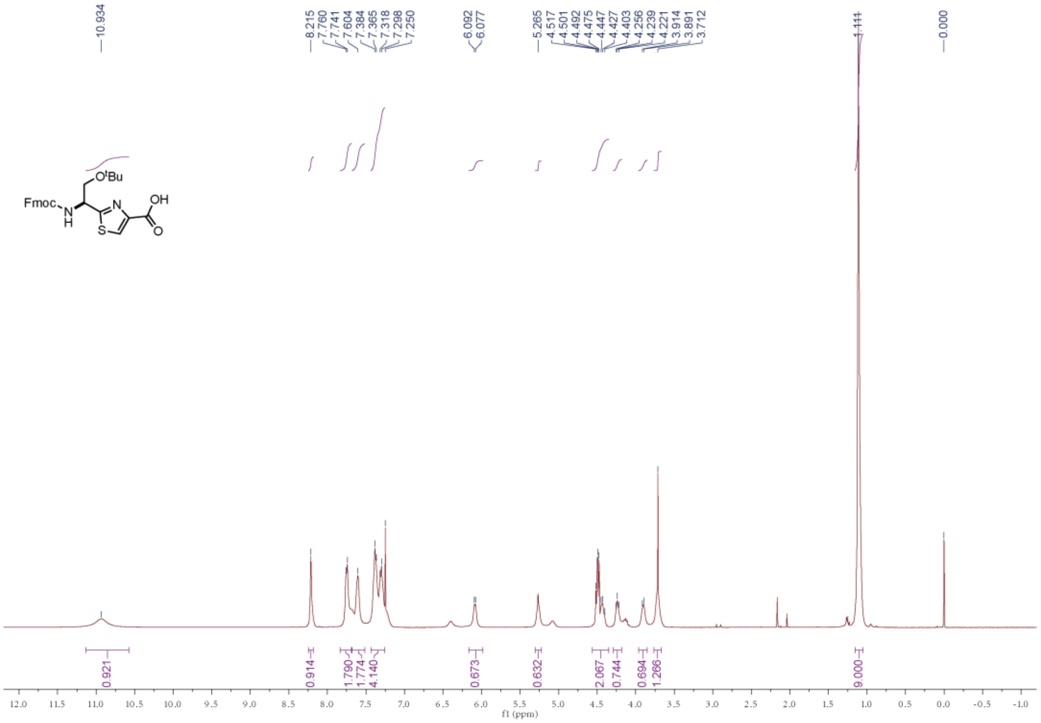

^{13}C NMR of Fmoc–Ser(O^{t}Bu)–Thz–OH (101 MHz, $CDCl_3$)

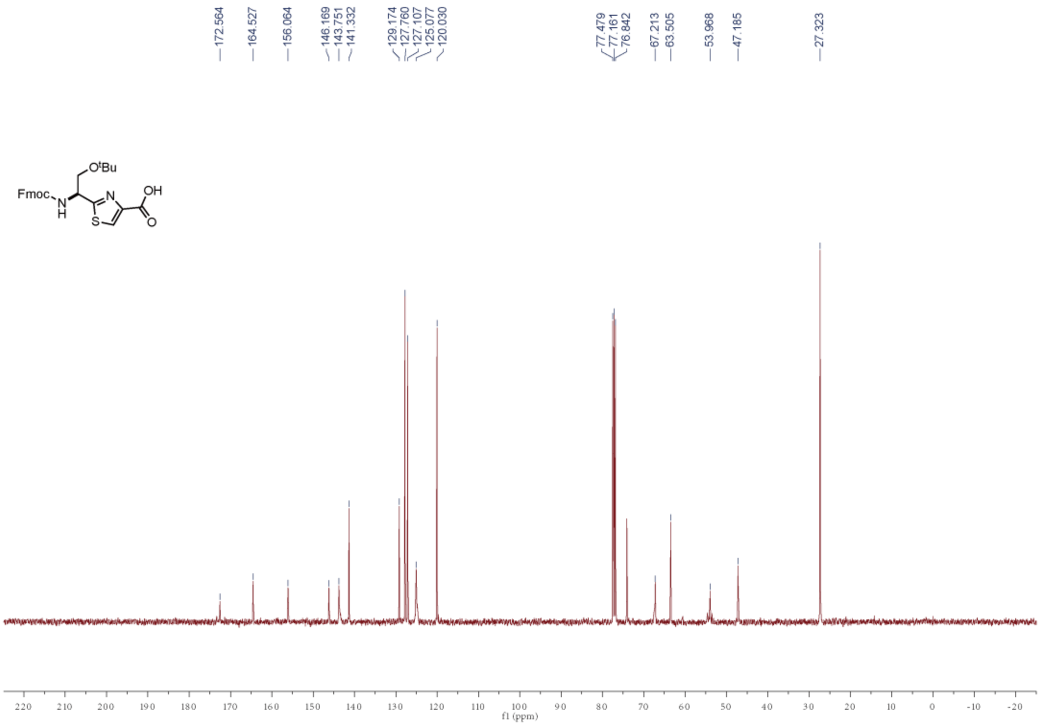

^{1}H NMR of Fmoc–$^{(R,S)}$Abu($^{\beta}$SePh)–CSNH$_2$ (400 MHz, $CDCl_3$)

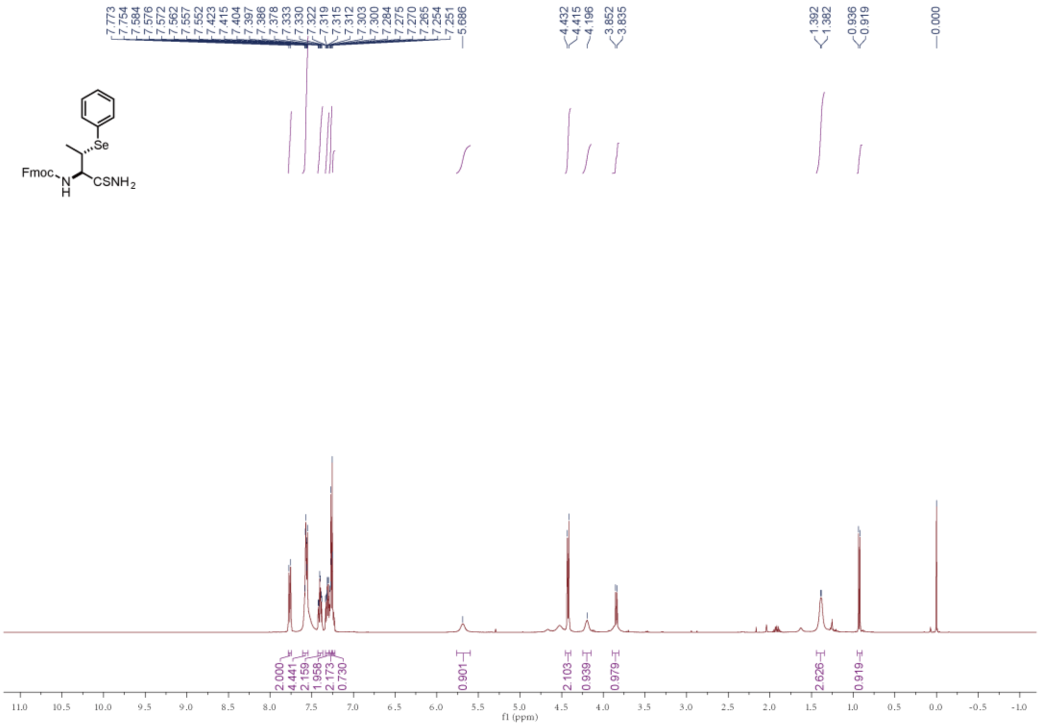

^{13}C NMR of Fmoc–$^{(R,S)}$Abu($^{\beta}$SePh)–CSNH$_2$ (101 MHz, $CDCl_3$)

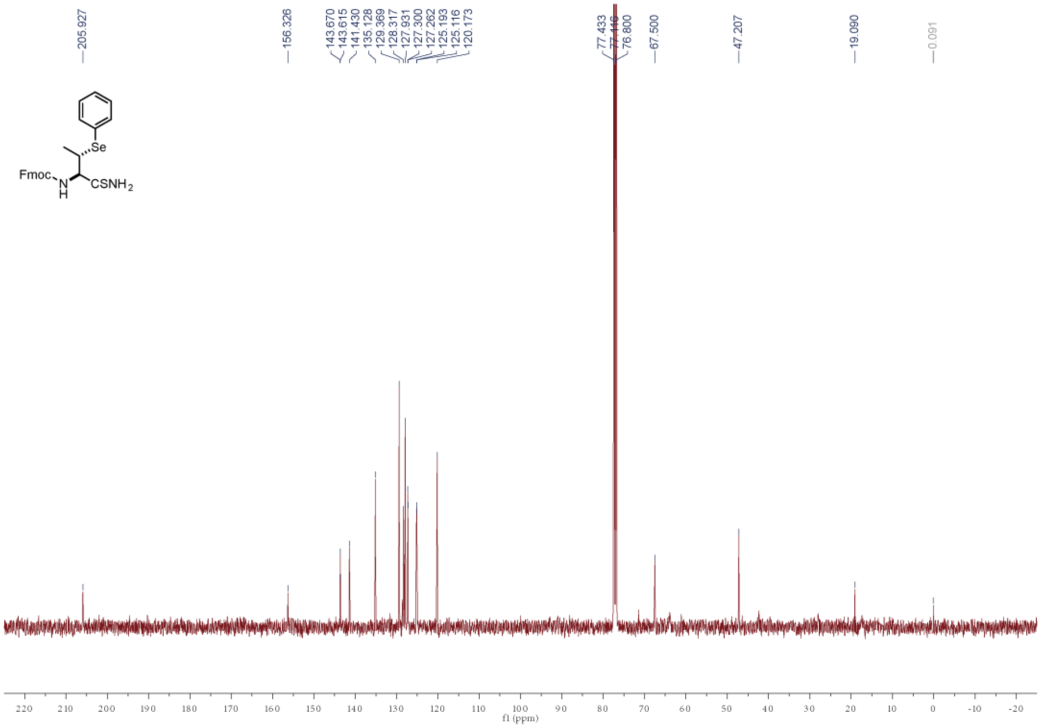

^{1}H NMR of Fmoc–$^{(R,S)}$Abu($^{\beta}$SePh)–Thz–OMe (400 MHz, $CDCl_3$)

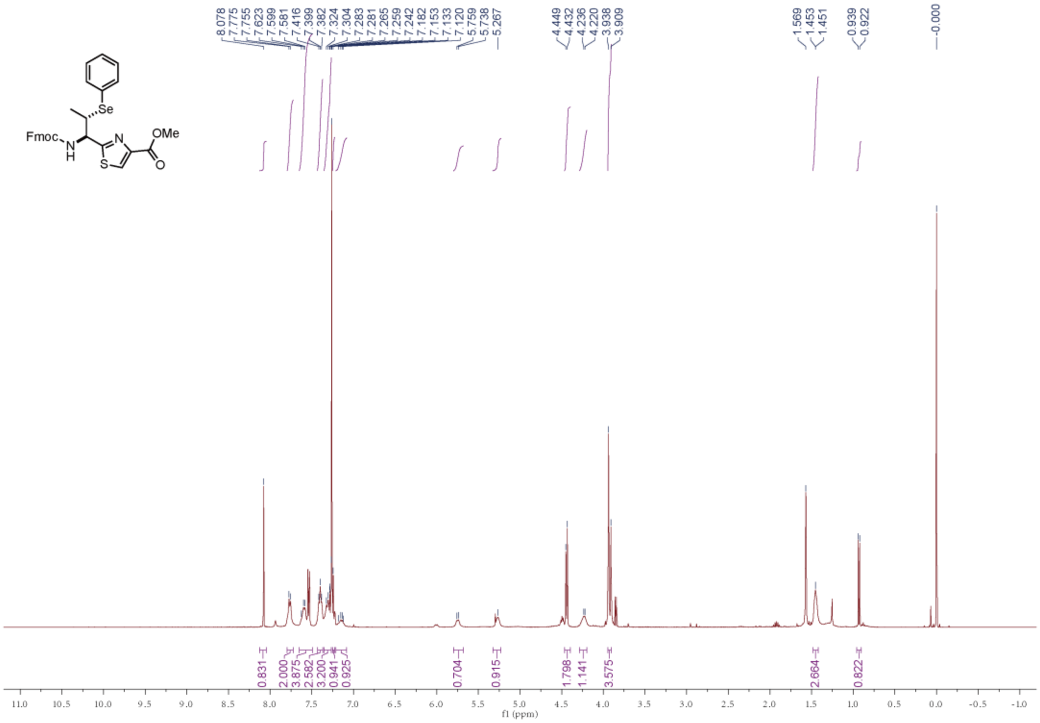

^{13}C NMR of Fmoc–$^{(R,S)}$Abu($^{\beta}$SePh)–Thz–OMe (101 MHz, $CDCl_3$)

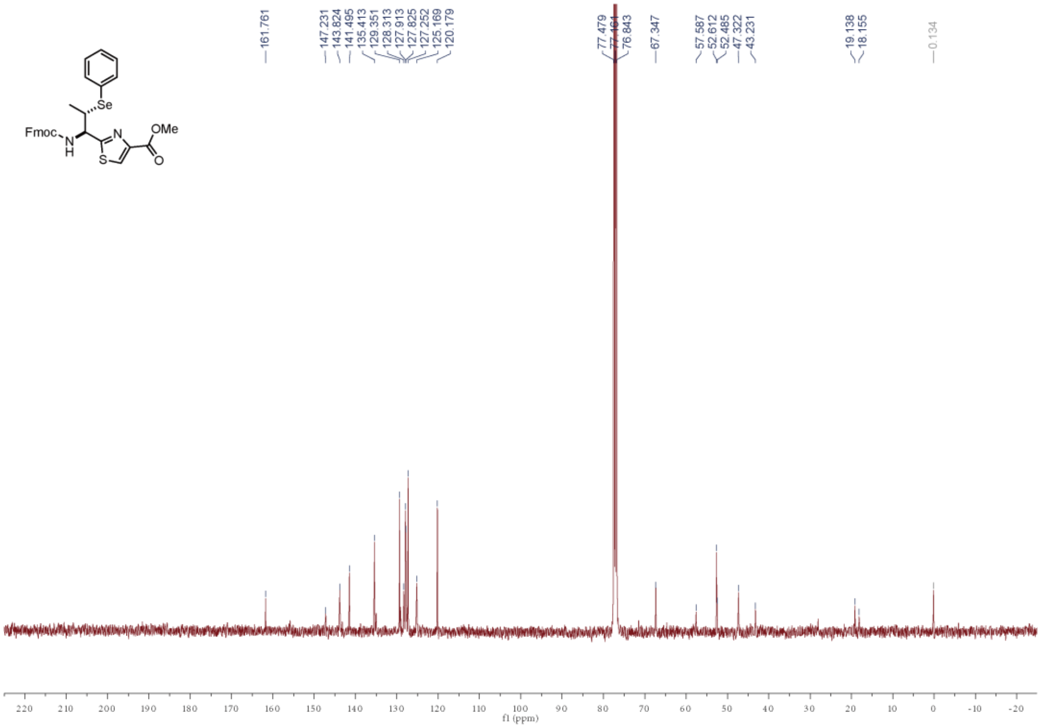

^{1}H NMR of Fmoc–$^{(R,S)}$Abu($^{\beta}$SePh)–Thz–OH (400 MHz, $CDCl_3$)

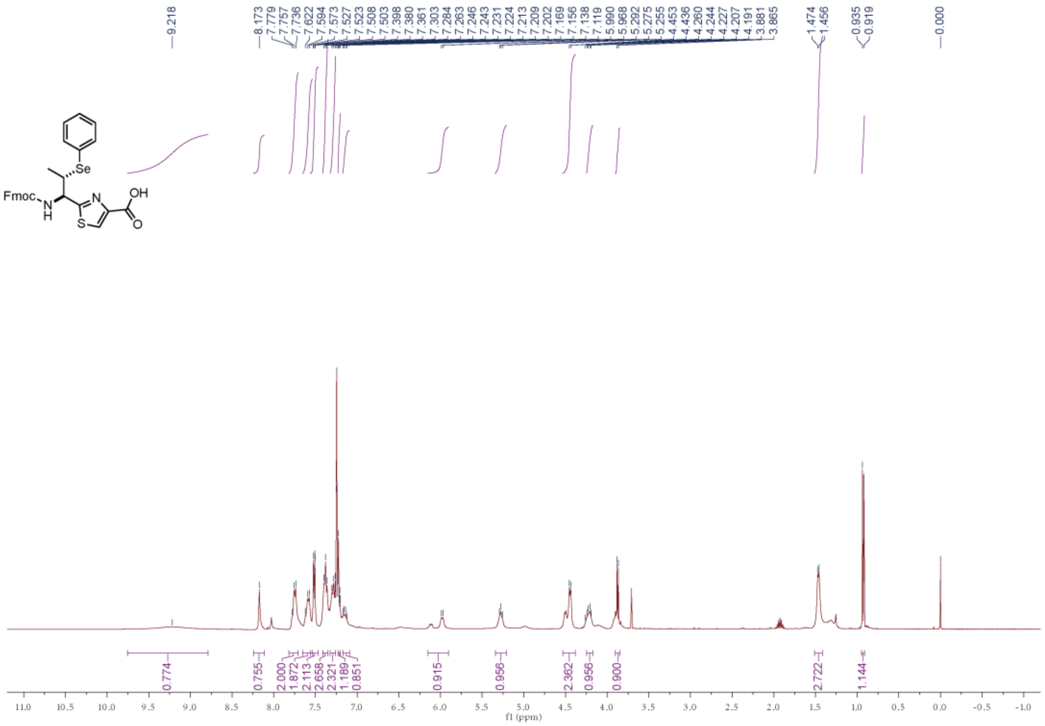

^{13}C NMR of Fmoc–$^{(R,S)}$Abu($^{\beta}$SePh)–Thz–OH (101 MHz, $CDCl_3$)

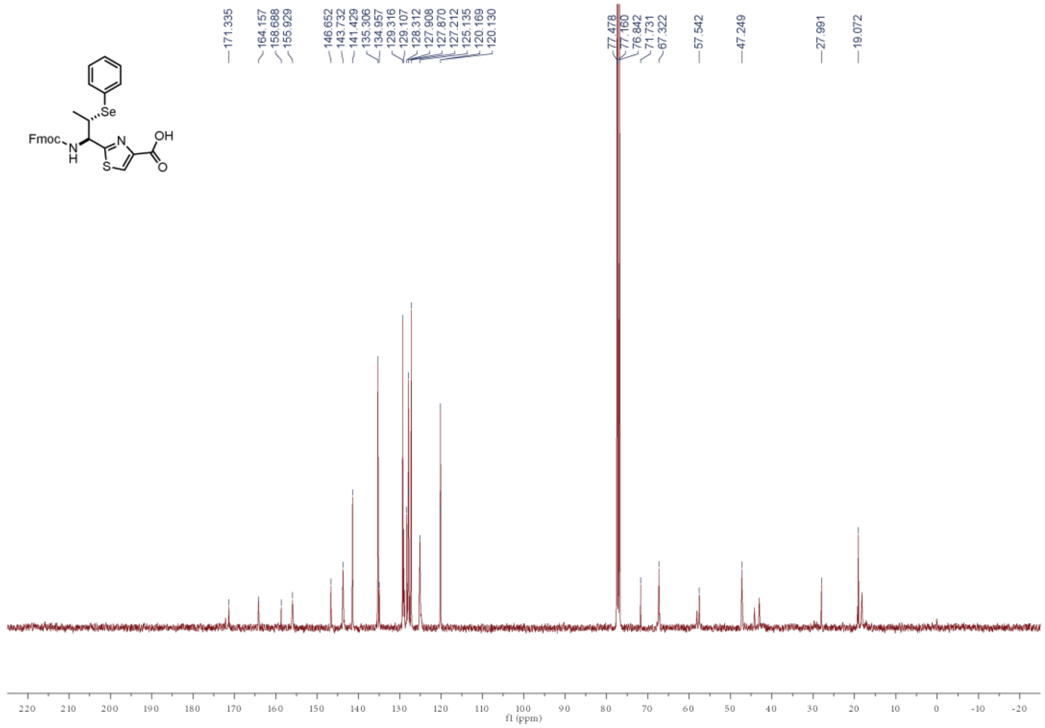

^{1}H NMR of thiopeptide hTP4 (400 MHz, DMSO-D_6)

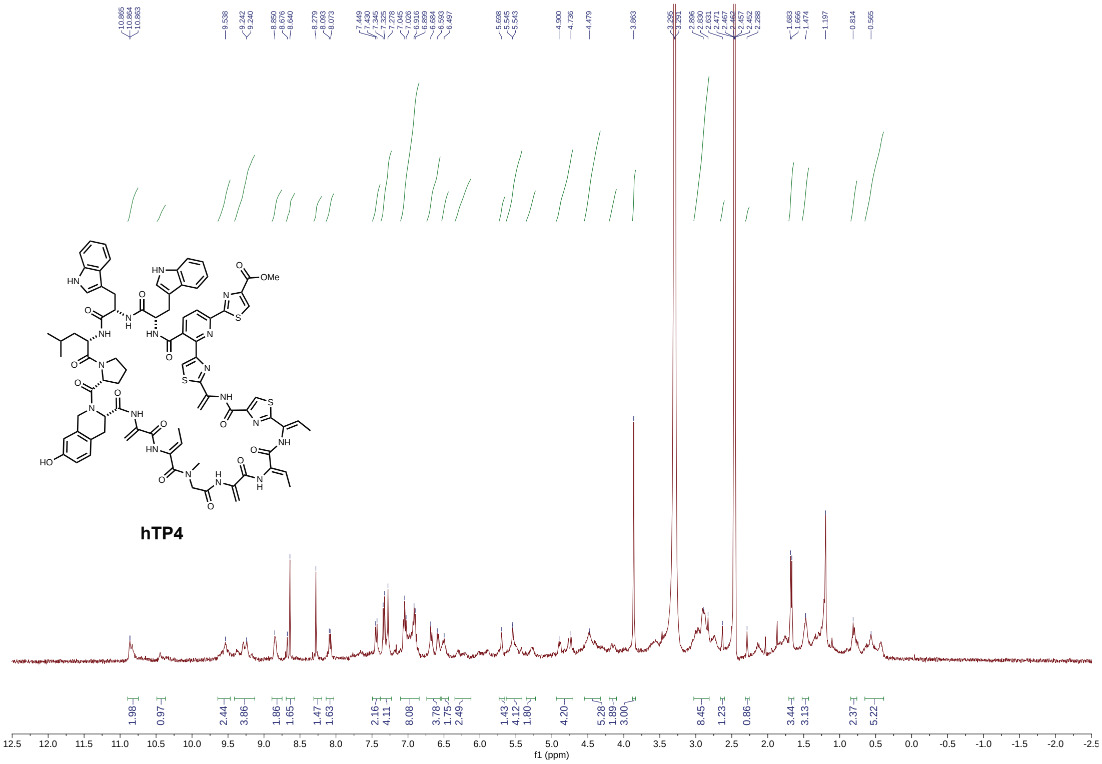

^{1}H NMR of thiopeptide hTP6 (400 MHz, DMSO-D_6)

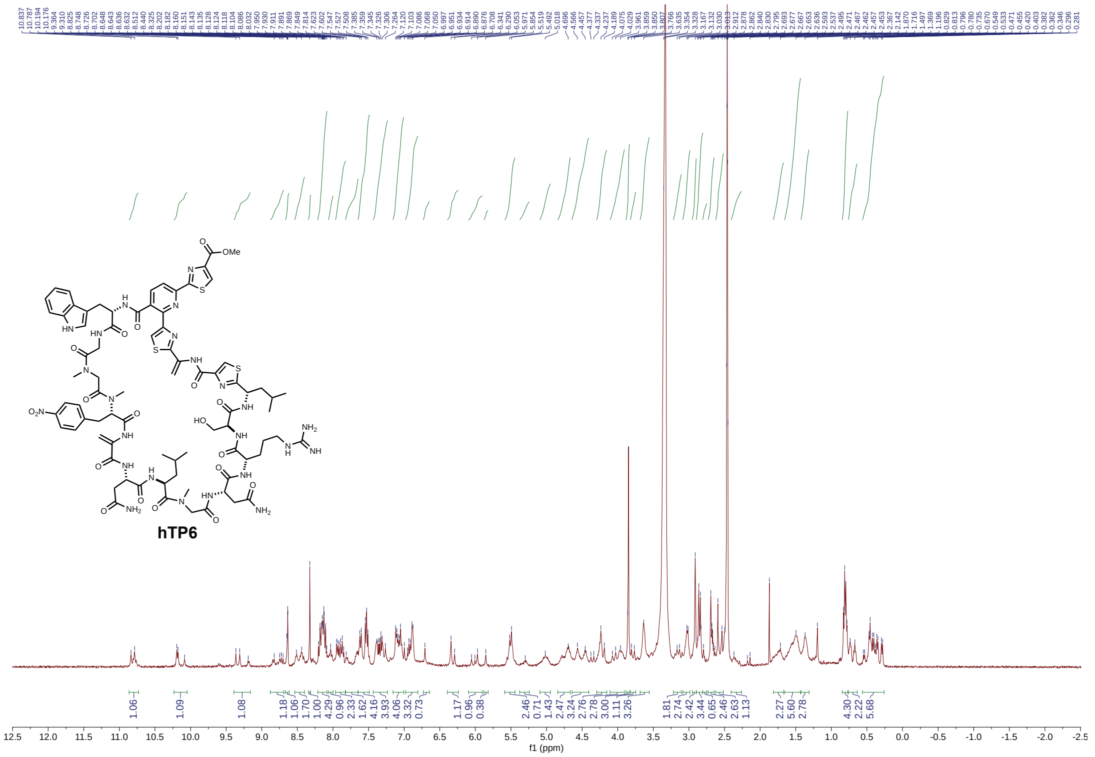

^{1}H NMR of thiopeptide hTP8 (400 MHz, DMSO-D_6)

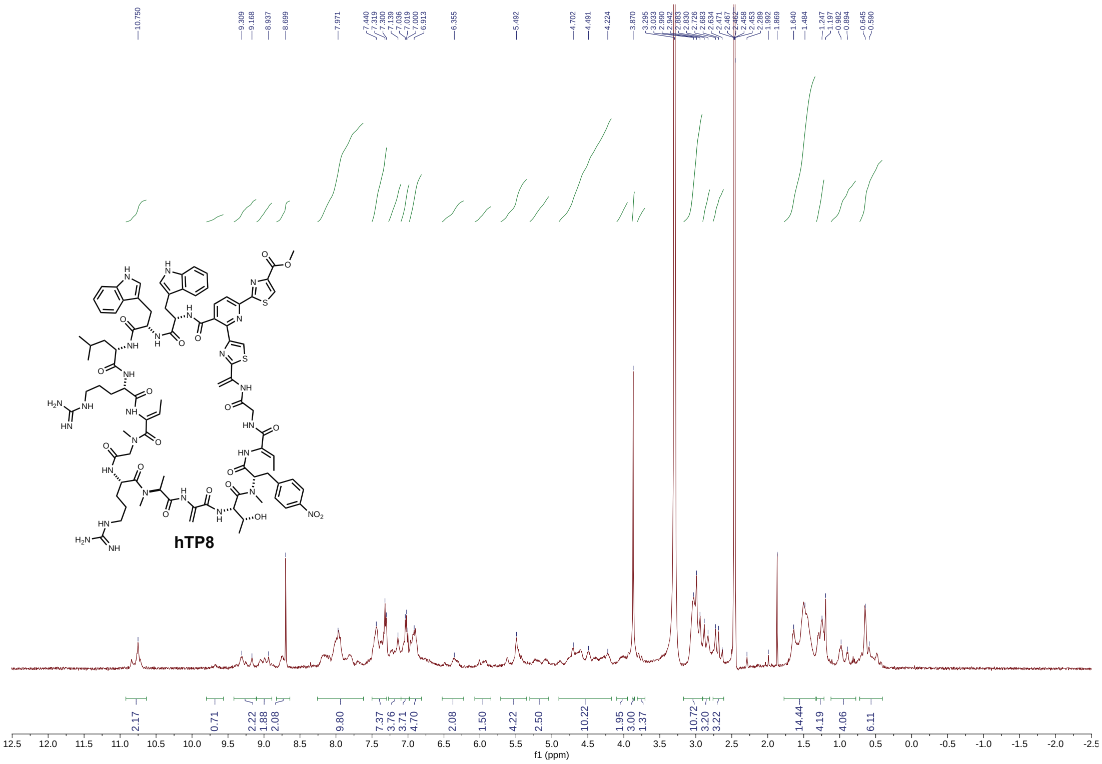

^{1}H NMR of thiopeptide hTP13 (400 MHz, DMSO-D_6)

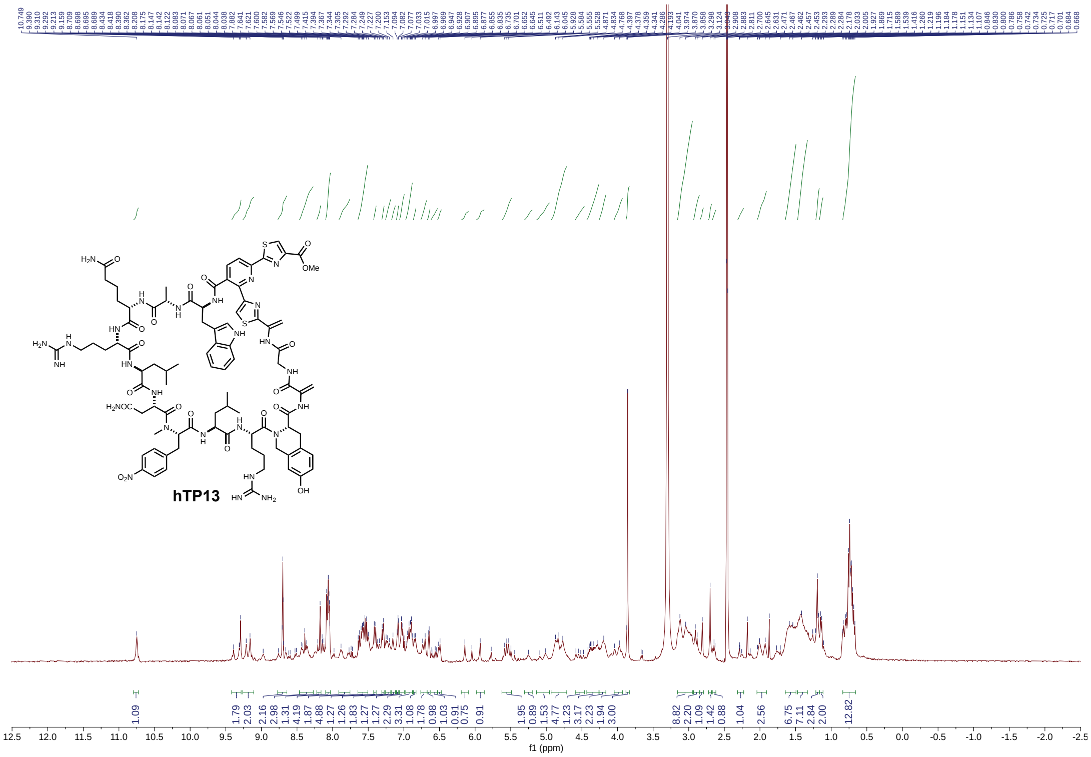

^{1}H NMR of thiopeptide wTP3 (400 MHz, DMSO-D_6)

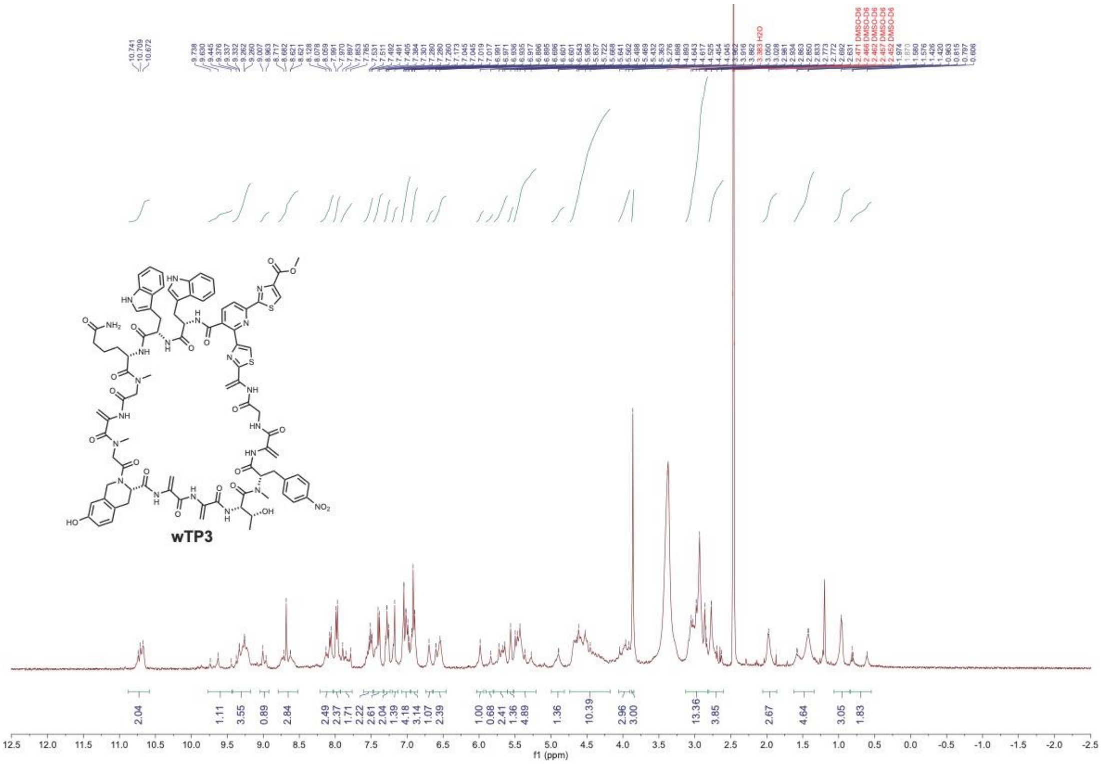

^{1}H NMR of thiopeptide wTP12 (400 MHz, DMSO-D_6)

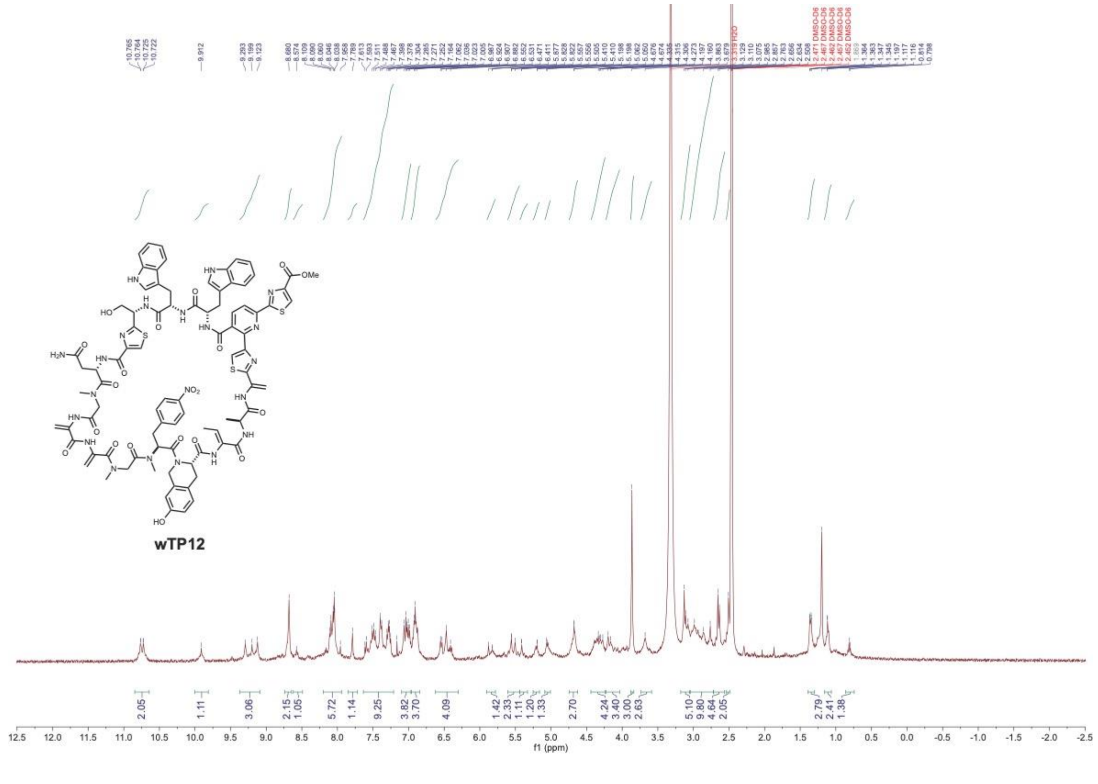

Supplement: Supplementary file 1 — ja3c12037_si_001.pdf [file ja3c12037_si_001.pdf]
